# Mapping the developing human immune system across organs*

**DOI:** 10.1126/science.abo0510

**Published:** 2022-06-03

**Authors:** Chenqu Suo, Emma Dann, Issac Goh, Laura Jardine, Vitalii Kleshchevnikov, Jong-Eun Park, Rachel A. Botting, Emily Stephenson, Justin Engelbert, Zewen Kelvin Tuong, Krzysztof Polanski, Nadav Yayon, Chuan Xu, Ondrej Suchanek, Rasa Elmentaite, Cecilia Domínguez Conde, Peng He, Sophie Pritchard, Mohi Miah, Corina Moldovan, Alexander S. Steemers, Pavel Mazin, Martin Prete, Dave Horsfall, John C. Marioni, Menna R. Clatworthy, Muzlifah Haniffa, Sarah A. Teichmann

**Affiliations:** 1 Wellcome Sanger Institute; Wellcome Genome Campus, Hinxton, Cambridge, UK; 2 Department of Paediatrics, Cambridge University Hospitals; Hills Road, Cambridge, UK; 3 Biosciences Institute, Newcastle University; Newcastle upon Tyne, UK; 4 Haematology Department, Freeman Hospital; Newcastle upon Tyne Hospitals NHS Foundation Trust, Newcastle upon Tyne, UK; 5 Graduate School of Medical Science and Engineering, Korea Advanced Institute of Science and Technology (KAIST); Daejeon, Korea; 6 Molecular Immunity Unit, University of Cambridge Department of Medicine; Cambridge, UK; 7 European Molecular Biology Laboratory European Bioinformatics Institute; Hinxton, Cambridge, UK; 8 Department of Cellular Pathology, Newcastle upon Tyne Hospitals NHS Foundation Trust; Newcastle upon Tyne, UK; 9 Cancer Research UK Cambridge Institute, Li Ka Shing Centre, University of Cambridge; Cambridge, UK; 10 Department of Dermatology and NIHR Newcastle Biomedical Research Centre, Newcastle upon Tyne Hospitals NHS Foundation Trust; Newcastle upon Tyne, UK; 11 Theory of Condensed Matter, Cavendish Laboratory, Department of Physics, University of Cambridge; Cambridge, UK

## Abstract

Single-cell genomics studies have decoded the immune-cell composition of several human prenatal organs but were limited in understanding the developing immune system as a distributed network across tissues. We profiled nine prenatal tissues combining single-cell RNA sequencing, antigen-receptor sequencing, and spatial transcriptomics to reconstruct the developing human immune system. This revealed the late acquisition of immune effector functions by myeloid and lymphoid cell subsets and the maturation of monocytes and T cells prior to peripheral tissue seeding. Moreover, we uncovered system-wide blood and immune cell development beyond primary hematopoietic organs, characterized human prenatal B1 cells, and shed light on the origin of unconventional T cells. Our atlas provides both valuable data resources and biological insights that will facilitate cell engineering, regenerative medicine, and disease understanding.

The human immune system develops across several anatomical sites throughout gestation. Immune cells differentiate initially from extra-embryonic yolk sac progenitors and subsequently from aorto-gonad-mesonephros-derived hematopoietic stem cells (HSCs), before liver and bone marrow take over as the primary sites of hematopoiesis (1, 2). Immune cells from these primary hematopoietic sites seed developing lymphoid organs such as the thymus, spleen, and lymph nodes, as well as peripheral non-lymphoid organs.

Recent advances in single-cell genomics technologies have revolutionized our understanding of the developing human organs (3–11). However, these studies have focused on one or a few organs, rather than reconstructing the entire immune system as a distributed network across all organs. Such a holistic understanding of the developing human immune system would have far-reaching implications for health and disease including cellular engineering, regenerative medicine, and a deeper mechanistic understanding of congenital disorders affecting the immune system.

Here we present a cross-tissue single-cell and spatial atlas of developing human immune cells across prenatal hematopoietic (yolk sac, liver, and bone marrow), lymphoid (thymus, spleen, and lymph node), and non-lymphoid peripheral organs (skin, kidney, and gut) to provide a detailed characterization of generic and tissue-specific properties of the developing immune system. We generated single-cell RNA sequencing (scRNA-seq) data from yolk sac, prenatal spleen, and skin, and integrated publicly available cell atlases of six additional organs, spanning weeks 4 to 17 post-conception (3, 4, 7, 8, 10, 11). We also generated single-cell γδ T cell receptor (γδTCR) sequencing data and additional αβ T cell receptor (αβTCR) and B cell receptor (BCR) sequencing data. Finally, we integrated the single-cell transcriptome profiles with in situ tissue location using spatial transcriptomics.

This study reveals the acquisition of immune effector functions of myeloid and lymphoid lineages from the second trimester, the maturation of developing monocytes and T cells prior to peripheral tissue seeding, and system-wide blood and immune cell development during human prenatal development. Moreover, we identify, characterize, and functionally validate the properties of human prenatal B1 cells and the origin of unconventional T cells.

## Integrated cross-organ map of prenatal cell states in distinct tissue microenvironments

**Fig. 1:**
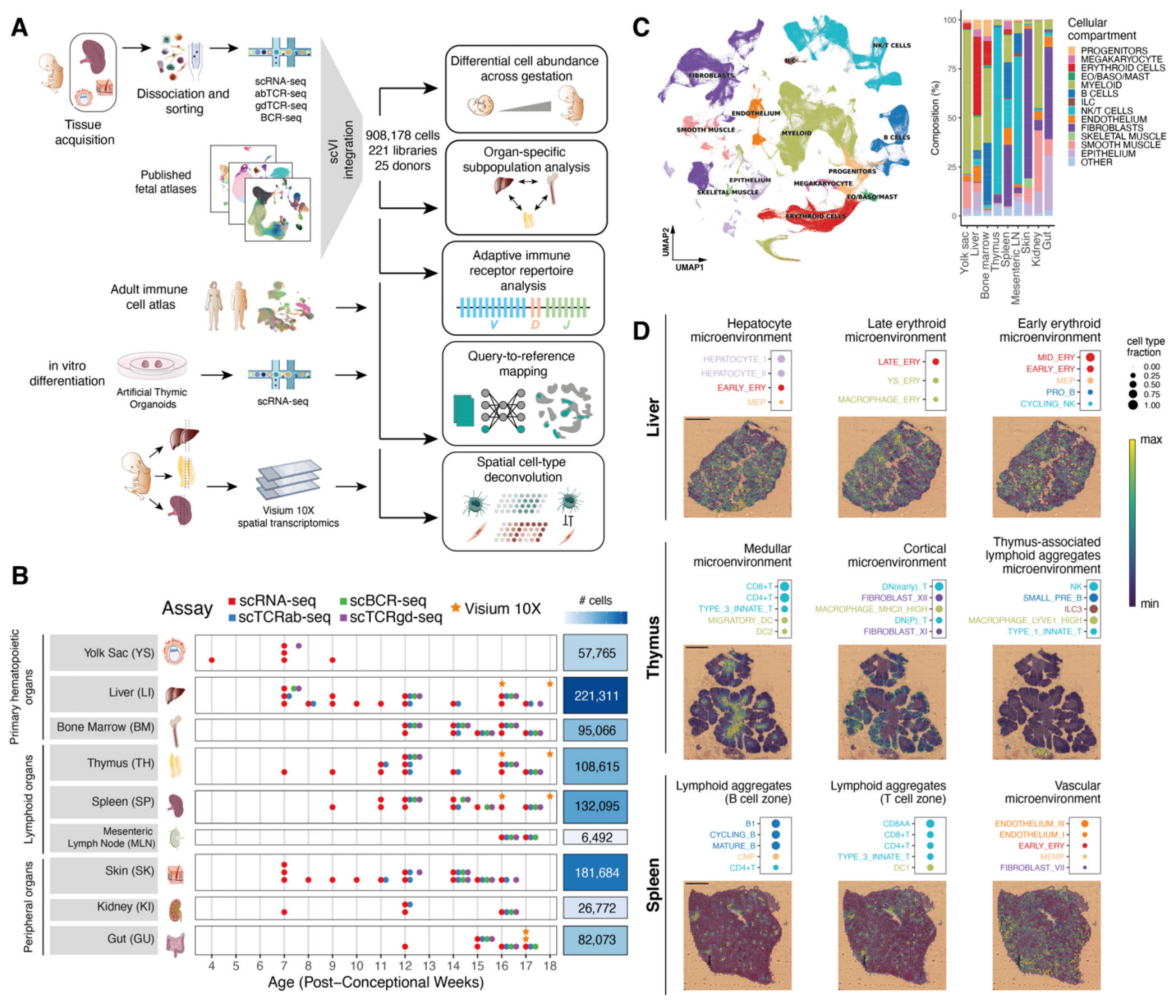
Cross-tissue cellular atlas of the developing human immune system. (A) Overview of study design and analysis pipeline. We generated scRNA-seq and scVDJ-seq data from prenatal spleen, yolk sac and skin which were integrated via scVI with a collection of publicly available single-cell RNA-seq datasets. This cell atlas was used for (i) differential abundance analysis across gestation and organs with Milo, (ii) antigen receptor repertoire analysis with scirpy and dandelion, (iii) comparison with adult immune cells and in vitro differentiated cells with scArches and CellTypist, and (iv) spatial cell type deconvolution on Visium 10X data of hematopoietic and lymphoid organs using cell2location. (B) Summary of analyzed samples by gestational stage (*x*-axis) and organ (*y*-axis). Colors denote the types of molecular assays performed for each sample. The side bar indicates the total number of cells collected for each organ (after quality control). (C) Left: UMAP embedding of scRNA-seq profiles in prenatal tissues (908,178 cells) colored by broad cellular compartments. Right: bar plot of percentage of cells assigned to each broad compartment for each of the profiled organs. Raw cell proportions are adjusted to account for FACS-based CD45 enrichment. The category “Other” denotes clusters annotated as low-quality cells. (Eo/Baso/Mast: eosinophils/basophils/mast cells; ILC: innate lymphoid cells; NK: natural killer cells). (D) Representative colocalization patterns identified with non-negative matrix factorization of spatial cell type abundances estimated with cell2location. For each annotated microenvironment, we show (top) a dot plot of relative contribution of cell types to microenvironment (dot size) and (bottom) spatial locations of microenvironments on tissue slides, with the color representing the weighted contribution of each microenvironment to each spot. Each scale bar indicates a length of 1 mm.

To systematically assess the heterogeneity of immune cell populations across human prenatal hematopoietic organs, lymphoid, and non-lymphoid tissues, we generated scRNA-seq data from prenatal spleen, yolk sac, and skin, which were integrated with a collection of publicly available single-cell datasets from the Human Developmental Cell Atlas initiative (3, 4, 7, 8, 10, 11). In total, our dataset comprised samples from 25 embryos/fetuses between 4 to 17 post-conception weeks (pcw) (Fig. 1A), profiled in 221 scRNA-seq libraries. For 65 of these libraries, paired antigen-receptor sequencing data was available for either αβTCR, γδTCR, or BCR (Fig. 1B). After mapping and preprocessing with a unified pipeline, a total of 908,178 cells were retained after quality control.

To facilitate joint analysis of the data, we integrated all libraries using scVI (12), minimizing protocol- and embryo-associated variation (fig. S1A) while retaining differences between organs.

In keeping with previous single-cell atlases of immune cells of prenatal and adult tissues (3, 11, 13), our data captured the emergence of myeloid and lymphoid lineages, as well as closely linked megakaryocytes (MK) and erythroid and non-neutrophilic granulocyte lineages from hematopoietic progenitors (Fig. 1C and figs. S1B to S3). Linking transcriptional phenotypes to paired antigen receptor sequence expression, we paired αβTCR sequences for 28,739 cells, paired γδTCR sequences for 813 cells, and paired BCR sequences for 14,506 cells (fig. S1C).

We repeated dimensionality reduction and clustering on subsets of cells from different lineages and used marker gene analysis and comparison with existing cell labels to comprehensively annotate cell types across tissues. In total, we defined 127 high-quality cell populations (figs. S4 and S5). Cross-tissue integration enabled the identification of cell populations that were too rare to be resolved through analysis of datasets from individual tissues, such as *AXL* and *SIGLEC6-*expressing dendritic cells (AS DC) (14) and plasma B cells (fig. S4). To facilitate the rapid reuse of our atlas for the analysis of newly collected samples, we made the weights from trained scVI models available to enable mapping of external scRNA-seq datasets using transfer learning with scArches (15).

To study the spatial localizations of the cell populations in an early hematopoietic tissue and lymphoid organs critical for B and T cell development, we profiled developing liver, thymus, and spleen from two donors at 16 and 18 pcw with spatial transcriptomics (10X Genomics Visium). Using our multi-organ scRNA-seq dataset as a reference, we performed spatial cell-type deconvolution with cell2location (16) to map cells in tissue (fig. S6). We used non-negative matrix factorization (NMF) of the cell-type-abundance estimates in tissue spots to identify microenvironments of colocalized cell types in the profiled tissues in an unbiased manner (Fig. 1D and figs. S7 to S10).

In the developing liver, we recovered expected signatures of tissue-specific parenchymal cells such as hepatocytes. In addition, we observed spatial segregation of early and late erythrocytes, suggesting distinctive hematopoietic zones (Fig. 1D and fig. S8). In the developing thymus, we recovered the localization of cell types in known histological structures. Developing T cells, for example, were largely localized to the thymic cortex, whereas mature T cells were consistently mapped to the thymic medulla. Furthermore, in two of the thymic tissue sections, we observed aggregates of lymphoid tissue (hereafter referred to as thymus-associated lymphoid aggregates). Within these, we mapped B cell subsets, innate lymphoid cells (ILCs), and macrophage subtypes (Fig. 1D and fig. S9). In the developing spleen, most of the tissue was highly vascularized. In addition, within splenic lymphoid aggregates, we were able to distinguish partially overlapping B cell zones and T cell zones (Fig. 1D and fig. S10).

## Heterogeneity of prenatal myeloid cells across organs and gestation

We first examined the main compartments of immune cells in our multi-organ dataset to identify gestation-specific and organ-specific variability within cell populations.

The myeloid compartment captured the development from committed myeloid progenitors to neutrophils, monocytes, macrophages, and DCs (fig. S4, G and H). Our cross-tissue analysis distinguished three distinct subsets of monocytes, characterized by a differential distribution between prenatal bone marrow and peripheral tissues and by the expression of *CXCR4, CCR2,* or *IL1B* (17). Among macrophages, we identified eight broad macrophage groupings based on their transcriptome profile (fig. S4H): “LYVE1^hi^”, “iron-recycling”, “MHC class II^hi^”, “Kupffer-like” *(18),* “microglia-like TREM2^hi^” (19), “osteoclasts” (11, 20), and “proliferating” macrophages. Assigning proliferating cells to the other identified subsets, we observed a high fraction of proliferating macrophages in the yolk sac and within the LYVE1^hi^ subset across organs, suggesting an increased self-renewal potential (fig. S11).

We compared prenatal and adult immune-cell populations by mapping a cross-tissue adult dataset of immune cells (21) onto our prenatal myeloid reference (fig. S12, A and B). We found the transcriptional profiles of DC subsets were conserved between adult and prenatal counterparts (fig. S12C). Adult monocytes were most similar to the IL1B^hi^ and CCR2^hi^ prenatal populations and no CXCR4^hi^ monocytes in non-lymphoid adult tissues were observed (fig. S13). The majority of adult macrophages clustered separately from the prenatal macrophages, with the exception of erythrophagocytic macrophages (fig. S12, B and C). This population includes macrophages primarily from the spleen and liver that carry out iron-recycling functions (21).

**Fig. 2:**
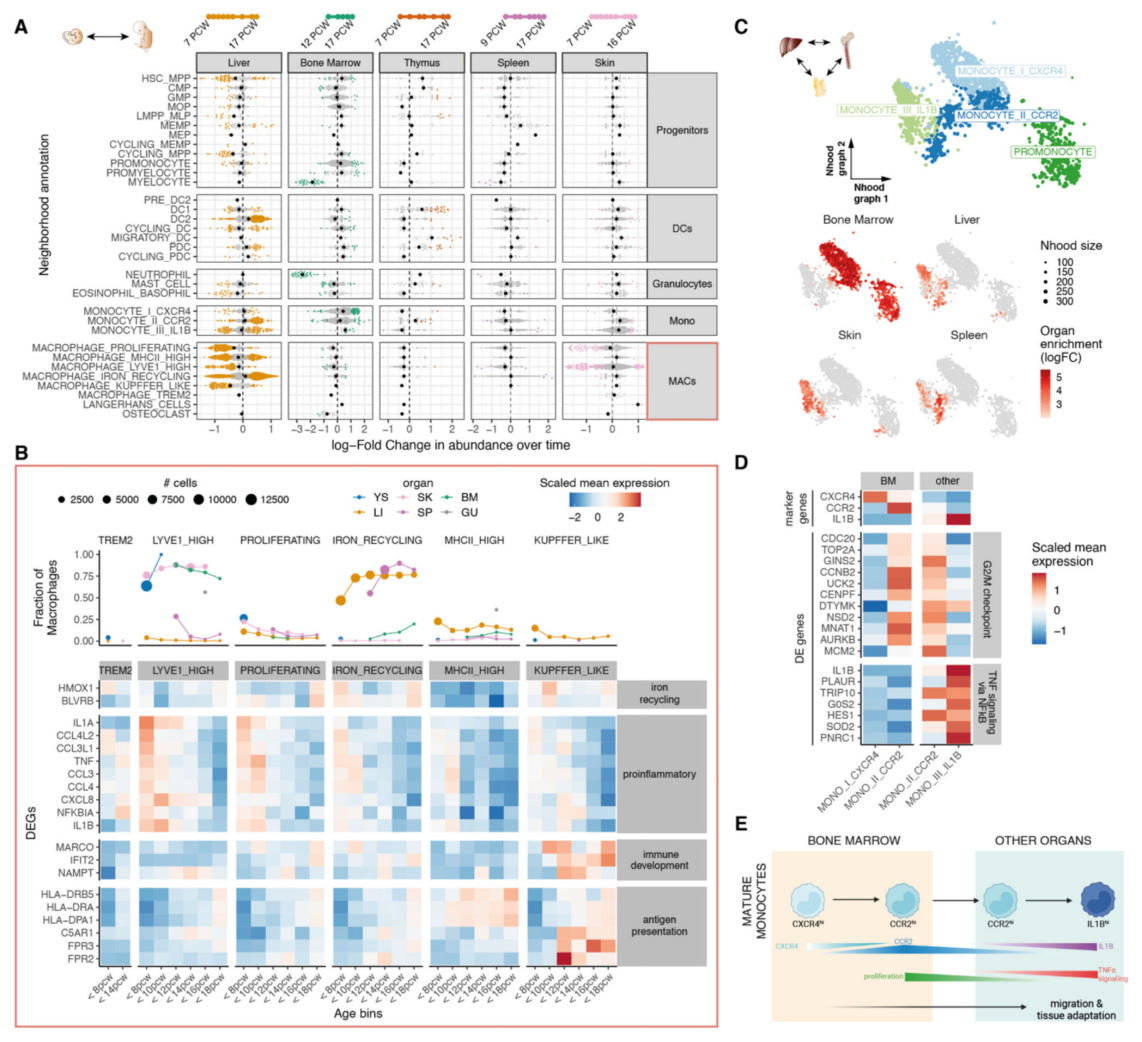
Myeloid variation across time and tissues. (A) Beeswarm plot of log-fold change (*x*-axis) in cell abundance across gestational stages in Milo neighborhoods of myeloid cells. Results from five organs are shown. Neighborhoods overlapping the same cell population are grouped together (*y*-axis), and colored if displaying significant differential abundance (DA) (SpatialFDR 10%). The black dot denotes the median log-fold change. The top bar denotes the range of gestational stages of the organ samples analyzed. (B) Heat map of average expression across time of a selection of markers of stage-specific macrophage neighborhoods. Mean log-normalized expression for each gene is scaled (*z*-score). Gestational ages are grouped in 5 age bins. Age bins where less than 30 cells of a given subset were present are not shown. The top panel shows the fraction of all macrophages belonging to the specified macrophage population in each time point and each organ (color). (C) Close-up view of monocytes on Milo neighborhood embedding of myeloid cells (subset from fig. S16). Top: neighborhoods are colored by overlapping cell population. Bottom: neighborhoods displaying significant DA (SpatialFDR 10%) are colored by log-fold change in abundance between the specified organ and all other organs. (D) Mean expression of a selection of differentially expressed genes between CCR2hi monocytes from bone marrow and other organs. Log-normalized expression for each gene is scaled (*z*-score). We show genes upregulated in bone marrow associated with G2/M checkpoint and genes downregulated in bone marrow associated with TNF signaling (from MSigDB Hallmark 2020 gene sets). (E) Schematic of the proposed process of monocyte egression from the bone marrow mediated by CXCR4 and CCR2 expression: CXCR4^hi^ monocytes are retained in the bone marrow, until they switch to a proliferative state with increased expression of CCR2, mediating tissue egression. CCR2^hi^ monocytes seed peripheral tissues and then mature further to the periphery-specific IL1B expressing subtype.

To quantify changes in cellular composition across gestation, we used differential abundance analysis on cell neighborhoods with Milo (Fig. 2A and fig. S14A) (22). This analysis reaffirmed well-known compositional shifts that happen during gestation. For example, myeloid progenitor cells decreased in the liver but increased in the bone marrow, recapitulating the transition from liver to bone marrow hematopoiesis. DCs increased in proportional abundance across multiple tissues as previously described in the liver and bone marrow (3). Notably, for several cell populations, we found that some neighborhoods were enriched and others depleted across gestation, suggesting evolving transcriptional heterogeneity during development. This was especially evident in the macrophage compartment in the skin and liver (Fig. 2A) with a large fraction of neighborhoods overlapping the LYVE1^hi^ and proliferating macrophages enriched in early gestation. Differential expression analysis revealed the upregulation of a proinflammatory gene signature with chemokines and cytokines specific to early stages in all macrophage subtypes across tissues (Fig. 2B, fig. S14B). TNF and NF-κB have been implicated in lymphoid tissue organogenesis (23) and the chemokines noted here have been associated with angiogenesis (24–27). Conversely, a large fraction of neighborhoods within the iron-recycling and MHCII^hi^ macrophage populations were enriched in later stages of gestation. We found that these subpopulations upregulated genes encoding for immune effector functions (Fig. 2B; fig. S14C; and table S1). In parallel to macrophages, we observed similar transcriptional heterogeneity during gestation in mast cells (Fig. 2A). Specifically, early mast cells in yolk sac, liver, and skin displayed a similar proinflammatory phenotype characterized by expression of *TNF* and NF-κB subunits, as well as chemokines associated with endothelial cell recruitment (*CXCL3*, *CXCL2*, and *CXCL8*) (26) (fig. S15).

These findings suggest that early macrophages and mast cells may contribute to angiogenesis, tissue morphogenesis and homeostasis as previously reported (28–30), prior to adopting traditional immunological functions. Notably, acquisition of macrophage antigen-presentation properties (e.g., MHCII upregulation) between 10 and 12 pcw coincided with the expansion of adaptive lymphocytes (fig. S1E) and development of lymphatic vessels and lymph nodes (31).

Differential abundance analysis on cell neighborhoods to test for organ-specific enrichment (fig. S16A) revealed that CXCR4^hi^ monocytes were enriched in bone marrow, and IL1B^hi^ monocytes in peripheral organs. Among CCR2^hi^ monocytes, we distinguished bone marrow- and peripheral organ-specific subpopulations (Fig. 2C). Bone marrow CCR2^hi^ monocytes expressed proliferation genes, whereas peripheral organ CCR2^hi^ monocytes upregulated *IL1B* and other TNF-α signaling genes (Fig. 2D; fig. S16B; and table S2). This suggests that a CXCR4^hi^-to-CCR2^hi^ transition accompanies monocyte egress from the bone marrow to seed peripheral tissues, and CCR2^hi^ monocytes further mature in tissues into IL1B^hi^ monocytes (Fig. 2, D and E). In mouse bone marrow, interactions between monocyte CXCR4 and stromal cell CXCL12 retain monocytes in situ until CCR2-CCL2 interactions predominate, potentially enabling monocyte egress (17). Here, we observed *CXCL12* expression in bone marrow fibroblasts and osteoblasts (fig. S16C). By contrast, the proportion of CXCR4^hi^ monocytes in the developing liver was much lower (fig. S16D), in keeping with reports that alternative mechanisms of monocyte retention and release operate in the murine developing liver (32).

## Heterogeneity of prenatal lymphoid cells across organs and gestation

The lymphoid compartment captures the development of B and T cells, together with ILC and natural killer (NK) cell subsets (fig. S4, I to L).

Mapping adult cells onto our prenatal lymphoid reference, NK cells and type 3 ILCs (ILC3) displayed high similarity between adult and prenatal counterparts (fig. S17, A and B). Among adult T cells, naive populations and regulatory T cells (Tregs) closely matched prenatal conventional T cells (CD4^+^ T, CD8^+^ T, and Tregs), whereas resident and effector memory T cells did not have a developmental equivalent (fig. S17, C and D), although we cannot exclude the possibility that memory T cells appear after 17 pcw as previously reported (33, 34). We did not find a clear matching between adult T cell subsets and prenatal unconventional T cells (type 1 and type 3 innate T and CD8AA in our annotation). All adult B cell progenitors, plasma B cells, naive B cells and memory B cells had prenatal counterparts, but no adult B cells were transcriptionally similar to prenatal putative B1 cells (fig. S17C).

**Fig. 3:**
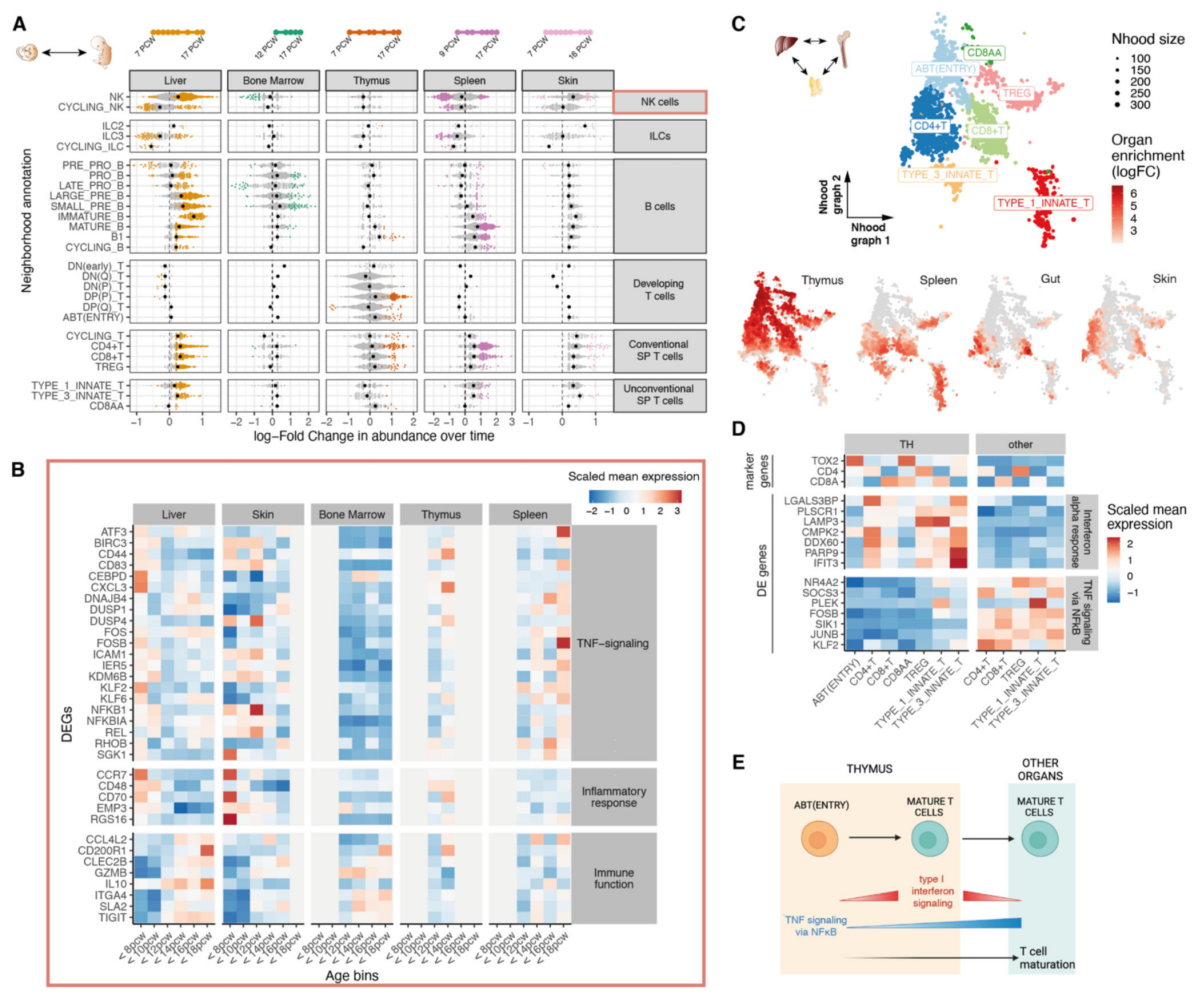
Lymphoid variation across time and tissues. (A) Beeswarm plot of log-fold change (*x*-axis) in cell abundance across gestational stages in Milo neighborhoods of lymphoid cells (as in fig. 2A). (B) Heat map showing average expression across time of a selection of genes identified as markers of early-specific and late-specific NK neighborhoods (as in fig. 2B): NK cells identified in liver and skin before 12 pcw express TNF proinflammatory genes, whereas expression of immune-effector genes such as cytokines, chemokines and granzyme genes increases after 12 pcw. Age bins where less than 30 NK cells were present in a given organ are grayed out. (C) Close-up view of single-positive T cells on Milo neighborhood embedding of lymphoid cells. Each point represents a neighborhood, the layout of points is determined by the position of the neighborhood index cell in the UMAP in fig. S4I. Top: neighborhoods are colored by the cell population they overlap. Bottom: neighborhoods are colored by their log-fold change in abundance between the specified organ and all other organs. Only neighborhoods displaying significant differential abundance (SpatialFDR 10%) are colored. (D) Mean expression of a selection of differentially expressed genes between single-positive T cells from thymus (TH) and other organs. We show genes downregulated in the thymus associated with TNF signaling (using MSigDB Hallmark 2020 gene sets) and genes upregulated in the thymus associated with an IFN-α response. (E) Schematic of the proposed mechanism of thymocyte maturation and egression from thymus mediated by type I interferon signaling and NF-κB signaling.

Differential abundance analysis across gestation identified a broad shift from innate to adaptive immune populations (Fig. 3A and fig. S18A). ILCs and NK cells included cell neighborhoods both enriched and depleted across gestation. Genes involved in inflammatory responses, including TNF signaling, were overexpressed in <12 pcw liver and skin NK cells, although late splenic NK cells also expressed these genes. Conversely, late NK cells across organs overexpressed genes involved in cytokine signaling and granzyme genes (Fig. 3B; fig. S18, B and C; and table S3). As is the case for macrophages, these results suggest the progressive development of immune-effector function by NK cells.

We next tested for organ-specific cell neighborhoods in the lymphoid compartment (fig. S19A). Although certain populations of mature T cells were exclusively enriched in the thymus (ABT(entry), CD8AA), we found that neighborhoods of conventional and unconventional T cells could be subdivided into a subset enriched in the thymus and other subsets enriched in peripheral organs (Fig. 3C). Thymic mature T cells overexpressed genes involved in interferon (IFN)-α signaling whereas peripheral mature T cells had higher expression of genes associated with TNF and NF-κB signaling (Fig. 3D; fig. S19B; and table S4). Both pathways have been implicated in last stages of functional maturation of murine T cells right before emigration out of the thymus (35, 36). In addition to the increase in type 1 IFN and NF-κB signaling accompanying ABT(entry) to thymic mature T cells, expression of NF-κB-signaling genes continued to increase when mature T cells migrate out to peripheral tissues (Fig. 3E).

## System-wide blood and immune cell development

**Fig. 4:**
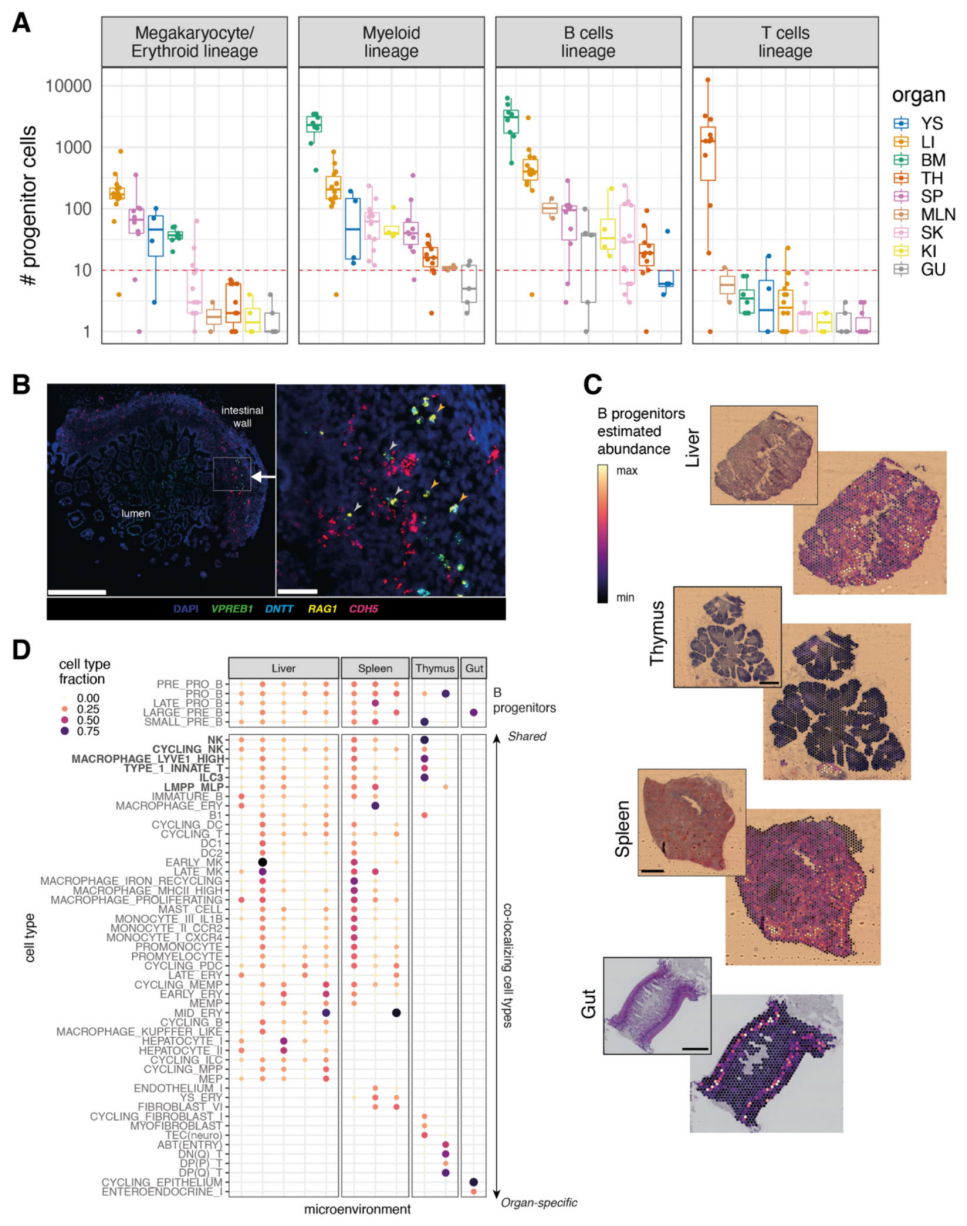
System-wide blood and immune cell development. (A) Boxplots of the number of progenitor cells in all donors across organs. Each point represents a donor, color-coded by organ (YS: yolk sac; LI: liver; BM: bone marrow; TH: thymus; SP: spleen; MLN: mesenteric lymph node; SK: skin; GU: gut; KI: kidney). The red dash line marks the threshold of 10 cells for potential technical artefacts. B_prog: B cell lineage progenitors; M/E_prog: megakaryocyte/erythroid progenitors; MYE_prog: myeloid progenitors; T_prog: T cell progenitors. Detailed cell types included in each lineage are shown in table S5. Boxes capture the first-to-third quartile of the cell number and whisks span a further 1.5X interquartile range on each side of the box. (B) Multiplex smFISH staining with DAPI, *CDH5* for endothelial cells, and *VPREB1, DNTT, RAG1* for B cell progenitors in the human prenatal intestine at 15 pcw. Left panel shows a zoomed-out view with the area of interest boxed in white (scale bar: 500 μm). Right panel shows a detailed view of the area of interest (scale bar: 50 μm). Gray arrows point to B cell progenitors associated with blood vessels and orange arrows point to B cell progenitors away from blood vessels. (C) Scaled sum of abundances of B progenitor cell types estimated with cell2location, shown on representative slides for each organ, with the corresponding H&E staining. Each scale bar represents the length of 1 mm. (D) Cell type contributions to microenvironments containing B cell progenitors in different organs identified with non-negative matrix factorization of spatial cell type abundances estimated with cell2location. The color and the size of the dots represent the relative fraction of cells of a type assigned to the microenvironment.

While examining the distribution of various cell types across different organ systems, we were surprised to find that lineage-committed hematopoietic progenitors were present in non-hematopoietic organs. In particular, we detected B cell progenitors in almost all prenatal organs, megakaryocyte/erythroid progenitors in developing spleen and skin, and myeloid progenitors in the thymus, spleen, skin, and kidney (Fig. 4A). By contrast, T cell progenitors were restricted to the thymus, potentially reflecting more stringent niche requirements for T cell development and consistent with the observed absence of T cells in children with congenital athymia (37). This finding suggests that hematopoiesis is not restricted to developing liver and bone marrow between 7-17 pcw (38) and other organs can also support blood and immune cell differentiation during prenatal development.

In addition, across progenitor lineages, cells of different developmental stages were simultaneously present in peripheral organs (fig. S20, A to D). Cell-fate-prediction analysis delineated a continuum of cells between HSCs and different lineage immune cells in multiple organs (fig. S20, C and D), supporting the conclusion that lineage-committed differentiation takes place within peripheral organs.

Single-molecule fluorescence in situ hybridization (smFISH) staining confirmed the existence of lineage-committed progenitors in multiple organs. Cells simultaneously expressing *VPREB1, RAG1*, and/or *DNTT* were present in the prenatal gut, spleen, and thymus (Fig. 4B and fig. S21, A to C), consistent with B cell progenitors. Although some B cell progenitors in the prenatal gut were associated with *CDH5*-expressing blood vessels, many could be detected extravascularly (Fig. 4B), further supporting the conclusion that B cells develop in prenatal peripheral organs. We also validated the presence of megakaryocyte/erythroid lineage progenitors in prenatal spleen and thymus (fig. S22, A to C), and that of myeloid lineage progenitors in prenatal gut and thymus (fig. S23, A to C).

Focusing on B lymphopoiesis given its widespread nature, we used cell2location (16) on Visium spatial transcriptomic data and found B cell progenitors were localized in the submucosa of the gut, in thymus-associated lymphoid aggregates, and proximal to lymphoid aggregates in the spleen (fig. S24B), in addition to their expected presence in the developing liver (Fig. 4C and fig. S24A). The widespread nature of B lymphopoiesis suggests that the cellular environments supporting B cell development are much more widely available than previously thought. Spatial transcriptomic data identified cells colocalizing with B cell progenitors across multiple organs, including ILC3, LYVE1^hi^ macrophages, NK cells, type 1 innate T cells, and LMPP_MLP (see fig. S24C for predicted cell–cell interactions), whereas other colocalizing cell types were organ-specific (Fig. 4D).

## Identification of putative prenatal B1 cells

**Fig. 5:**
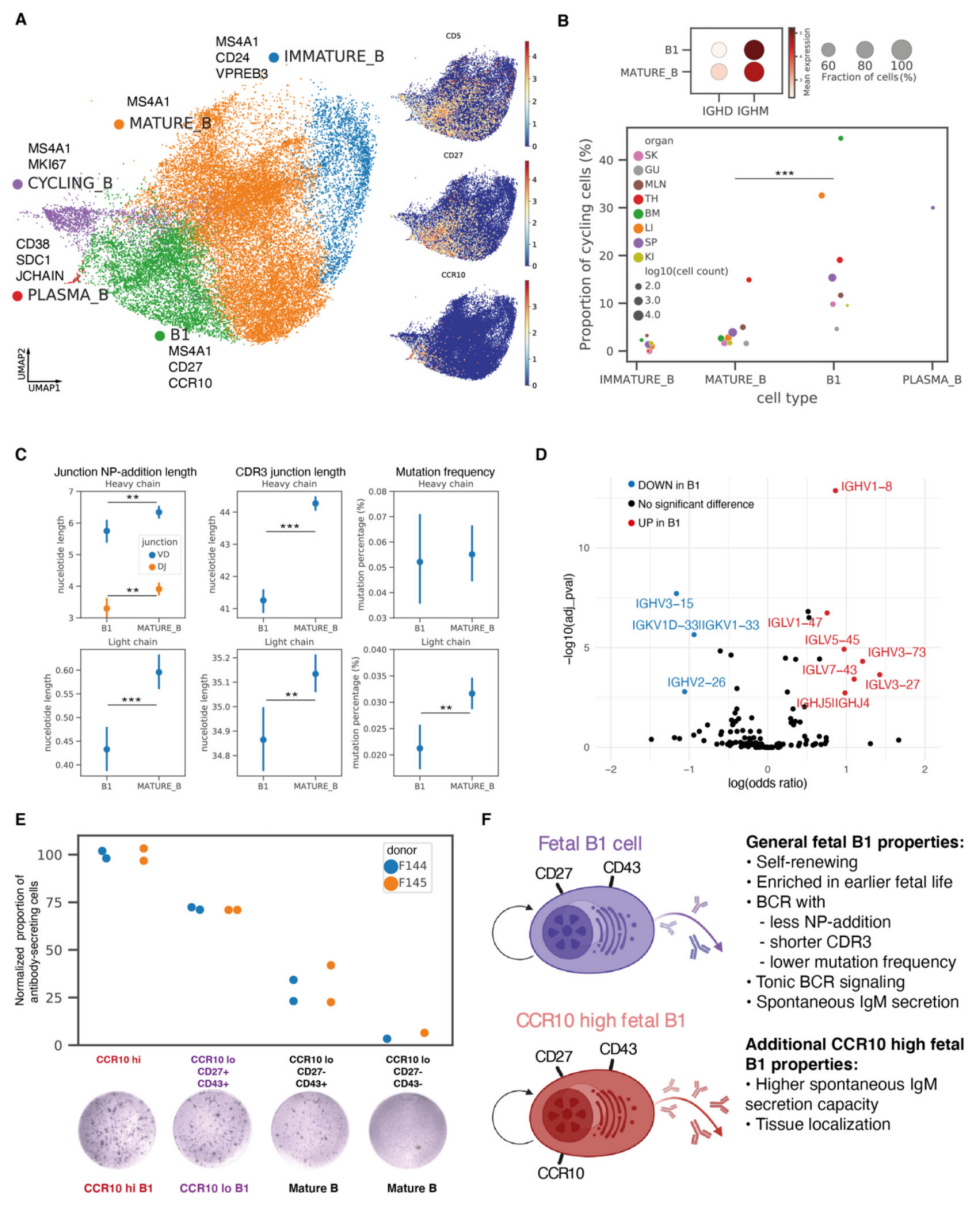
Identification of putative prenatal B1 cells. (A) Left: Close-up view of non-progenitor B cell populations on UMAP embedding of all lymphoid cells (fig. S4I), with marker genes listed next to each cell type. Right: expression of B1 marker genes on UMAP. (B) Top: dot plot of *IGHM* and *IGHD* expressions in B1 and mature B cells, with color of dots representing the mean expression and size representing the fraction of cells expressing the gene. Bottom: cycling cell proportions within each B cell subtype colored by organs, with dot size representing log_10_(cell count) and only dots with at least 10 cells shown. B1 cells had significantly higher cycling proportions than mature B cells in a logistic regression controlling for donors and organs. (C) Point plots of NP-addition length, CDR3 junction length, and mutation frequency in BCR heavy chains or light chains in B1 (N=2,357) and mature B (N=7,387) cells, with points representing the mean and lines representing 95% confidence intervals. Heavy chain VD and DJ junction NP-addition lengths are only calculated for cells with high-quality D gene mapping (B1: N=615, mature B: N=2,430). Difference in characteristics were tested with linear regressions controlling for donors and organs. (D) Volcano plot summarizing results of BCR heavy and light chain V, J gene segment usage comparison between B1 and mature B cells. The *y*-axis is the −log_10_(Benjamini–Hochberg adjusted *P*-value) and the *x*-axis is log(odds ratio) computed using logistic regression controlling for donors and organs. (E) Normalized proportions of antibody-secreting cells in different sorted fractions of the ELISpot experiments (raw counts in table S6), colored by donor. Each point represents a reaction well. The proportions of antibody-secreting cells were normalized against the average proportion in CCR10^hi^ wells for each donor to remove donor-specific effects. A representative well image for each sorted fraction is shown on the bottom. (F) Schematic illustration summarizing the features of all human prenatal B1 cells, and additional features specific to CCR10^hi^ prenatal B1 cells.

Among prenatal non-progenitor B cells that had productive BCR light chains and low *IL7R* expression (fig. S25A), we identified immature B, mature B, cycling B, plasma B cells, and putative B1 cells (Fig. 5A and fig. S25B). These putative B1 cells had the highest expression of *CD5, CD27*, and *SPN*(CD43), consistent with previously reported markers (39–41), as well as *CCR10,* a highly-specific marker that was expressed in a subset of B1 cells (Fig. 5A).

We next evaluated characteristics typical of murine B1 cells, including self-renewal (42, 43), high IgM and low IgD expression (44), emergence in early development (45), low levels of non-templated nucleotide BCR insertions (46, 47), tonic BCR signaling (39), and spontaneous antibody secretion (42).

With regards to B1 cell self-renewal, we calculated the percentage of cycling cells (as indicated by non-zero *MKI67* expression) within immature B, mature B, B1, and plasma B cells, respectively (Fig. 5B and fig. S26A). The proportion of cycling B1 cells was significantly higher than cycling mature B cells, consistent with their capacity for self-renewal. B1 cells expressed lower levels of *IGHD* and higher levels of *IGHM* compared to mature B cells (Fig. 5B). Moreover, the highest frequency of B1 cells was found in early embryonic stages. These were gradually replaced by other subsets of non-progenitor B cells over time. The ratio of B1 to mature B cells showed a general decrease from the first to second trimester across most organs except the thymus (fig. S26B) where B1 cells persisted, consistent with a previous report of a shared phenotype between thymic B cells and B1 cells (48).

Analysis of non-templated nucleotide insertions in the BCR showed that both N/P-additions and CDR3 junctions in heavy and light chains were shorter in B1 cells compared to mature B cells (Fig. 5C). Moreover, a lower mutation frequency was observed in the light chains of the B1 cells compared to that in mature B cells, and the average mutation frequency was lower than that observed in adult B cells (21, 49). We next examined the V(D)J usage within different B cell subtypes along the developmental path (fig. S26C). Prenatal B1 and mature B cells both exhibited a varied BCR repertoire with minimal clonal expansion (fig. S26D) and had differing preferential usage of V(D)J segments (Fig. 5D).

Our putative B1 cells showed features of tonic BCR signaling, with higher B cell activation scores (fig. S26E), as well as higher transcription factor (TF) activity in the TNF-α and NF-κB signaling pathway (fig. S26F), which is downstream of BCR signaling (50), compared with mature B cells.

We assessed spontaneous antibody secretion capacity in B1 cells by flow-sorting B cell subsets, (fig. S26G) and assessing spontaneous IgM secretion by enzyme-linked immune absorbent spot (ELISpot) assay. The normalized antibody-secreting spot counts were higher in the two B1 fractions than the two mature B fractions, with the CCR10^hi^ B1 fraction showing the highest spot counts (Fig. 5E). scRNA-seq of the sorted B cell fractions on a different sample using the same gating strategy further confirmed that the two sorted B1 fractions were indeed B1 cell-enriched compared to the mature B fractions (fig. S26H). We also explored the potential role of *CCR10* in prenatal B1 cells and observed the expression of one of its ligands *CCL28* in bone marrow stroma (chondrocyte and osteoblast), gut epithelium, and keratinocytes and melanocytes in skin (fig. S26I). Thus, *CCR10* may play a role in the tissue localization of prenatal B1 cells.

Overall, our scRNA-seq, paired V(D)J sequencing data as well as the functional assay we have performed provide an extended characterization of human prenatal B1 cells (Fig. 5F).

## Human unconventional T cells are trained by thymocyte-thymocyte selection

The mature T cell compartment consisted of conventional T cells (CD4^+^ T, CD8^+^ T and Tregs) and unconventional T cells. The origin of the latter in humans is poorly understood. Unconventional T cells expressed the key innate marker *ZBTB16* (PLZF) (fig. S27A) (51) and could be further separated into three different subtypes: *RORC-* and *CCR6-* expressing type 3 innate T cells; *EOMES-* and *TBX21-* expressing type 1 innate T cells; and *PDCD1*- expressing and thymus-restricted CD8AA cells (figs. S2 and S4L), corresponding, respectively, to T_H_17-like cells, NKT-like cells, and CD8αα^+^ T cells (7).

The proportions of unconventional T cells among all mature T cells exhibited a decreasing trend from 7-9 pcw to 10-12 pcw across most of the organs surveyed here (fig. S27B). Type 1 and 3 innate T cells were almost negligible in postnatal thymus, whereas CD8AA T cell abundance rebounded in pediatric age groups before a further decline in adulthood (fig. S27B). Thus, type 1 and 3 innate T cells, but not CD8AA, appear to be developmental-specific unconventional T cells.

Spatially, we found that mature T cells segregated into two microenvironments in the thymic medulla (fig. S27C). Conventional CD4^+^ T and CD8^+^ T cells colocalized with mTECs close to the inner medulla, whereas CD8AA and type 1 innate T cells colocalized with type 1 DCs (DC1) near the cortico-medullary junction (fig. S27, D and E). Treg and type 3 innate T cells were located within both microenvironments. Thus, in contrast to conventional T cells, CD8AA and type I innate T cells likely undergo distinct negative selection processes mediated by DCs rather than mTECs and may also be involved in DC activation as previously suggested (7).

**Fig. 6:**
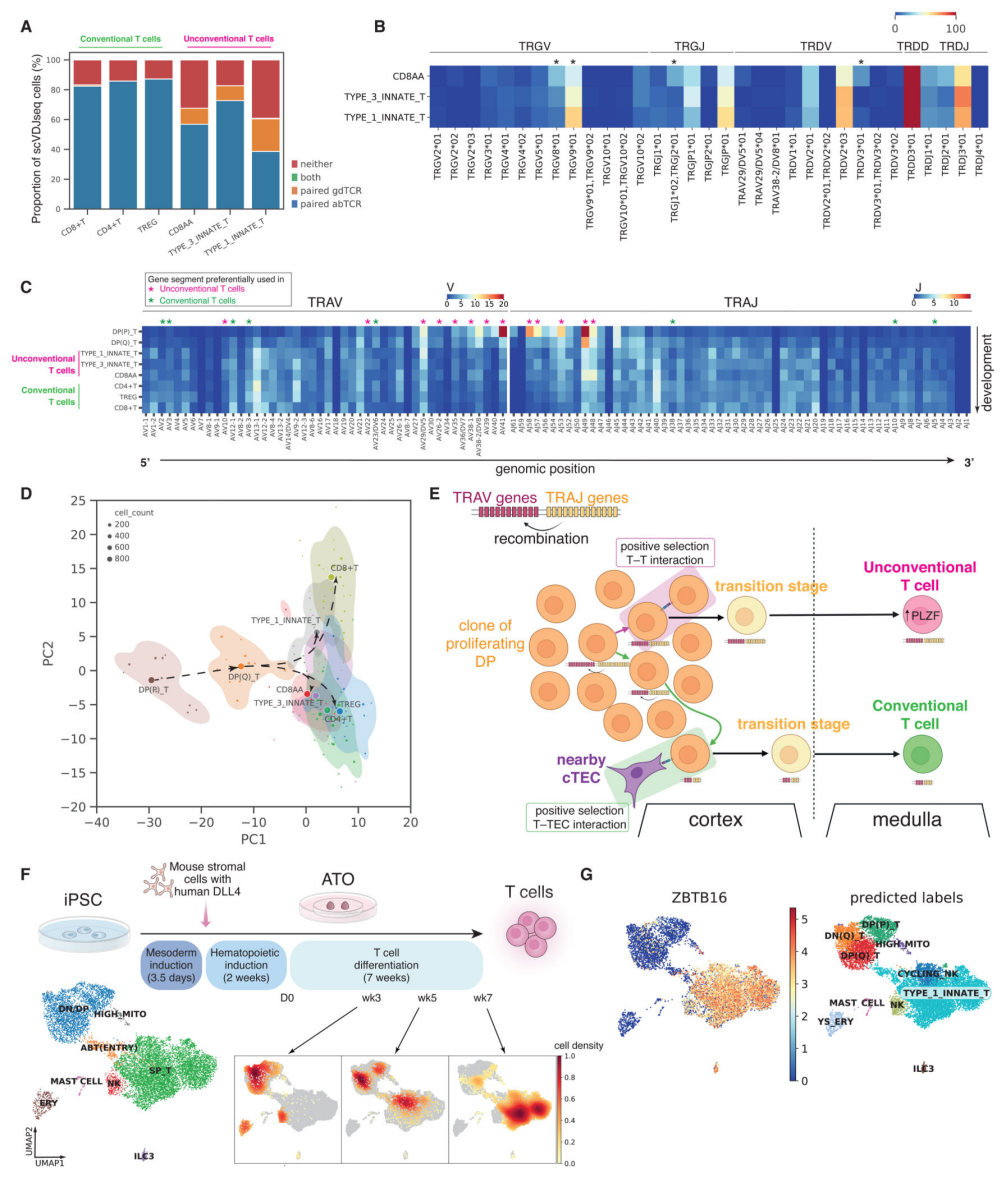
Deep characterization of human unconventional T cells. (A) Proportions of cells expressing paired γδTCR, paired αβTCR, or both or neither. The proportions were calculated over cells that have had both single-cell αβTCR and γδTCR sequencing. The cells that expressed neither paired αβTCR nor paired γδTCR could be due to dropouts in single-cell TCR sequencing as over 50% of these contained orphan VDJ or VJ chains of αβTCR or γδTCR (fig. S28A). (B) Heat map showing the percentage of each γδTCR gene segment present in different T cell subtypes. Differential usage between cell subtypes was computed using the chi-squared test and gene segments with Benjamini–Hochberg adjusted *P*-values < 0.05 are marked with *. (C) Heat map showing the proportion of each TCRα gene segment present in different T cell subtypes. The gene segment usage in unconventional T cells and conventional T cells was compared using logistic regression, controlling for donors and organs. Gene segments with Benjamini–Hochberg adjusted P-value<0.05 are marked with magenta * (for preferential usage in unconventional T cells) and green * (for preferential usage in conventional T cells). (D) PCA plot summarizing TRAV, TRAJ, TRBV, TRBJ gene segment usage proportion in different T cell subtypes. Each dot represents a sample of at least 20 cells, with its size representing the cell count. The centroid of each cell type is shown as a filled circle, and 80% confidence contours are shown around the centroids. Arrows illustrate the proposed developmental trajectories. (E) Schematic illustration showing the T–T training origin of unconventional T cells in contrast to the T–TEC training origin of conventional T cells. (F) Top: schematic showing the experimental set-up of T cell differentiation from iPSCs in ATOs. Bottom left: UMAP visualization of different cell types in the ATO. Bottom right: density plots of cells from each time point over UMAP embedding. (G) Left: predicted annotations from logistic regression overlaid on the same UMAP plot as in (F); right: *ZBTB16* expression pattern overlaid onto the same UMAP plot.

Single-cell sequencing of γδTCR and αβTCR was performed on a subset of samples to characterize antigen-receptor repertoires in unconventional T cells (Fig. 1B). The vast majority of unconventional T cells expressed paired αβTCR but some of these cells expressed paired γδTCR (Fig. 6A). The majority of the γδT cells expressed *TRGV9* and *TRDV2* (Fig. 6B), consistent with previous reports (52, 53). However, there was also a large proportion of γδT cells, particularly those of CD8AA and type 3 innate T cell subtypes, expressing *TRGV8* or *TRGV10* instead (Fig. 6B). Thus, the γδTCR showed a relatively restricted repertoire and substantial clonal expansion (fig. S28B).

Prenatal unconventional T cells expressed a varied αβTCR repertoire (Fig. 6C and fig. S28C) with minimal clonal expansion (fig. S28D), unlike well-described unconventional T cell subsets (e.g., type I NKT and mucosal-associated invariant T (MAIT) cells) in adults (54). V-J gene usage in TCRα was previously observed to have a strong association with T cell developmental timing (7, 55). Specifically, double-positive (DP) T cells tend to use proximal TRAV, TRAJ gene segments, whereas mature T cells tend to use more distal pairs, governed by the processive depletion of proximal segments in V-J gene recombination (55). The V-J gene usage of αβTCR-expressing unconventional T cells lies between that of DP cells and conventional T cells, shown by the more proximal gene usage in unconventional T cells (Fig. 6C) and the principal component analysis (PCA) of TCR repertoire (Fig. 6D). This suggests that unconventional T cells are developmentally closer to DP cells (Fig. 6E) and undergo fewer recombinations before positive selection.

Previous studies have suggested that these PLZF-expressing unconventional T cells may originate from positive selection on neighboring T cells (51, 56–58) in contrast to conventional T cells arising from positive selection on cortical thymic epithelial cells (cTECs). Post β-selection, DP T cells undergo proliferation prior to recombination of TCRα (7, 59, 60). Each DP cell is hence surrounded by several neighboring DP cells from the same clone. It is therefore plausible that it requires less physical migration and hence is quicker for a DP T cell to receive positive signaling from a neighboring DP T cell rather than having to migrate to meet a nearby cTEC. Thus, the fact that unconventional T cells have a more similar TCR usage to DP cells agrees with the thymocyte–thymocyte (T–T) origin hypothesis.

To test our hypothesis for T–T-mediated selection of unconventional T cells, we differentiated induced pluripotent stem cells (iPSCs) into mature T cells using the artificial thymic organoid (ATO) (61). Importantly, there were no human thymic epithelial cells present in the ATO system (Fig. 6F). scRNA-seq analysis of differentiated cells harvested at weeks 3, 5, and 7 from two iPSC lines confirmed that the in vitro culture system recapitulated T cell development from double-negative (DN) and DP, to ABT(ENTRY) then to single-positive mature T cells (SP_T) (Fig. 6F, fig. S29A to C). SP_T cells differentiated in vitro were dominated by *ZBTB16*-expressing unconventional T cells (Fig. 6G). Both label transfer (Fig. 6G) and similarity scoring on merged embeddings (fig. S29D) showed that the in vitro SP_T were most similar to in vivo type 1 innate T cells. Thus, our in vitro experiments support the T–T origin hypothesis of unconventional T cells.

## Discussion

Our study provides a comprehensive single-cell dataset of the developing human immune system, spanning over 900,000 single-cell profiles from nine tissues, encompassing over 100 cell states. Compared to previous multi-organ developmental atlases (9), we increased coverage of developmental organs and gestation stages, sequencing depth, and generated paired BCR, αβTCR, and γδTCR datasets. Moreover, we demonstrate the utility of scRNA-seq reference to delineate tissue organization and cellular communication in spatial transcriptomics, providing a proof-of-concept study of the localizations of immune cells across prenatal tissues. Our pre-processed data and pre-trained models (scVI and CellTypist models) will facilitate alignment of new data to our dataset and streamline future expansion and analysis of human developmental atlases.

Our cross-organ analysis reveals several important biological phenomena. First, human macrophages, mast cells and NK cells transcriptomically acquire immune effector functions between 10 and 12 pcw. Their transcriptomic signatures prior to this time-point suggest a role in tissue morphogenesis, in line with previous suggestion for murine macrophages (62) and may explain why these cells appear in early development. The coincidental development of the lymphatic system around 12 pcw (31) raises the possibility of its potential role in initiating this transcriptional switch. Second, there are conserved processes of proliferation and maturation for monocytes and T cells prior to their migration from the bone marrow and thymus respectively into peripheral tissues.

Third, in contrast to the previous dogma of hematopoiesis being restricted to the yolk sac, liver, and bone marrow during human development, system-wide blood and immune cell development takes place in peripheral organs, although at varying extents in different lineages. It is possible that hematopoiesis is supported to varying levels in prenatal organs, including the adrenal gland (9), prior to the onset of functional organ maturation as exemplified by the fetal liver, which transitions from a hematopoietic to a metabolic organ. The potential for other peripheral organs to support hematopoiesis is evidenced by the re-emergence of extramedullary hematopoiesis in adults, primarily in pathological settings (63–66), as well as the recent description of B lymphopoiesis in murine and non-human primate meninges (67–69).

Finally, this work identifies and functionally validates the properties of human prenatal innate-like B and T cells and provides an extensive characterization of human B1 cells. Our in vivo αβTCR V(D)J usage patterns and in vitro T cell differentiation data proposes T–T selection underpinning unconventional T cell development. Further studies are required to establish if B1 cells arise from different progenitors (lineage model) (70–72) or from the same progenitors but with different signaling (selection model) (73, 74), similar to the conventional and unconventional T cell model proposed here. Notably, both innate-like B and T cells were abundant during early development and their precise role at this developmental time-point warrants further investigations. Their reported debris-removal (41, 42), antigen-reactivity (41, 42, 54), and regulatory functions (42) may confer these prenatal innate-like B and T cells with tissue-homeostatic and important immunological roles.

In summary, this comprehensive atlas of the developing human immune system provides valuable resources and biological insights to facilitate in vitro cell engineering, regenerative medicine, and enhance our understanding of congenital disorders affecting the immune system.

## Materials and Methods

A more detailed version of the materials and methods is provided in the Supplementary Materials.

### Tissue acquisition and processing

Human developmental tissue samples (4 to 17 pcw, metadata in table S7) used for this study were obtained from the MRC–Wellcome Trust-funded Human Developmental Biology Resource (HDBR; http://www.hdbr.org) with written consent and approval from the Newcastle and North Tyneside NHS Health Authority Joint Ethics Committee (08/H0906/21+5). All tissues were digested into single-cell suspensions with 1.6 mg/ml of type IV collagenase (Worthington).

### Single-cell RNA sequencing experiment

Dissociated cells were stained with anti-CD45 antibody and DAPI prior to sorting. For scRNA-seq experiments, either Chromium single cell 3′ reagent kit or Chromium single cell V(D)J reagent kits from 10X Genomics were used. Unsorted, or DAPI^–^CD45^+^, or DAPI^–^CD45^–^ FACS-isolated cells were loaded onto each channel of the Chromium chip. Single-cell cDNA synthesis, amplification, gene expression (GEX) and targeted BCR and TCR libraries were generated. Targeted enrichment for γδTCR was performed following the TCR enrichment protocol from 10X with customized primers (table S8) (75). Sequencing was performed on the Illumina Novaseq 6000 system. The gene expression libraries were sequenced at a target depth of 50,000 reads per cell using the following parameters: Read1: 26 cycles, i7: 8 cycles, i5: 0 cycles; Read2: 91 cycles to generate 75-bp paired-end reads. BCR and TCR libraries were sequenced at a target depth of 5000 reads per cell.

### ATO cell cultures

We followed the PSC-ATO protocol as previously described (61) (more details in the Supplementary Materials). Two iPSC lines HPSI0114i-kolf_2 (Kolf) and HPSI0514i-fiaj_1 (Fiaj) obtained from the Human Induced Pluripotent Stem Cell initiative (HipSci: www.hipsci.org) collection were used.

### Visium

OCT-embedded freshly frozen samples (table S9) were used for 10X Genomics Visium. All tissues were sectioned with a thickness of 15 μm. Optimal 18-min permeabilization was selected for fetal spleen and liver, whereas a 24-min permeabilization was used for fetal thymus. The spatial gene expression library was then generated following the manufacturer's protocol. All images for this process were acquired with a Zeiss AxioImager (Carl Zeiss Microscopy) and a 20X air objective (0.8 NA) using either fluorescence (Zeiss Axiocam 503 monochrome camera) for optimization or brightfield mode (Zeiss Axiocam 105 color camera) for H&E imaging. ZEN (Blue edition) v.3.1 was used for acquisition and stitching of the image tiles.

### Single-molecule fluorescence in situ hybridization (smFISH)

smFISH was performed on thymus, spleen, and gut sections, using the RNAScope 2.5 LS multiplex fluorescent assay (ACD, Bio-Techne) on the automated BOND RX system (Leica).

Slides were stained for DAPI (nuclei) and three or four probes of interest, with fluorophores atto 425, opal 520, opal 570, and opal 650. Positive and negative control probes were used to optimize staining conditions for all tissues.

For fetal gut and spleen, OCT-embedded freshly frozen 10 μm-thick sections were pretreated offline for 15 min with chilled 4% paraformaldehyde and dehydrated through an ethanol series (50%, 70%, 100%, and 100% ethanol), before processing on the Leica BOND RX with protease IV for 30 min at room temperature. The sections were imaged on a Perkin Elmer Opera Phenix High Content Screening System (16-bit sCMOS camera, PerkinElmer) with a 20X water objective (High NA, PerkinElmer). Due to high levels of endogenous autofluorescence, we imaged one of the spleen sections (fig. S21A) with a confocal microscope (Leica SP8) with a 40X 1.3NA oil immersion objective and SP8 Leica HyD and PMT detectors.

Due to the high cellular density in thymic sections, we used 3 μm-thick FFPE sections that were treated on the Leica Bond RX with epitope retrieval 2 for 15 min at 95°C and protease III for 15 min at 40°C. Imaging was performed on an Operetta CLS High Content Screening System (16-bit sCMOS camera, PerkinElmer) with a 40X water objective (High NA, PerkinElmer) and 2-μm *z*-steps.

### scRNA-seq analysis

#### Preprocessing

The gene expression data was mapped with cellranger 3.0.2 to an Ensembl 93 based GRCh38 reference (10X-distributed 3.0.0 version). Ambient RNA was removed with cellbender v0.2.0 (76). Low-quality cells were filtered out (minimum number of reads = 2000, minimum number of genes = 500, Scrublet (v0.2.3) (77) doublet detection score <0.4). We identified possible maternal contamination using the souporcell pipeline for genotyping (v.2.4.0) (78) (see Supplementary Materials for details).

#### Data integration and annotation

Data normalization and preprocessing were performed using the Scanpy workflow (v1.8.1) (79). We normalized raw gene read counts by sequencing depth in each cell (*scanpy*.*pp*.*normalize*_*per*_*cell*, with parameters *counts*_*per*_*cell*_*after*=*10e4*) and performed *ln(x)* + *1* transformation. We then selected highly variable genes (HVG) for joint embedding by dispersion (*scanpy*.*pp*.*highly*_*variable*_*genes* with parameters *min*_*mean* = *0.001*, *max*_*mean* = *10*). We performed dimensionality reduction and batch correction using the scVI model (12) as implemented in scvi-tools (v0.14.5) (80), considering 10X chemistry (5′ and 3′) and the donor ID for each cell as the technical covariates to correct for (training parameters: *dropout_rate* = *0*.*2*, *n*_*layers* = *2*). The model was trained on raw counts of the 7500 most highly variable genes, excluding cell cycle genes and TCR/BCR genes (7) with 20 latent dimensions. To verify conservation of biological variation after integration, we collected and harmonized the available cell type labels from the published datasets (66% of cells) and quantified the agreement between labels across different datasets in the cell clusters identified post-integration, using the normalized mutual information (NMI) score, as implemented in *scikit-learn* (81). Unless otherwise specified, cell clustering was performed using the Leiden algorithm (82) (resolution = 1.5, *n_neighbors* = 30). To verify robustness to the choice of integration method, we performed in parallel integration using BBKNN (83) as previously described (7) (fig. S30A). We found that clustering post-integration both with scVI and BBKNN was consistent with previous annotations (fig. S30B).

To annotate fine cell populations across tissues, we clustered cells in the scVI latent space and preliminarily assigned cells to broad lineages, using expression of marker genes and previous annotations. For each broad lineage we repeated scVI integration and clustering as described above and defined further subsets (see hierarchy in fig. S5). Leiden clusters for the highest resolution subsets (stroma, megakaryocyte/erythroid, progenitors, lymphoid, myeloid) were annotated manually, using marker panels shown in fig. S4 (see Supplementary Materials for a more detailed description of annotation strategy). We verified that refined annotations were highly consistent with unsupervised clustering post-integration on the full dataset both with scVI and BBKNN (fig. S30C).

After full annotation, 23,156 cells (2.5% of total) were assigned to low quality clusters (doublet clusters, maternal contaminants clusters and clusters displaying a high percentage of reads from mitochondrial genes).

#### Differential abundance analysis

We tested for differences in cell abundances associated with gestational age or organ using the Milo framework for differential abundance testing (22), with the python implementation milopy (https://github.com/emdann/milopy). A more detailed description of this analysis can be found in the Supplementary Methods.

Briefly, we subsetted the dataset to cells from libraries obtained with CD45^+^ FACS, CD45^–^ FACS or no FACS, excluding FACS-isolated samples for which we were not able to recover the true sorting fraction quantification. In total, we retained 228,731 lymphoid cells and 214,874 myeloid cells. To further minimize the differences in cell numbers driven by FACS efficiency, we calculated a FACS correction factor for each sample to use as confounding covariate in differential abundance testing (fig. S32, see Supplementary Methods). We constructed a KNN graph using similarity in the scVI embedding (k = 30 for test across gestation, k = 100 for test across tissues) and assigned cells to neighborhoods (*milopy.core.make_nhoods*, parameters: *prop* = *0.05*). We then counted the number of cells belonging to each sample in each neighborhood (*milopy.core.count_cells*). We assigned each neighborhood a cell type label based on majority voting of the cells belonging to that neighborhood. We assigned a “Mixed” label if the most abundant label is present in less than 50% of cells within that neighborhood.

To test for differential abundance across gestational age, we divided the sample ages into six equally sized bins (bin size = 2 pcw) and excluded samples from organs where less than three consecutive age bins were profiled (yolk sac, mesenteric lymph node, kidney, and gut). We model the cell count in neighborhoods as a negative binomial generalized linear model, using a log-linear model to model the effects of age on cell counts, while accounting for FACS correction factor and the total number of cells over all the neighborhoods. We control for multiple testing using the weighted BH correction as described in (22). To detect markers of early-specific neighborhoods (SpatialFDR < 0.1, logFC < 0) and/or late-specific neighborhoods (SpatialFDR < 0.1, logFC > 0) in cell type *c* and organ *o*, we tested for differential expression between cells from organ *o* assigned to the significant neighborhoods labeled as cell type *c* and cells belonging to all other neighborhoods labeled as cell type *c*. We used the *t* test implementation in scanpy (*scanpy.tl.rank_genes_groups, method = "t-test_overestim_var"*). Genes expressed in > 70% of tested cells were excluded. We considered genes as significantly overexpressed (i.e., markers) if the differential expression logFC > 1 and FDR < 0.1%. Gene set enrichment analysis was performed using the implementation of the EnrichR workflow (84) in the python package gseapy (https://gseapy.readthedocs.io/). The list of significantly overexpressed genes for all organs and cell types where differential expression testing was carried out can be found in tables S1 and S3.

To test for differential abundance between organs, we modeled the cell count in neighborhoods as above, using a log-linear model to model the effects of organ on cell counts, while accounting for FACS correction factor, library prep protocol, and the total number of cells over all the neighborhoods. We considered the neighborhoods where $\beta_{n}^{o}>0$ and SpatialFDR < 0.01 as cell subpopulations that show organ-specific transcriptional signatures.

Having identified a subset of neighborhoods overlapping a cell type that were enriched in a certain organ, we performed differential expression (DE) analysis between these cells and cells from the same cell type (see Supplementary Methods for a more detailed description). Briefly, we first aggregated single-cell expression profiles into pseudo-bulk expression profiles $\hat{x}$ for each cell type and *c* sample *s* (as recommended by (85, 86)).

We then model the mRNA counts of gene *g* in pseudobulk *p* by a NB-GLM:

$${\bar{x}}^{g,p}=NB(\mu_{g,p,}\phi_{g,p})$$

The expected count value *μ*_*n*,*p*_ is given by the following log-linear model

$$\text{log}\;\mu_{g,p}=\beta_{0}+d_{p}\beta_{g}^{\text{donor}}+o_{p}\beta_{g}^{\text{organ}}+c_{p}\beta_{g}^{\text{celltype}}+c_{p}o_{p}\beta_{g}^{\text{organ}\times \text{celtype}}+\text{log}\;L_{p}$$

We estimated the log-fold change $\beta_{g}^{\text{organ}\times \text{celtype}}$ in expression in a given cell type for organ $\hat{o}$ using the quasi-likelihood method (87) implemented in the R package glmGamPoi (85). We used the estimated logFC from the test on a set of control cell types (where we don't expect to see organ-specific differences) to filter out genes where differential expression is driven by technical differences in tissue processing. We provide the full results for the differential expression analysis between organs in mature T cells and monocytes in tables S2 and S4.

#### TCR analysis

Single-cell αβTCR sequencing data was mapped with cellranger-vdj (v.6.0.0). The output file *filtered_contig_annotations.csv* was used and analyzed with scirpy (v.0.6.0) (88). Single-cell γδTCR sequencing data was mapped with cellranger-vdj (v.4.0.0). All contigs deemed high-quality were selected, and re-annotated with igblastn (v.1.17.1) against IMGT reference sequences (last downloaded: 01/08/2021), via a workflow provided in dandelion (v0.2.0) (89) (https://github.com/zktuong/dandelion). The output file *all_contig_dandelion.tsv* was used and analyzed with scirpy (v0.6.0).

#### BCR analysis

Single-cell BCR data was initially processed with cellranger-vdj (v.6.0.0). BCR contigs contained in *all_contigs.fasta* and *all_contig_annotations.csv* were then processed further using dandelion (89) singularity container (v.0.2.0). BCR mutation frequencies were obtained using the *observedMutations* function in shazam (v.1.0.2) (90) with default settings.

#### B cell activation scoring

Gene Ontology B Cell Activation gene list was downloaded from Gene Set Enrichment Analysis website (http://www.gsea-msigdb.org/gsea/msigdb/genesets.jsp). Cells were scored according to expression values of all genes in this gene list apart from three genes that were not present in the dataset using *scanpy.tl.score_genes()* function.

#### Transcription factor activity inference

We used the DoRothEA Python package (v.1.0.5) (91) to infer TF activities in B1 and mature B cells. TFs that had higher activities (positive “meanchange”) in B1 cells were then ranked according to their adjusted *P*-values and only top 25 TFs are shown in fig. S26F.

#### Cell—cell interaction analysis

We used the CellPhoneDB Python package (v.3.0) (92, 93) to infer cell–cell interactions. The scRNA-seq dataset was split by organ and cell types with fewer than 20 cells in a given organ were filtered out. CellPhoneDB was run separately to infer cell–cell interactions in each organ, using default parameters. To explore cell–cell interactions between B cell progenitors and colocalizing cell types (fig. S24D), we aggregated the interactions predicted between each colocalizing cell type, by averaging the means and using the minimum of the *P*-values. We then filtered for the ligand-receptor pairs that were significant (*P*<0.05) across all three organs of liver, spleen and thymus, and ranked by the maximum aggregated means. Only the top 60 ligand-receptor pairs are shown in fig. S24C.

#### Query-to-reference mapping

We mapped query data to our prenatal data embeddings using online update of the scVI models following the scArches method (15), as implemented in the *scvi-tools* package (80). The model was trained for 200 epochs and setting *weight_decay* = *0*, to ensure that the latent representation of the reference cells remained exactly the same. Reference genes missing in the query were set to 0, as recommended in (15). To generate a joint embedding of query and reference cells, we concatenated the latent dimensions learnt for query cells to the latent dimensions used for the reference embedding and computed the KNN graph and UMAP as described above. To assess that the mapping to the developmental reference conserves biological variation while minimising technical variation in the query data, we compared query cell type labels and batch labels with clusters obtained from Leiden clustering on the learnt latent dimensions, using the Normalized Mutual Information score (see fig. S33 for mapping of adult query data).

#### Annotation prediction using CellTypist

We used CellTypist v.0.1.9 Python package (21) to perform annotation prediction with logistic regression models. For prediction on cycling B cells, the rest of the non-progenitor B cells, including immature B, mature B, B1, and plasma B cells were used as training dataset. Default parameters were used for model building and prediction was made without majority voting for accurate enumeration of predicted B cell subtypes within cycling B cells.

#### Comparison with human adult immune cells

Single-cell RNA-seq data from adult immune cells was generated and preprocessed as described (21). The dataset including cell type annotations were provided by the authors. We mapped 264,929 adult lymphoid cells to the lymphoid embeddings of our developmental dataset and 54,047 adult myeloid cells to our myeloid embedding. In order to use cell annotations in our developmental dataset to predict adult cell types in the joint embedding, we adapted the KNN-classifier approach described in (15) and we calculated the similarity to prenatal cells labeled taking the Euclidean distance in the joint embedding, weighted by a Gaussian kernel.

#### Blood and immune cell progenitors scRNA-seq data analysis

For the cell-fate-prediction analysis shown in fig. S20, C and D, we used the Palantir method as implemented in CellRank (94, 95). Briefly, from the scVI embedding on all immune cells (fig. S20A), we selected cells belonging to progenitor populations and computed a KNN graph on scVI latent dimensions on these cells (k=30). Then transition probabilities were calculated using the *ConnectivityKernel* in the cellrank package. We computed coarse-grained macrostates with Generalized Perron Cluster Cluster Analysis, setting the number of macrostates to the number of annotated progenitor cell populations. We manually set the four target terminal states for each lineage (small pre B cells, DN(Q) T cells, early MKs, and promonocytes) and computed the probability of each cell to transition to one of the four terminal states. The fate simplex visualization in fig. S20, C and D, was generated using the function *cellrank.pl.circular_projection*.

#### Artificial thymic organoids scRNA-seq data analysis

Raw scRNA-seq reads were mapped with cellranger 3.0.2 with combined human reference of GRCh38.93 and mouse reference of mm10-3.1.0. Low quality cells were filtered out (minimum number of reads = 2000, minimum number of genes = 500, minimum Scrublet (77) doublet detection score <0.4). Cells where the percentage of counts from human genes was <90% were considered as mouse cells and excluded from downstream analysis. Cells were assigned to different cell lines (Kolf, Fiaj) using genotype prediction with souporcell (v.2.4.0) (78). We performed batch correction to minimize the differences between cells from different cell lines using scVI and clustered cells using the Leiden algorithm on the latent embedding as described above. We used CellTypist v.0.1.9 Python package (21) to perform annotation prediction with logistic regression using the whole in vivo scRNA-seq developmental dataset for training. For the in vivo-to-in vitro similarity analysis in fig. S29D, we mapped in vitro cells to the scVI model of lymphoid cells as described above. For each cell in the in vitro dataset, we calculated the Euclidean distance weighted by a Gaussian kernel to the closest in vivo cell from each in vivo cell population.

### Spatial data analysis

Spatial transcriptomics data was mapped using spaceranger (v.1.2.1) and we used a custom image-processing script to identify regions overlapping tissues. To map cell types identified by scRNA-seq in the profiled spatial transcriptomics slides, we used the cell2location method (16) (see Supplementary Methods). Briefly, for the reference model training step, we excluded very lowly expressed genes using a recommended filtering strategy (16). Cell types where fewer than 20 cells were profiled in the organ of interest and cell types labeled as low-quality cells were excluded from the reference. For the analysis of unconventional T cell localization in thymus (fig. S27C), we trained a reference adding all the prenatal thymic epithelial cells from a thymus cell atlas (7) (data was downloaded from Zenodo (96)). For the spatial cell type deconvolution step, all slides representing a given organ were analyzed jointly. To identify microenvironments of colocalizing cell types, we used non-negative matrix factorization (NMF) on the matrix of estimated cell type abundances. Here latent factors correspond to tissue microenvironments defined by a set of colocalized cell types. We use the NMF implementation in scikit-learn (81), setting the number of factors *d* = 10. For downstream analysis, we excluded cell types where the 99% quantile of cell abundance across locations in every slide from the same organ was always below the detection threshold of 0.15. Unless otherwise specified, we consider a cell type to be part of a microenvironment if the cell type fraction was over 0.2.

For analysis of mature T cell localization in the thymic medulla (fig. S27, D and E), we retained factors where the sum of the cell type fractions for mature T cells (CD4^+^ T, CD8^+^ T, Treg, type 1 innate T, type 3 innate T, and CD8AA) was above 0.8. We assigned spots to the inner medulla or cortico-medullary microenvironment if the factor value in the spot was above the 90% quantile of all values in the slide. To annotate cortex and medulla from histology images, we extracted image features from the high resolution images of H&E staining using the python package squidpy (v1.1.2) (97) and performed Leiden clustering on image features. We then defined the cortico-medullary junction (CMJ) using spatial neighbor graph functionality in squidpy (see Supplementary Methods).

### B1 functional validation experiment

Cryopreserved single-cell suspensions from F144 (17 pcw) and F145 (15 pcw) spleen samples were used for the ELISpot experiment. B cells were gated as singlet DAPI^−^CD3^−^CD20^+^ cells. Plasma cells should generally be CD20^lo^ and therefore not included. To further exclude plasma cell contamination, we also gated out the top 1% of B cells expressing the highest level of CD38. The rest of the B cells were then sorted into four fractions: CCR10^hi^, CCR10^lo^CD27^+^CD43^+^, CCR10^lo^CD27^–^CD43^+^, and CCR10^lo^CD27^–^CD43^–^. CD27 and CD43 gates were chosen based on fluorescence minus one (FMO) controls.

The ELISpot experiment was performed with Human IgM ELISpot^BASIC^ kit (ALP) from Mabtech AB. Post sorting, 7000-8000 cells were added into ELISpot plate pre-coated with anti-IgM antibody and incubated at 37°C for 22 hours. The plate was then washed and incubated with biotinylated anti-IgM for 2 hours at room temperature, followed by a 1-hour incubation with streptavidin-ALP. The colored spots were developed with a 15-min incubation of BCIP/NBT substrate solution. Spots were counted with the AID ELISpot reader and iSpot software version 4.

In addition, we performed scRNA-seq of the sorted B cell fractions on a different donor (F149, 18 pcw fetal spleen), using the same gating strategy to further confirm the identity of sorted cells. The scRNA-seq data was preprocessed with scVI as above. Cell annotations were predicted using CellTypist v.0.1.9 (21).

## Supplementary Material

Table S1

Table S2

Table S3

Table S4

Table S5

Table S6

Table S7

Table S8

Table S9

Supplementary Materials

## Data Availability

Raw sequencing data for newly generated sequencing libraries have been deposited in ArrayExpress (scRNA-seq libraries: accession number E-MTAB-11343; scVDJ-seq libraries: accession number E-MTAB-11388; Visium 10X libraries: accession number E-MTAB-11341). Processed data objects are available for online visualization and download in AnnData format (98) at https://developmental.cellatlas.io/fetal-immune, as well as trained scVI models for query to reference mapping and trained Celltypist models for cell annotation. All code scripts and notebooks for analysis presented in the manuscript are available at https://github.com/Teichlab/Pan_fetal_immune (99). This publication is part of the Human Cell Atlas (www.humancellatlas.org/publications).

## One-Sentence Summary

We present a human developing immune system atlas across time and multiple organs with important biological discoveries.
