# Supplementary material for: Mapping the developing human immune system across organs
Source: Science. Author manuscript; Available in PMC 2022 Jun 7. (PMC7612819; doi:10.1126/science.abo0510)
Supplement: Supplementary Materials [file EMS144959-supplement-Supplementary_Materials.pdf]

Supplementary Materials:
Supplementary Materials and Methods
Figs. S1 to S33
Tables S1 to S9

Supplementary Materials for

Mapping the developing human immune system across organs

Chenqu Suo*, Emma Dann*, Issac Goh, Laura Jardine, Vitalii Kleshchevnikov, Jong-Eun Park, Rachel A. Botting, Emily Stephenson, Justin Engelbert, Zewen Kelvin Tuong, Krzysztof Polanski, Nadav Yayon, Chuan Xu, Ondrej Suchanek, Rasa Elmentaite, Cecilia Domínguez Conde, Peng He, Sophie Pritchard, Mohi Miah, Corina Moldovan, Alexander S. Steemers, Pavel Mazin, Martin Prete, Dave Horsfall, John C. Marioni, Menna R. Clatworthy†, Muzlifah Haniffa†, Sarah A. Teichmann†

Correspondence to: mrc38@cam.ac.uk (M.C.), m.a.haniffa@newcastle.ac.uk (M.H.); st9@sanger.ac.uk (S.A.T.)

This PDF file includes:

Supplementary Materials and Methods
Figs. S1 to S33
Tables S1 to S9

Supplementary Materials and Methods

Tissue acquisition and processing

All human developmental tissue samples used for this study were obtained from the MRC–Wellcome Trust-funded Human Developmental Biology Resource (HDBR; http://www.hdbr.org) with written consent and approval from the Newcastle and North Tyneside NHS Health Authority Joint Ethics Committee (08/H0906/21+5).

All tissues were processed into single-cell suspensions immediately upon receipt. Tissue was first minced in a tissue culture dish using scalpel. It was then digested with type IV collagenase (final concentration of 1.6 mg/ml; Worthington) in RPMI (Sigma-Aldrich) supplemented with 10% fetal bovine serum (FBS; Gibco), at 37°C for 30 min with intermittent agitation. Digested tissue was then passed through a 100-µm cell strainer and cells were pelleted by centrifugation at 500*g* for 5 min at 4°C. Cells were then resuspended in 5 ml of red blood cell lysis buffer (eBioscience) and left for 5-10 min at room temperature. It was then topped up with flow buffer (PBS containing 2% (v/v) FBS and 2 mM EDTA) to 45 ml prior to cell counting and antibody staining. Single-cell suspensions were generated from 76 samples across yolk sac (7), liver (6), spleen (30), thymus (4), kidney (2), and skin (27) of 16 donors. The ages of the donors spanned from 4 pcw (post conception weeks) to 17 pcw. The metadata of all samples, including previously published data, can be found in table S7.

Single-cell RNA sequencing experiment

Dissociated cells were stained with anti-CD45 antibody (BUV395 anti-human CD45 antibody, BD Biosciences, 563791) and DAPI (Sigma-Aldrich, D9542) prior to sorting. For all FACS experiments performed in this study, DAPI was used at a final concentration of 2.8 µM, and all antibody solutions were used at a final concentration of 2 µl per 100 µl cell suspensions containing fewer than 5 million cells. Sorting by flow cytometry was performed with BD FACSAria Fusion Flow Cytometer. The $CD45^+$ fraction was sorted from $DAPI^-CD45^+$ gate and $CD45^-$ fraction was sorted from $DAPI^-CD45^-$ gate. CD45 gating was contiguous so that no live cells were lost in sorting.

For scRNA-seq experiments, either Chromium single cell 3′ reagent kit or Chromium single cell V(D)J reagent kits from 10X Genomics were used. Unsorted, or $DAPI^-CD45^+$, or $DAPI^-CD45^-$ FACS-isolated cells were loaded onto each channel of the Chromium chip following the manufacturer's instructions before droplet encapsulation on the Chromium controller. Single-cell cDNA synthesis, amplification, gene expression (GEX) and targeted B cell receptor (BCR) and T cell receptor (TCR) libraries were generated. Targeted enrichment for γδTCR was performed following the TCR enrichment protocol from 10X with customized primers binding to the constant region of the TRD and TRG genes as described (*75*). Primers are listed in table S8.

Sequencing was performed on the Illumina Novaseq 6000 system. The gene expression libraries were sequenced at a target depth of 50,000 reads per cell using the following parameters: Read1: 26 cycles, i7: 8 cycles, i5: 0 cycles; Read2: 91 cycles to generate 75-bp paired-end reads. BCR and TCR libraries were sequenced at a target depth of 5000 reads per cell.

Cell cultures for artificial thymic organoid (ATO)

MS5 line transduced with human DLL4 was obtained from G. Crooks (UCLA) as a gift. The MS5-hDLL4 cells were cultured in DMEM (Gibco) with 10% FBS. Two iPSC lines were used in this study. Cell lines HPSI0114i-kolf_2 (Kolf) and HPSI0514i-fiaj_1 (Fiaj) were obtained from the Human Induced Pluripotent Stem Cell initiative (HipSci: www.hipsci.org) collection. All iPSC lines were cultured on vitronectin (diluted 1:25 in PBS; Gibco) coated plates, in TeSR-E8 media (Stemcell Technologies).

We followed the PSC-ATO protocol as previously described (*61*). iPSC cells were harvested as a single-cell suspension and seeded (3×10^6 cells per well) in GFR reduced Matrigel (Corning) - coated 6-well plates in X-VIVO 15 media (Lonza), supplemented with rhActivin A, rhBMP4, rhVEGF, rhFGF (all from R&D Systems), and ROCK inhibitor (Y27632; LKT Labs) on day −17, and only rhBMP4, rhVEGF and rhFGF on days −16 and −15. Cells were harvested 3.5 days later, and isolated by FACS for CD326$^-$CD56$^+$ (PE anti-human CD326 antibody, Biolegend, 324205; APC anti-human CD56 antibody, Biolegend, 318309) human embryonic mesodermal progenitors (hEMPs).

Isolated hEMPs were combined with MS5-hDLL4 at a ratio of 1:50. Two or three cell-dense droplets (5×10^5 cells in 6 µl hematopoietic induction medium) were deposited on top of an insert in each well of a six-well plate. Hematopoietic induction medium composed of EGM2 (Lonza) supplemented with ROCK inhibitor and SB blocker (TGF-β receptor kinase inhibitor SB-431542; Abcam) was added into the wells outside the inserts so that the cells sat at the air-liquid interface. The organoids were then cultured in EGM2 with SB blocker for 7 days (days −14 to −7), before the addition of cytokines rhSCF, rhFLT3L, rhTPO (all from Peprotech) between days −6 to 0. These 2 weeks formed the hematopoietic induction phase. On day 1, media was changed again to RB27 (RPMI supplemented with B27 (Gibco), ascorbic acid (Sigma-Aldrich), penicillin/streptomycin (Sigma-Aldrich) and glutamax (Thermo Fisher Scientific)) with rhSCF, rhFLT3L and rhIL7. The organoids can be maintained in culture for 7 more weeks in this medium.

For dissociation and checking of ATO, a cell scraper was used to detach ATOs from cell culture insert membranes and detached ATOs were then submerged in cold flow buffer. Culture inserts were washed and detached ATOs were pipetted up and down to form single-cell suspension before passing through a 50-µm strainer. Cells were then stained with designed panels of antibodies and analyzed by flow cytometry. FACS was performed at the same time and live human DAPI$^-$anti-mouse CD29$^-$ (APC/Cy7 anti-mouse CD29 antibody, Biolegend, 102225) cells were sorted for week 3 ATO cells, and live (DAPI$^-$) cells were sorted for week 5 and week 7 ATO cells before loading onto each channel of the Chromium chip from Chromium single cell V(D)J kit (10X Genomics).

Visium

OCT embedded freshly frozen samples were used for 10X Genomics Visium, and samples were processed following manufacturer's instructions. All tissues were sectioned with a thickness of 15 µm on a cryostat (OTF5000, Bright instruments). Tissue optimization was then performed with an 18-min permeabilization for fetal spleen and liver, whereas a 24-min permeabilization

was used for fetal thymus. The spatial gene expression library was then generated following the manufacturer's protocol. All images for this process were acquired with a Zeiss AxioImager (Carl Zeiss Microscopy) and a 20X air objective (0.8 NA) using either fluorescence (Zeiss Axiocam 503 monochrome camera) for optimization or brightfield mode (Zeiss Axiocam 105 color camera) for H&E imaging. ZEN (Blue edition) v.3.1 was used for acquisition and stitching of the image tiles. The metadata of all samples can be found in table S9.

Single molecule fluorescence in situ hybridization (smFISH)

The smFISH technique RNAscope was performed on thymus, spleen, and gut sections, using the RNAScope 2.5 LS multiplex fluorescent assay (ACD, Bio-Techne) on the automated BOND RX system (Leica). Prior to running RNAscope probes of interest, positive and negative control probes were used for optimization of these tissues. Tissue sections were placed onto superfrost plus slides (Fisher scientific) and stained for DAPI (nuclei) and three or four probes of interest, with fluorophores opal 520, opal 570, opal 650 and atto 425. DAPI was used at 1:50,000 concentration; opals at 1:1000 (1:500 for thymus) and atto 425 at 1:400 concentration.

For the fetal gut and spleen, OCT-embedded freshly frozen samples were sectioned to 10 µm-thick. Following optimization, sections were pretreated offline for 15 min with chilled 4% paraformaldehyde and dehydrated through an ethanol series (50%, 70%, 100%, 100% ethanol), before processing on the Leica BOND RX with protease IV for 30 min at room temperature. The sections were imaged on a Perkin Elmer Opera Phenix High Content Screening System (16-bit sCMOS camera, PerkinElmer) with a 20X water objective (High NA, PerkinElmer). Due to high levels of endogenous autofluorescence, we imaged one of the spleen sections (fig. S21A) with a confocal microscope (Leica SP8) with a 40X 1.3NA oil immersion objective and SP8 Leica HyD and PMT detectors. Emission spectral filters were set for DAPI, opal 520 (*VPREB1*), opal 570 (*RAG1*), opal 650 (*CDH5*). Images were processed with Fiji as follows. All channels were subjected to *z*-max projection followed by 2D Gaussian filtering (sigma = 0.5 pixels). The DAPI channel was flat field corrected (biovoxxel – pseudo flat field correction plugin) with a rolling ball radius of 200 pixels. The large image was cropped around the tissue area to remove flat field corrections edges.

Due to the high cellular density in thymic sections, we used 3 µm-thick FFPE sections. These were treated on the Leica Bond RX with epitope retrieval 2 for 15 min at 95°C and protease III for 15 min at 40°C. FFPE thymus did not require any offline pretreatment. Imaging was performed on an Operetta CLS High Content Screening System (16-bit sCMOS camera, PerkinElmer) with a 40X water objective (High NA, PerkinElmer) and 2-µm *z*-steps.

Cells were identified and annotated manually with an in-house OMERO platform (https://www.openmicroscopy.org/omero/).

scRNA-seq analysis

Preprocessing

The gene expression data was mapped with cellranger 3.0.2 to an Ensembl 93 based GRCh38 reference (10X-distributed 3.0.0 version). Ambient RNA was removed with cellbender v0.2.0

(*76*). Low-quality cells were filtered out (minimum number of reads = 2000, minimum number of genes = 500, Scrublet (v0.2.3) (*77*) doublet detection score <0.4).

In order to identify possible maternal contamination, the samples were pooled on a per-donor basis and processed with souporcell (v.2.4.0) (*78*). The common GRCh38 variants file (SNPs with ≥2% frequency from 1k genomes) provided by souporcell authors was used. The pipeline was run twice, setting the number of genotype clusters to 1 and 2 to obtain models for no maternal contamination and possible maternal contamination. The better of these models was identified via BIC (Bayesian Information Criterion), calculated using the formula below:

$$BIC = kn \log(m) - 2l$$

Whereby k is the number of genotype clusters set for each souporcell run, n denotes the number of loci used for genotype deconvolution, m is the cell count for a given donor, and l is the log likelihood obtained after running the pipeline with each k. In two donors (F19 and F37), the BIC was smaller when $k = 2$. The cells with the minor genotype were identified as possible maternal contaminants, which mainly consisted of NK cells, monocytes, mature B and T cells. The cells of minor genotype from the remaining donors were further screened for similar cell compositions, and a further donor (F33) was identified with possible maternal contamination. For these three donors, cells from the minor genotype were excluded from the downstream analysis.

Data integration and annotation

Data normalization and preprocessing were performed using the Scanpy workflow (v1.8.1) (*79*). We normalized raw gene read counts by sequencing depth in each cell (*scanpy.pp.normalize_per_cell,* with parameters *counts_per_cell_after=10e4*) and performed *ln(x)+1* transformation. Expression levels reported in this manuscript refer to normalized and log-transformed gene read counts. We then selected highly variable genes (HVG) for joint embedding by dispersion (*scanpy.pp.highly_variable_genes* with parameters *min_mean = 0.001*, *max_mean = 10*). We considered the 10X chemistry (5′ and 3′) and the donor ID for each cell as the technical covariates to correct for. We performed dimensionality reduction and batch correction using the scVI model (*12*) as implemented in scvi-tools (v0.14.5) (*80*). For model specification and training we used the recommended parameters to enable scArches mapping (*dropout_rate = 0.2*, *n_layers = 2*). To verify conservation of biological variation after integration, we collected and harmonized the available cell type labels from the published datasets (66% of cells) and quantified the agreement between labels across different datasets in the cell clusters identified post-integration, using the normalized mutual information (NMI) score, as implemented in *scikit-learn* (*81*). The model was trained on raw counts of the 7500 most highly variable genes, excluding cell cycle genes and TCR/BCR genes (*7*) with 20 latent dimensions. These parameters (number of HVGs, number of latent dimensions, exclusion/inclusion of cell cycle and TCR/BCR genes) were picked through a parameter sweep, focused on maximizing the NMI between clusters after embedding and pre-existing cell type label annotation (data not shown). Unless otherwise specified, cell clustering was performed using the Leiden algorithm (*82*) with resolution = 1.5 on a k-nearest neighbor graph with $k = 30$. To verify that our cell type clusters were robust to the choice of integration method, we performed in parallel integration on the full dataset using BBKNN (*83*) as previously described (*7*) (fig. S30A). We found that clustering post-integration both with scVI and BBKNN was consistent with previous annotations (fig. S30B).

To annotate fine cell populations across tissues, we clustered cells in the scVI latent space and preliminarily assigned cells to broad lineages examining expression of marker genes and assigning putative cell labels based on previous annotations (we propagated existing cell type labels to unannotated cells by taking the most abundant label in the *k*-nearest neighbors for each unannotated cell). For each broad lineage we repeated scVI integration and clustering as described above and defined further subsets (see hierarchy in fig. S5). Leiden clusters for the highest resolution subsets (Stroma, megakaryocyte/erythroid, progenitors, lymphoid, myeloid) were annotated manually, using marker panels shown in fig. S4. A common subset of progenitor cells was included in scVI embeddings for all hematopoietic-derived cell subsets (megakaryocyte/erythroid, lymphoid, myeloid, NK/T), to allow feature selection and dimensionality reduction to capture the differentiation process of different lineages. A distinct embedding of progenitor cells was then used to finely annotate these cell populations (fig. S4E-F). Blood and immune cell progenitor annotation was based on the subsets and marker genes identified by Jardine et al. (*11*) and Popescu et al. (*3*) (SFig. 31). Macrophage subsets were annotated by analysis of marker genes from human studies in both adult and developmental tissues (*18–20*, *62*) and by unbiased marker gene detection (using *scanpy.tl.rank_genes_groups*) and groupings were defined as follows: "LYVE1^{hi}" expressing *F13A1*, *LYVE1*, and *SPP1*; "Iron-recycling" expressing the highest levels of ferroportin (*SLC40A1*) and phosphatidylserine receptor *TIMD4* but best characterized by expression of *CD5L*, *VCAM1*, and *APOE*; "MHC class II^{hi}" expressing the highest levels of *HLA-DRA*, *HLA-DPA1*, and *CLEC7A* among macrophages; "Kupffer-like" expressing endothelial transcripts *ENG*, *KDR*, and *CAV*; "TREM2" with expression of microglia-associated transcripts *TREM2* and *P2RY12*; "Osteoclasts" expressing characteristic *MMP9* and *ACP5*; and "Proliferating macrophages" expressing genes associated with cell-cycle progression (fig. S4H). Fetal macrophage subsets show a phenotype corresponding to (TIMD4/LYVE1/FOLR2) TLF+ murine macrophages, with potentially additional heterogeneity within the human fraction (*100*) (fig. S4H).

We verified that refined annotations were highly consistent with unsupervised clustering post-integration on the full dataset both with scVI and BBKNN (fig. S30C).

After full annotation 23,156 cells (2.5% of total) were assigned to low quality clusters. These comprised doublet clusters, maternal contaminants clusters and clusters displaying a high percentage of reads from mitochondrial genes.

Differential abundance analysis

We tested for differences in cell abundances associated with gestational age or organ using the Milo framework for differential abundance testing (*22*), with the python implementation milopy (https://github.com/emdann/milopy). Briefly, we subsetted the dataset to cells from libraries obtained with CD45$^+$ FACS, CD45$^-$ FACS or no FACS. In addition, we excluded FACS-isolated samples for which we were not able to recover the true sorting fraction quantification. In total, we retained 228,731 lymphoid cells and 214,874 myeloid cells. To further minimize the differences in cell numbers driven by differences in FACS efficiency, we calculated a FACS correction factor for each tissue sample s sorted with gate i (where i is either CD45$^+$ or CD45$^-$):

$$f_s = \log(p_i S / S_i)$$

where p_i represents the true proportion of cells from gate i in the tissue samples from the same organ and donor, S represents the total number of cells recovered from both CD45$^+$ and CD45$^-$ gates for this organ and donor and S_i represents the number of cells recovered in gate i. For the

unsorted samples we set $f_s = 0$. Encoding the true proportions of CD45$^{+/-}$ cells with f_s reduced the proportion of false positives that were found without regressing out the effect of FACS isolation, or when encoding the effect of sorting as a label rather than a proportion (where CD45^{+} = 1, CD45^{-} = -1, unsorted = 0) (fig. S32A). To validate this approach, we confirmed agreement between estimated fold-changes testing on unsorted samples with the fold-changes estimated accounting for FACS on sorted samples from the same organ (fig. S32B).

We constructed a KNN graph of remaining cells using similarity in the scVI embedding (k = 30 for test across gestation, k = 100 for test across tissues). We assigned cells to neighborhoods on the KNN graph using the function *milopy.core.make_nhoods* (parameters: *prop = 0.05*). We then counted the number of cells belonging to each sample in each neighborhood, creating a cell count matrix with rows representing neighborhoods and columns representing samples (using the function *milopy.core.count_cells*). We assigned each neighborhood a cell type label based on majority voting of the cells belonging to that neighborhood. We assigned a "Mixed" label if the most abundant label is present in less than 50% of cells within that neighborhood.

<u>Differential abundance across time:</u> To test for differences in cell numbers across gestational age, we divided the sample ages into six equally sized bins (bin size = 2 pcw) and excluded from the cell count matrix samples from organs where less than three consecutive age bins were profiled (yolk sac, mesenteric lymph node, kidney, gut). For the matrix of cell counts from samples in each organ, we modeled the cell count $c_{n,s}$ of cells from sample *s* in neighborhood *n* as a negative binomial generalized linear model (NB-GLM):

$$c_{n,s} \sim NB(\mu_{n,s}, \phi_n)$$

where $\mu_{n,s}$ is the mean number of cells from sample *s* in neighborhood *n* and ϕ_n is the dispersion parameter. We used a log-linear model to model the effect of age on cell counts:

$$\log \mu_{n,s} = f_s\beta_n^{\text{facs}} + a_s\beta_n^{\text{age}} + \log L_s$$

here:

- L_s is the sum of counts of cells of sample *s* over all the neighborhoods.
- a_s is the age bin associated to sample *s*.
- β_n^{age} is the regression coefficient encoding the effect of age on the number of cells in neighborhood *n*, that represents the log-fold change (logFC) that can be interpreted as the per-bin linear change in neighborhood cell abundance.
- f_s is the FACS correction factor associated to sample *s*.
- β_n^{facs} is the regression coefficient encoding the effect of CD45 enrichment on the number of cells in neighborhood *n*.

To control for multiple testing, we used the weighted BH correction as previously implemented (*22*). In addition, we tested in parallel for differential abundance associated with the library prep protocol (instead of gestational age) and excluded neighborhoods where we detected significant differential abundance associated with library prep protocol (SpatialFDR < 0.1). We applied this stringent filtering step instead of including the library prep protocol as a covariate in the model (as described below for the test on organ enrichment) to exclude from downstream analysis false positive neighborhoods identified in a small number of thymus samples, where we observed strong confounding between age bins and library prep protocol.

To detect markers of early-specific neighborhoods (SpatialFDR < 0.1, logFC < 0) and/or late-specific neighborhoods (SpatialFDR < 0.1, logFC > 0) in cell type *c* and organ *o*, we tested for differential expression between cells from organ *o* assigned to the significant neighborhoods labeled as cell type *c* and cells belonging to all other neighborhoods labeled as cell type *c*. We used the t-test implementation in scanpy (*scanpy.tl.rank_genes_groups*, *method = "t-test_overestim_var"*). Genes expressed in > 70% of tested cells were excluded. We considered genes as significantly overexpressed (i.e. markers) if the differential expression logFC > 1 and FDR < 0.1%. Gene set enrichment analysis was performed using the implementation of the EnrichR workflow (*84*) in the python package gseapy (https://gseapy.readthedocs.io/). The list of significantly overexpressed genes for all organs and cell types where differential expression testing was carried out can be found in table S1 and S3.

Differential abundance between organs: We modeled the cell counts $y_{n,s}$ for each experimental sample *s* in neighborhood *n* by a Negative Binomial distribution:

$$y_{n,s} = NB(\mu_{n,s}, \phi_{n,s})$$

Where the expected count value $\mu_{n,s}$ is given by the following log-linear model

$$\log \mu_{n,s} = p_s\beta_n^{\text{prep}} + f_s\beta_n^{\text{facs}} + o_s\beta_n^{o} + \log L_s$$

here:

- L_s is the sum of counts of cells of sample *s* over all the neighborhoods.
- o_s is a binary factor indicating whether sample *s* is derived from organ *o*.
- β_n^o is the regression coefficient encoding the effect of the organ on the number of cells in neighborhood *n*, that represents the log-fold change in abundance of cells from organ *o* compared to the cells from other organs.
- f_s is the FACS correction factor associated to sample *s*.
- β_n^{facs} is the regression coefficient encoding the effect of CD45 enrichment on the number of cells in neighborhood *n*.
- p_s is the binary design matrix associating sample *s* to a library prep protocol.
- β_n^{prep} is the regression coefficient encoding the effect of the library prep protocol on the number of cells in neighborhood *n*.

We estimated β_n^o for each *n* and *o* by fitting the NB-GLM to the count data for each neighborhood, i.e. by estimating the dispersion $\phi_{n,s}$ that models the variability of cell counts in replicate samples for each neighborhood. To control for multiple testing we use the weighted BH correction as implemented by Dann et al. (*22*).

We considered the neighborhoods where $\beta_n^o > 0$ and SpatialFDR < 0.01 as cell subpopulations that show organ-specific transcriptional signatures.

Having identified a subset of neighborhoods overlapping a cell type or a subset of transcriptionally related cell types $\hat{c}$ that were enriched in an organ $\hat{o}$, we performed differential expression (DE) analysis between these cells and cells from cell type *c* in other organs. Let $x_i^{g,c,s}$ be the raw gene expression counts of gene g in the *i*th cell from sample *s* and of cell type *c*. We first aggregated single-cell expression profiles into pseudo-bulk expression profiles $\hat{x}$ for each (c,s) (as recommended by (*85*, *86*)):

$$\bar{x}^{g,c,s} = \sum_i x_i^{g,c,s}$$

We next defined a subset of cell types and samples where we will fit the model to test for differentially expressed genes in organ $\hat{o}$. First, we subsetted to the samples from donors where organ $\hat{o}$ and at least 3 other organs were profiled. We then identified 3 cell types $c_j^{\text{ctrl}} \neq \hat{c}$ where at least 2 pseudobulks aggregated from at least 50 cells are profiled in the selected donors, for organ $\hat{o}$ and at least 3 other organs. These cell types represent populations where we don't expect to see biological differences in expression in organ $\hat{o}$.

After sample selection, we subsetted the number of genes for DE testing selecting the top 7500 highly variable genes in $\bar{x}^{\hat{c},s}$ using the method implemented in the R package scran. We further excluded genes where the sum of expression values across pseudobulks from either c_j^{ctrl} or $\hat{c}$ is equal to 0.

These steps yielded a P-by-G data matrix $\bar{X}$, where P is the number of selected pseudobulks and G is the number of selected genes.

We modeled the mRNA counts of gene g in pseudobulk p by a NB-GLM:

$$\bar{x}^{g,p} = NB(\mu_{g,p}, \phi_{g,p})$$

Where the expected count value $\mu_{n,p}$ is given by the following log-linear model

$$\log \mu_{g,p} = \beta_0 + d_p\beta_g^{\text{donor}} + o_p\beta_g^{\text{organ}} + c_p\beta_g^{\text{celltype}} + c_p o_p \beta_g^{\text{organ}\times\text{celltype}} + \log L_p$$

We estimated the log-fold change $\beta_g^{\text{organ}\times\text{celltype}}$ in expression in a given cell type for organ $\hat{o}$ using the quasi-likelihood method (*87*) implemented in the R package glmGamPoi (*85*).

We used the estimated logFC from the test on the control cell types to filter out genes where differential expression is driven by technical differences in tissue processing. In particular, we considered a gene to be significantly overexpressed in cell types $\hat{c}$ and organ $\hat{o}$ if it is significant in the test on $\hat{c}$ (FDR < 10% and logFC > 1) but not in the test on control cell types (FDR > 10% and logFC > 0). We provide the full results for the differential expression analysis between organs in mature T cells and monocytes in tables S2 and S4.

TCR analysis

Single-cell αβTCR sequencing data was mapped with cellranger-vdj (v.6.0.0). The output file *filtered_contig_annotations.csv* was used and analyzed with scirpy (v.0.6.0) (*88*).

Single-cell γδTCR sequencing data was mapped with cellranger-vdj (v.4.0.0). All contigs deemed high-quality were selected, and re-annotated with igblastn (v.1.17.1) against IMGT reference sequences (last downloaded: 01/08/2021), via a workflow provided in dandelion (v0.2.0) (*89*) (https://github.com/zktuong/dandelion). The workflow runs igblastn with the following parameters: minimum D gene nucleotide match = 9, V gene e-value cutoff = 10^{-4}. It also reannotates D and J genes separately using blastn with the following parameters: dust = "no", word size (J = 7; D = 9), e-value cutoff (J = 10^{-4}; D = 10^{-3}). igblastn outputs were parsed into AIRR format with change-o scripts (*90*). The output file *all_contig_dandelion.tsv* was used and analyzed with scirpy (v0.6.0).

We determined productive TCR chain pairing status with *scirpy.tl.chain_qc()* function. For TCR usage PCA and clonotype analysis, cells with orphan VDJ or orphan VJ were filtered out so that each cell has at least one paired TCR. For clonotype analysis, only mature T cells were included to look at clonotype sharing. Clonotypes were determined using *scirpy.pp.ir_neighbors()* and *scirpy.tl.define_clonotypes()* functions with the CDR3 nucleotide sequence identity from both TCR chains as a metric.

Two samples from F67, F67_TH_CD137_FCAImmP7851896 and F67_TH_MAIT_FCAImmP7851897 were excluded from all downstream TCR analysis as they were sorted for specific T cell subpopulations, instead of the CD45 sorting in all other donor samples, and inclusion might result in biased TCR sampling within this donor.

BCR analysis

Single-cell BCR data was initially processed with cellranger-vdj (v.6.0.0). BCR contigs contained in *all_contigs.fasta* and *all_contig_annotations.csv* were then processed as follows: i) re-annotated with igblastn as per above; ii) re-annotated heavy-chain constant region calls using blastn (v.2.12.0+) against curated sequences from CH1 regions of respective isotype class; and iii) heavy-chain v-gene allele correction using tigger (v1.0.0) (*101*). Contigs were then filtered for basic quality control as described previously (*91*). Briefly, the following would lead to removal of contigs from further analysis: i) contigs were annotated with mismatched V, D, J, or constant gene calls not from the same locus; ii) multiple heavy-chain contigs. Exceptions to this would be when a) contigs were assessed to have identical V(D)J sequences but assigned as a different contig by cellranger-vdj (due to difference in non-V(D)J elements), b) when UMI count differences were large in which case the contig with the highest UMI count is retained, and c) if only IgM and IgD were both assigned to a cell; iii) only light-chain contigs in a cell; iv) multiple light-chain contigs in a cell. These were performed using dandelion (*89*) singularity container (v.0.2.0). BCR mutation frequencies were obtained using the *observedMutations* function in shazam (v.1.0.2) (*90*) with default settings (mutation counts for the different regions and mutation types were combined and returned as one frequency value per contig). Mutation rates per cell were averaged across contigs if multiple combinations of productive BCRs pairings were found in a single cell.

BCR clonotypes were determined with *dandelion.tl.find_clones()* function, based on the following criteria for both heavy-chain and light-chain contigs: (1) identical V and J gene usage, (2) identical junctional CDR3 amino acid length, and (3) at least 85% amino acid sequence similarity at the CDR3 junction (based on hamming distance). This strategy was chosen instead of using exact CDR3 nucleotide sequence identity to account for possible somatic hypermutations that happen within the same B cell clone.

B cell activation scoring

Gene Ontology B Cell Activation gene list was downloaded from Gene Set Enrichment Analysis website (http://www.gsea-msigdb.org/gsea/msigdb/genesets.jsp). Cells were scored according to expression values of all genes in this gene list apart from three genes that were not present in the dataset (*MIR17HG, MIR185, MIR19A*) using *scanpy.tl.score_genes()* function.

Transcription factor activity inference

We used the DoRothEA Python package (v.1.0.5) (*91*) to infer TF activities in B1 and mature B cells. Human regulons with confidence level A, B and C in DoRothEA database were utilized, and TF activities were inferred in each cell using *dorothea.run(adata, regulons, center=True,*

num_perm=100, norm=True, scale=True, use_raw=False, min_size=5, use_hvg=False) function. TFs that had higher activities (positive "meanchange") in B1 cells were then ranked according to their adjusted *P*-values and only top 25 TFs are shown in fig. S26F.

Cell–cell interaction analysis

We used the CellPhoneDB Python package (v.3.0) (*92*, *93*) to infer cell–cell interactions. The scRNA-seq dataset was split by organ and cell types with fewer than 20 cells in a given organ were filtered out. CellPhoneDB was run separately to infer cell–cell interactions in each organ, using default parameters. We used *P*-values from the permutation test (*pvalues.txt* output from CellPhoneDB), as well as the average expressions ("means") of the ligand and receptor within their corresponding cell types (*means.txt* output from CellPhoneDB). To explore cell–cell interactions between B cell progenitors and colocalizing cell types (fig. S24D), we aggregated the interactions predicted between each colocalizing cell type (e.g., ILC3 and different subtypes of B cell progenitors (pre-pro B, pro B, late pro B, large pre B and small pre B cells)), by averaging the means and using the minimum of the *P*-values. We then filtered for the ligand-receptor pairs that were significant (P<0.05) across all three organs of liver, spleen, and thymus, and ranked by the maximum aggregated means. Only the top 60 ligand-receptor pairs are shown in fig. S24C.

Query-to-reference mapping

We mapped query data to our prenatal data embeddings using online update of the scVI models following the scArches method (*15*), as implemented in the *scvi-tools* package (*80*). The model was trained for 200 epochs and setting *weight_decay = 0*, to ensure that the latent representation of the reference cells remained exactly the same. Reference genes missing in the query were set to 0, as recommended in (*15*). To generate a joint embedding of query and reference cells, we concatenated the latent dimensions learnt for query cells to the latent dimensions used for the reference embedding and computed the KNN graph and UMAP as described above. To assess that the mapping to the developmental reference conserves biological variation while minimising technical variation in the query data, we compared query cell type labels and batch labels with clusters obtained from Leiden clustering on the learnt latent dimensions, using the Normalized Mutual Information score (see fig. S33 for mapping of adult query data).

Annotation prediction using CellTypist

We used CellTypist v.0.1.9 Python package (*21*) to perform annotation prediction with logistic regression models. For prediction on cycling B cells, the rest of the non-progenitor B cells, including immature B, mature B, B1 and plasma B cells were used as training dataset. Default parameters were used for model building and prediction was made without majority voting for accurate enumeration of predicted B cell subtypes within cycling B cells.

Comparison with human adult immune cells

Single-cell RNA-seq data from adult immune cells was generated and preprocessed as described (*21*). The dataset including cell type annotations were provided by the authors. We mapped 264,929 adult lymphoid cells to the lymphoid embeddings of our developmental dataset and 54,047 adult myeloid cells to our myeloid embedding.

In order to use cell annotations in our developmental dataset to predict adult cell types in the joint embedding, for each adult cell c we identified its k nearest prenatal cell neighbors (N_c) (k=50), and calculated the probability of assigning a label y to adult cell c as

$$\Pr(Y = y \mid X = c, N_c) = \frac{1}{k}\sum_{i=1}^{k} I(y^{(i)} = y)$$

where $y^{(i)}$ is the label of the *i*th nearest neighbor and *I* is the binary indicator function. To label each cell we calculate $\hat{y}_c$ as follows

$$\hat{y}_c = \mathrm{argmax}_y \Pr(Y = y \mid X = c, N_c)$$

and label c as $\hat{y}_c$ if $\Pr(Y = y \mid X = c, N_c) > 0.8$, otherwise c is labeled as "low confidence".

To quantify similarity of adult cells to prenatal cells (fig. S12C, fig. S17C), for each adult cell c, we calculated its similarity to prenatal cells labeled as $\hat{y}_c$ taking the Euclidean distance in the joint embedding, weighted by a Gaussian kernel following the approach described in (*15*).

Blood and immune cell progenitors scRNA-seq data analysis

For the cell fate prediction analysis shown in fig. S20, C and D, we used the Palantir method as implemented in CellRank (*94*, *95*). Briefly, from the scVI embedding on all immune cells (fig. S20A) we selected cells belonging to progenitor populations and computed a KNN graph on scVI latent dimensions on these cells (k=30). Then transition probabilities were calculated using the *ConnectivityKernel* in the *cellrank* package. We computed coarse-grained macrostates with Generalized Perron Cluster Cluster Analysis, setting the number of macrostates to the number of annotated progenitor cell populations. We manually set the four target terminal states for each lineage (small pre B cells, DN(Q) T cells, early MKs, and promonocytes) and computed the probability of each cell to transition to one of the four terminal states. The fate simplex visualization in fig. S20, C and D, was generated using the function *cellrank.pl.circular_projection*.

Artificial thymic organoids scRNA-seq data analysis

Raw scRNA-seq reads were mapped with cellranger 3.0.2 with combined human reference of GRCh38.93 and mouse reference of mm10-3.1.0. Low quality cells were filtered out (minimum number of reads = 2000, minimum number of genes = 500, minimum Scrublet (*77*) doublet detection score <0.4). Cells where the percentage of counts from human genes was <90% were considered as mouse cells and excluded from downstream analysis. Cells were assigned to different cell lines (Kolf, Fiaj) using genotype prediction with souporcell (v.2.4.0) (*78*). We performed batch correction to minimize the differences between cells from different cell lines using scVI and clustered cells using the Leiden algorithm on the latent embedding as described above. We used CellTypist v.0.1.9 Python package (*21*) to perform annotation prediction with logistic regression using the whole in vivo scRNA-seq developmental dataset for training. Stochastic gradient descent was used (setting *use_SGD = True*), and maximum iterations were set to 1000 in model building to reduce the run time. Predicted annotations were then aggregated using a majority voting scheme with *majority_voting = True*, *over_clustering = leiden* in CellTypist prediction to refine cell identities within Leiden clusters. For the in vivo-to-in vitro similarity analysis in fig. S29D, we mapped in vitro cells to the scVI model of lymphoid cells as described above. For each cell in the in vitro dataset we calculated the Euclidean distance (weighted by a Gaussian kernel as described above) to the closest in vivo cell from each in vivo cell population.

Spatial data analysis

Spatial transcriptomics data was mapped using spaceranger v.1.2.1. In parallel, we used a custom image-processing script to identify regions overlapping tissues and retained for analysis the intersection of the tissue spots identified by this pipeline and by tissue calling by spaceranger. To map cell types identified by scRNA-seq in the profiled spatial transcriptomics slides, we used the cell2location method (*16*). Briefly, this consists of two steps. First, for each of the profiled organs we trained a negative binomial regression model to estimate reference transcriptomic profiles for all the cell types profiled with scRNA-seq in the organ. Here we excluded very lowly expressed genes using a recommended filtering strategy (*16*). Cell types where fewer than 20 cells were profiled in the organ of interest and cell types labeled as low-quality cells were excluded from the reference. For the analysis of unconventional T cell localization in thymus (fig. S27C), we trained a reference adding all the prenatal thymic epithelial cells from a thymus cell atlas (*7*) (data was downloaded from Zenodo (*96*)). Next, we estimated the abundance of cell types in the spatial transcriptomics slides using reference transcriptomic profiles of different cell types. All slides representing a given organ were analyzed jointly. Cell2location requires the choice of two hyperparameters: (1) expected cell abundance (*N_cells_per_location = 30*) which was determined by counting average number of nuclei in the histology images corresponding to Visium spots; (2) regularization strength of detection efficiency effect (*detection_alpha = 20*) was used at the low setting to account for variations in RNA detection sensitivity across different spots of Visium slides. The training was stopped after the cell2location model converged, the number of training iterations was 50,000 for thymus, liver, spleen and 30,000 for gut. All other parameters were used at default settings. Cell2location estimates the posterior distribution of cell abundance of every cell type in every spot. Posterior distribution was summarized as 5% quantile, representing the value of cell abundance that the model has high confidence in, and thus incorporating the uncertainty in the estimate into values reported in the paper and used for downstream colocalization analysis.

To identify microenvironments of colocalizing cell types, we used non-negative matrix factorization (NMF) on the matrix of estimated cell type abundances X of dimensions $n \times c$, where n is the total number of spots in the Visium slides and c is the number of cell types in the reference. We decomposed the estimated cell type abundances X as $X = WZ^{\top}$, where Z is a $n \times d$ matrix of latent factor values for each spot and W is a $d \times c$ matrix representing the fraction of abundance of each cell type attributed to each latent factor. Here latent factors correspond to tissue microenvironments defined by a set of colocalized cell types. We use the NMF implementation in scikit-learn (*81*), with the wrapper in the cell2location package, setting the number of factors d = 10. For downstream analysis, we excluded cell types where the 99% quantile of cell abundance across locations in every slide from the same organ was always below the detection threshold of 0.15. Unless otherwise specified, we consider a cell type to be part of a microenvironment if the cell type fraction was over 0.2.

For analysis of mature T cell localization in the thymic medulla (fig. S27, D and E), we retained factors where the sum of the cell type fractions for mature T cells ($CD4^{+}$ T, $CD8^{+}$ T, Treg, type 1 innate T, type 3 innate T, and CD8AA) was above 0.8. We assigned spots to the inner medulla or cortico-medullary microenvironment if the factor value in the spot was above the 90% quantile of all values in the slide. To annotate histological regions in the thymus, we extracted image features from the high resolution images of H&E staining using the python package *squidpy*

(v1.1.2) (*97*) (running *sq.im.calculate_image_features*, with parameters *features = "histogram"*, *spot_scale = 1*, *mask_circle = True*). We scaled and mean centered image feature matrix, and performed Leiden clustering on the first 10 principal components. We manually annotated spot clusters overlapping the thymic cortex and medulla. To define the cortico-medullary junction (CMJ), we detected the spatial neighbors of each spot in medulla or cortex (using the function *squidpy.gr.spatial_neighbours*, with parameters *n_rings = 1*, *coord_type = "grid"*, *n_neighs = 6*). We then labeled a spot as CMJ if it had at least five neighbors (to exclude tissue borders), and if the neighbors included spots from both medulla and cortex regions. For each spot we calculated the distance to the CMJ as the Euclidean distance between the spatial coordinates of the spot and the closest spot annotated as CMJ.

B1 functional validation experiment

Spleens were isolated from two donors, F144 (17 pcw) and F145 (15 pcw). A single-cell suspension was obtained following the protocol described in the "Tissue acquisition and processing" section. Cells were then cryopreserved with 90% FBS and 10% DMSO (Sigma-Aldrich). On the day of the ELISpot experiment, cells were thawed and stained with anti-CD3 (BV510 anti-human CD3 antibody, BD Biosciences, 563109), anti-CD20 (FITC anti-human CD20 antibody, Biolegend, 302303), anti-CD43 (PE anti-human CD43 antibody, BD Biosciences, 560199), anti-CD27 (APC/Cy7 anti-human CD27 antibody, Biolegend, 356424), anti-CD38 (BV711 anti-human CD38 antibody, Biolegend, 303527), anti-CCR10 (APC anti-human CCR10 antibody, Biolegend, 341505) antibodies and DAPI together with control peripheral blood mononuclear cells (PBMC; Stemcell Technologies). B cells were gated as singlet $DAPI^{-}CD3^{-}CD20^{+}$ cells. Plasma cells should generally be $CD20^{lo}$ and therefore not included. To further exclude plasma cell contamination, we also gated out the top 1% of B cells expressing the highest level of CD38. The rest of the B cells were then sorted into four fractions: $CCR10^{hi}$, $CCR10^{lo}CD27^{+}CD43^{+}$, $CCR10^{lo}CD27^{-}CD43^{+}$, and $CCR10^{lo}CD27^{-}CD43^{-}$. CD27 and CD43 gates were chosen based on fluorescence minus one (FMO) controls. The cells were sorted into RPMI supplemented with 10% FBS, penicillin–streptomycin (Gibco) and glutamax (Thermo Fisher Scientific).

The ELISpot experiment was performed with Human IgM $ELISpot^{BASIC}$ kit (ALP) from Mabtech AB. Post sorting, 7000-8000 cells were added into ELISpot plate pre-coated with anti-IgM antibody following manufacturer's instructions and incubated in a 37°C humidified incubator with 5% CO_2 for 22 hours. The plate was then washed and incubated with biotinylated anti-IgM for 2 hours at room temperature, followed by 1-hour incubation of streptavidin-ALP. The colored spots were developed with 15-min incubation of BCIP/NBT substrate solution (Thermo Fisher Scientific). Five rounds of washing were performed between each step of incubation as per manufacturer's instructions. After the colored spots appeared clearly, the reaction was then stopped by rinsing under running tap water for 5 min. Spots were counted with the AID ELISpot reader and iSpot software version 4.

In addition, we performed scRNA-seq of the sorted B cell fractions on a different donor (F149, 18 pcw fetal spleen), using the same gating strategy to further confirm the identity of sorted cells. The scRNA-seq data was preprocessed with scVI as above. Cell annotations were predicted using CellTypist v.0.1.9 (*21*), initially with our original developmental dataset as the training data to find the non-B cells, then with only the non-cycling mature B cells from the developmental dataset as the training data to map the B cells in the sorted data. Default parameters were used for model building and prediction was made with majority voting.

Supplementary Figures

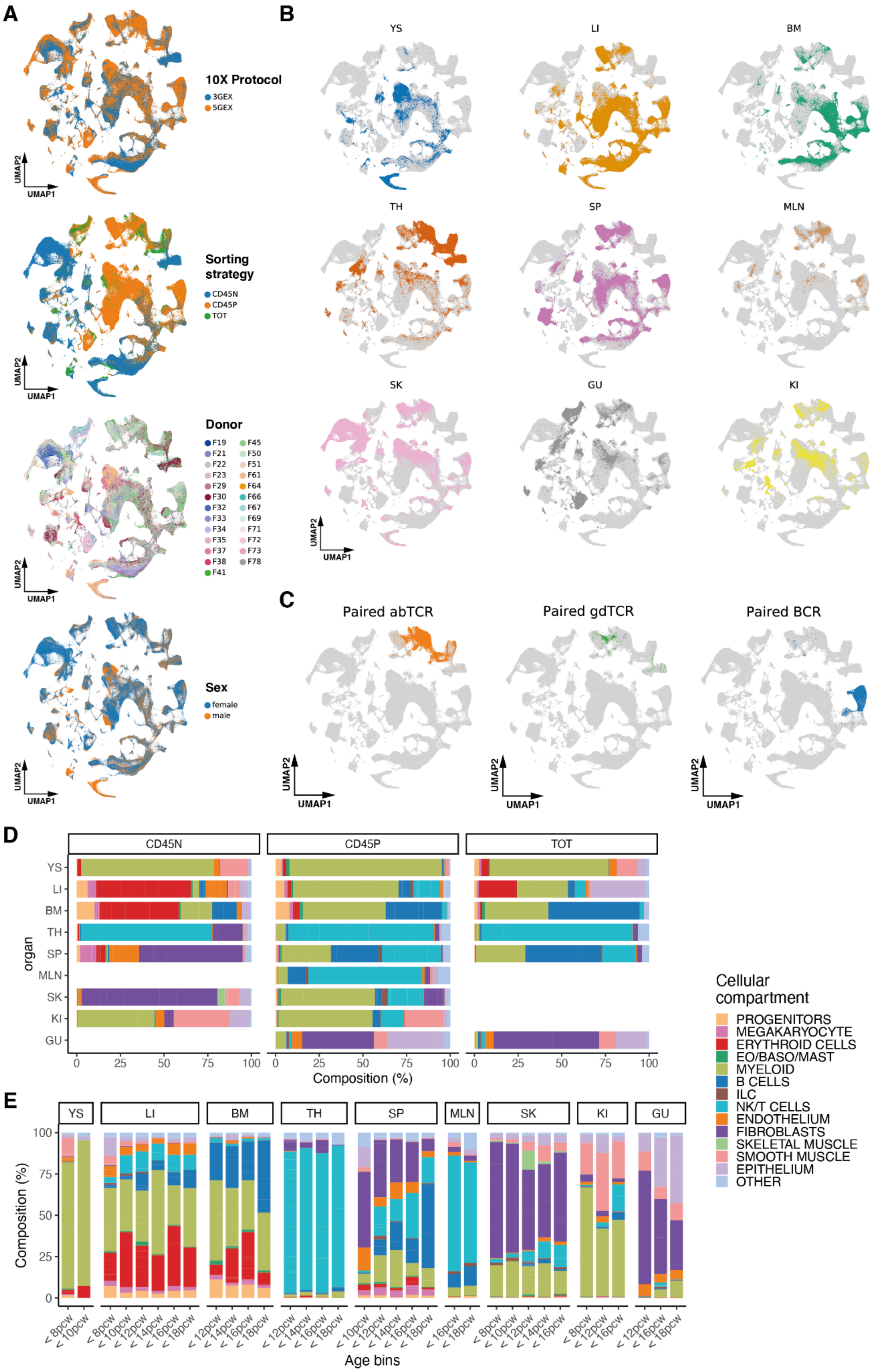
A
10X Protocol
3GEX
5GEX
Sorting strategy
CD45N
CD45P
TOT
Donor
F19
F21
F22
F23
F29
F30
F32
F33
F34
F35
F37
F38
F41
F45
F50
F51
F61
F64
F66
F67
F69
F71
F72
F73
F78
Sex
female
male
UMAP1
UMAP2
B
YS
LI
BM
TH
SP
MLN
SK
GU
KI
C
Paired abTCR
Paired gdTCR
Paired BCR
D
CD45N
CD45P
TOT
organ
Composition (%)
0
25
50
75
100
Cellular compartment
PROGENITORS
MEGAKARYOCYTE
ERYTHROID CELLS
EO/BASO/MAST
MYELOID
B CELLS
ILC
NK/T CELLS
ENDOTHELIUM
FIBROBLASTS
SKELETAL MUSCLE
SMOOTH MUSCLE
EPITHELIUM
OTHER
E
Composition (%)
Age bins
< 8pcw
< 10pcw
< 12pcw
< 14pcw
< 16pcw
< 18pcw

fig. S1: Characterization of cross-organ developmental scRNA-seq atlas. (**A**) Sample characteristics in integrated atlas. UMAP embeddings (as in Fig. 1C) of scRNA-seq profiles colored by (top to bottom): 10X chemistry protocol, FACS protocol (CD45P: $CD45^{+}$; CD45N: $CD45^{-}$; TOT: unsorted), donor ID, sex of donor. (**B**) Distribution of cells from different organs in integrated atlas. UMAP embeddings (as in Fig. 1C) of scRNA-seq profiles, highlighting cells from each organ. (**C**) UMAP embeddings (as in Fig. 1C) of scRNA-seq profiles, highlighting cells for which paired αβTCR, γδTCR or BCR sequences were detected. (**D**) Percentage of cells of each broad type in each organ, stratified by FACS protocol. (**E**) Percentage of cells of each broad type in each gestational age group, stratified by organ. (YS: yolk sac; LI: liver; BM: bone marrow; TH: thymus; SP: spleen; SK: skin; GU: gut; KI: kidney).

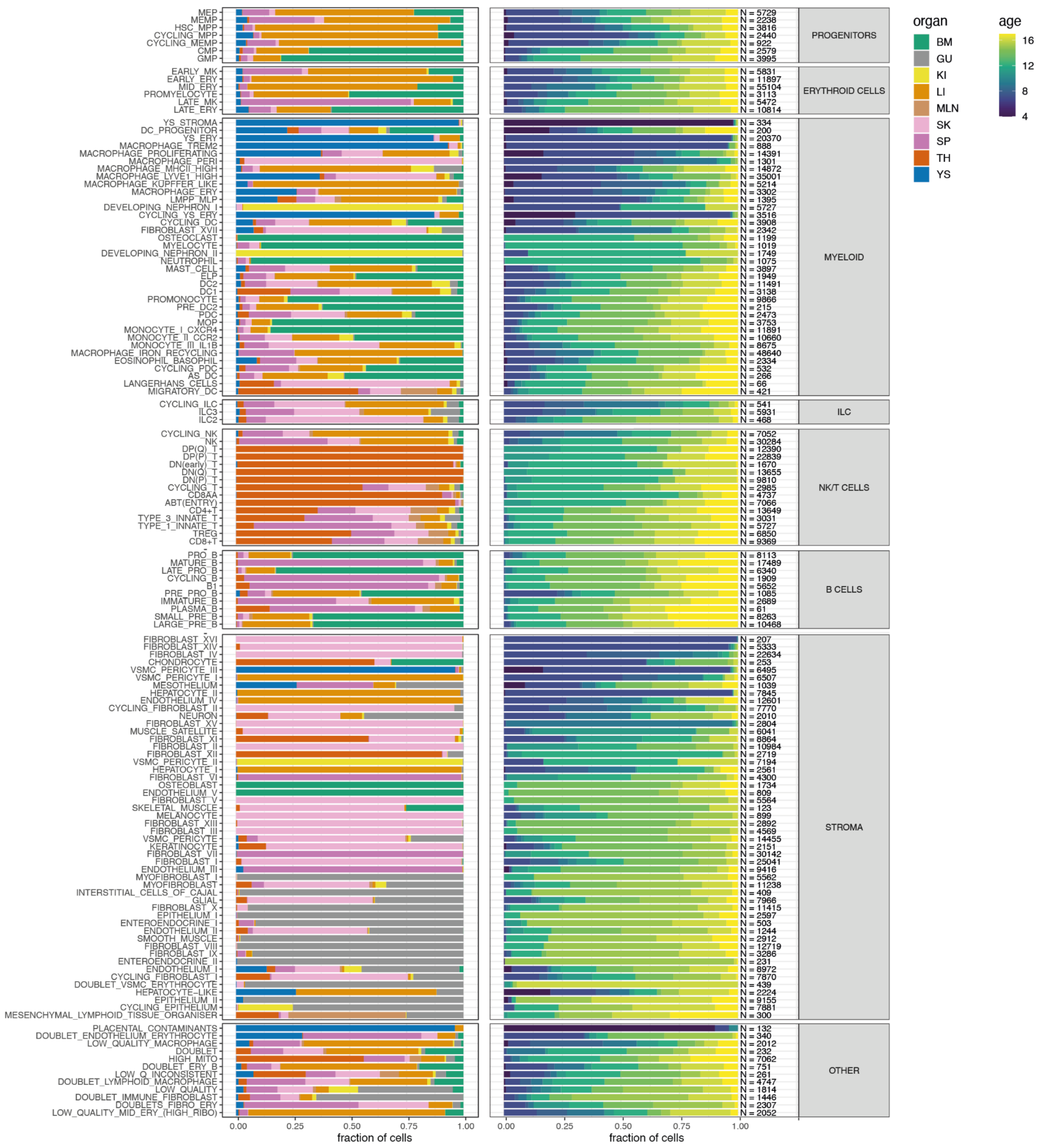

fig. S2: Distribution across organs (left) and gestational age (pcw, right) of annotated cell populations. Cell populations are grouped according to broad population annotations. The category "Other" denotes clusters annotated as low-quality cells. N indicates the total number of cells across the dataset for each annotation. (YS: yolk sac; LI: liver; BM: bone marrow; TH: thymus; SP: spleen; SK: skin; GU: gut; KI: kidney).

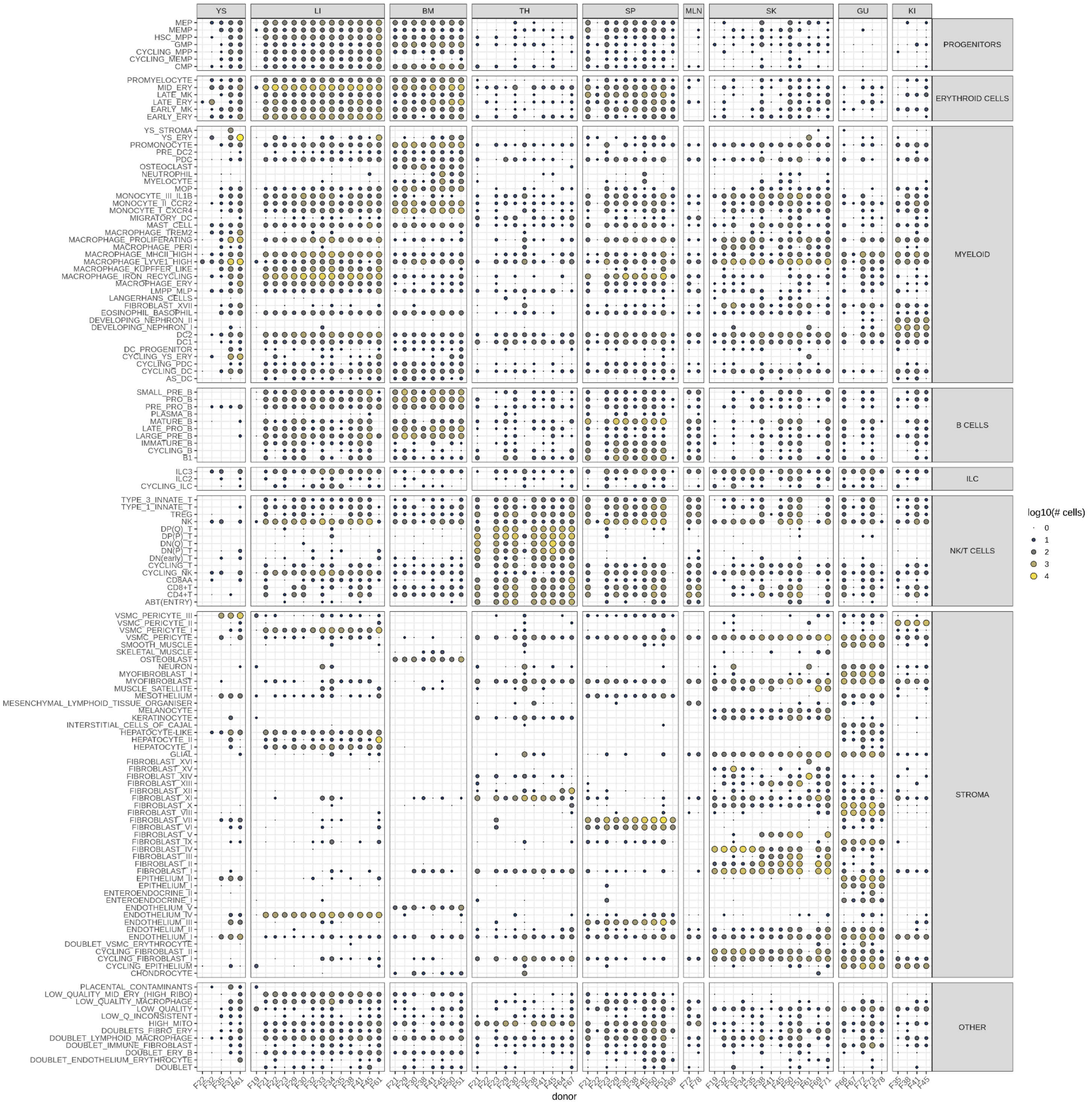

fig. S3: Consistency across donors of annotated cell populations. Dot size and color are proportional to the number of cells of the annotated population from each donor and organ. Cell populations are grouped according to broad population annotations. The category "Other" denotes clusters annotated as low-quality cells.

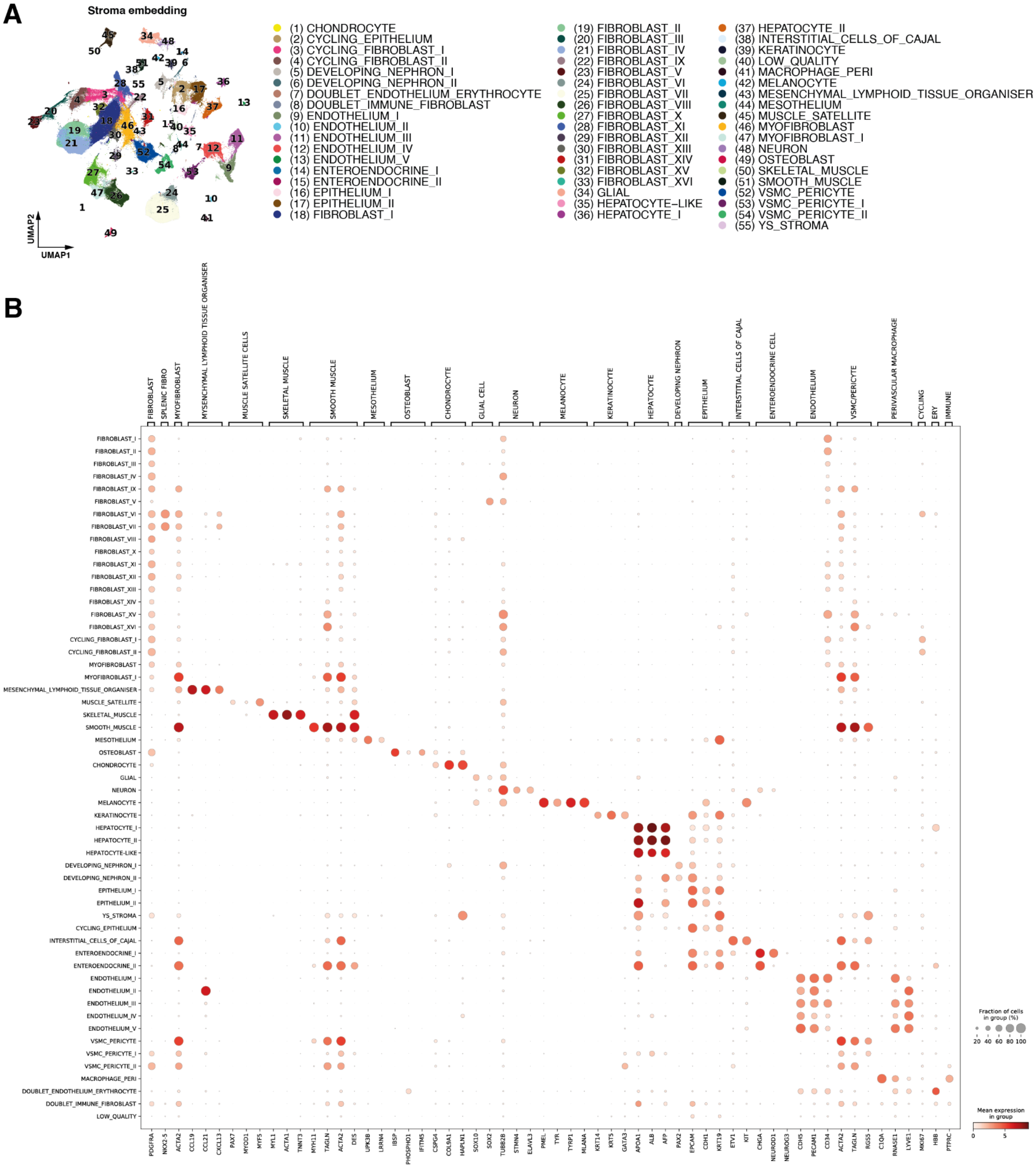
A
Stroma embedding
UMAP2
UMAP1
(1) CHONDROCYTE
(2) CYCLING_EPITHELIUM
(3) CYCLING_FIBROBLAST_I
(4) CYCLING_FIBROBLAST_II
(5) DEVELOPING_NEPHRON_I
(6) DEVELOPING_NEPHRON_II
(7) DOUBLET_ENDOTHELIUM_ERYTHROCYTE
(8) DOUBLET_IMMUNE_FIBROBLAST
(9) ENDOTHELIUM_I
(10) ENDOTHELIUM_II
(11) ENDOTHELIUM_III
(12) ENDOTHELIUM_IV
(13) ENDOTHELIUM_V
(14) ENTEROENDOCRINE_I
(15) ENTEROENDOCRINE_II
(16) EPITHELIUM_I
(17) EPITHELIUM_II
(18) FIBROBLAST_I
(19) FIBROBLAST_II
(20) FIBROBLAST_III
(21) FIBROBLAST_IV
(22) FIBROBLAST_IX
(23) FIBROBLAST_V
(24) FIBROBLAST_VI
(25) FIBROBLAST_VII
(26) FIBROBLAST_VIII
(27) FIBROBLAST_X
(28) FIBROBLAST_XI
(29) FIBROBLAST_XII
(30) FIBROBLAST_XIII
(31) FIBROBLAST_XIV
(32) FIBROBLAST_XV
(33) FIBROBLAST_XVI
(34) GLIAL
(35) HEPATOCYTE-LIKE
(36) HEPATOCYTE_I
(37) HEPATOCYTE_II
(38) INTERSTITIAL_CELLS_OF_CAJAL
(39) KERATINOCYTE
(40) LOW_QUALITY
(41) MACROPHAGE_PERI
(42) MELANOCYTE
(43) MESENCHYMAL_LYMPHOID_TISSUE_ORGANISER
(44) MESOTHELIUM
(45) MUSCLE_SATELLITE
(46) MYOFIBROBLAST
(47) MYOFIBROBLAST_I
(48) NEURON
(49) OSTEOBLAST
(50) SKELETAL_MUSCLE
(51) SMOOTH_MUSCLE
(52) VSMC_PERICYTE
(53) VSMC_PERICYTE_I
(54) VSMC_PERICYTE_II
(55) YS_STROMA
B
FIBROBLAST
SPLENIC FIBRO
MYOFIBROBLAST
MYSENCHYMAL LYMPHOID TISSUE ORGANISER
MUSCLE SATELLITE CELLS
SKELETAL MUSCLE
SMOOTH MUSCLE
MESOTHELIUM
OSTEOBLAST
CHONDROCYTE
GLIAL CELL
NEURON
MELANOCYTE
KERATINOCYTE
HEPATOCYTE
DEVELOPING NEPHRON
EPITHELIUM
INTERSTITIAL CELLS OF CAJAL
ENTEROENDOCRINE CELL
ENDOTHELIUM
VSMC/PERICYTE
PERIVASCULAR MACROPHAGE
CYCLING
ERY
IMMUNE
FIBROBLAST_I
FIBROBLAST_II
FIBROBLAST_III
FIBROBLAST_IV
FIBROBLAST_IX
FIBROBLAST_V
FIBROBLAST_VI
FIBROBLAST_VII
FIBROBLAST_VIII
FIBROBLAST_X
FIBROBLAST_XI
FIBROBLAST_XII
FIBROBLAST_XIII
FIBROBLAST_XIV
FIBROBLAST_XV
FIBROBLAST_XVI
CYCLING_FIBROBLAST_I
CYCLING_FIBROBLAST_II
MYOFIBROBLAST
MYOFIBROBLAST_I
MESENCHYMAL_LYMPHOID_TISSUE_ORGANISER
MUSCLE_SATELLITE
SKELETAL_MUSCLE
SMOOTH_MUSCLE
MESOTHELIUM
OSTEOBLAST
CHONDROCYTE
GLIAL
NEURON
MELANOCYTE
KERATINOCYTE
HEPATOCYTE_I
HEPATOCYTE_II
HEPATOCYTE-LIKE
DEVELOPING_NEPHRON_I
DEVELOPING_NEPHRON_II
EPITHELIUM_I
EPITHELIUM_II
YS_STROMA
CYCLING_EPITHELIUM
INTERSTITIAL_CELLS_OF_CAJAL
ENTEROENDOCRINE_I
ENTEROENDOCRINE_II
ENDOTHELIUM_I
ENDOTHELIUM_II
ENDOTHELIUM_III
ENDOTHELIUM_IV
ENDOTHELIUM_V
VSMC_PERICYTE
VSMC_PERICYTE_I
VSMC_PERICYTE_II
MACROPHAGE_PERI
DOUBLET_ENDOTHELIUM_ERYTHROCYTE
DOUBLET_IMMUNE_FIBROBLAST
LOW_QUALITY
PDGFRA NKX2-5 ACTA2 CCL19 CCL21 CXCL13 PAX7 MYOD1 MYF5 MYL1 ACTA1 TNNT3 MYH11 TAGLN ACTA2 DES UPK3B LRRN4 IBSP PHOSPHO1 IFITM5 CSPG4 COL9A1 HAPLN1 SOX10 SOX2 TUBB2B STMN4 ELAVL3 PMEL TYR TYRP1 MLANA KRT14 KRT5 GATA3 APOA1 ALB AFP PAX2 EPCAM CDH1 KRT19 ETV1 KIT CHGA NEUROD1 NEUROG3 CDH5 PECAM1 CD34 ACTA2 TAGLN RGS5 C1QA RNASE1 LYVE1 MKI67 HBB PTPRC
Fraction of cells in group (%)
20 40 60 80 100
Mean expression in group
0 5

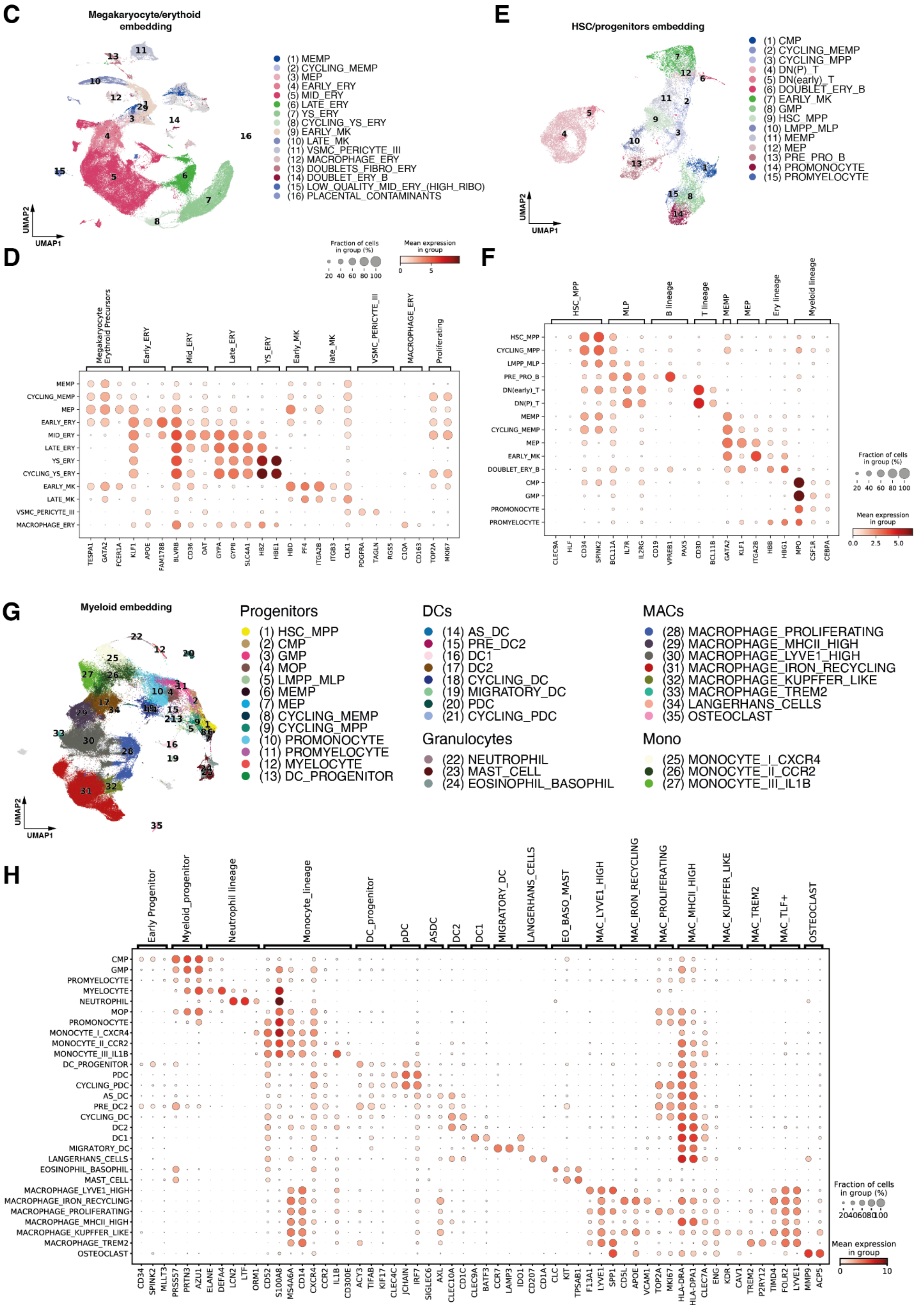
C
Megakaryocyte/erythoid embedding
(1) MEMP
(2) CYCLING_MEMP
(3) MEP
(4) EARLY_ERY
(5) MID_ERY
(6) LATE_ERY
(7) YS_ERY
(8) CYCLING_YS_ERY
(9) EARLY_MK
(10) LATE_MK
(11) VSMC_PERICYTE_III
(12) MACROPHAGE_ERY
(13) DOUBLETS_FIBRO_ERY
(14) DOUBLET_ERY_B
(15) LOW_QUALITY_MID_ERY_(HIGH_RIBO)
(16) PLACENTAL_CONTAMINANTS
UMAP2
UMAP1
E
HSC/progenitors embedding
(1) CMP
(2) CYCLING_MEMP
(3) CYCLING_MPP
(4) DN(P)_T
(5) DN(early)_T
(6) DOUBLET_ERY_B
(7) EARLY_MK
(8) GMP
(9) HSC_MPP
(10) LMPP_MLP
(11) MEMP
(12) MEP
(13) PRE_PRO_B
(14) PROMONOCYTE
(15) PROMYELOCYTE
UMAP2
UMAP1
D
Fraction of cells in group (%)
20 40 60 80 100
Mean expression in group
0 5
Megakaryocyte Erythroid Precursors
Early_ERY
Mid_ERY
Late_ERY
YS_ERY
Early_MK
late_MK
VSMC_PERICYTE_III
MACROPHAGE_ERY
Proliferating
MEMP
CYCLING_MEMP
MEP
EARLY_ERY
MID_ERY
LATE_ERY
YS_ERY
CYCLING_YS_ERY
EARLY_MK
LATE_MK
VSMC_PERICYTE_III
MACROPHAGE_ERY
TESPA1
GATA2
FCER1A
KLF1
APOE
FAM178B
BLVRB
CD36
OAT
GYPA
GYPB
SLC4A1
HBZ
HBE1
HBD
PF4
ITGA2B
ITGB3
CLK1
PDGFRA
TAGLN
RGS5
C1QA
CD163
TOP2A
MKI67
F
HSC_MPP
MLP
B lineage
T lineage
MEMP
MEP
Ery lineage
Myeloid lineage
HSC_MPP
CYCLING_MPP
LMPP_MLP
PRE_PRO_B
DN(early)_T
DN(P)_T
MEMP
CYCLING_MEMP
MEP
EARLY_MK
DOUBLET_ERY_B
CMP
GMP
PROMONOCYTE
PROMYELOCYTE
CLEC9A
HLF
CD34
SPINK2
BCL11A
IL7R
IL2RG
CD19
VPREB1
PAX5
CD3D
BCL11B
GATA2
KLF1
ITGA2B
HBB
HBG1
MPO
CSF1R
CEBPA
Fraction of cells in group (%)
20 40 60 80 100
Mean expression in group
0.0 2.5 5.0
G
Myeloid embedding
Progenitors
(1) HSC_MPP
(2) CMP
(3) GMP
(4) MOP
(5) LMPP_MLP
(6) MEMP
(7) MEP
(8) CYCLING_MEMP
(9) CYCLING_MPP
(10) PROMONOCYTE
(11) PROMYELOCYTE
(12) MYELOCYTE
(13) DC_PROGENITOR
DCs
(14) AS_DC
(15) PRE_DC2
(16) DC1
(17) DC2
(18) CYCLING_DC
(19) MIGRATORY_DC
(20) PDC
(21) CYCLING_PDC
Granulocytes
(22) NEUTROPHIL
(23) MAST_CELL
(24) EOSINOPHIL_BASOPHIL
MACs
(28) MACROPHAGE_PROLIFERATING
(29) MACROPHAGE_MHCII_HIGH
(30) MACROPHAGE_LYVE1_HIGH
(31) MACROPHAGE_IRON_RECYCLING
(32) MACROPHAGE_KUPFFER_LIKE
(33) MACROPHAGE_TREM2
(34) LANGERHANS_CELLS
(35) OSTEOCLAST
Mono
(25) MONOCYTE_I_CXCR4
(26) MONOCYTE_II_CCR2
(27) MONOCYTE_III_IL1B
UMAP2
UMAP1
H
Early Progenitor
Myeloid_progenitor
Neutrophil lineage
Monocyte_lineage
DC_progenitor
pDC
ASDC
DC2
DC1
MIGRATORY_DC
LANGERHANS_CELLS
EO_BASO_MAST
MAC_LYVE1_HIGH
MAC_IRON_RECYCLING
MAC_PROLIFERATING
MAC_MHCII_HIGH
MAC_KUPFFER_LIKE
MAC_TREM2
MAC_TLF+
OSTEOCLAST
CMP
GMP
PROMYELOCYTE
MYELOCYTE
NEUTROPHIL
MOP
PROMONOCYTE
MONOCYTE_I_CXCR4
MONOCYTE_II_CCR2
MONOCYTE_III_IL1B
DC_PROGENITOR
PDC
CYCLING_PDC
AS_DC
PRE_DC2
CYCLING_DC
DC2
DC1
MIGRATORY_DC
LANGERHANS_CELLS
EOSINOPHIL_BASOPHIL
MAST_CELL
MACROPHAGE_LYVE1_HIGH
MACROPHAGE_IRON_RECYCLING
MACROPHAGE_PROLIFERATING
MACROPHAGE_MHCII_HIGH
MACROPHAGE_KUPFFER_LIKE
MACROPHAGE_TREM2
OSTEOCLAST
CD34
SPINK2
MLLT3
PRSS57
PRTN3
AZU1
ELANE
DEFA4
LCN2
LTF
ORM1
CD52
S100A8
MS4A6A
CD14
CXCR4
CCR2
IL1B
CD300E
ACY3
TIFAB
KIF17
CLEC4C
JCHAIN
IRF7
SIGLEC6
AXL
CLEC10A
CD1C
CLEC9A
BATF3
CCR7
LAMP3
IDO1
CD207
CD1A
CLC
KIT
TPSAB1
F13A1
LYVE1
SPP1
CD5L
APOE
VCAM1
TOP2A
MKI67
HLA-DRA
HLA-DPA1
CLEC7A
ENG
KDR
CAV1
TREM2
P2RY12
TIMD4
FOLR2
LYVE1
MMP9
ACP5
Fraction of cells in group (%)
20406080100
Mean expression in group
0 10

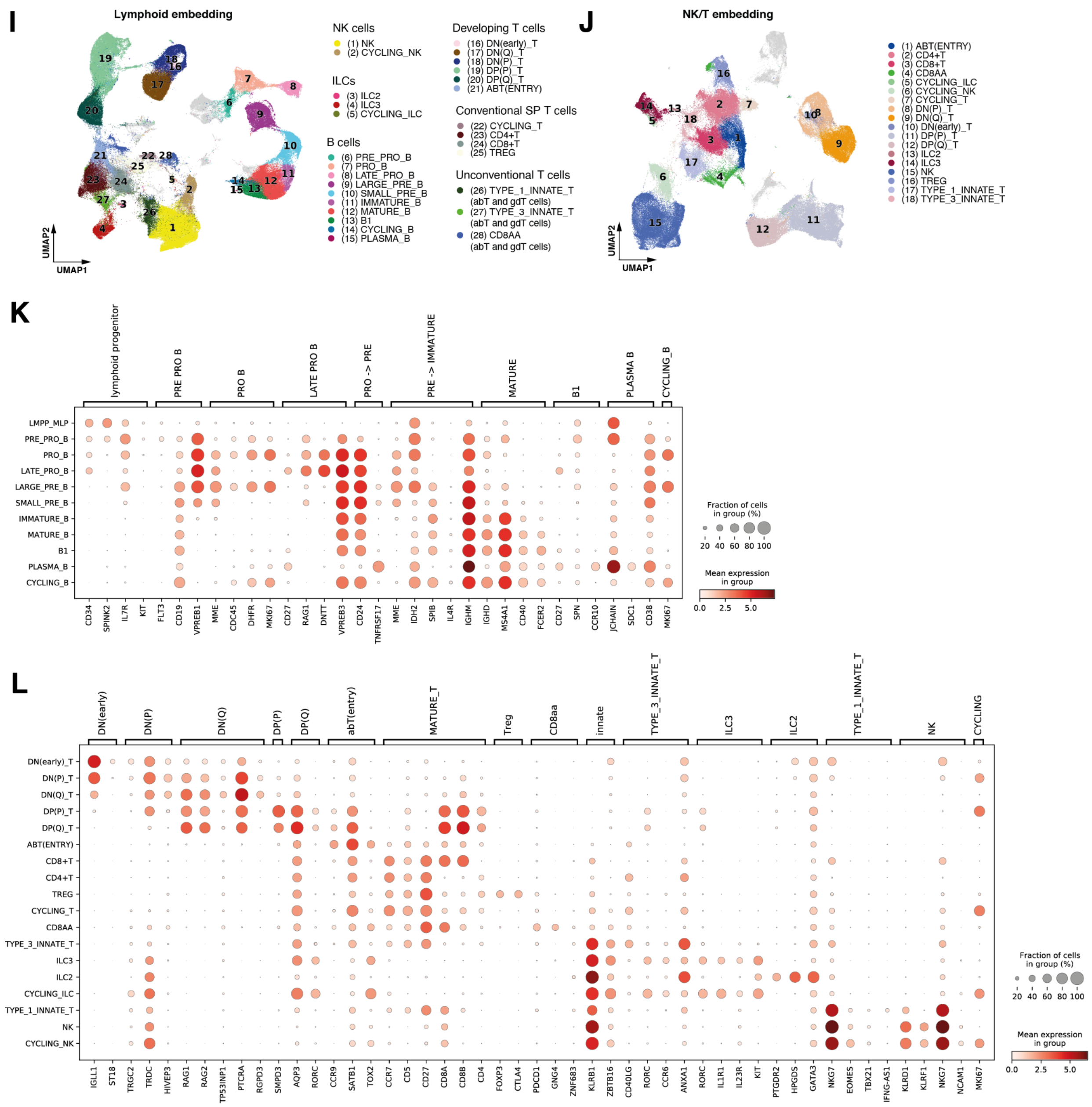

fig. S4: Cross-tissue annotation of hierarchical subsets of scRNA-seq integrated dataset. For each subset embedding generated through scVI, we show UMAP embeddings of cells colored by annotated cell populations and dot plots of mean expression (log-normalized counts, dot color) and fraction of expressing cells (dot size) of marker genes (columns) used for cell population annotation (rows). (**A** and **B**) Annotation of stromal cells. (**C** and **D**) Annotation of megakaryocyte and erythroid cells (cells in gray are progenitors annotated through embedding shown in (E)). (**E** and **F**) Annotation of hematopoietic and immune cell progenitors. (**G** and **H**) Annotation of myeloid cells (cells in gray are progenitors annotated through embedding shown in (E) or low-quality clusters) (**I** and **L**) Annotation of lymphoid cells (cells in gray are progenitors annotated through embedding shown in (E) or low-quality clusters). The embedding of all lymphoid cells is shown in (I), the embedding used for annotation of NK/T cells is visualized in (J). The dot plot for annotation of B cells is shown in (K), and the dot plot for annotation of T cells is displayed in (L).

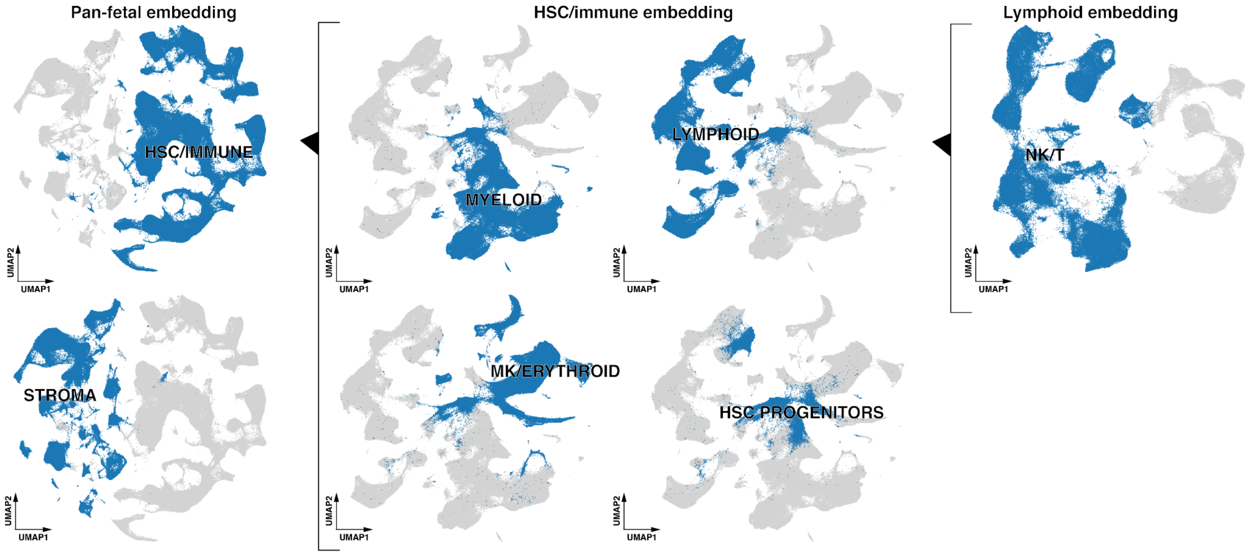

fig. S5: Overview of the hierarchical subsetting strategy used for annotation of fine immune subtypes. In each embedding, the cells that make up the data views in fig. S4 are highlighted in blue.

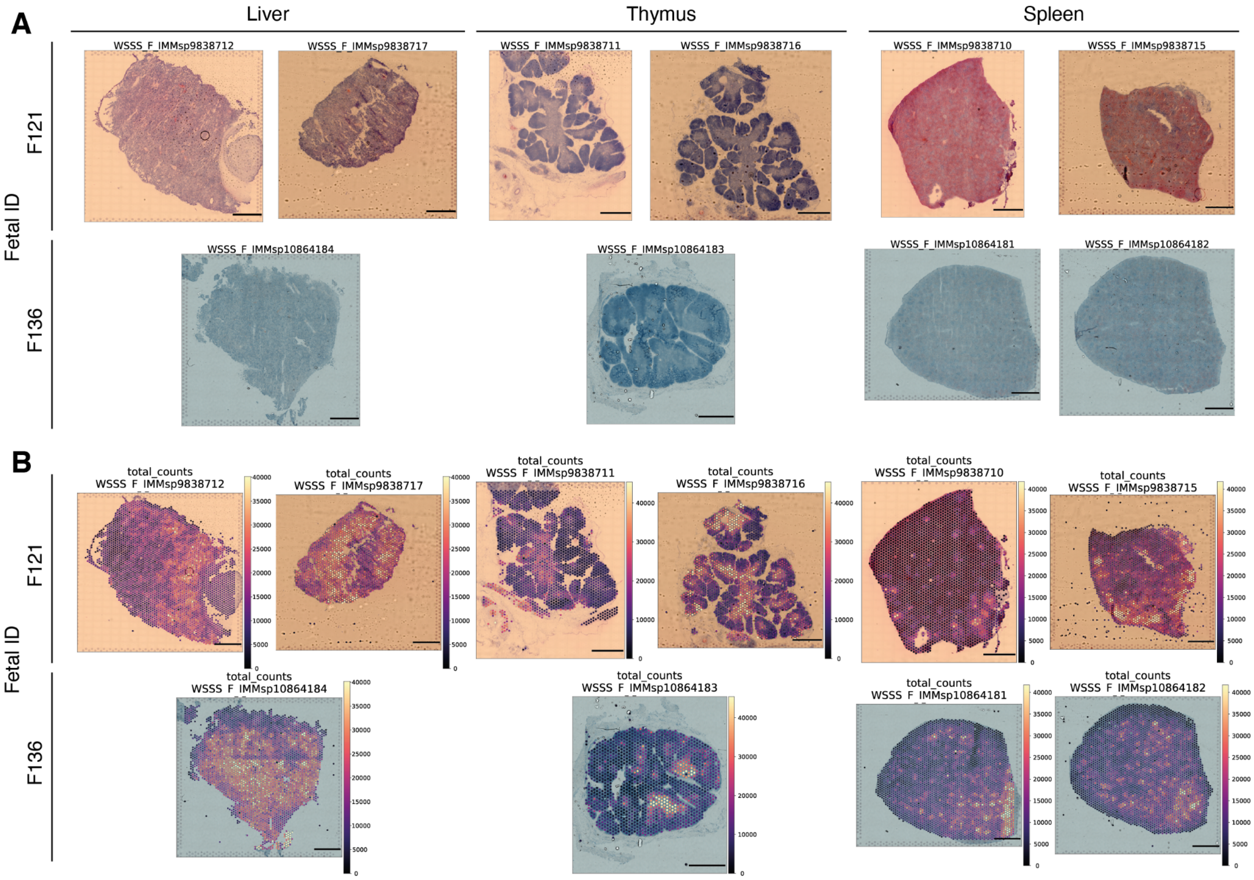

fig. S6: Experimental design and library QC for Visium 10X data (**A**) H&E staining of tissue slides processed for spatial transcriptomics with Visium 10X protocol. Slides are grouped by organ (columns) and embryo/fetus ID (rows). (**B**) Total RNA counts in analyzed tissue spots (scale bar: 1 mm).

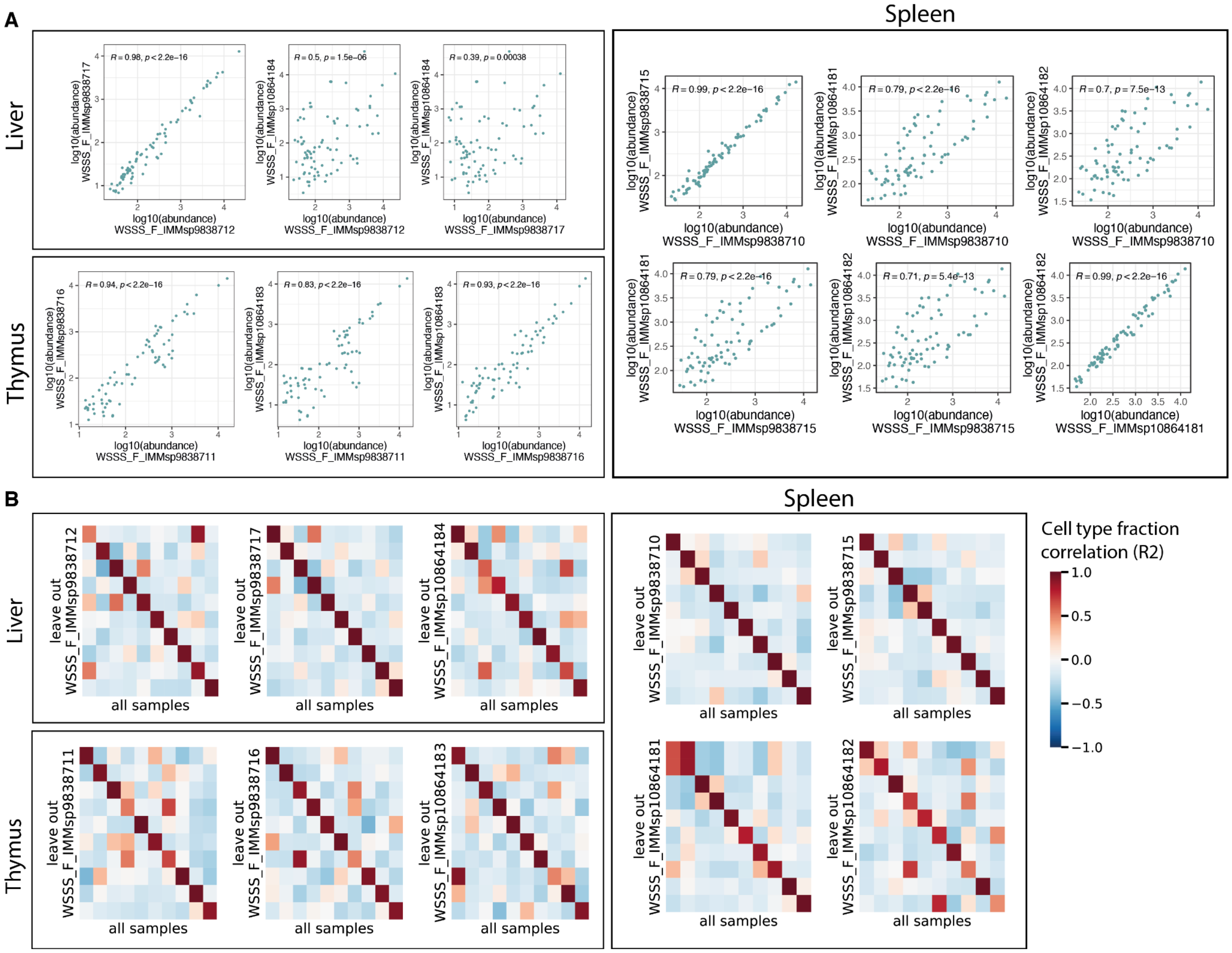

fig. S7: Robustness of spatial cell type abundance predictions with cell2location. (**A**) Analysis of robustness of cell type mapping with cell2location: for mapping on each organ we correlate the total abundance (in $\log_{10}$ scale) of each cell type (points) in different tissue slides from the same organ (biological replicates). The Pearson correlation coefficient and *P*-value for permutation test are reported. (**B**) Robustness of colocation predictions with NMF. We compare the NMF model learnt on all samples from the same organ with NMF models learnt leaving one sample out (as indicated by *y*-axis label). The color of the heatmaps represents the Pearson correlation between cell type fraction attributed to each factor in the compared models.

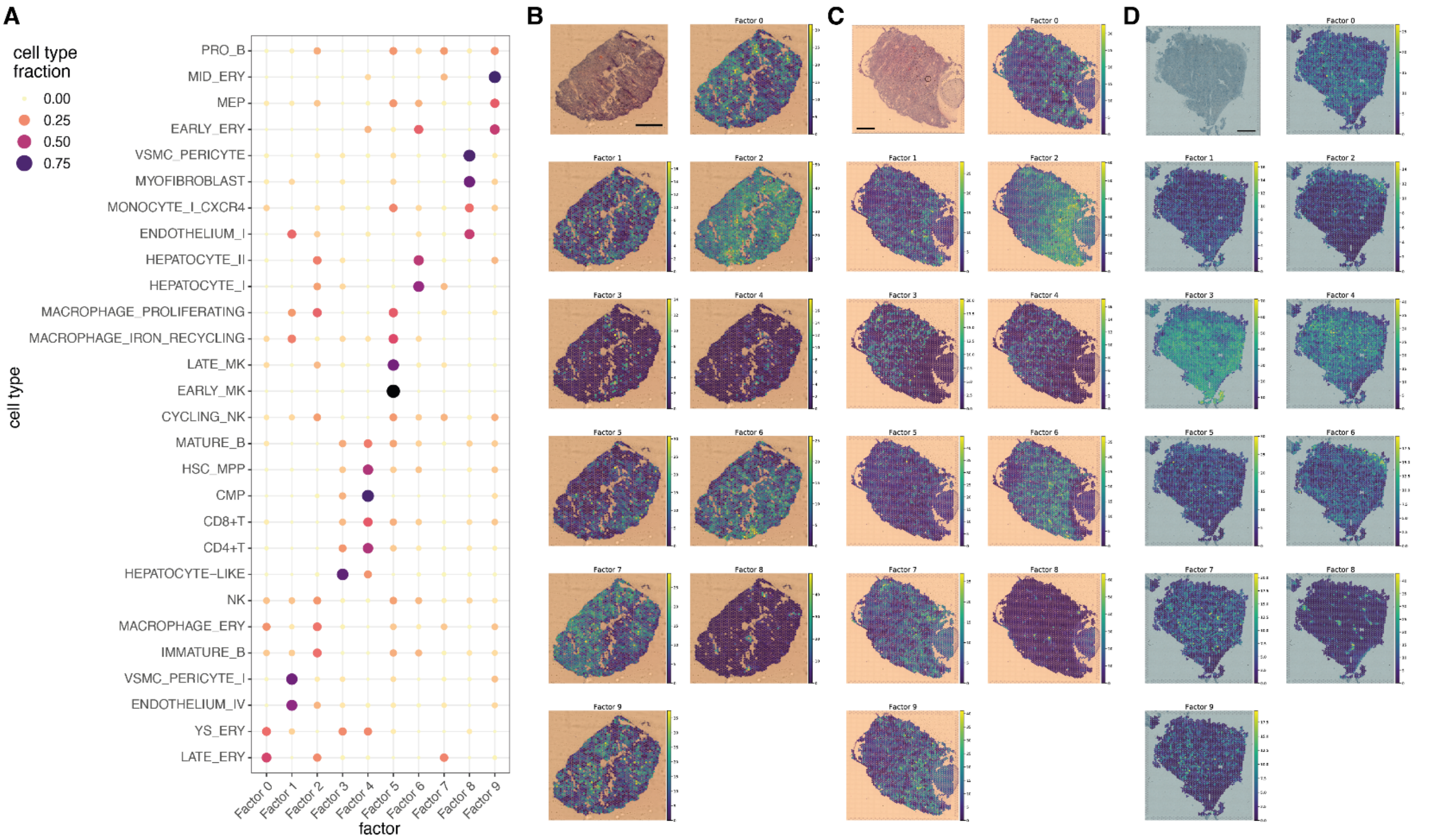

fig. S8: Cell type spatial microenvironments in the fetal liver detected by non-negative matrix factorization on spatial cell type abundances. (**A**) Dot plot of cell type contributions to latent factors (microenvironments) identified with non-negative matrix factorization of spatial cell type abundances estimated with cell2location. The color and the size of the dots represent the relative fraction of the cell population assigned to the factor. We exclude cell types where the value for the 99% quantile of cell abundance in all the slides from the same organ is always below the detection threshold of 0.15. (**B** to **D**) Spatial locations of microenvironments on liver slides, with the color representing the weighted contribution of each microenvironment to each spot. H&E staining images for each slide are shown for reference (scale bar: 1 mm).

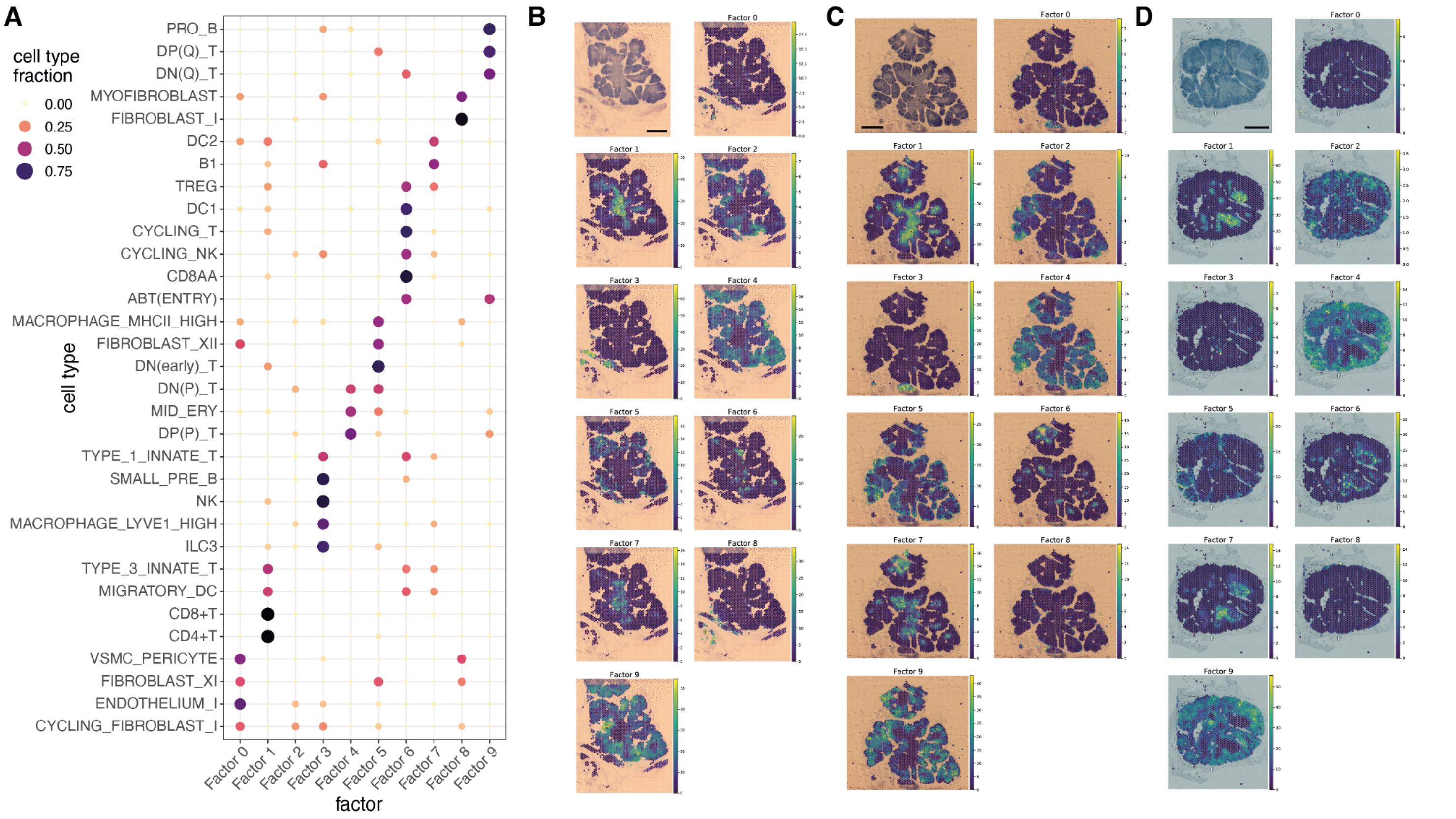

fig. S9: Cell type spatial microenvironments in the fetal thymus detected by non-negative matrix factorization on spatial abundances. (**A**) Dot plot of cell type contributions to latent factors (microenvironments) identified with non-negative matrix factorization of spatial cell type abundances estimated with cell2location. The color and the size of the dots represent the relative fraction of the cell population assigned to the factor. We exclude cell types where the value for the 99% quantile of cell abundance in all the slides from the same organ is always below the detection threshold of 0.15. (**B** to **D**) Spatial locations of microenvironments on thymus slides, with the color representing the weighted contribution of each microenvironment to each spot. H&E staining images for each slide are shown for reference (scale bar: 1 mm).

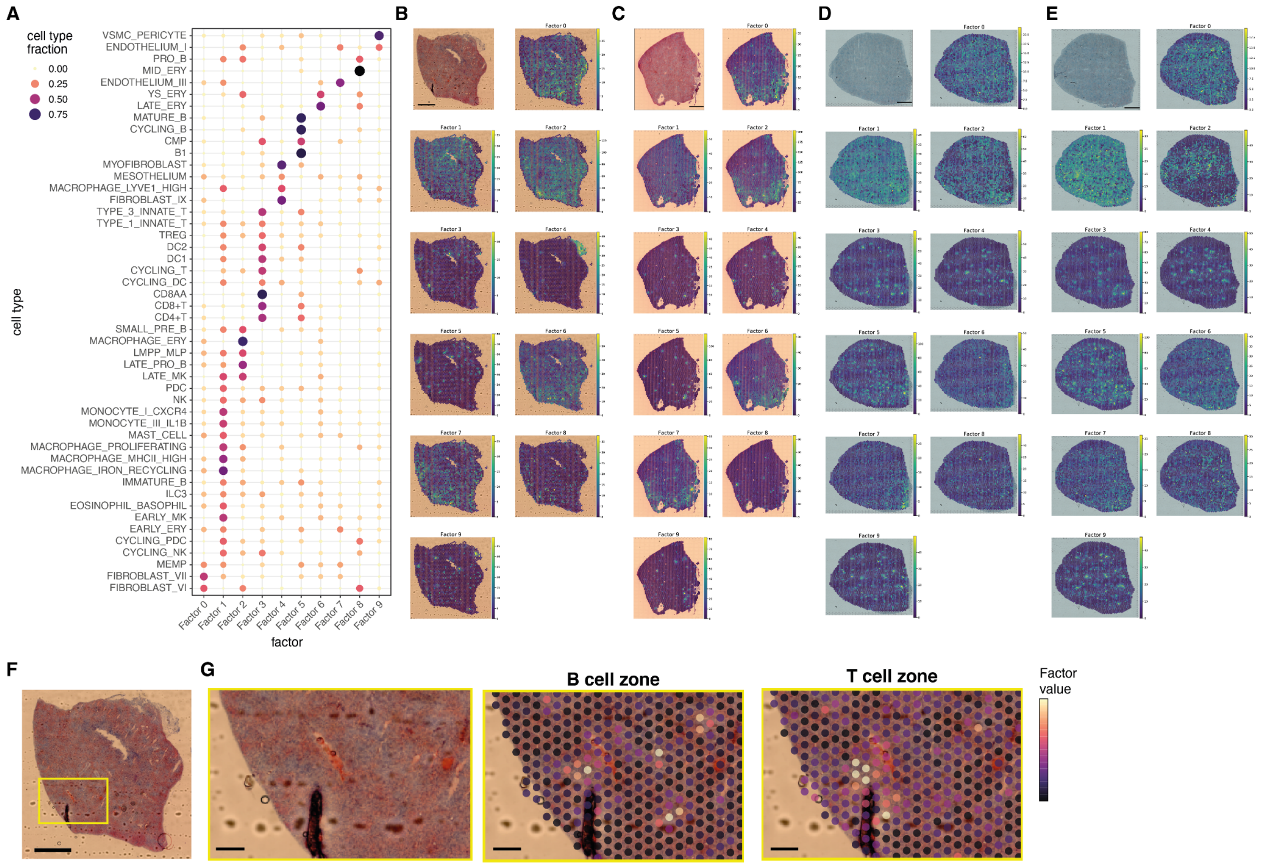

fig. S10: Cell type spatial microenvironments in the fetal spleen detected by non-negative matrix factorization on spatial abundances. (**A**) Dot plot of cell type contributions to latent factors (microenvironments) identified with non-negative matrix factorization of spatial cell type abundances estimated with cell2location. The color and the size of the dots represent the relative fraction of the cell population assigned to the factor. We exclude cell types where the value for the 99% quantile of cell abundance in all the slides from the same organ is always below the detection threshold of 0.15. (**B** to **E**) Spatial locations of microenvironments on spleen slides, with the color representing the weighted contribution of each microenvironment to each spot. H&E staining images for each slide are shown for reference (scale bar: 1 mm). (**F**) Illustration of inset displayed in G of fetal spleen tissue slide shown in B (scale bar: 1 mm). (**G**) Higher magnification view of slide in F showing weighted microenvironment contribution (factor values) of lymphoid aggregates B cell zone microenvironment (Factor 8) and T cell zone microenvironment (Factor 9) (scale bar: 200 μm). These exemplify how the B and T cell zones were proximal to each other but did not completely overlap.

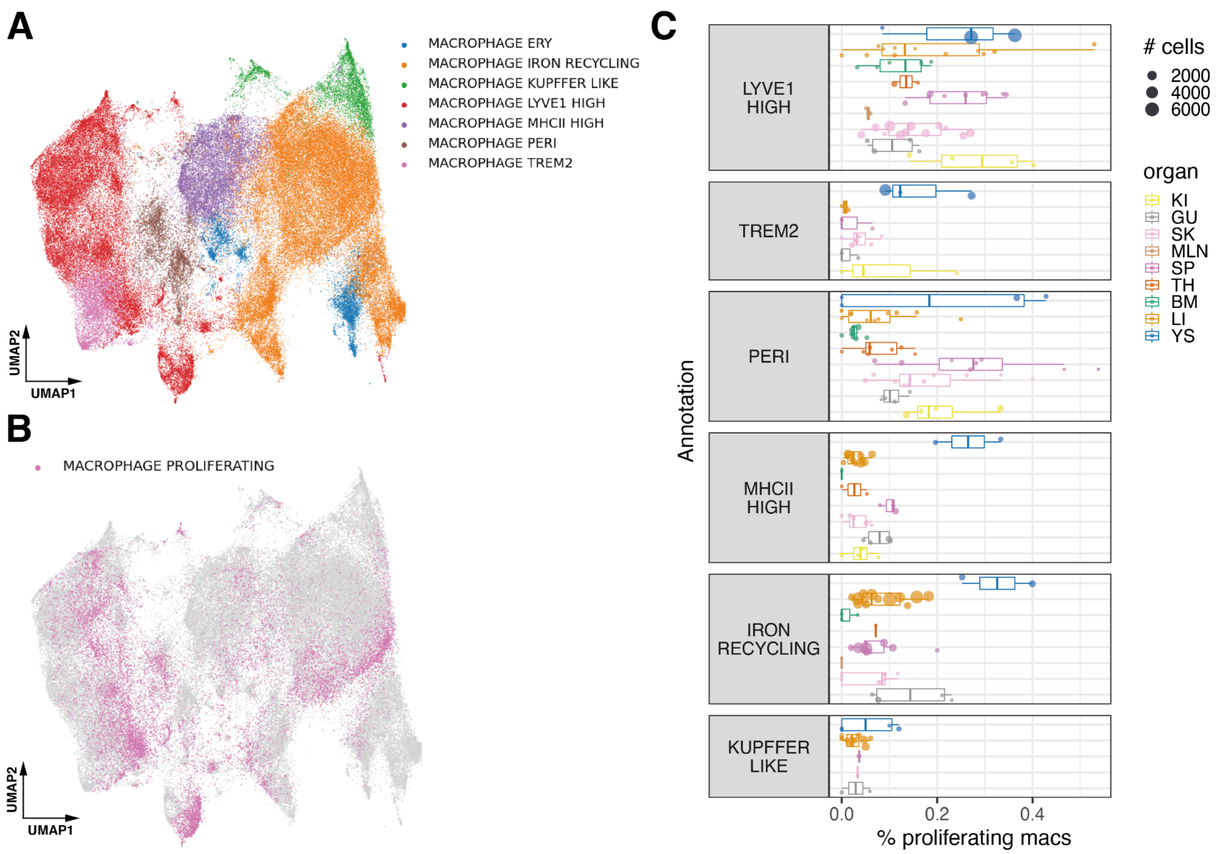

fig. S11: Distribution of proliferating macrophages. (**A**) UMAP embedding of macrophage cells colored by annotated subpopulations. Dimensionality reduction with scVI, KNN graph construction and UMAP embedding were performed on macrophages as described for other subsets. We repeated Leiden clustering on this embedding and propagated labels from the annotations obtained on the myeloid embedding. (**B**) Distribution and mixing of proliferating ($MKI67^{+}TOP2A^{+}$) macrophages identified in myeloid clustering (fig. S4G). (**C**) Fraction of proliferating macrophages within each macrophage subpopulation defined as in (A). Each point represents one embryo/fetus, color-coded by organ. The size of the point represents the cell count (YS: yolk sac; LI: liver; BM: bone marrow; TH: thymus; SP: spleen; MLN: mesenteric lymph node; SK: skin; GU: gut; KI: kidney).

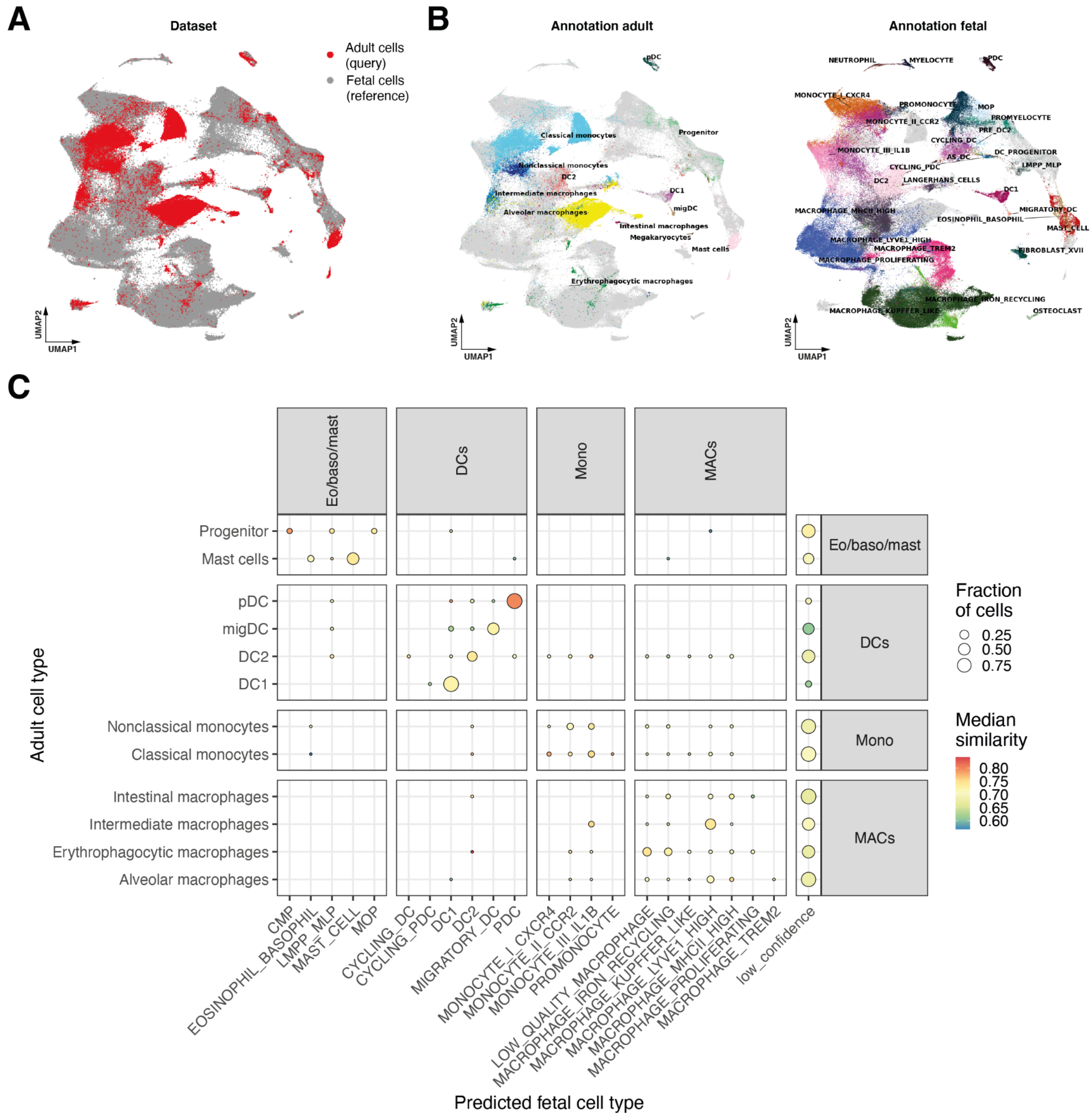

fig. S12: Mapping of adult myeloid cells to prenatal reference with transfer learning. (**A**) UMAP embeddings of mapping of adult myeloid cells (54,047 cells) to developmental myeloid reference (218,758 cells) using scArches on scVI model. Points are colored by the dataset of origin. (**B**) UMAP embedding as in A, points are colored by cell population annotation label, for adult cells (left) and developmental cells (right). (**C**) Correspondence between developmental and adult myeloid transcriptional phenotypes estimated by label transfer after mapping with scArches. The dot size is proportional to the fraction of cells in the adult population (*y*-axis) with a given predicted prenatal cell population label (*x*-axis). The dot color denotes the median similarity of the adult cells to prenatal cells in the common embedding. Adult cells where less than 80% of prenatal neighbors have a uniform annotation are labeled as "low confidence".

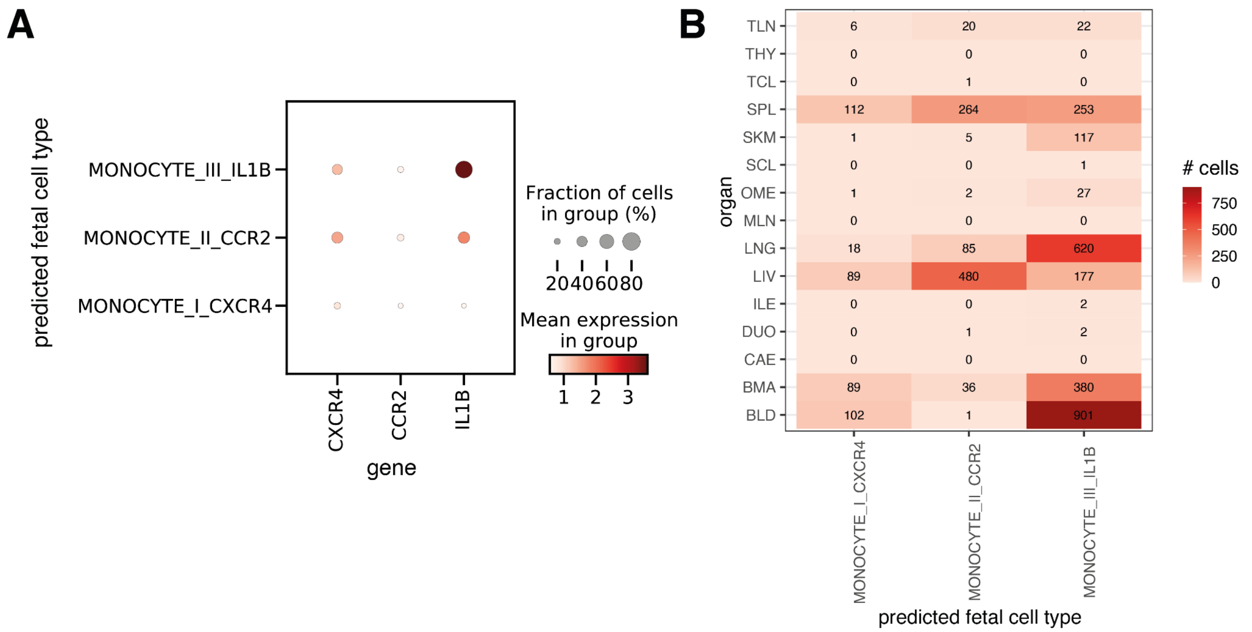

fig. S13: Prenatal-adult comparison in monocytes. (**A**) Dot plot of expression of monocyte subtype markers in adult cells aligned to prenatal monocyte subtypes. (**B**) Heat map of distribution across organs of adult cells aligned to prenatal monocyte subtypes (TLN: thoracic lymph nodes, THY: thymus, TCL: transverse colon, SPL: spleen, SKM: skeletal muscle, SCL: sigmoid colon, OME: omentum, MLN: mesenteric lymph node, LNG: lung, LIV: liver, ILE: ileum, DUO: duodenum, CAE: cecum, BMA: bone marrow, BLD: blood).

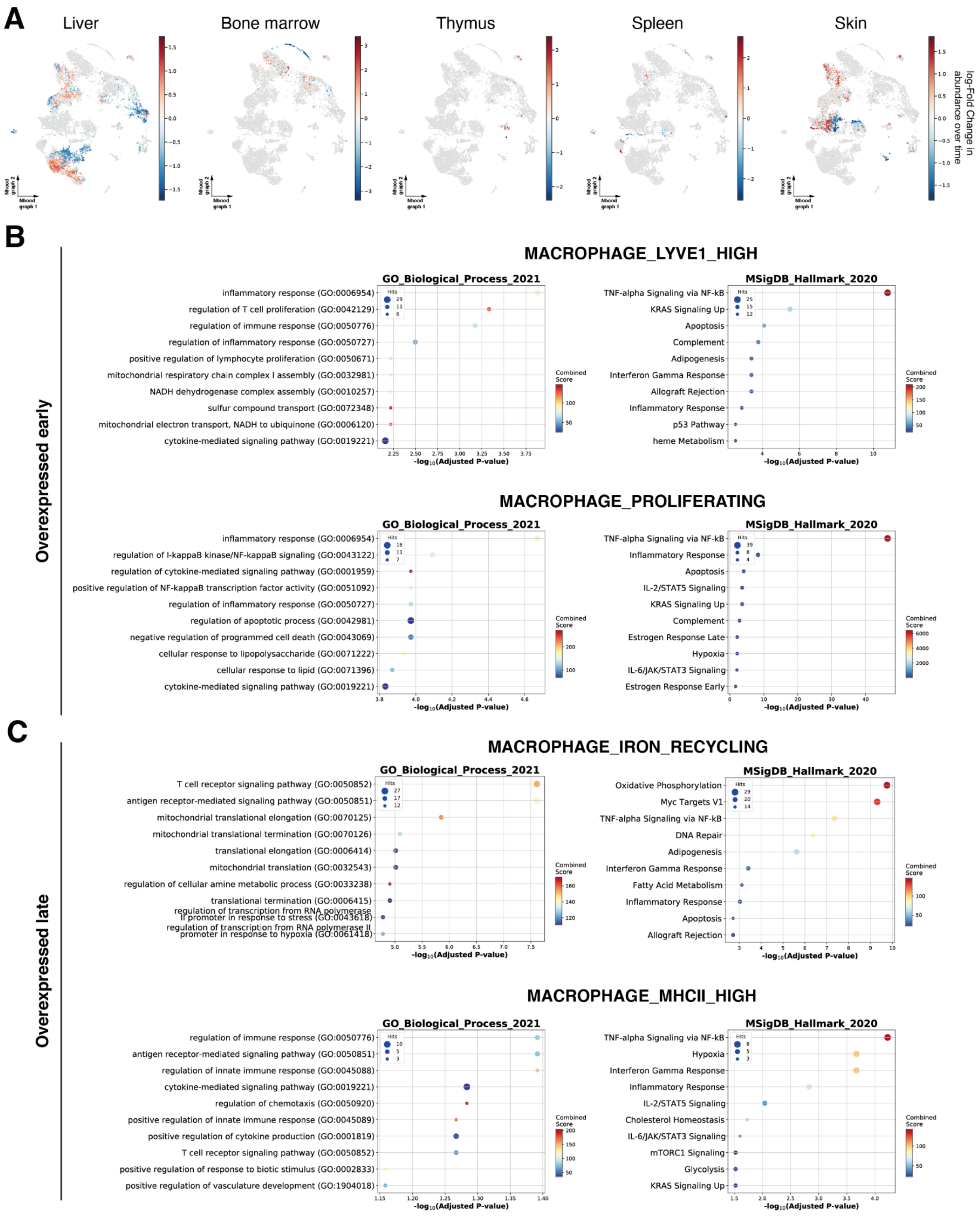
A
Liver
Bone marrow
Thymus
Spleen
Skin
log-Fold Change in abundance over time
Nhood graph 1
Nhood graph 2
B
Overexpressed early
MACROPHAGE_LYVE1_HIGH
GO_Biological_Process_2021
inflammatory response (GO:0006954)
regulation of T cell proliferation (GO:0042129)
regulation of immune response (GO:0050776)
regulation of inflammatory response (GO:0050727)
positive regulation of lymphocyte proliferation (GO:0050671)
mitochondrial respiratory chain complex I assembly (GO:0032981)
NADH dehydrogenase complex assembly (GO:0010257)
sulfur compound transport (GO:0072348)
mitochondrial electron transport, NADH to ubiquinone (GO:0006120)
cytokine-mediated signaling pathway (GO:0019221)
MSigDB_Hallmark_2020
TNF-alpha Signaling via NF-kB
KRAS Signaling Up
Apoptosis
Complement
Adipogenesis
Interferon Gamma Response
Allograft Rejection
Inflammatory Response
p53 Pathway
heme Metabolism
Hits
Combined Score
-log10(Adjusted P-value)
MACROPHAGE_PROLIFERATING
GO_Biological_Process_2021
inflammatory response (GO:0006954)
regulation of I-kappaB kinase/NF-kappaB signaling (GO:0043122)
regulation of cytokine-mediated signaling pathway (GO:0001959)
positive regulation of NF-kappaB transcription factor activity (GO:0051092)
regulation of inflammatory response (GO:0050727)
regulation of apoptotic process (GO:0042981)
negative regulation of programmed cell death (GO:0043069)
cellular response to lipopolysaccharide (GO:0071222)
cellular response to lipid (GO:0071396)
cytokine-mediated signaling pathway (GO:0019221)
MSigDB_Hallmark_2020
TNF-alpha Signaling via NF-kB
Inflammatory Response
Apoptosis
IL-2/STAT5 Signaling
KRAS Signaling Up
Complement
Estrogen Response Late
Hypoxia
IL-6/JAK/STAT3 Signaling
Estrogen Response Early
C
Overexpressed late
MACROPHAGE_IRON_RECYCLING
GO_Biological_Process_2021
T cell receptor signaling pathway (GO:0050852)
antigen receptor-mediated signaling pathway (GO:0050851)
mitochondrial translational elongation (GO:0070125)
mitochondrial translational termination (GO:0070126)
translational elongation (GO:0006414)
mitochondrial translation (GO:0032543)
regulation of cellular amine metabolic process (GO:0033238)
translational termination (GO:0006415)
regulation of transcription from RNA polymerase II promoter in response to stress (GO:0043618)
regulation of transcription from RNA polymerase II promoter in response to hypoxia (GO:0061418)
MSigDB_Hallmark_2020
Oxidative Phosphorylation
Myc Targets V1
TNF-alpha Signaling via NF-kB
DNA Repair
Adipogenesis
Interferon Gamma Response
Fatty Acid Metabolism
Inflammatory Response
Apoptosis
Allograft Rejection
MACROPHAGE_MHCII_HIGH
GO_Biological_Process_2021
regulation of immune response (GO:0050776)
antigen receptor-mediated signaling pathway (GO:0050851)
regulation of innate immune response (GO:0045088)
cytokine-mediated signaling pathway (GO:0019221)
regulation of chemotaxis (GO:0050920)
positive regulation of innate immune response (GO:0045089)
positive regulation of cytokine production (GO:0001819)
T cell receptor signaling pathway (GO:0050852)
positive regulation of response to biotic stimulus (GO:0002833)
positive regulation of vasculature development (GO:1904018)
MSigDB_Hallmark_2020
TNF-alpha Signaling via NF-kB
Hypoxia
Interferon Gamma Response
Inflammatory Response
IL-2/STAT5 Signaling
Cholesterol Homeostasis
IL-6/JAK/STAT3 Signaling
mTORC1 Signaling
Glycolysis
KRAS Signaling Up

fig. S14: Differential abundance across gestation in myeloid cell populations. (**A**) Milo neighborhood embedding of myeloid cells showing differential abundance across gestation. Each point represents a neighborhood, the layout of points is determined by the position of the neighborhood index cell in the UMAP in fig. S4G, the size of points is proportional to the number of cells in the neighborhood. Neighborhoods are colored by their log-fold change (logFC) in abundance over time, where logFC>0 indicates significant enrichment in early cells and logFC > 0 indicates significant enrichment in late cells. Only neighborhoods showing significant differential abundance (SpatialFDR<10%) are colored. (**B** and **C**) Gene set enrichment analysis results for differentially expressed genes in gestation stage-specific neighborhoods of macrophages. Each plot shows the top 10 significant hits for the gene list. The *x*-axis shows the negative $\log_{10}$ of the *P*-value adjusted for multiple testing (Benjamini–Hochberg correction). The size of the dots is proportional to the number of genes associated with the gene set. The color represents the combined enrichr score calculated with gseapy. Results using the Gene Ontology Biological Process and the MSigDB Hallmark 2020 databases are shown. (B) Gene set enrichment analysis for genes overexpressed in early-specific neighborhoods of LYVE1^{hi} macrophages and proliferating macrophages. (C) Gene set enrichment analysis for genes overexpressed in late-specific neighborhoods of iron-recycling macrophages and MHCII^{hi} macrophages.

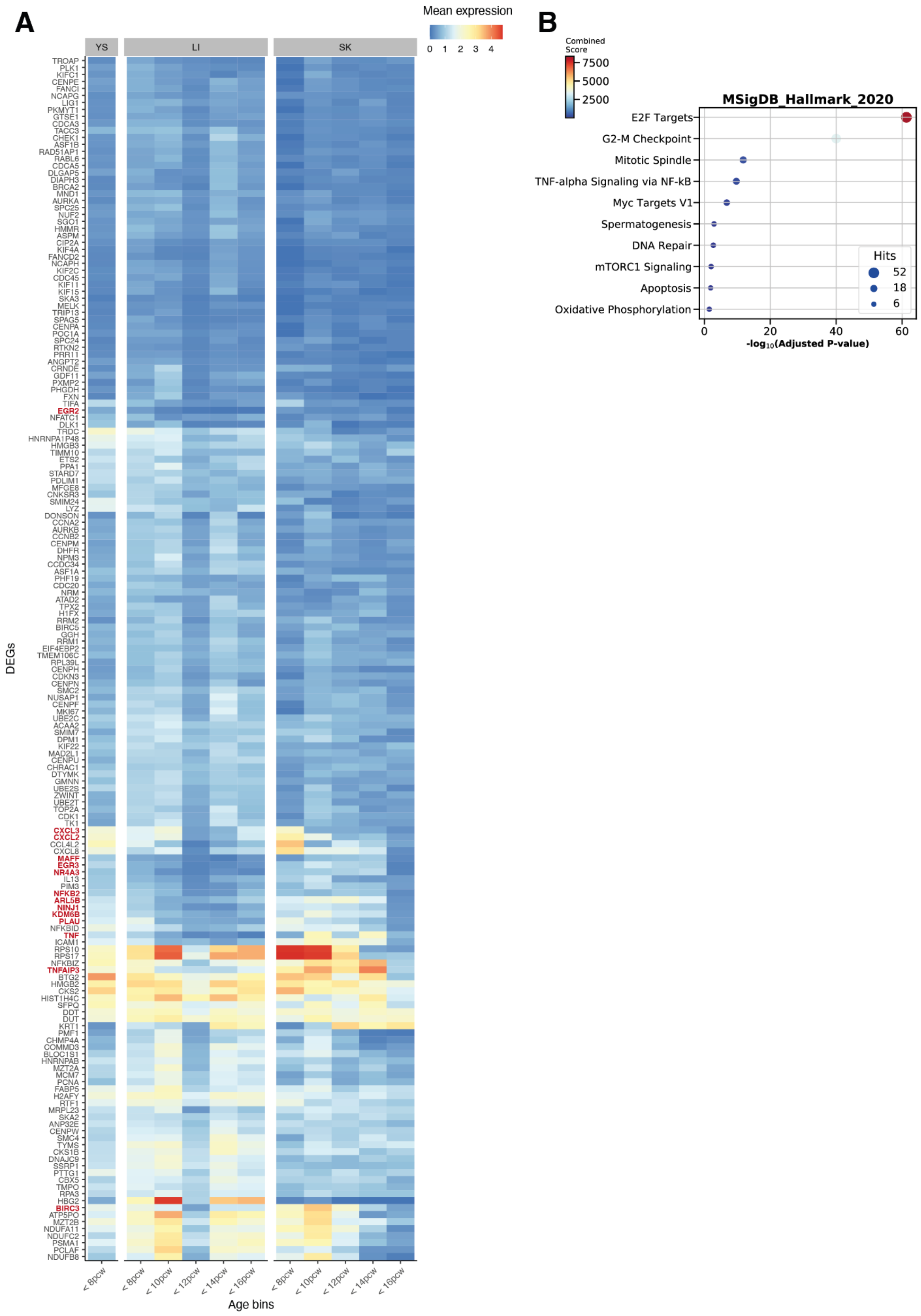
A
Mean expression
0 1 2 3 4
YS
LI
SK
DEGs
TROAP
PLK1
KIFC1
CENPE
FANCI
NCAPG
LIG1
PKMYT1
GTSE1
CDCA3
TACC3
CHEK1
ASF1B
RAD51AP1
RABL6
CDCA5
DLGAP5
DIAPH3
BRCA2
MND1
AURKA
SPC25
NUF2
SGO1
HMMR
ASPM
CIP2A
KIF4A
FANCD2
NCAPH
KIF2C
CDC45
KIF11
KIF15
SKA3
MELK
TRIP13
SPAG5
CENPA
POC1A
SPC24
RTKN2
PRR11
ANGPT2
CRNDE
GDF11
PXMP2
PHGDH
FXN
TIFA
EGR2
NFATC1
DLK1
TRDC
HNRNPA1P48
HMGB3
TIMM10
ETS2
PPA1
STARD7
PDLIM1
MFGE8
CNKSR3
SMIM24
LYZ
DONSON
CCNA2
AURKB
CCNB2
CENPM
DHFR
NPM3
CCDC34
ASF1A
PHF19
CDC20
NRM
ATAD2
TPX2
H1FX
RRM2
BIRC5
GGH
RRM1
EIF4EBP2
TMEM106C
RPL39L
CENPH
CDKN3
CENPN
SMC2
NUSAP1
CENPF
MKI67
UBE2C
ACAA2
SMIM7
DPM1
KIF22
MAD2L1
CENPU
CHRAC1
DTYMK
GMNN
UBE2S
ZWINT
UBE2T
TOP2A
CDK1
TK1
CXCL3
CXCL2
CCL4L2
CXCL8
MAFF
EGR3
NR4A3
IL13
PIM3
NFKB2
ARL5B
NINJ1
KDM6B
PLAU
NFKBID
TNF
ICAM1
RPS10
RPS17
NFKBIZ
TNFAIP3
BTG2
HMGB2
CKS2
HIST1H4C
SFPQ
DDT
DUT
KRT1
PMF1
CHMP4A
COMMD3
BLOC1S1
HNRNPAB
MZT2A
MCM7
PCNA
FABP5
H2AFY
RTF1
MRPL23
SKA2
ANP32E
CENPW
SMC4
TYMS
CKS1B
DNAJC9
SSRP1
PTTG1
CBX5
TMPO
RPA3
HBG2
BIRC3
ATP5PO
MZT2B
NDUFA11
NDUFC2
PSMA1
PCLAF
NDUFB8
< 8pcw
< 8pcw
< 10pcw
< 12pcw
< 14pcw
< 16pcw
< 8pcw
< 10pcw
< 12pcw
< 14pcw
< 16pcw
Age bins
B
Combined Score
7500
5000
2500
MSigDB_Hallmark_2020
E2F Targets
G2-M Checkpoint
Mitotic Spindle
TNF-alpha Signaling via NF-kB
Myc Targets V1
Spermatogenesis
DNA Repair
mTORC1 Signaling
Apoptosis
Oxidative Phosphorylation
Hits
52
18
6
0
20
40
60
-log10(Adjusted P-value)

fig. S15: Differential expression analysis on early-specific neighborhoods of mast cells. (**A**) Average expression by time point of 185 genes overexpressed in early-specific neighborhoods of mast cells. Genes associated with TNF signaling via NF-κB are highlighted in red. (**B**) Gene set enrichment analysis results using the MSigDB Hallmark 2020 database. The *x*-axis shows the negative $\log_{10}$ of the *P*-value adjusted for multiple testing (Benjamini–Hochberg correction). The size of the dots is proportional to the number of genes associated with the gene set. The color represents the combined enrichr score calculated with gseapy.

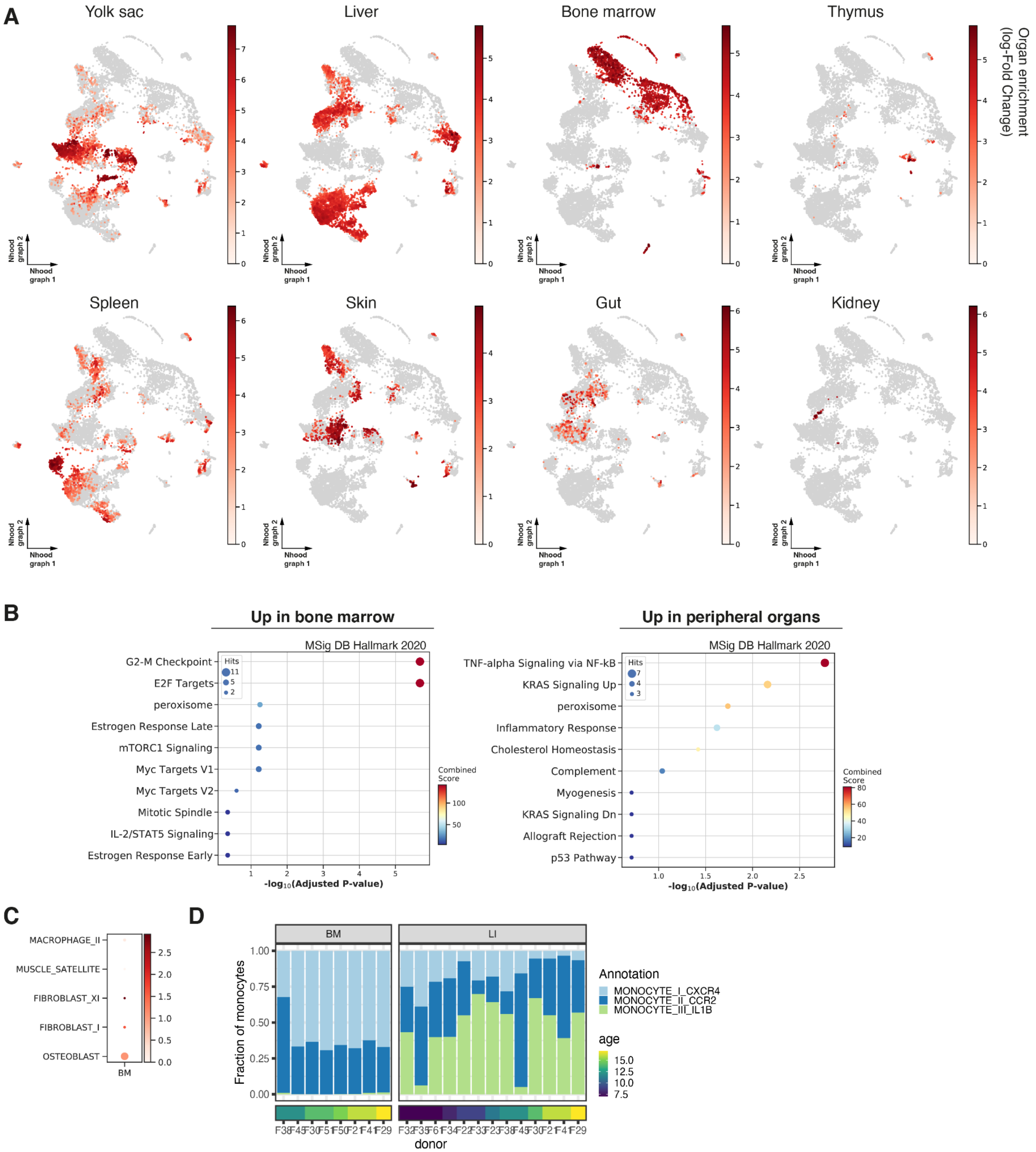

fig. S16: Differential abundance across organs in myeloid cell populations. (**A**) Milo neighborhood embedding of myeloid cells. Each point represents a neighborhood, the layout of points is determined by the position of the neighborhood index cell in the UMAP in fig. S4G, the size of points is proportional to the number of cells in the neighborhood. Neighborhoods are colored by their log-fold change in abundance between the specified organ and all other organs. Only neighborhoods showing significant enrichment (SpatialFDR<10% and logFC≥2) are colored. (**B**) Dot plot of enrichment analysis results on genes upregulated (left) and downregulated (right) in bone marrow CCR2^{hi} monocytes compared to other organs. The *x*-axis represents the negative $\log_{10}$ of *P*-values adjusted for multiple testing (Benjamini–Hochberg correction) The *y*-axis shows the top 10 enriched gene sets (using the MSigDB Hallmark 2020 database). The size of the dots is proportional to the number of genes associated with the

gene set. The color represents the combined enrichr score calculated with gseapy. (**C**) Dot plot of *CXCL12* expression in cell populations in the bone marrow. The color represents the average expression level (normalized and log-transformed counts) and the size represents the cell count of each cell type within bone marrow. Only cell populations with average expression > 1, and cell count > 10 are shown. (**D**) Fraction of abundance of monocyte subsets for each donor in liver (LI) and bone marrow (BM). Donors are ordered by gestational age.

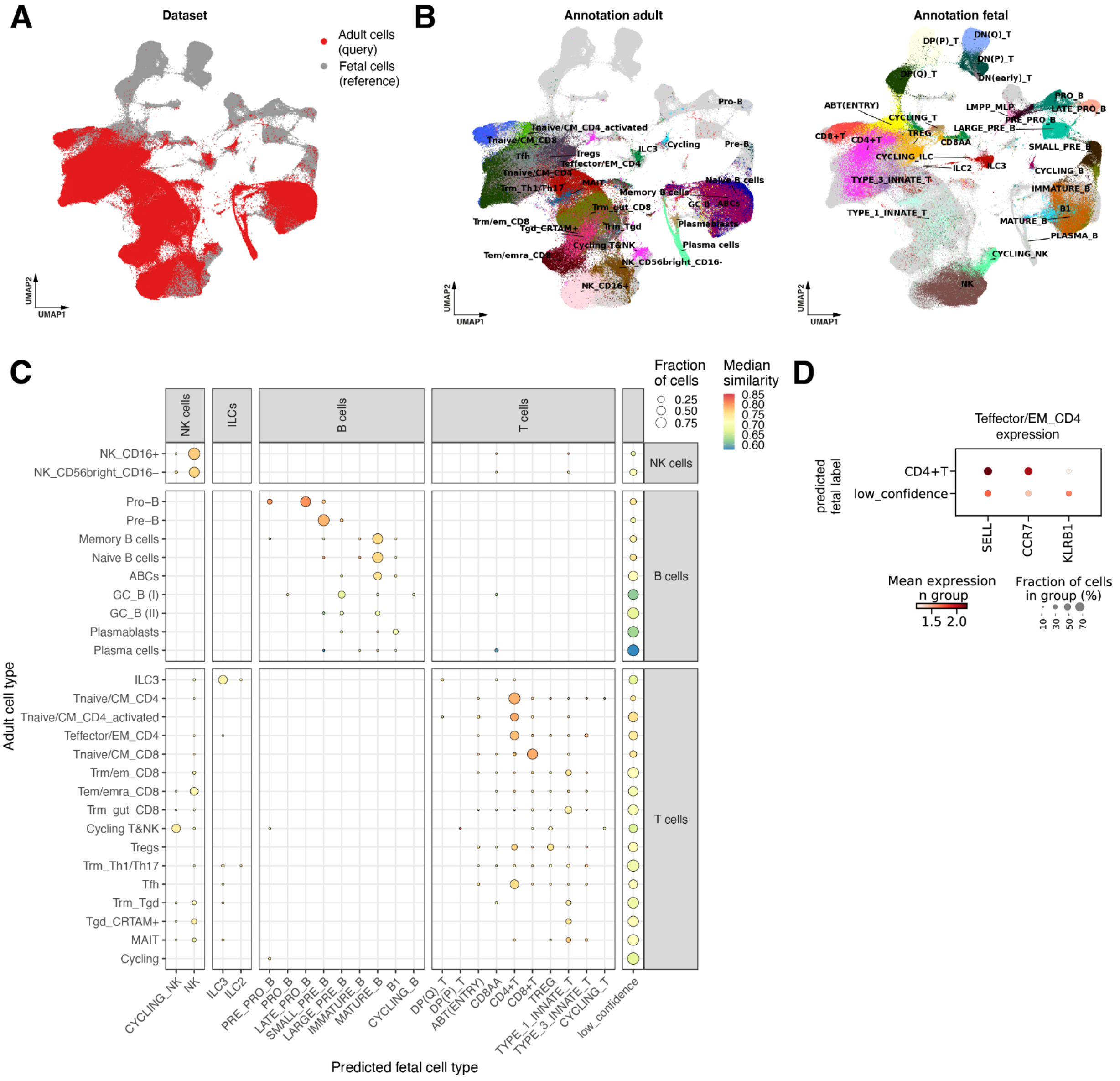

fig. S17: Mapping of adult lymphoid cells to prenatal reference with transfer learning. (**A**) UMAP embeddings of mapping of adult lymphoid cells (264,929) to prenatal lymphoid reference (218,758 cells) using scArches on scVI model. Points are colored by the dataset of origin. (**B**) UMAP embedding as in A, points are colored by cell population annotation label, for adult cells (left) and prenatal cells (right). (**C**) Correspondence between prenatal and adult myeloid transcriptional phenotypes estimated by label transfer after mapping with scArches. The dot size is proportional to the fraction of cells in the adult population (y-axis) with a given predicted prenatal cell population label (x-axis). The dot color denotes the median similarity of the adult cells to prenatal cells in the common embedding. Adult cells where less than 80% of prenatal neighbors have a uniform annotation are labeled as "low confidence". (**D**) The fraction of adult $CD4^+$ effector T cells that were matched to fetal $CD4^+$ T cells show increased expression of naive markers (SELL, CCR7) and $CD4^+$T effector markers (KLRB1) compared to effector cells not having a developmental equivalent (low confidence). This indicates that the matching might be driven by additional heterogeneity in this adult cell compartment, instead of the true correspondent of adult memory T cells within fetal cells.

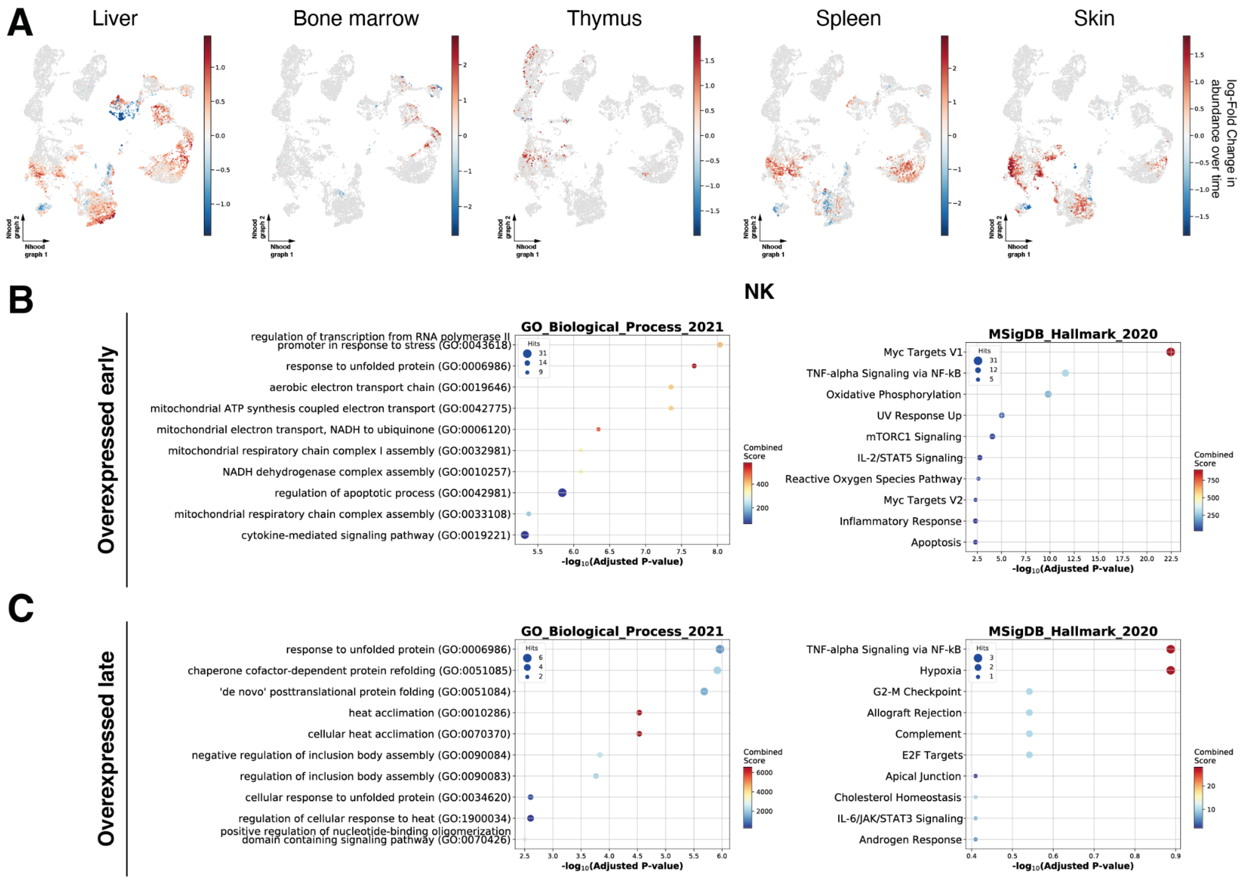

fig. S18: Differential abundance across gestation in lymphoid cell populations. (**A**) Milo neighborhood embedding of lymphoid cells showing differential abundance across gestation. Each point represents a neighborhood, the layout of points is determined by the position of the neighborhood index cell in the UMAP in fig. S4I, the size of points is proportional to the number of cells in the neighborhood. Neighborhoods are colored by their log-fold change (logFC) in abundance over time, where logFC > 0 indicates significant enrichment in early cells and logFC > 0 indicates significant enrichment in late cells. Only neighborhoods showing significant differential abundance (SpatialFDR < 10%) are colored. (**B-C**) Gene set enrichment analysis results for differentially expressed genes in early-specific neighborhoods (B) and late specific neighborhoods (C) of NK cells. Each plot shows the top 10 significant hits for the gene set. The x-axis shows the negative $\log_{10}$ of the P-value adjusted for multiple testing (Benjamini–Hochberg correction). The size of the dots is proportional to the number of genes associated with the gene set. The color represents the combined enrichr score calculated with gseapy. Results using the Gene Ontology Biological Process and the MSigDB Hallmark 2020 databases are shown.

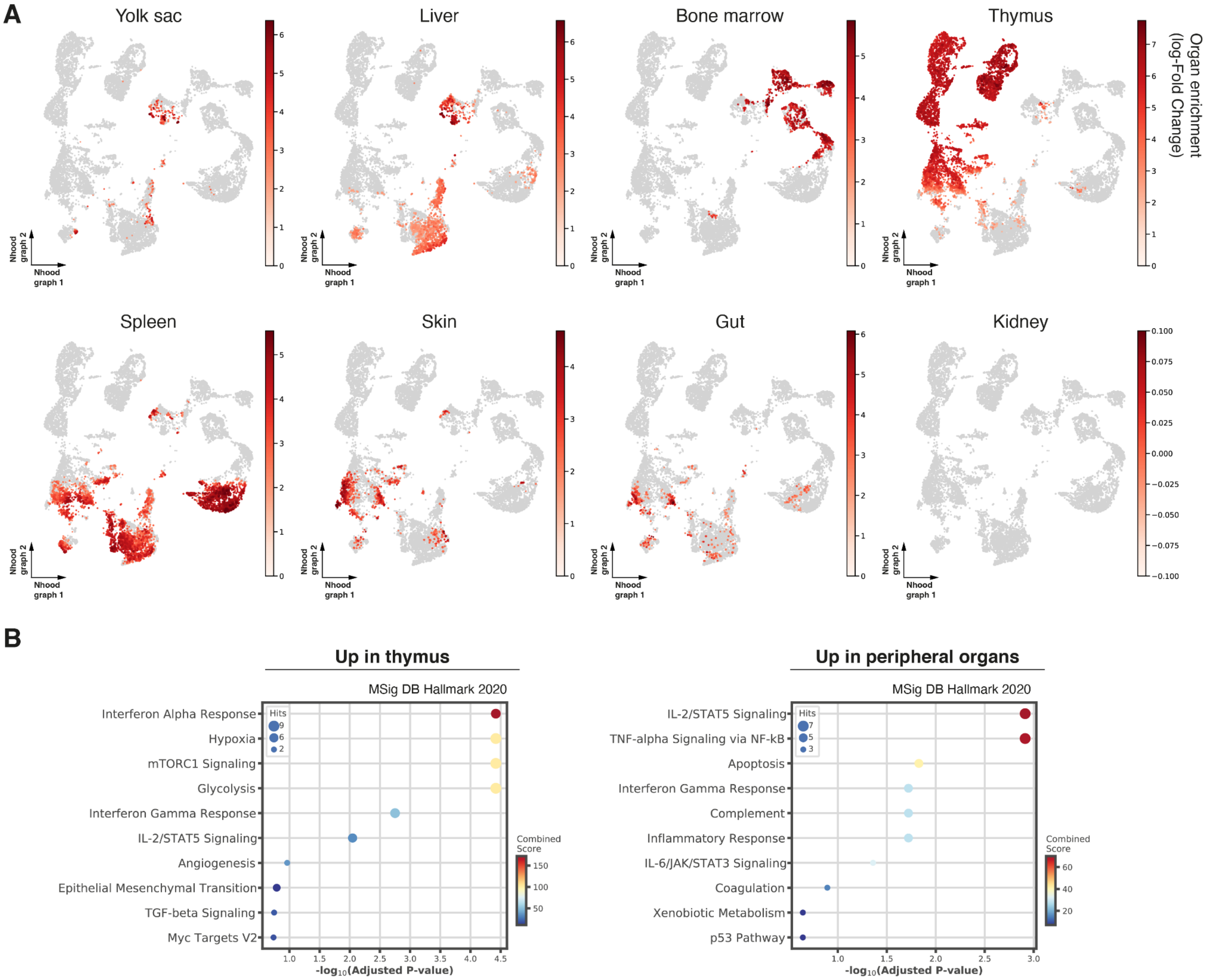

fig. S19: Differential abundance across organs in lymphoid compartment. (**A**) Milo neighborhood embedding of lymphoid cells showing differential abundance between organs. Each point represents a neighborhood, the layout of points is determined by the position of the neighborhood index cell in the UMAP in fig. S4I, the size of points is proportional to the number of cells in the neighborhood. Neighborhoods are colored by their log-fold change in abundance between the specified organ and all other organs. Only neighborhoods showing significant enrichment (SpatialFDR < 10% and log-fold change > 2) are colored. (**B**) Dot plot of enrichment analysis results on genes upregulated (left) and downregulated (right) in thymic mature T cells compared to other organs. The x-axis represents the negative $\log_{10}$ of P-values adjusted for multiple testing (Benjamini–Hochberg correction) The y-axis shows the top 10 enriched gene sets (using the MSigDB Hallmark 2020 database). The size of the dots is proportional to the number of genes associated with the gene set. The color represents the combined enrichr score calculated with gseapy.

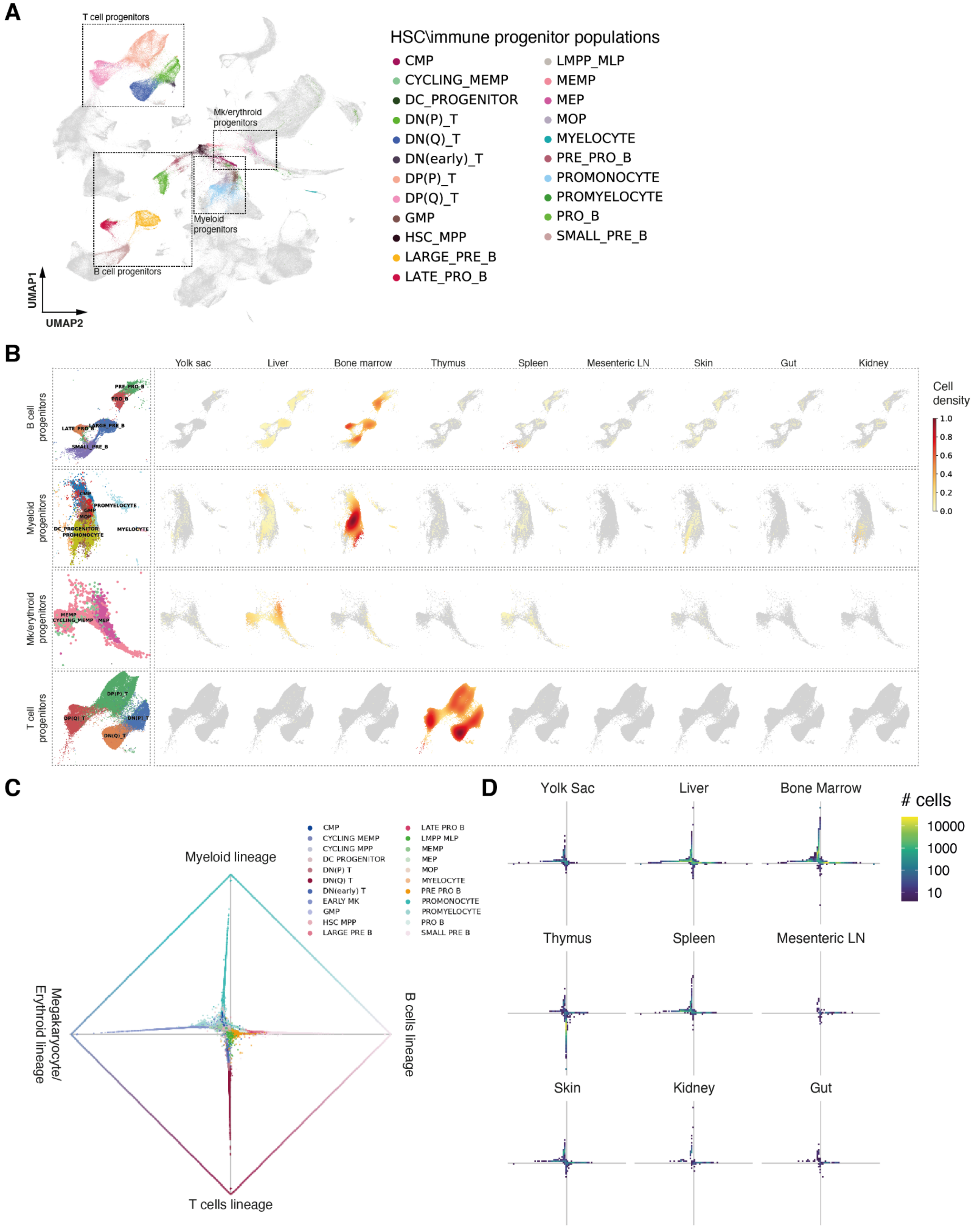
A
T cell progenitors
Mk/erythroid progenitors
Myeloid progenitors
B cell progenitors
UMAP1
UMAP2
HSC\immune progenitor populations
CMP
CYCLING_MEMP
DC_PROGENITOR
DN(P)_T
DN(Q)_T
DN(early)_T
DP(P)_T
DP(Q)_T
GMP
HSC_MPP
LARGE_PRE_B
LATE_PRO_B
LMPP_MLP
MEMP
MEP
MOP
MYELOCYTE
PRE_PRO_B
PROMONOCYTE
PROMYELOCYTE
PRO_B
SMALL_PRE_B
B
Yolk sac
Liver
Bone marrow
Thymus
Spleen
Mesenteric LN
Skin
Gut
Kidney
Cell density
1.0
0.8
0.6
0.4
0.2
0.0
B cell progenitors
Myeloid progenitors
Mk/erythroid progenitors
T cell progenitors
C
Myeloid lineage
Megakaryocyte/ Erythroid lineage
B cells lineage
T cells lineage
CMP
CYCLING MEMP
CYCLING MPP
DC PROGENITOR
DN(P) T
DN(Q) T
DN(early) T
EARLY MK
GMP
HSC MPP
LARGE PRE B
LATE PRO B
LMPP MLP
MEMP
MEP
MOP
MYELOCYTE
PRE PRO B
PROMONOCYTE
PROMYELOCYTE
PRO B
SMALL PRE B
D
Yolk Sac
Liver
Bone Marrow
Thymus
Spleen
Mesenteric LN
Skin
Kidney
Gut
cells
10000
1000
100
10

fig. S20. Full spectrum of hematopoietic progenitors in peripheral organs. (**A**) UMAP embedding of all immune and blood cells, highlighting the progenitor cell populations. Dashed boxes highlight lineage populations shown in B. (**B**) Density plot of cells from each organ on a subset of UMAP embedding for different hematopoietic lineages. Density is calculated over all immune cells within each organ. (**C**) Simplex projection of cells in progenitor populations according to fate probabilities for each immune and blood cell lineage. Each cell is placed inside the simplex according to its probability of reaching any of the terminal states. Cells in the center have higher multilineage potential, whereas cells closer to one of the corners are more committed. (**D**) Binned density of cells for each organ over the fate simplex shown in C, where the color of the 2D bin represents the number of cells from the organ in that position in the simplex.

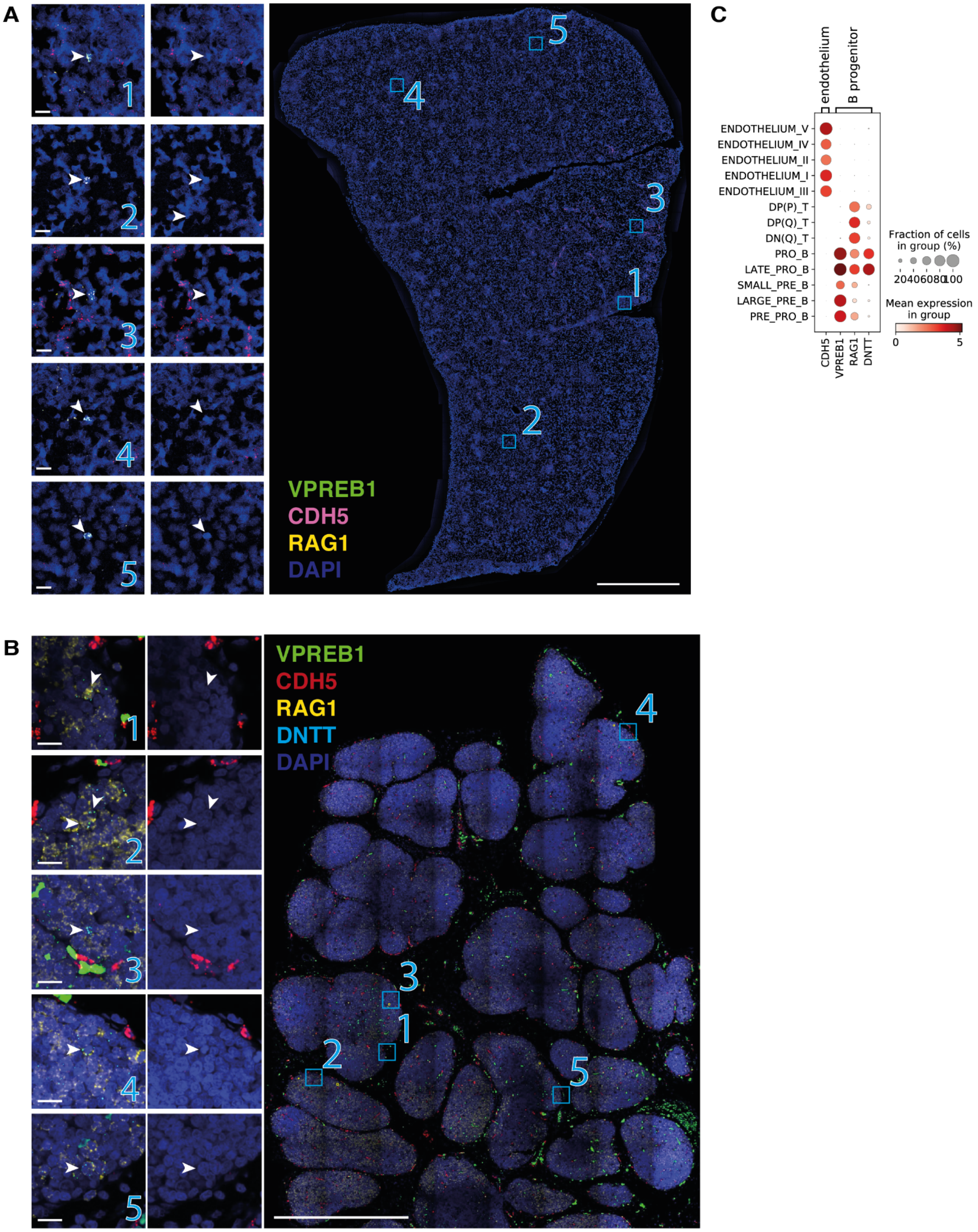

fig. S21. Multiplex smFISH validation of B cell progenitors in peripheral tissues. Multiplex smFISH staining of DAPI, *CDH5* for endothelial cells, and *VPREB1*, *RAG1* with/without *DNTT* for B cell progenitors in the human (**A**) prenatal spleen at 14 pcw and (**B**) prenatal thymus at 16 pcw. Left: cells highlighted from corresponding regions in the right panel overview matched by numbers (scale bar: 20 µm). Middle: *CDH5* channel alone depicting endothelial cells. Right: full section view with the areas of interest boxed (scale bar: 1 mm). White arrows point to B cell progenitors identified. (**C**) Dot plot showing log normalized expressions of *CDH5*, *VPREB1*, *RAG1*, and *DNTT* in the corresponding cell populations. Only cell types with log normalized expression at least 2 in at least one of the four genes are shown here.

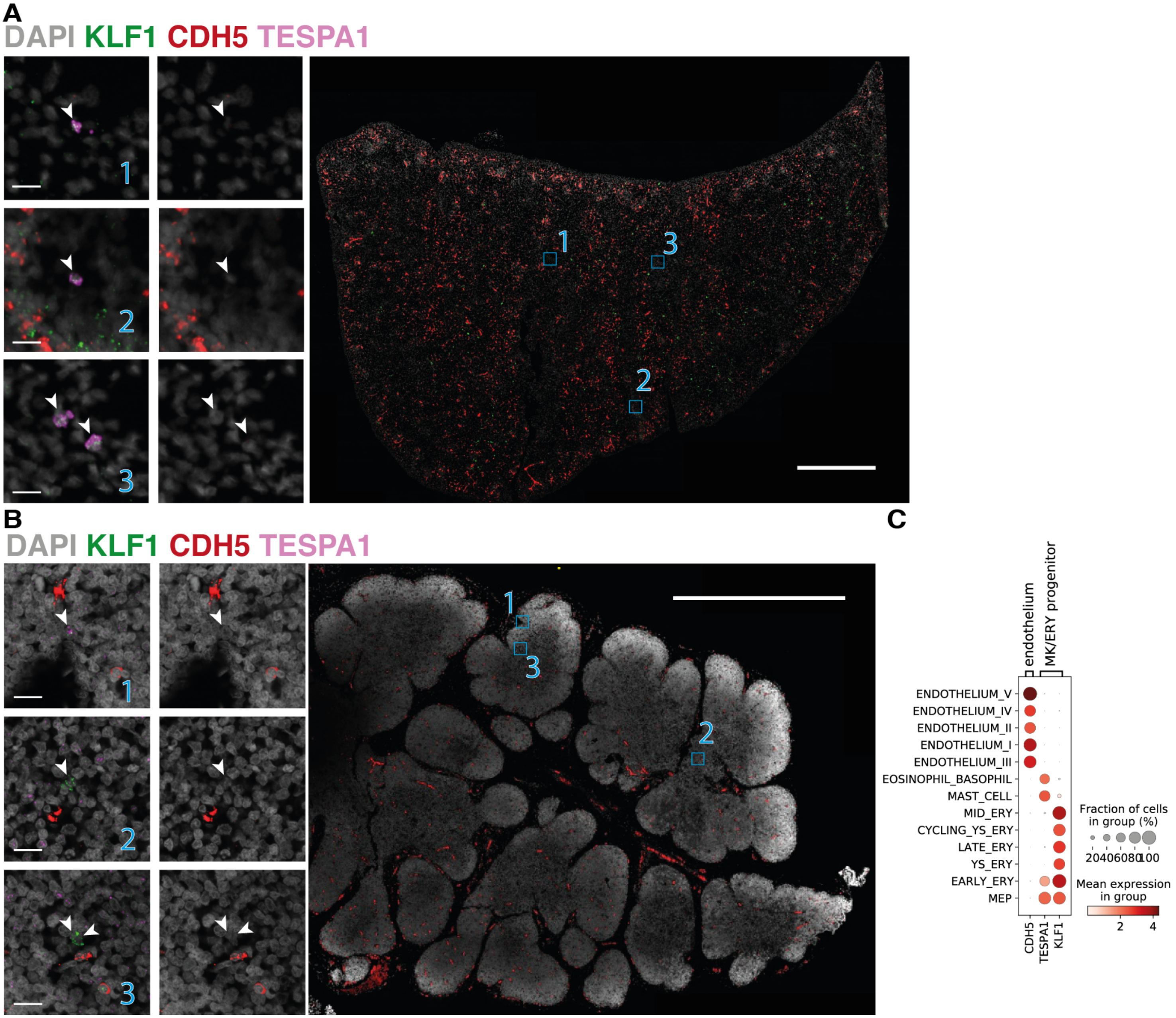

fig. S22. Multiplex smFISH validation of megakaryocyte/erythroid progenitors in peripheral tissues. Multiplex smFISH staining of DAPI, *CDH5* for endothelial cells, and *KLF1*, *TESPA1* for megakaryocyte/erythroid progenitors in the human (**A**) prenatal spleen at 14 pcw and (**B**) prenatal thymus at 16 pcw. Left: cells highlighted from corresponding regions in the right panel overview matched by numbers (scale bar: 20 µm). Middle: *CDH5* channel alone depicting endothelial cells. Right: full section view with the areas of interest boxed (scale bar: 1 mm). White arrows point to megakaryocyte/erythroid progenitors identified. (**C**) Dot plot showing log normalized expressions of *CDH5*, *KLF1*, and *TESPA1* in the corresponding cell populations. Only cell types with log normalized expression at least 2 in at least one of the three genes are shown here.

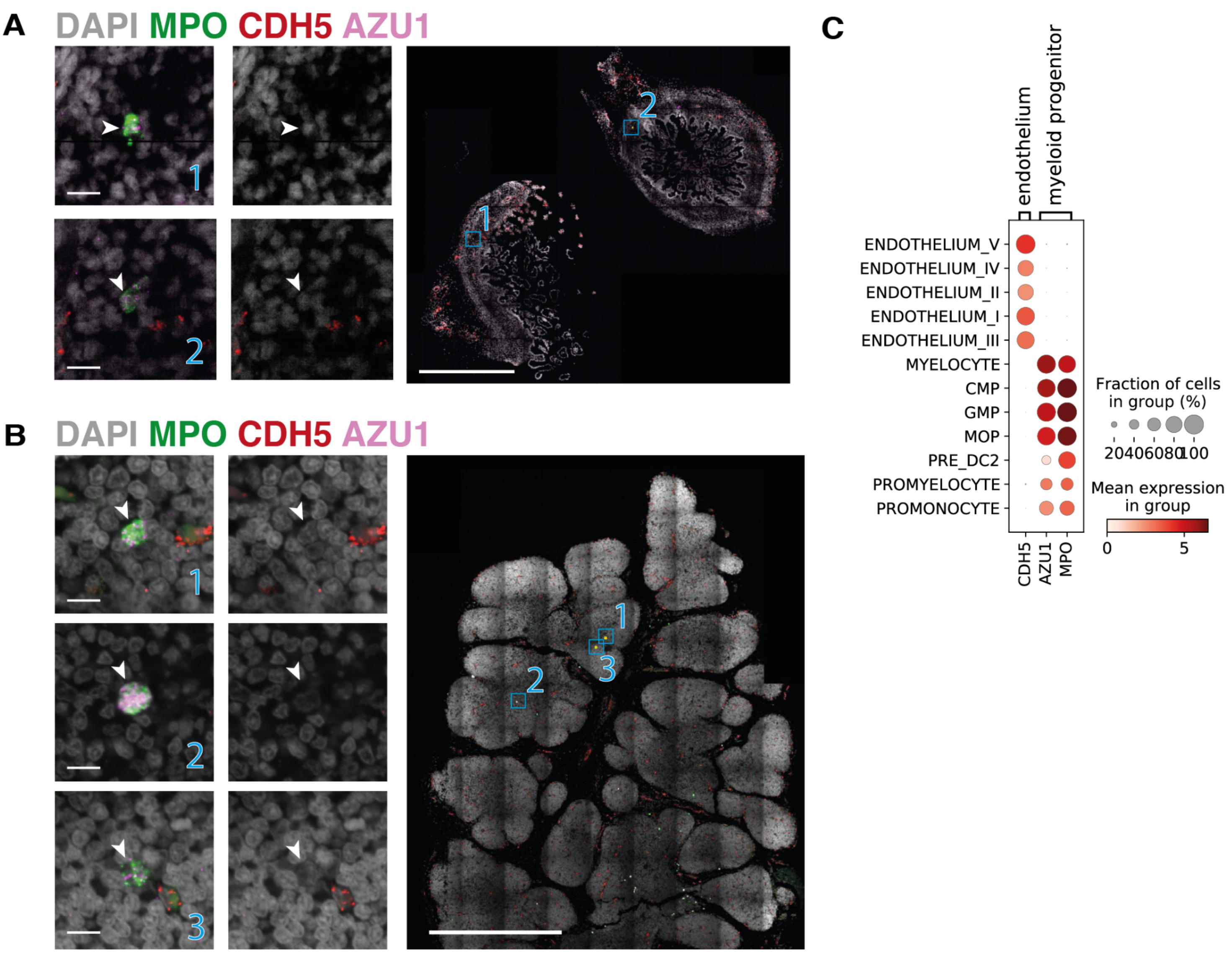

fig. S23. Multiplex smFISH validation of myeloid progenitors in peripheral tissues. Multiplex smFISH staining of DAPI, *CDH5* for endothelial cells, and *MPO* and *AZU1* for myeloid progenitors in the human (**A**) prenatal intestine at 15 pcw and (**B**) prenatal thymus at 16 pcw. Left: cells highlighted from corresponding regions in the right panel overview matched by numbers (scale bar: 20 µm in (**A**) and 10 µm in (**B**)). Middle: *CDH5* channel alone depicting endothelial cells. Right: full section view with the areas of interest boxed (scale bar: 1 mm). White arrows point to myeloid progenitors identified. (**C**) Dot plot showing log normalized expressions of *CDH5*, *MPO*, and *AZU1* in the corresponding cell populations. Only cell types with log normalized expression at least 2 in at least one of the three genes are shown here.

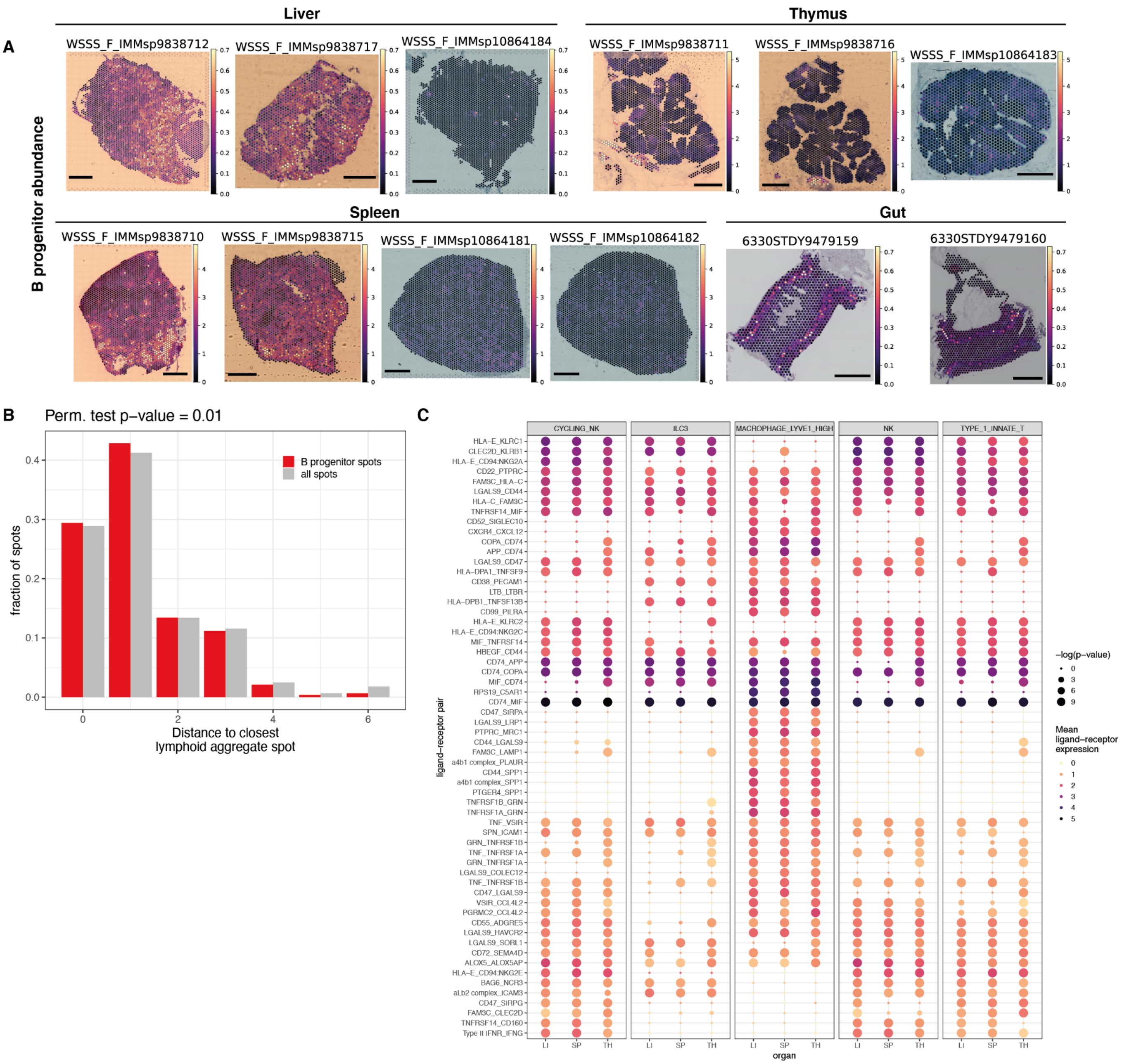

fig. S24. System-wide B lymphopoiesis. (**A**) Sum of abundances of B progenitor cell populations in spatial transcriptomics slides estimated with cell2location (scale bar: 1 mm). (**B**) Distribution of distance to the closest spot assigned as splenic lymphoid aggregate microenvironment from spots containing B cell progenitors (red) and from all analyzed spots (gray). Distance is measured as Euclidean distance of spots in spatial coordinates. We test if the distance to lymphoid aggregates is significantly smaller in B cell progenitor spots compared to other spots with a permutation test (5000 samples). Spots were assigned to the lymphoid aggregate microenvironment if the NMF factor value for the microenvironment (Fig. 1D, "B cell zone") was above the 95% quantile for that slide. We considered spots to contain B cell progenitors if the sum of abundances of the B cell progenitors was above the 95% quantile for that slide. (**C**) Predicted cell–cell interactions between B cell progenitors and colocalizing cell types (ILC3, LYVE1^{hi} macrophage, NK cells, cycling NK cells and type 1 innate T cells, excluding LMPP_MLP as they are likely to be upstream progenitors of B lineage progenitors) from CellPhoneDB across liver (LI), spleen (SP) and thymus (TH). The first gene in each ligand-receptor pair is expressed in B cell progenitors and the second in the

interacting cell type. The color represents the average expression values of the ligand and receptor within their corresponding cell types, and the size represents −log(*P*-value). In addition to the previously described CXCL12–CXCR4 interaction in murine studies (*102*, *103*), our analysis identified many additional novel interactions that may inform efforts to generate and engineer B cells in vitro.

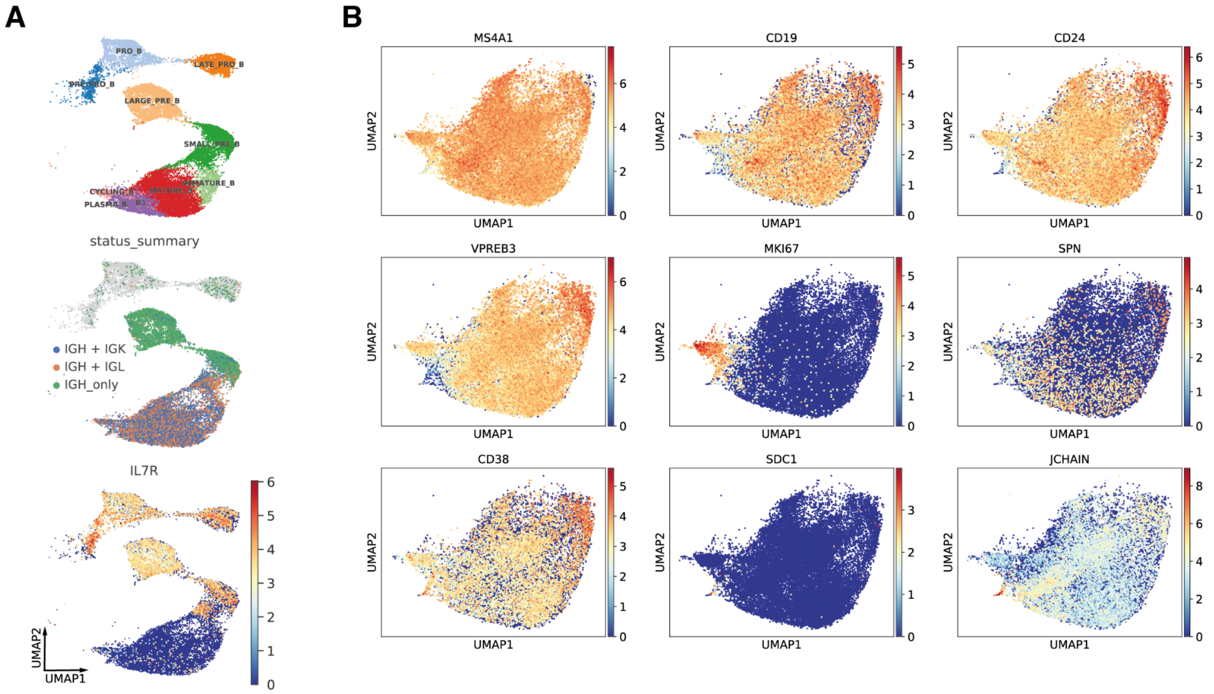

fig. S25. Characterization of B cells. (**A**) Close-up view of B cell populations on the UMAP embedding of all lymphoid cells (as shown in fig. S4I), colored by annotated cell population identity (top), status summary of cells expressing productive heavy (IGH) and/or light chains (IGK or IGL) of BCR from single-cell BCR sequencing (middle), and colored by *IL7R* expression pattern (bottom). (**B**) Marker gene expression patterns overlaid onto the same UMAP plot in Fig. 5A. Immature B cells were characterized by higher expression of *CD19*, *CD24*, and *VPREB3*. Mature B, cycling B, plasma B, and putative B1 cells expressed *MS4A1* except plasma B (*MS4A1*^{lo} and expressing *CD38*, *SDC1*, and *JCHAIN*). Cycling B cells were additionally marked with *MKI67*.

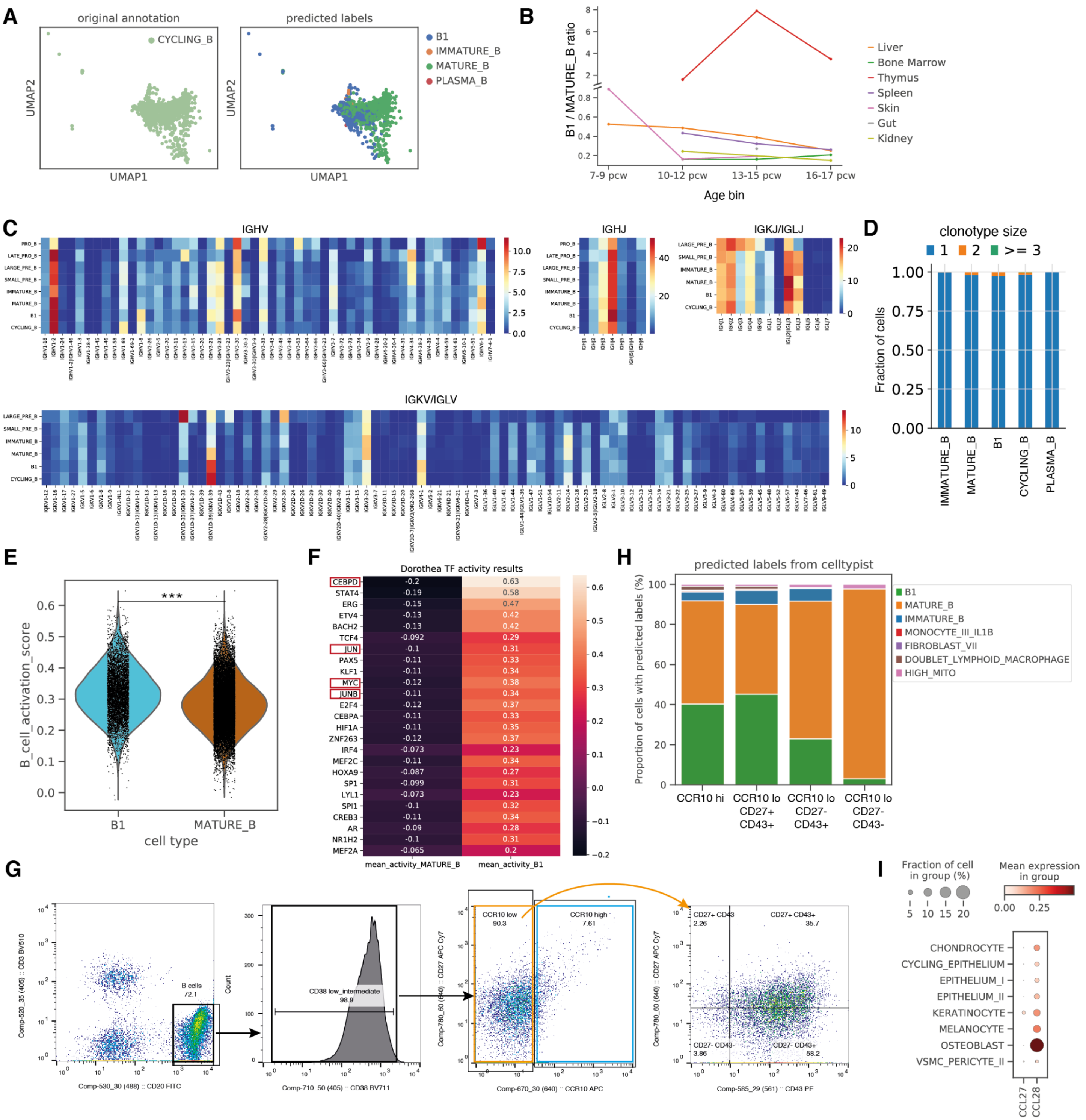
A
original annotation
CYCLING_B
UMAP2
UMAP1
predicted labels
B1
IMMATURE_B
MATURE_B
PLASMA_B
B
B1 / MATURE_B ratio
Age bin
7-9 pcw
10-12 pcw
13-15 pcw
16-17 pcw
Liver
Bone Marrow
Thymus
Spleen
Skin
Gut
Kidney
C
IGHV
IGHJ
IGKJ/IGLJ
IGKV/IGLV
PRO_B
LATE_PRO_B
LARGE_PRE_B
SMALL_PRE_B
IMMATURE_B
MATURE_B
B1
CYCLING_B
D
clonotype size
1
2
>= 3
Fraction of cells
IMMATURE_B
MATURE_B
B1
CYCLING_B
PLASMA_B
E
B_cell_activation_score
***
B1
MATURE_B
cell type
F
Dorothea TF activity results
CEBPD -0.2 0.63
STAT4 -0.19 0.58
ERG -0.15 0.47
ETV4 -0.13 0.42
BACH2 -0.13 0.42
TCF4 -0.092 0.29
JUN -0.1 0.31
PAX5 -0.11 0.33
KLF1 -0.11 0.34
MYC -0.12 0.38
JUNB -0.11 0.34
E2F4 -0.12 0.37
CEBPA -0.11 0.33
HIF1A -0.11 0.35
ZNF263 -0.12 0.37
IRF4 -0.073 0.23
MEF2C -0.11 0.34
HOXA9 -0.087 0.27
SP1 -0.099 0.31
LYL1 -0.073 0.23
SPI1 -0.1 0.32
CREB3 -0.11 0.34
AR -0.09 0.28
NR1H2 -0.1 0.31
MEF2A -0.065 0.2
mean_activity_MATURE_B
mean_activity_B1
H
predicted labels from celltypist
Proportion of cells with predicted labels (%)
B1
MATURE_B
IMMATURE_B
MONOCYTE_III_IL1B
FIBROBLAST_VII
DOUBLET_LYMPHOID_MACROPHAGE
HIGH_MITO
CCR10 hi
CCR10 lo CD27+ CD43+
CCR10 lo CD27- CD43+
CCR10 lo CD27- CD43-
G
B cells 72.1
CD38 low_intermediate 98.9
CCR10 low 90.3
CCR10 high 7.61
CD27+ CD43- 2.26
CD27+ CD43+ 35.7
CD27- CD43- 3.86
CD27- CD43+ 58.2
Comp-530_30 (488) :: CD20 FITC
Comp-710_50 (405) :: CD38 BV711
Comp-670_30 (640) :: CCR10 APC
Comp-585_29 (561) :: CD43 PE
I
Fraction of cell in group (%)
Mean expression in group
CHONDROCYTE
CYCLING_EPITHELIUM
EPITHELIUM_I
EPITHELIUM_II
KERATINOCYTE
MELANOCYTE
OSTEOBLAST
VSMC_PERICYTE_II
CCL27
CCL28

fig. S26. Characterization of putative B1 cells. (**A**) Close-up view of cycling B cell population on UMAP embedding of all lymphoid cells (as shown in fig. S4I), colored by original cell population annotation (left) and annotations predicted by logistic regression trained on all non-progenitor B cell subsets (right). This assigns specific cell identity to each of the cells within the cycling B cell group. The results are used in Fig. 5B. (**B**) Ratio of B1 cell number over mature B cell number in different organs across different gestational age bins. (**C**) Heat map showing the percentage of each BCR heavy (IGHV, IGHJ) and light chain (IGKV/IGLV, IGKJ/IGLJ) V and J gene segments present in different B cell subtypes. (**D**) Barplot of cell fractions with different clonotype size across different mature B cell subtypes. (**E**) Violin plot of B cell activation scores in B1 and mature B cells. Cells were scored according to expression values of all genes in the Gene Ontology B Cell Activation gene list. A significant difference in B cell activation scores was observed between B1 and mature B cells after controlling for donors and organs with linear regression (****P*-value$<10^{-10}$). (**F**) Heatmap of TF activity means in B1 and mature B cells. The color and the number represent the average TF activity estimated by DoRothEA (*94*). Only the top 25 TFs that had significantly higher activities in B1 cells were shown here. TFs in TNF-α and NF-κB signaling pathway (*CEBPD*, *JUN*, *MYC*, *JUNB*) are boxed in red. (**G**) Representative flow cytometry plots showing the sorting strategy for the ELISpot experiment shown in Fig. 5E. The splenic B cells were gated from live single cells which were $CD3^{-}CD20^{+}$, excluding the top 1% of cells expressing the highest level of CD38 to avoid plasma cells (which should also be CD20 low and therefore not gated in), and split the rest into four fractions: $CCR10^{hi}$, $CCR10^{lo}CD27^{+}CD43^{+}$, $CCR10^{lo}CD27^{-}CD43^{+}$, and $CCR10^{lo}CD27^{-}CD43^{-}$. We then performed an ELISpot experiment on all four fractions without any stimulation. (**H**) Barplot of cell proportions with different predicted annotations in the four sort fractions: $CCR10^{hi}$, $CCR10^{lo}CD27^{+}CD43^{+}$, $CCR10^{lo}CD27^{-}CD43^{+}$, and $CCR10^{lo}CD27^{-}CD43^{-}$. (**I**) Dot plot showing gene expressions of *CCL27* and *CCL28* within the stromal cell populations. Only cell types with log normalized expression of *CCL27* or that of *CCL28* above 0.05 are shown here.

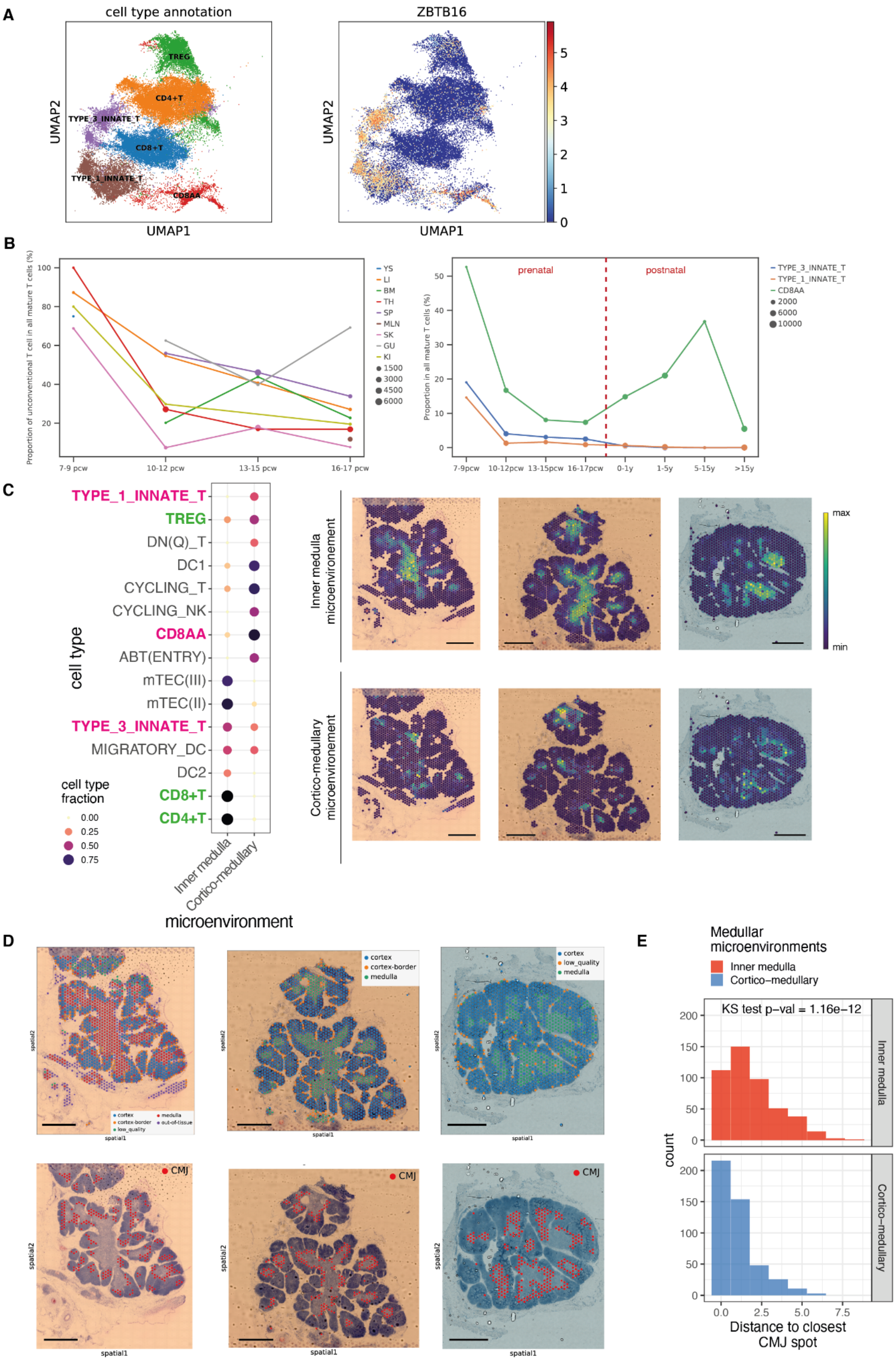
A
cell type annotation
TREG
CD4+T
TYPE_3_INNATE_T
CD8+T
TYPE_1_INNATE_T
CD8AA
UMAP1
UMAP2
ZBTB16
B
prenatal
postnatal
YS
LI
BM
TH
SP
MLN
SK
GU
KI
TYPE_3_INNATE_T
TYPE_1_INNATE_T
CD8AA
7-9 pcw
10-12 pcw
13-15 pcw
16-17 pcw
0-1y
1-5y
5-15y
>15y
C
TYPE_1_INNATE_T
TREG
DN(Q)_T
DC1
CYCLING_T
CYCLING_NK
CD8AA
ABT(ENTRY)
mTEC(III)
mTEC(II)
TYPE_3_INNATE_T
MIGRATORY_DC
DC2
CD8+T
CD4+T
cell type
cell type fraction
Inner medulla
Cortico-medullary
microenvironment
Inner medulla microenvironement
Cortico-medullary microenvironement
max
min
D
cortex
cortex-border
medulla
low_quality
out-of-tissue
CMJ
spatial1
spatial2
E
Medullar microenvironments
Inner medulla
Cortico−medullary
KS test p−val = 1.16e−12
count
Distance to closest CMJ spot

fig. S27. Distribution of unconventional T cells across gestation and in thymic tissue. (**A**) Left: Close-up view of mature T cells on UMAP embedding of all NK/T cells (as shown in fig. S4J). Type 1 innate T, type 3 innate T and CD8AA contain both αβT cells and γδT cells. Right: *ZBTB16* expression pattern overlaid onto the same UMAP plot. (**B**) Left: proportion of unconventional T cells in all mature T cells in different organs across different gestational age bins. Point size represents the number of mature T cells in a given organ within that age bin. Lines and points are color-coded by organs (YS: yolk sac; LI: liver; BM: bone marrow; TH: thymus; SP: spleen; MLN: mesenteric lymph node; SK: skin; GU: gut; KI: kidney). Right: proportion of each unconventional T cell subtype in all mature T cells in thymus across different age groups, using dataset from (*7*). Point size represents the number of mature T cells in thymus within that age bin. (**C**) Left: cell type contributions to medullary microenvironments containing mature T cells in thymus, identified with non-negative matrix factorization of spatial cell type abundances estimated with cell2location. The color and the size of the dots represent the relative fraction of the cell population assigned to the microenvironment. Unconventional T cell types are highlighted in magenta. Conventional T cell types are highlighted in green. Right: spatial locations of medullary microenvironments on different thymic slides, with the color representing the weighted contribution of each microenvironment to each spot (scale bar: 1 mm). (**D**) Top: annotation of tissue regions on Visium spots inferred by clustering of H&E image features. Bottom: location of interface region between cortex and medulla region, which we consider as the cortico-medullary junction (CMJ) (scale bar: 1 mm). (**E**) Histogram of Euclidean distance to the nearest CMJ spot for spots assigned to inner-medulla microenvironment (red) or cortico-medullary microenvironment (blue). Spots were assigned to a microenvironment if the NMF factor value for the microenvironment (see C) was above the 90% quantile. The *P*-value for the Kolmogorov–Smirnov test comparing the two distributions is reported.

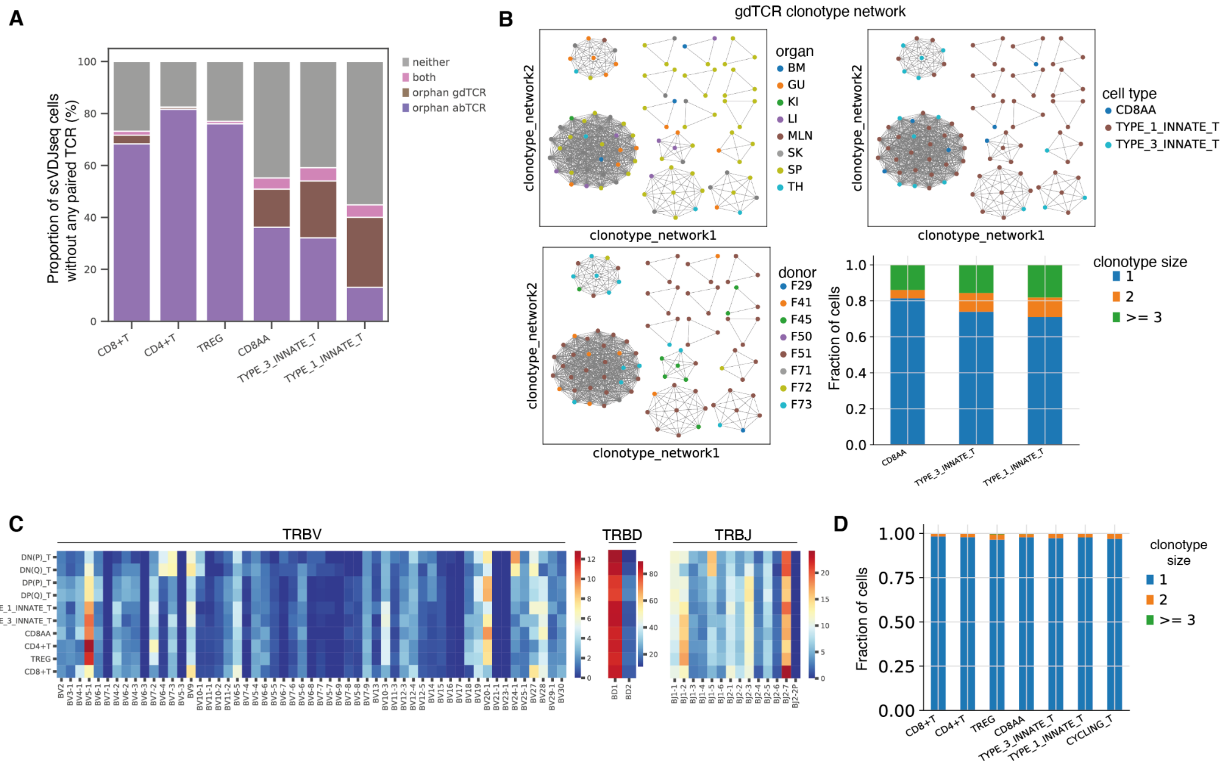

fig. S28. T cell receptor (TCR) sequence analysis. (**A**) Proportions of cells without any paired TCR expressing orphan γδTCR, i.e. one of γ or δ chain, or orphan αβTCR, i.e. one of α or β chain, or both or neither. (**B**) Top left: clonotype network graph of γδTCR. Each fully connected subnetwork represents a clonotype cluster, with each dot representing a cell. The dots are color-coded by organs (YS: yolk sac; LI: liver; BM: bone marrow; TH: thymus; SP: spleen; MLN: mesenteric lymph node; SK: skin; GU: gut; KI: kidney). Top right: γδTCR clonotype network graph color-coded by cell types. Bottom left: γδTCR clonotype network graph color-coded by donors. Bottom right: barplot of cell fractions with different γδTCR clonotype size across different unconventional T cell subtypes. (**C**) Heat map showing the percentage of each TRBV, TRBD and TRBJ gene segment present in different T cell subtypes. (**D**) Barplot of cell fractions with different clonotype size across different mature T cell subtypes.

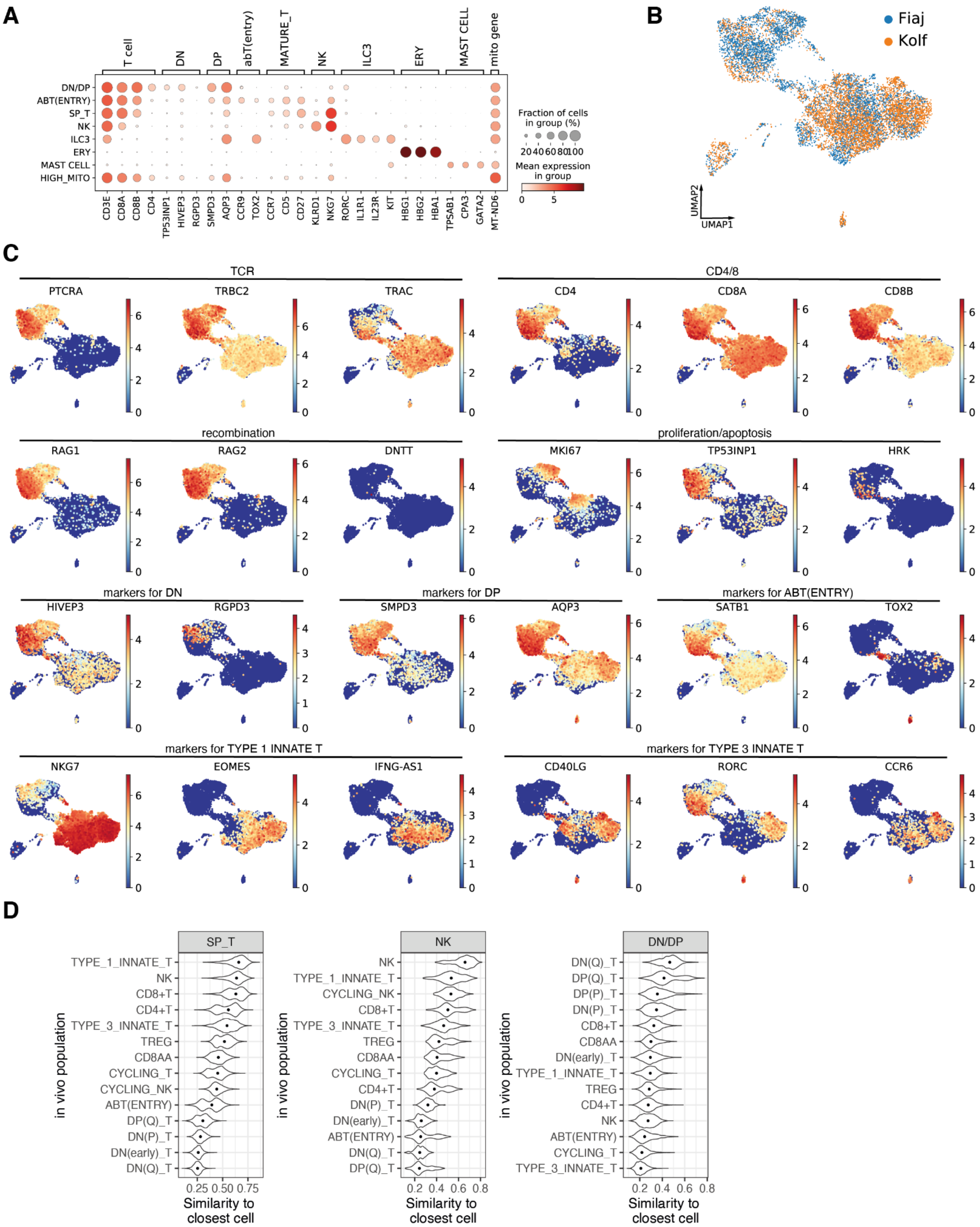

fig. S29. Analysis of artificial thymic organoids scRNA-seq data. (**A**) Dot plot of marker genes for ATO cell populations. (**B**) Cells colored by the starting iPSC lines in ATO overlaid on UMAP embedding shown in Fig. 6F. (**C**) Expression of T cell marker genes in ATO overlaid on UMAP embedding shown in Fig. 6F. (**D**) Violin plots of similarity to closest in vivo cell (*x*-axis) for each cell type in the in vivo dataset (*y*-axis) for in vitro single-positive T

cells (SP_T), NK cells and developing T cells (DN/DP). Similarities are calculated in the scVI latent space for lymphoid cells after mapping in vitro cells with scArches.

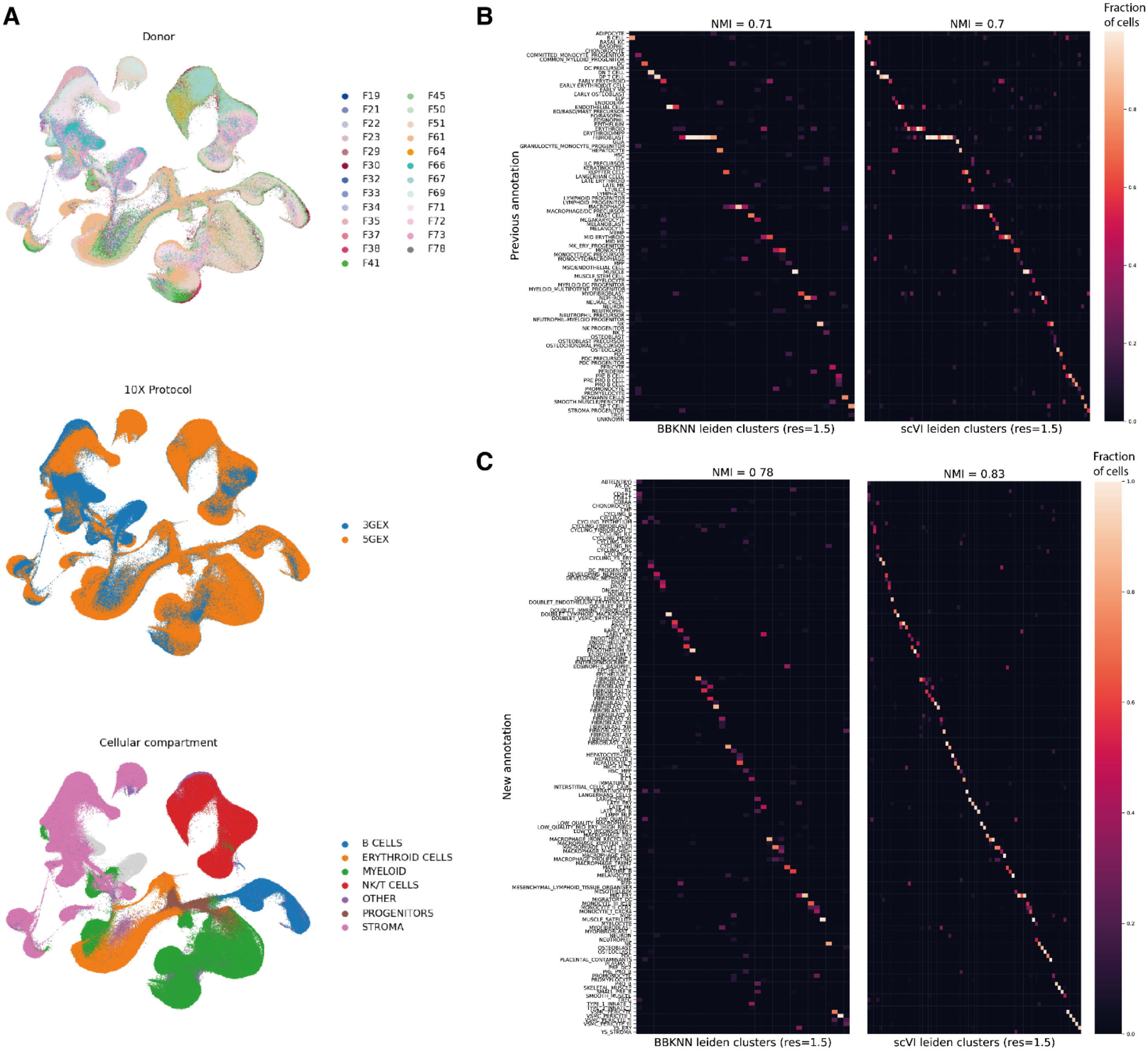

fig. S30: Comparison across data integration methods. (**A**) UMAP embeddings of scRNA-seq profiles after data integration with BBKNN colored by (top to bottom): 10X library prep protocol, donor ID, cellular compartment. (**B**) heat map of confusion matrices between Leiden clusters and previously annotated cell type labels, with clustering on BBKNN integration (left, 37 clusters) or scVI integration (right, 75 clusters) of the full dataset (clustering resolution = 1.5). (**C**) heat map of confusion matrices between Leiden clusters and newly annotated cell type labels, with clustering on BBKNN integration (left, 37 clusters) or scVI integration (right, 75 clusters) of the full dataset (clustering resolution = 1.5). For each confusion matrix, the normalized mutual information (NMI) score between cluster labels and annotation labels is shown.

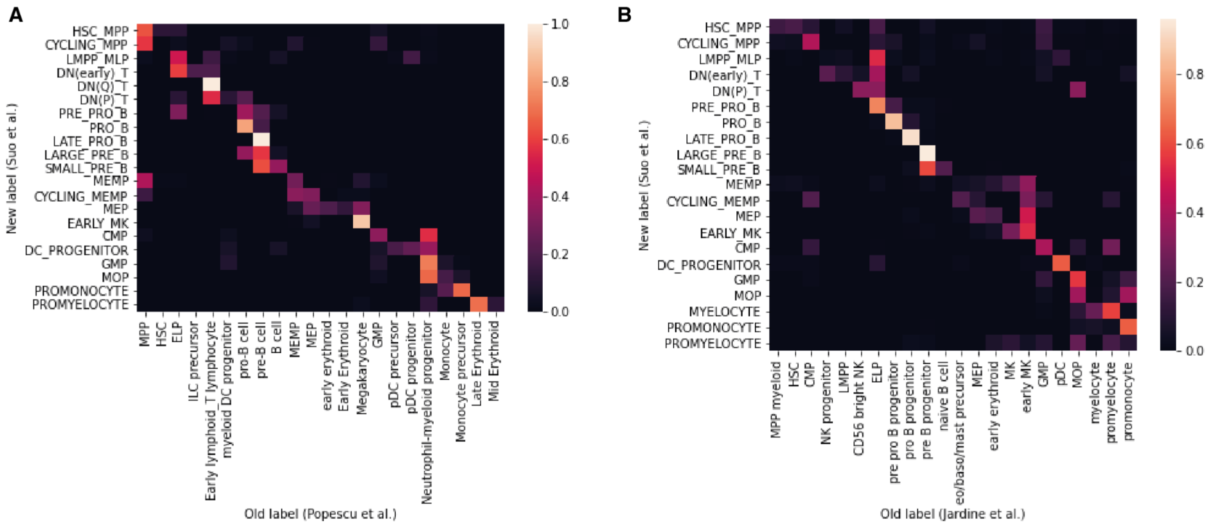

fig. S31: Agreement between annotations of progenitor cells in this study and annotations from fetal bone marrow atlas (*11*)**.** (**A**) Confusion table between annotations of progenitor cells in bone marrow in this study and the annotations for the same cells in fetal bone marrow atlas (*11*). The color is proportional to the fraction of cells in the new population with a given old label. Only bone marrow cells for which a previous annotation was available are shown (32,274/46,448 bone marrow cells). (**B**) Confusion table between annotations of progenitor cells in bone marrow in this study and the annotations for the same cells in fetal liver atlas (*3*). The color is proportional to the fraction of cells in the new population with a given old label. Only liver cells for which a previous annotation was available are shown (11,330/26,377 liver cells).

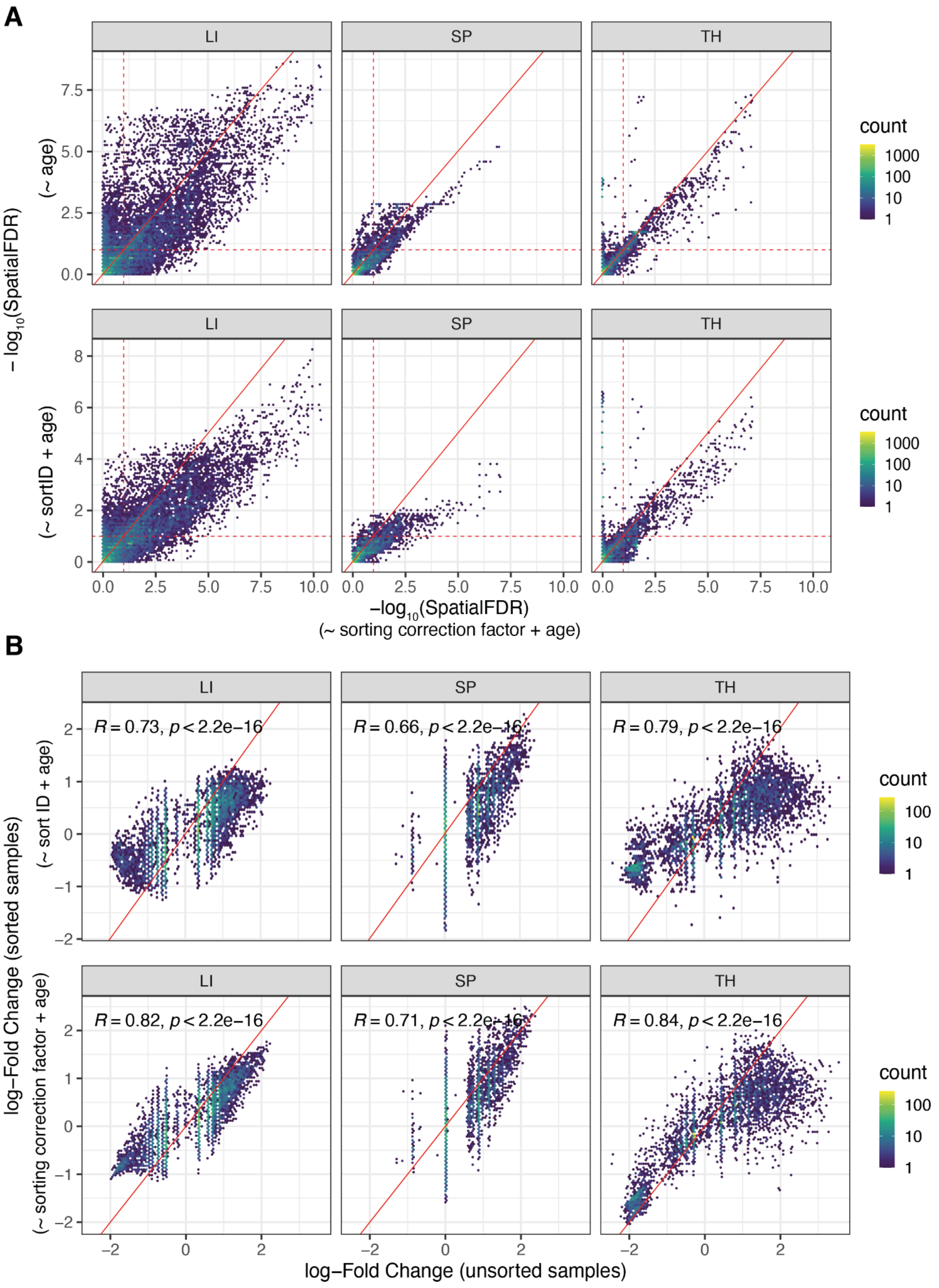
A
LI
SP
TH
− log10(SpatialFDR)
(~ age)
(~ sortID + age)
−log10(SpatialFDR)
(~ sorting correction factor + age)
count
1000
100
10
1
B
R = 0.73, p < 2.2e−16
R = 0.66, p < 2.2e−16
R = 0.79, p < 2.2e−16
R = 0.82, p < 2.2e−16
R = 0.71, p < 2.2e−16
R = 0.84, p < 2.2e−16
log−Fold Change (sorted samples)
(~ sort ID + age)
(~ sorting correction factor + age)
log−Fold Change (unsorted samples)

fig. S32: Validation of quantification of FACS effect on cell abundances in Milo neighborhoods. (**A**) Scatter plot of SpatialFDR (in $-\log_{10}$ scale) estimated in test for differential abundance across gestational age, regressing out continuous FACS factor (*x*-axis), without regressing out FACS effect (*y*-axis, top) and regressing out FACS protocol label ($CD45^+$/$CD45^-$/unsorted) (*y*-axis, bottom). Results for the test on cells from liver (LI), spleen (SP) and thymus (TH) are shown. The dotted red lines indicate the significance threshold of 10% SpatialFDR. (**B**) Scatterplot of log-fold change estimated in test for differential abundance across gestational age testing on the subset of unsorted samples (*x*-axis) and on FACS-isolated samples (y-axis), regressing out the FACS protocol label (top) or the FACS factor (bottom).

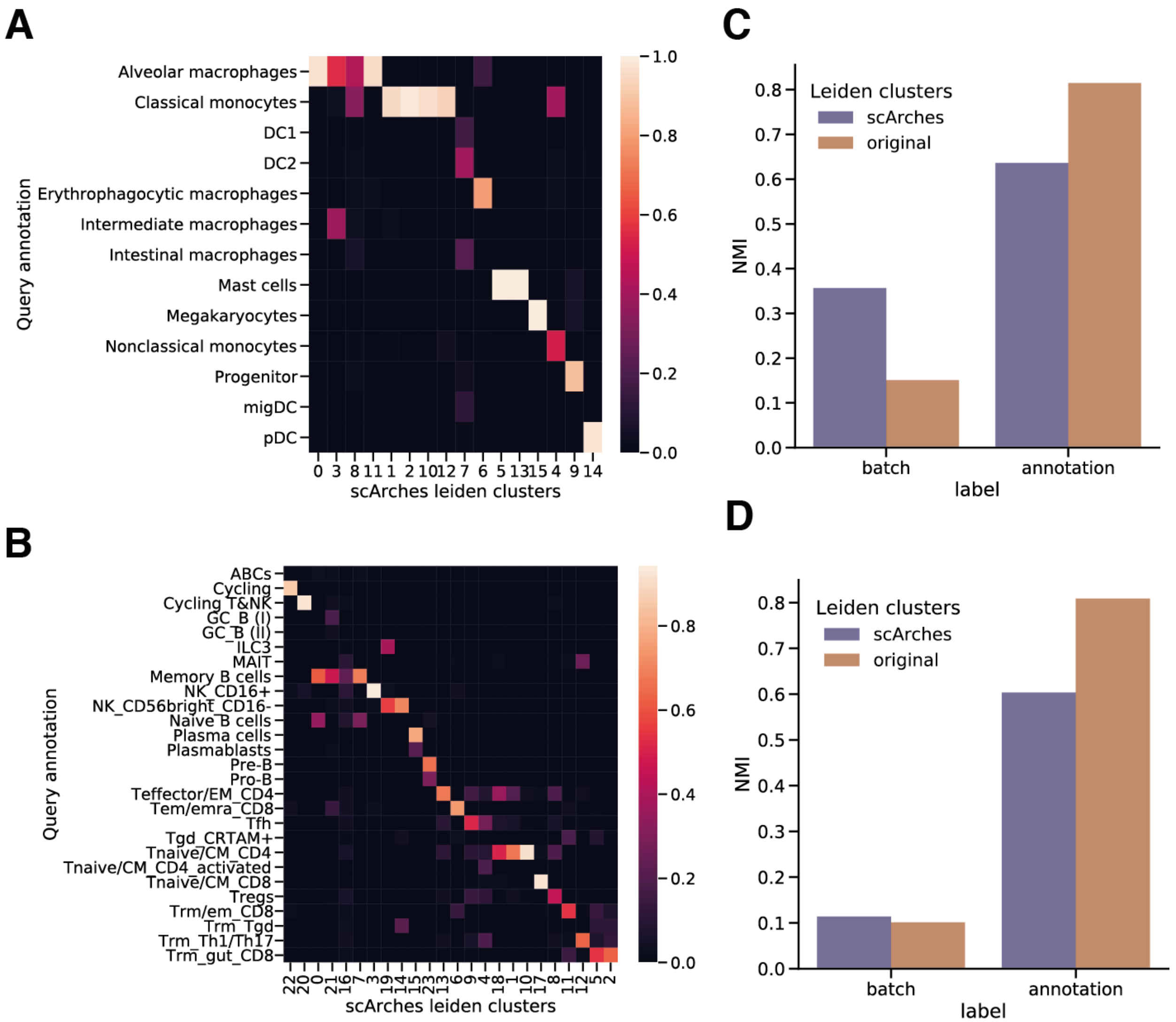

fig. S33: Validation of biological conservation after scArches mapping of adult cells to prenatal reference. (**A** and **B**) Heat maps of confusion matrix between adult immune cell annotations (*21*) and Leiden clusters obtained with latent dimensions after mapping adult cells on the prenatal reference with scArches, for myeloid cells (A) and lymphoid cells (B). (**C** and **D**) Barplots of normalized mutual information (NMI) between technical batch or cell type annotation and Leiden clusters from scArches mapping or the clusters in the original BBKNN embedding *(21*).

Supplementary Tables

table S1: Table_S1.csv (separate file)
Differential expression analysis results for test on gestation-stage specific macrophage and mast cells neighborhoods

table S2: Table_S2.csv (separate file)
Differential expression analysis results for comparison of bone marrow and peripheral CCR2hi monocytes

table S3: Table_S3.csv (separate file)
Differential expression analysis results for test on gestation-stage specific NK cells neighborhoods

table S4: Table_S4.csv (separate file)
Differential expression analysis results for comparison of thymic and peripheral mature T cells

table S5: Table_S5.csv (separate file)
Cell types included in each lineage progenitors shown in Fig. 4A.

table S6: Table_S6.csv (separate file)
Results from the ELISpot experiment.

table S7: Table_S7.csv (separate file)
Metadata for sequenced samples.

[File] Prefix for raw sequencing files
[Organ] YS: yolk sac; LI: liver; BM: bone marrow; TH: thymus; SP: spleen; MLN: mesenteric lymph node; SK: skin; GU: gut; KI: kidney
[Sort_id] Sorting scheme used for each sample. CD45P: $CD45^{+}$, CD45N: $CD45^{-}$, CD137: $CD3^{+}$ and $CD137^{hi}$; MAIT: $CD3^{+}CD25^{hi}$, TOT: Unsorted
[Age] Age of donor (post conceptional weeks)
[Method] 3GEX: 10X 3' chemistry gene expression profiling, 5GEX: 10X 5' chemistry gene expression profiling
[Donor] Unique ID assigned for each donor
[Sample]: Sample description (DonorID-Organ-Sort-File)
[Sex] Sex of donor
[Processing_method] Protocol used for dissociation
[Anatomical_region] Region of dissection
[N_cells] Total number of cells in sample (post-QC)
[Termination_method] Termination of pregnancy method

table S8: Table_S8.csv (separate file)
Primers used in targeted γδTCR amplification.

table S9: Table_S9.csv (separate file)
Metadata for samples profiled with Visium 10X spatial transcriptomics.

[File] Prefix for raw sequencing files

[Image_id] Unique ID assigned to each image for H&E staining
[Organ] Sampled tissue
[Donor] Unique ID assigned for each donor
[Slide_number] Unique ID for Visium 10X slide (experimental batch)
[Visium_area_id] Unique ID for position of tissue on Visium 10X slide
[Age] Age of donor (post conceptional weeks)
[Sex] Sex of donor
[Digestion time] Time of digestion in tissue optimization (minutes)
[Termination_method] Termination of pregnancy method

Supplementary Materials for

Mapping the developing human immune system across organs

Chenqu Suo*, Emma Dann*, Issac Goh, Laura Jardine, Vitalii Kleshchevnikov, Jong-Eun Park, Rachel A. Botting, Emily Stephenson, Justin Engelbert, Zewen Kelvin Tuong, Krzysztof Polanski, Nadav Yayon, Chuan Xu, Ondrej Suchanek, Rasa Elmentaite, Cecilia Domínguez Conde, Peng He, Sophie Pritchard, Mohi Miah, Corina Moldovan, Alexander S. Steemers, Pavel Mazin, Martin Prete, Dave Horsfall, John C. Marioni, Menna R. Clatworthy†, Muzlifah Haniffa†, Sarah A. Teichmann†

Correspondence to: mrc38@cam.ac.uk (M.C.), m.a.haniffa@newcastle.ac.uk (M.H.); st9@sanger.ac.uk (S.A.T.)

This PDF file includes:

Supplementary Materials and Methods
Figs. S1 to S33
Tables S1 to S9

Supplementary Materials and Methods

Tissue acquisition and processing

All human developmental tissue samples used for this study were obtained from the MRC–Wellcome Trust-funded Human Developmental Biology Resource (HDBR; http://www.hdbr.org) with written consent and approval from the Newcastle and North Tyneside NHS Health Authority Joint Ethics Committee (08/H0906/21+5).

All tissues were processed into single-cell suspensions immediately upon receipt. Tissue was first minced in a tissue culture dish using scalpel. It was then digested with type IV collagenase (final concentration of 1.6 mg/ml; Worthington) in RPMI (Sigma-Aldrich) supplemented with 10% fetal bovine serum (FBS; Gibco), at 37°C for 30 min with intermittent agitation. Digested tissue was then passed through a 100-µm cell strainer and cells were pelleted by centrifugation at 500*g* for 5 min at 4°C. Cells were then resuspended in 5 ml of red blood cell lysis buffer (eBioscience) and left for 5-10 min at room temperature. It was then topped up with flow buffer (PBS containing 2% (v/v) FBS and 2 mM EDTA) to 45 ml prior to cell counting and antibody staining. Single-cell suspensions were generated from 76 samples across yolk sac (7), liver (6), spleen (30), thymus (4), kidney (2), and skin (27) of 16 donors. The ages of the donors spanned from 4 pcw (post conception weeks) to 17 pcw. The metadata of all samples, including previously published data, can be found in table S7.

Single-cell RNA sequencing experiment

Dissociated cells were stained with anti-CD45 antibody (BUV395 anti-human CD45 antibody, BD Biosciences, 563791) and DAPI (Sigma-Aldrich, D9542) prior to sorting. For all FACS experiments performed in this study, DAPI was used at a final concentration of 2.8 µM, and all antibody solutions were used at a final concentration of 2 µl per 100 µl cell suspensions containing fewer than 5 million cells. Sorting by flow cytometry was performed with BD FACSAria Fusion Flow Cytometer. The $CD45^+$ fraction was sorted from $DAPI^-CD45^+$ gate and $CD45^-$ fraction was sorted from $DAPI^-CD45^-$ gate. CD45 gating was contiguous so that no live cells were lost in sorting.

For scRNA-seq experiments, either Chromium single cell 3′ reagent kit or Chromium single cell V(D)J reagent kits from 10X Genomics were used. Unsorted, or $DAPI^-CD45^+$, or $DAPI^-CD45^-$ FACS-isolated cells were loaded onto each channel of the Chromium chip following the manufacturer's instructions before droplet encapsulation on the Chromium controller. Single-cell cDNA synthesis, amplification, gene expression (GEX) and targeted B cell receptor (BCR) and T cell receptor (TCR) libraries were generated. Targeted enrichment for γδTCR was performed following the TCR enrichment protocol from 10X with customized primers binding to the constant region of the TRD and TRG genes as described (*75*). Primers are listed in table S8.

Sequencing was performed on the Illumina Novaseq 6000 system. The gene expression libraries were sequenced at a target depth of 50,000 reads per cell using the following parameters: Read1: 26 cycles, i7: 8 cycles, i5: 0 cycles; Read2: 91 cycles to generate 75-bp paired-end reads. BCR and TCR libraries were sequenced at a target depth of 5000 reads per cell.

Cell cultures for artificial thymic organoid (ATO)

MS5 line transduced with human DLL4 was obtained from G. Crooks (UCLA) as a gift. The MS5-hDLL4 cells were cultured in DMEM (Gibco) with 10% FBS. Two iPSC lines were used in this study. Cell lines HPSI0114i-kolf_2 (Kolf) and HPSI0514i-fiaj_1 (Fiaj) were obtained from the Human Induced Pluripotent Stem Cell initiative (HipSci: www.hipsci.org) collection. All iPSC lines were cultured on vitronectin (diluted 1:25 in PBS; Gibco) coated plates, in TeSR-E8 media (Stemcell Technologies).

We followed the PSC-ATO protocol as previously described (*61*). iPSC cells were harvested as a single-cell suspension and seeded (3×10^6 cells per well) in GFR reduced Matrigel (Corning) - coated 6-well plates in X-VIVO 15 media (Lonza), supplemented with rhActivin A, rhBMP4, rhVEGF, rhFGF (all from R&D Systems), and ROCK inhibitor (Y27632; LKT Labs) on day −17, and only rhBMP4, rhVEGF and rhFGF on days −16 and −15. Cells were harvested 3.5 days later, and isolated by FACS for $CD326^{-}CD56^{+}$ (PE anti-human CD326 antibody, Biolegend, 324205; APC anti-human CD56 antibody, Biolegend, 318309) human embryonic mesodermal progenitors (hEMPs).

Isolated hEMPs were combined with MS5-hDLL4 at a ratio of 1:50. Two or three cell-dense droplets (5×10^5 cells in 6 μl hematopoietic induction medium) were deposited on top of an insert in each well of a six-well plate. Hematopoietic induction medium composed of EGM2 (Lonza) supplemented with ROCK inhibitor and SB blocker (TGF-β receptor kinase inhibitor SB-431542; Abcam) was added into the wells outside the inserts so that the cells sat at the air-liquid interface. The organoids were then cultured in EGM2 with SB blocker for 7 days (days −14 to −7), before the addition of cytokines rhSCF, rhFLT3L, rhTPO (all from Peprotech) between days −6 to 0. These 2 weeks formed the hematopoietic induction phase. On day 1, media was changed again to RB27 (RPMI supplemented with B27 (Gibco), ascorbic acid (Sigma-Aldrich), penicillin/streptomycin (Sigma-Aldrich) and glutamax (Thermo Fisher Scientific)) with rhSCF, rhFLT3L and rhIL7. The organoids can be maintained in culture for 7 more weeks in this medium.

For dissociation and checking of ATO, a cell scraper was used to detach ATOs from cell culture insert membranes and detached ATOs were then submerged in cold flow buffer. Culture inserts were washed and detached ATOs were pipetted up and down to form single-cell suspension before passing through a 50-μm strainer. Cells were then stained with designed panels of antibodies and analyzed by flow cytometry. FACS was performed at the same time and live human $DAPI^{-}$anti-mouse $CD29^{-}$ (APC/Cy7 anti-mouse CD29 antibody, Biolegend, 102225) cells were sorted for week 3 ATO cells, and live ($DAPI^{-}$) cells were sorted for week 5 and week 7 ATO cells before loading onto each channel of the Chromium chip from Chromium single cell V(D)J kit (10X Genomics).

Visium

OCT embedded freshly frozen samples were used for 10X Genomics Visium, and samples were processed following manufacturer's instructions. All tissues were sectioned with a thickness of 15 μm on a cryostat (OTF5000, Bright instruments). Tissue optimization was then performed with an 18-min permeabilization for fetal spleen and liver, whereas a 24-min permeabilization

was used for fetal thymus. The spatial gene expression library was then generated following the manufacturer's protocol. All images for this process were acquired with a Zeiss AxioImager (Carl Zeiss Microscopy) and a 20X air objective (0.8 NA) using either fluorescence (Zeiss Axiocam 503 monochrome camera) for optimization or brightfield mode (Zeiss Axiocam 105 color camera) for H&E imaging. ZEN (Blue edition) v.3.1 was used for acquisition and stitching of the image tiles. The metadata of all samples can be found in table S9.

Single molecule fluorescence in situ hybridization (smFISH)

The smFISH technique RNAscope was performed on thymus, spleen, and gut sections, using the RNAScope 2.5 LS multiplex fluorescent assay (ACD, Bio-Techne) on the automated BOND RX system (Leica). Prior to running RNAscope probes of interest, positive and negative control probes were used for optimization of these tissues. Tissue sections were placed onto superfrost plus slides (Fisher scientific) and stained for DAPI (nuclei) and three or four probes of interest, with fluorophores opal 520, opal 570, opal 650 and atto 425. DAPI was used at 1:50,000 concentration; opals at 1:1000 (1:500 for thymus) and atto 425 at 1:400 concentration.

For the fetal gut and spleen, OCT-embedded freshly frozen samples were sectioned to 10 µm-thick. Following optimization, sections were pretreated offline for 15 min with chilled 4% paraformaldehyde and dehydrated through an ethanol series (50%, 70%, 100%, 100% ethanol), before processing on the Leica BOND RX with protease IV for 30 min at room temperature. The sections were imaged on a Perkin Elmer Opera Phenix High Content Screening System (16-bit sCMOS camera, PerkinElmer) with a 20X water objective (High NA, PerkinElmer). Due to high levels of endogenous autofluorescence, we imaged one of the spleen sections (fig. S21A) with a confocal microscope (Leica SP8) with a 40X 1.3NA oil immersion objective and SP8 Leica HyD and PMT detectors. Emission spectral filters were set for DAPI, opal 520 (*VPREB1*), opal 570 (*RAG1*), opal 650 (*CDH5*). Images were processed with Fiji as follows. All channels were subjected to *z*-max projection followed by 2D Gaussian filtering (sigma = 0.5 pixels). The DAPI channel was flat field corrected (biovoxxel – pseudo flat field correction plugin) with a rolling ball radius of 200 pixels. The large image was cropped around the tissue area to remove flat field corrections edges.

Due to the high cellular density in thymic sections, we used 3 µm-thick FFPE sections. These were treated on the Leica Bond RX with epitope retrieval 2 for 15 min at 95°C and protease III for 15 min at 40°C. FFPE thymus did not require any offline pretreatment. Imaging was performed on an Operetta CLS High Content Screening System (16-bit sCMOS camera, PerkinElmer) with a 40X water objective (High NA, PerkinElmer) and 2-µm *z*-steps.

Cells were identified and annotated manually with an in-house OMERO platform (https://www.openmicroscopy.org/omero/).

scRNA-seq analysis

Preprocessing

The gene expression data was mapped with cellranger 3.0.2 to an Ensembl 93 based GRCh38 reference (10X-distributed 3.0.0 version). Ambient RNA was removed with cellbender v0.2.0

(*76*). Low-quality cells were filtered out (minimum number of reads = 2000, minimum number of genes = 500, Scrublet (v0.2.3) (*77*) doublet detection score <0.4).

In order to identify possible maternal contamination, the samples were pooled on a per-donor basis and processed with souporcell (v.2.4.0) (*78*). The common GRCh38 variants file (SNPs with ≥2% frequency from 1k genomes) provided by souporcell authors was used. The pipeline was run twice, setting the number of genotype clusters to 1 and 2 to obtain models for no maternal contamination and possible maternal contamination. The better of these models was identified via BIC (Bayesian Information Criterion), calculated using the formula below:

$$BIC = kn\log(m) - 2l$$

Whereby k is the number of genotype clusters set for each souporcell run, n denotes the number of loci used for genotype deconvolution, m is the cell count for a given donor, and l is the log likelihood obtained after running the pipeline with each k. In two donors (F19 and F37), the BIC was smaller when $k = 2$. The cells with the minor genotype were identified as possible maternal contaminants, which mainly consisted of NK cells, monocytes, mature B and T cells. The cells of minor genotype from the remaining donors were further screened for similar cell compositions, and a further donor (F33) was identified with possible maternal contamination. For these three donors, cells from the minor genotype were excluded from the downstream analysis.

Data integration and annotation

Data normalization and preprocessing were performed using the Scanpy workflow (v1.8.1) (*79*). We normalized raw gene read counts by sequencing depth in each cell (*scanpy.pp.normalize_per_cell,* with parameters *counts_per_cell_after=10e4*) and performed *ln(x)+1* transformation. Expression levels reported in this manuscript refer to normalized and log-transformed gene read counts. We then selected highly variable genes (HVG) for joint embedding by dispersion (*scanpy.pp.highly_variable_genes* with parameters *min_mean = 0.001*, *max_mean = 10*). We considered the 10X chemistry (5′ and 3′) and the donor ID for each cell as the technical covariates to correct for. We performed dimensionality reduction and batch correction using the scVI model (*12*) as implemented in scvi-tools (v0.14.5) (*80*). For model specification and training we used the recommended parameters to enable scArches mapping (*dropout_rate = 0.2*, *n_layers = 2*). To verify conservation of biological variation after integration, we collected and harmonized the available cell type labels from the published datasets (66% of cells) and quantified the agreement between labels across different datasets in the cell clusters identified post-integration, using the normalized mutual information (NMI) score, as implemented in *scikit-learn* (*81*). The model was trained on raw counts of the 7500 most highly variable genes, excluding cell cycle genes and TCR/BCR genes (*7*) with 20 latent dimensions. These parameters (number of HVGs, number of latent dimensions, exclusion/inclusion of cell cycle and TCR/BCR genes) were picked through a parameter sweep, focused on maximizing the NMI between clusters after embedding and pre-existing cell type label annotation (data not shown). Unless otherwise specified, cell clustering was performed using the Leiden algorithm (*82*) with resolution = 1.5 on a k-nearest neighbor graph with $k = 30$. To verify that our cell type clusters were robust to the choice of integration method, we performed in parallel integration on the full dataset using BBKNN (*83*) as previously described (*7*) (fig. S30A). We found that clustering post-integration both with scVI and BBKNN was consistent with previous annotations (fig. S30B).

To annotate fine cell populations across tissues, we clustered cells in the scVI latent space and preliminarily assigned cells to broad lineages examining expression of marker genes and assigning putative cell labels based on previous annotations (we propagated existing cell type labels to unannotated cells by taking the most abundant label in the *k*-nearest neighbors for each unannotated cell). For each broad lineage we repeated scVI integration and clustering as described above and defined further subsets (see hierarchy in fig. S5). Leiden clusters for the highest resolution subsets (Stroma, megakaryocyte/erythroid, progenitors, lymphoid, myeloid) were annotated manually, using marker panels shown in fig. S4. A common subset of progenitor cells was included in scVI embeddings for all hematopoietic-derived cell subsets (megakaryocyte/erythroid, lymphoid, myeloid, NK/T), to allow feature selection and dimensionality reduction to capture the differentiation process of different lineages. A distinct embedding of progenitor cells was then used to finely annotate these cell populations (fig. S4E-F). Blood and immune cell progenitor annotation was based on the subsets and marker genes identified by Jardine et al. (*11*) and Popescu et al. (*3*) (SFig. 31). Macrophage subsets were annotated by analysis of marker genes from human studies in both adult and developmental tissues (*18–20*, *62*) and by unbiased marker gene detection (using *scanpy.tl.rank_genes_groups*) and groupings were defined as follows: "LYVE1^{hi}" expressing *F13A1*, *LYVE1*, and *SPP1*; "Iron-recycling" expressing the highest levels of ferroportin (*SLC40A1*) and phosphatidylserine receptor *TIMD4* but best characterized by expression of *CD5L*, *VCAM1*, and *APOE*; "MHC class II^{hi}" expressing the highest levels of *HLA-DRA*, *HLA-DPA1*, and *CLEC7A* among macrophages; "Kupffer-like" expressing endothelial transcripts *ENG*, *KDR*, and *CAV*; "TREM2" with expression of microglia-associated transcripts *TREM2* and *P2RY12*; "Osteoclasts" expressing characteristic *MMP9* and *ACP5*; and "Proliferating macrophages" expressing genes associated with cell-cycle progression (fig. S4H). Fetal macrophage subsets show a phenotype corresponding to (TIMD4/LYVE1/FOLR2) TLF+ murine macrophages, with potentially additional heterogeneity within the human fraction (*100*) (fig. S4H).

We verified that refined annotations were highly consistent with unsupervised clustering post-integration on the full dataset both with scVI and BBKNN (fig. S30C).

After full annotation 23,156 cells (2.5% of total) were assigned to low quality clusters. These comprised doublet clusters, maternal contaminants clusters and clusters displaying a high percentage of reads from mitochondrial genes.

Differential abundance analysis

We tested for differences in cell abundances associated with gestational age or organ using the Milo framework for differential abundance testing (*22*), with the python implementation milopy (https://github.com/emdann/milopy). Briefly, we subsetted the dataset to cells from libraries obtained with CD45$^+$ FACS, CD45$^-$ FACS or no FACS. In addition, we excluded FACS-isolated samples for which we were not able to recover the true sorting fraction quantification. In total, we retained 228,731 lymphoid cells and 214,874 myeloid cells. To further minimize the differences in cell numbers driven by differences in FACS efficiency, we calculated a FACS correction factor for each tissue sample s sorted with gate i (where i is either CD45$^+$ or CD45$^-$):

$$f_s = \log(p_i S / S_i)$$

where p_i represents the true proportion of cells from gate i in the tissue samples from the same organ and donor, S represents the total number of cells recovered from both CD45$^+$ and CD45$^-$ gates for this organ and donor and S_i represents the number of cells recovered in gate i. For the

unsorted samples we set $f_s = 0$. Encoding the true proportions of CD45$^{+/-}$ cells with f_s reduced the proportion of false positives that were found without regressing out the effect of FACS isolation, or when encoding the effect of sorting as a label rather than a proportion (where CD45^{+} = 1, CD45^{-} = -1, unsorted = 0) (fig. S32A). To validate this approach, we confirmed agreement between estimated fold-changes testing on unsorted samples with the fold-changes estimated accounting for FACS on sorted samples from the same organ (fig. S32B).

We constructed a KNN graph of remaining cells using similarity in the scVI embedding (k = 30 for test across gestation, k = 100 for test across tissues). We assigned cells to neighborhoods on the KNN graph using the function *milopy.core.make_nhoods* (parameters: *prop = 0.05*). We then counted the number of cells belonging to each sample in each neighborhood, creating a cell count matrix with rows representing neighborhoods and columns representing samples (using the function *milopy.core.count_cells*). We assigned each neighborhood a cell type label based on majority voting of the cells belonging to that neighborhood. We assigned a "Mixed" label if the most abundant label is present in less than 50% of cells within that neighborhood.

<u>Differential abundance across time:</u> To test for differences in cell numbers across gestational age, we divided the sample ages into six equally sized bins (bin size = 2 pcw) and excluded from the cell count matrix samples from organs where less than three consecutive age bins were profiled (yolk sac, mesenteric lymph node, kidney, gut). For the matrix of cell counts from samples in each organ, we modeled the cell count $c_{n,s}$ of cells from sample *s* in neighborhood *n* as a negative binomial generalized linear model (NB-GLM):

$$c_{n,s} \sim NB(\mu_{n,s}, \phi_n)$$

where $\mu_{n,s}$ is the mean number of cells from sample *s* in neighborhood *n* and ϕ_n is the dispersion parameter. We used a log-linear model to model the effect of age on cell counts:

$$\log \mu_{n,s} = f_s\beta_n^{\text{facs}} + a_s\beta_n^{\text{age}} + \log L_s$$

here:
- L_s is the sum of counts of cells of sample *s* over all the neighborhoods.
- a_s is the age bin associated to sample *s*.
- β_n^{age} *i*s the regression coefficient encoding the effect of age on the number of cells in neighborhood *n*, that represents the log-fold change (logFC) that can be interpreted as the per-bin linear change in neighborhood cell abundance.
- f_s is the FACS correction factor associated to sample *s*.
- β_n^{facs} is the regression coefficient encoding the effect of CD45 enrichment on the number of cells in neighborhood *n*.

To control for multiple testing, we used the weighted BH correction as previously implemented (*22*). In addition, we tested in parallel for differential abundance associated with the library prep protocol (instead of gestational age) and excluded neighborhoods where we detected significant differential abundance associated with library prep protocol (SpatialFDR < 0.1). We applied this stringent filtering step instead of including the library prep protocol as a covariate in the model (as described below for the test on organ enrichment) to exclude from downstream analysis false positive neighborhoods identified in a small number of thymus samples, where we observed strong confounding between age bins and library prep protocol.

To detect markers of early-specific neighborhoods (SpatialFDR < 0.1, logFC < 0) and/or late-specific neighborhoods (SpatialFDR < 0.1, logFC > 0) in cell type *c* and organ *o*, we tested for differential expression between cells from organ *o* assigned to the significant neighborhoods labeled as cell type *c* and cells belonging to all other neighborhoods labeled as cell type *c*. We used the t-test implementation in scanpy (*scanpy.tl.rank_genes_groups*, *method = "t-test_overestim_var"*). Genes expressed in > 70% of tested cells were excluded. We considered genes as significantly overexpressed (i.e. markers) if the differential expression logFC > 1 and FDR < 0.1%. Gene set enrichment analysis was performed using the implementation of the EnrichR workflow (*84*) in the python package gseapy (https://gseapy.readthedocs.io/). The list of significantly overexpressed genes for all organs and cell types where differential expression testing was carried out can be found in table S1 and S3.

Differential abundance between organs: We modeled the cell counts $y_{n,s}$ for each experimental sample *s* in neighborhood *n* by a Negative Binomial distribution:

$$y_{n,s} = NB(\mu_{n,s}, \phi_{n,s})$$

Where the expected count value $\mu_{n,s}$ is given by the following log-linear model

$$\log \mu_{n,s} = p_s \beta_n^{\text{prep}} + f_s \beta_n^{\text{facs}} + o_s \beta_n^{o} + \log L_s$$

here:

- L_s is the sum of counts of cells of sample *s* over all the neighborhoods.
- o_s is a binary factor indicating whether sample *s* is derived from organ *o*.
- β_n^{o} is the regression coefficient encoding the effect of the organ on the number of cells in neighborhood *n*, that represents the log-fold change in abundance of cells from organ *o* compared to the cells from other organs.
- f_s is the FACS correction factor associated to sample *s*.
- β_n^{facs} is the regression coefficient encoding the effect of CD45 enrichment on the number of cells in neighborhood *n*.
- p_s is the binary design matrix associating sample *s* to a library prep protocol.
- β_n^{prep} is the regression coefficient encoding the effect of the library prep protocol on the number of cells in neighborhood *n*.

We estimated β_n^{o} for each *n* and *o* by fitting the NB-GLM to the count data for each neighborhood, i.e. by estimating the dispersion $\phi_{n,s}$ that models the variability of cell counts in replicate samples for each neighborhood. To control for multiple testing we use the weighted BH correction as implemented by Dann et al. (*22*).

We considered the neighborhoods where $\beta_n^{o} > 0$ and SpatialFDR < 0.01 as cell subpopulations that show organ-specific transcriptional signatures.

Having identified a subset of neighborhoods overlapping a cell type or a subset of transcriptionally related cell types $\hat{c}$ that were enriched in an organ $\hat{o}$, we performed differential expression (DE) analysis between these cells and cells from cell type *c* in other organs. Let $x_i^{g,c,s}$ be the raw gene expression counts of gene g in the *i*th cell from sample *s* and of cell type *c*. We first aggregated single-cell expression profiles into pseudo-bulk expression profiles $\hat{x}$ for each (c,s) (as recommended by (*85*, *86*)):

$$\bar{x}^{g,c,s} = \sum_i x_i^{g,c,s}$$

We next defined a subset of cell types and samples where we will fit the model to test for differentially expressed genes in organ $\hat{o}$. First, we subsetted to the samples from donors where organ $\hat{o}$ and at least 3 other organs were profiled. We then identified 3 cell types $c_j^{\text{ctrl}} \neq \hat{c}$ where at least 2 pseudobulks aggregated from at least 50 cells are profiled in the selected donors, for organ $\hat{o}$ and at least 3 other organs. These cell types represent populations where we don't expect to see biological differences in expression in organ $\hat{o}$.

After sample selection, we subsetted the number of genes for DE testing selecting the top 7500 highly variable genes in $\bar{x}^{\hat{c},s}$ using the method implemented in the R package scran. We further excluded genes where the sum of expression values across pseudobulks from either c_j^{ctrl} or $\hat{c}$ is equal to 0.

These steps yielded a P-by-G data matrix $\bar{X}$, where P is the number of selected pseudobulks and G is the number of selected genes.

We modeled the mRNA counts of gene g in pseudobulk p by a NB-GLM:

$$\bar{x}^{g,p} = NB(\mu_{g,p}, \phi_{g,p})$$

Where the expected count value $\mu_{n,p}$ is given by the following log-linear model

$$\log \mu_{g,p} = \beta_0 + d_p\beta_g^{\text{donor}} + o_p\beta_g^{\text{organ}} + c_p\beta_g^{\text{celltype}} + c_p o_p \beta_g^{\text{organ}\times\text{celltype}} + \log L_p$$

We estimated the log-fold change $\beta_g^{\text{organ}\times\text{celltype}}$ in expression in a given cell type for organ $\hat{o}$ using the quasi-likelihood method (*87*) implemented in the R package glmGamPoi (*85*).

We used the estimated logFC from the test on the control cell types to filter out genes where differential expression is driven by technical differences in tissue processing. In particular, we considered a gene to be significantly overexpressed in cell types $\hat{c}$ and organ $\hat{o}$ if it is significant in the test on $\hat{c}$ (FDR < 10% and logFC > 1) but not in the test on control cell types (FDR > 10% and logFC > 0). We provide the full results for the differential expression analysis between organs in mature T cells and monocytes in tables S2 and S4.

TCR analysis

Single-cell αβTCR sequencing data was mapped with cellranger-vdj (v.6.0.0). The output file *filtered_contig_annotations.csv* was used and analyzed with scirpy (v.0.6.0) (*88*).

Single-cell γδTCR sequencing data was mapped with cellranger-vdj (v.4.0.0). All contigs deemed high-quality were selected, and re-annotated with igblastn (v.1.17.1) against IMGT reference sequences (last downloaded: 01/08/2021), via a workflow provided in dandelion (v0.2.0) (*89*) (https://github.com/zktuong/dandelion). The workflow runs igblastn with the following parameters: minimum D gene nucleotide match = 9, V gene e-value cutoff = 10^{-4}. It also reannotates D and J genes separately using blastn with the following parameters: dust = "no", word size (J = 7; D = 9), e-value cutoff (J = 10^{-4}; D = 10^{-3}). igblastn outputs were parsed into AIRR format with change-o scripts (*90*). The output file *all_contig_dandelion.tsv* was used and analyzed with scirpy (v0.6.0).

We determined productive TCR chain pairing status with *scirpy.tl.chain_qc()* function. For TCR usage PCA and clonotype analysis, cells with orphan VDJ or orphan VJ were filtered out so that each cell has at least one paired TCR. For clonotype analysis, only mature T cells were included to look at clonotype sharing. Clonotypes were determined using *scirpy.pp.ir_neighbors()* and *scirpy.tl.define_clonotypes()* functions with the CDR3 nucleotide sequence identity from both TCR chains as a metric.

Two samples from F67, F67_TH_CD137_FCAImmP7851896 and F67_TH_MAIT_FCAImmP7851897 were excluded from all downstream TCR analysis as they were sorted for specific T cell subpopulations, instead of the CD45 sorting in all other donor samples, and inclusion might result in biased TCR sampling within this donor.

BCR analysis

Single-cell BCR data was initially processed with cellranger-vdj (v.6.0.0). BCR contigs contained in *all_contigs.fasta* and *all_contig_annotations.csv* were then processed as follows: i) re-annotated with igblastn as per above; ii) re-annotated heavy-chain constant region calls using blastn (v.2.12.0+) against curated sequences from CH1 regions of respective isotype class; and iii) heavy-chain v-gene allele correction using tigger (v1.0.0) (*101*). Contigs were then filtered for basic quality control as described previously (*91*). Briefly, the following would lead to removal of contigs from further analysis: i) contigs were annotated with mismatched V, D, J, or constant gene calls not from the same locus; ii) multiple heavy-chain contigs. Exceptions to this would be when a) contigs were assessed to have identical V(D)J sequences but assigned as a different contig by cellranger-vdj (due to difference in non-V(D)J elements), b) when UMI count differences were large in which case the contig with the highest UMI count is retained, and c) if only IgM and IgD were both assigned to a cell; iii) only light-chain contigs in a cell; iv) multiple light-chain contigs in a cell. These were performed using dandelion (*89*) singularity container (v.0.2.0). BCR mutation frequencies were obtained using the *observedMutations* function in shazam (v.1.0.2) (*90*) with default settings (mutation counts for the different regions and mutation types were combined and returned as one frequency value per contig). Mutation rates per cell were averaged across contigs if multiple combinations of productive BCRs pairings were found in a single cell.

BCR clonotypes were determined with *dandelion.tl.find_clones()* function, based on the following criteria for both heavy-chain and light-chain contigs: (1) identical V and J gene usage, (2) identical junctional CDR3 amino acid length, and (3) at least 85% amino acid sequence similarity at the CDR3 junction (based on hamming distance). This strategy was chosen instead of using exact CDR3 nucleotide sequence identity to account for possible somatic hypermutations that happen within the same B cell clone.

B cell activation scoring

Gene Ontology B Cell Activation gene list was downloaded from Gene Set Enrichment Analysis website (http://www.gsea-msigdb.org/gsea/msigdb/genesets.jsp). Cells were scored according to expression values of all genes in this gene list apart from three genes that were not present in the dataset (*MIR17HG, MIR185, MIR19A*) using *scanpy.tl.score_genes()* function.

Transcription factor activity inference

We used the DoRothEA Python package (v.1.0.5) (*91*) to infer TF activities in B1 and mature B cells. Human regulons with confidence level A, B and C in DoRothEA database were utilized, and TF activities were inferred in each cell using *dorothea.run(adata, regulons, center=True,*

num_perm=100, norm=True, scale=True, use_raw=False, min_size=5, use_hvg=False) function. TFs that had higher activities (positive "meanchange") in B1 cells were then ranked according to their adjusted *P*-values and only top 25 TFs are shown in fig. S26F.

Cell–cell interaction analysis

We used the CellPhoneDB Python package (v.3.0) (*92*, *93*) to infer cell–cell interactions. The scRNA-seq dataset was split by organ and cell types with fewer than 20 cells in a given organ were filtered out. CellPhoneDB was run separately to infer cell–cell interactions in each organ, using default parameters. We used *P*-values from the permutation test (*pvalues.txt* output from CellPhoneDB), as well as the average expressions ("means") of the ligand and receptor within their corresponding cell types (*means.txt* output from CellPhoneDB). To explore cell–cell interactions between B cell progenitors and colocalizing cell types (fig. S24D), we aggregated the interactions predicted between each colocalizing cell type (e.g., ILC3 and different subtypes of B cell progenitors (pre-pro B, pro B, late pro B, large pre B and small pre B cells)), by averaging the means and using the minimum of the *P*-values. We then filtered for the ligand-receptor pairs that were significant (P<0.05) across all three organs of liver, spleen, and thymus, and ranked by the maximum aggregated means. Only the top 60 ligand-receptor pairs are shown in fig. S24C.

Query-to-reference mapping

We mapped query data to our prenatal data embeddings using online update of the scVI models following the scArches method (*15*), as implemented in the *scvi-tools* package (*80*). The model was trained for 200 epochs and setting *weight_decay = 0*, to ensure that the latent representation of the reference cells remained exactly the same. Reference genes missing in the query were set to 0, as recommended in (*15*). To generate a joint embedding of query and reference cells, we concatenated the latent dimensions learnt for query cells to the latent dimensions used for the reference embedding and computed the KNN graph and UMAP as described above. To assess that the mapping to the developmental reference conserves biological variation while minimising technical variation in the query data, we compared query cell type labels and batch labels with clusters obtained from Leiden clustering on the learnt latent dimensions, using the Normalized Mutual Information score (see fig. S33 for mapping of adult query data).

Annotation prediction using CellTypist

We used CellTypist v.0.1.9 Python package (*21*) to perform annotation prediction with logistic regression models. For prediction on cycling B cells, the rest of the non-progenitor B cells, including immature B, mature B, B1 and plasma B cells were used as training dataset. Default parameters were used for model building and prediction was made without majority voting for accurate enumeration of predicted B cell subtypes within cycling B cells.

Comparison with human adult immune cells

Single-cell RNA-seq data from adult immune cells was generated and preprocessed as described (*21*). The dataset including cell type annotations were provided by the authors. We mapped 264,929 adult lymphoid cells to the lymphoid embeddings of our developmental dataset and 54,047 adult myeloid cells to our myeloid embedding.

In order to use cell annotations in our developmental dataset to predict adult cell types in the joint embedding, for each adult cell c we identified its k nearest prenatal cell neighbors (N_c) (k=50), and calculated the probability of assigning a label y to adult cell c as

$$\Pr(Y = y \mid X = c, N_c) = \frac{1}{k}\sum_{i=1}^{k} I(y^{(i)} = y)$$

where $y^{(i)}$ is the label of the *i*th nearest neighbor and *I* is the binary indicator function. To label each cell we calculate $\hat{y}_c$ as follows

$$\hat{y}_c = \text{argmax}_y \Pr(Y = y \mid X = c, N_c)$$

and label c as $\hat{y}_c$ if $\Pr(Y = y \mid X = c, N_c) > 0.8$, otherwise c is labeled as "low confidence".

To quantify similarity of adult cells to prenatal cells (fig. S12C, fig. S17C), for each adult cell c, we calculated its similarity to prenatal cells labeled as $\hat{y}_c$ taking the Euclidean distance in the joint embedding, weighted by a Gaussian kernel following the approach described in (*15*).

Blood and immune cell progenitors scRNA-seq data analysis

For the cell fate prediction analysis shown in fig. S20, C and D, we used the Palantir method as implemented in CellRank (*94*, *95*). Briefly, from the scVI embedding on all immune cells (fig. S20A) we selected cells belonging to progenitor populations and computed a KNN graph on scVI latent dimensions on these cells (k=30). Then transition probabilities were calculated using the *ConnectivityKernel* in the *cellrank* package. We computed coarse-grained macrostates with Generalized Perron Cluster Cluster Analysis, setting the number of macrostates to the number of annotated progenitor cell populations. We manually set the four target terminal states for each lineage (small pre B cells, DN(Q) T cells, early MKs, and promonocytes) and computed the probability of each cell to transition to one of the four terminal states. The fate simplex visualization in fig. S20, C and D, was generated using the function *cellrank.pl.circular_projection*.

Artificial thymic organoids scRNA-seq data analysis

Raw scRNA-seq reads were mapped with cellranger 3.0.2 with combined human reference of GRCh38.93 and mouse reference of mm10-3.1.0. Low quality cells were filtered out (minimum number of reads = 2000, minimum number of genes = 500, minimum Scrublet (*77*) doublet detection score <0.4). Cells where the percentage of counts from human genes was <90% were considered as mouse cells and excluded from downstream analysis. Cells were assigned to different cell lines (Kolf, Fiaj) using genotype prediction with souporcell (v.2.4.0) (*78*). We performed batch correction to minimize the differences between cells from different cell lines using scVI and clustered cells using the Leiden algorithm on the latent embedding as described above. We used CellTypist v.0.1.9 Python package (*21*) to perform annotation prediction with logistic regression using the whole in vivo scRNA-seq developmental dataset for training. Stochastic gradient descent was used (setting *use_SGD = True*), and maximum iterations were set to 1000 in model building to reduce the run time. Predicted annotations were then aggregated using a majority voting scheme with *majority_voting = True*, *over_clustering = leiden* in CellTypist prediction to refine cell identities within Leiden clusters. For the in vivo-to-in vitro similarity analysis in fig. S29D, we mapped in vitro cells to the scVI model of lymphoid cells as described above. For each cell in the in vitro dataset we calculated the Euclidean distance (weighted by a Gaussian kernel as described above) to the closest in vivo cell from each in vivo cell population.

Spatial data analysis

Spatial transcriptomics data was mapped using spaceranger v.1.2.1. In parallel, we used a custom image-processing script to identify regions overlapping tissues and retained for analysis the intersection of the tissue spots identified by this pipeline and by tissue calling by spaceranger. To map cell types identified by scRNA-seq in the profiled spatial transcriptomics slides, we used the cell2location method (*16*). Briefly, this consists of two steps. First, for each of the profiled organs we trained a negative binomial regression model to estimate reference transcriptomic profiles for all the cell types profiled with scRNA-seq in the organ. Here we excluded very lowly expressed genes using a recommended filtering strategy (*16*). Cell types where fewer than 20 cells were profiled in the organ of interest and cell types labeled as low-quality cells were excluded from the reference. For the analysis of unconventional T cell localization in thymus (fig. S27C), we trained a reference adding all the prenatal thymic epithelial cells from a thymus cell atlas (*7*) (data was downloaded from Zenodo (*96*)). Next, we estimated the abundance of cell types in the spatial transcriptomics slides using reference transcriptomic profiles of different cell types. All slides representing a given organ were analyzed jointly. Cell2location requires the choice of two hyperparameters: (1) expected cell abundance (*N_cells_per_location = 30*) which was determined by counting average number of nuclei in the histology images corresponding to Visium spots; (2) regularization strength of detection efficiency effect (*detection_alpha = 20*) was used at the low setting to account for variations in RNA detection sensitivity across different spots of Visium slides. The training was stopped after the cell2location model converged, the number of training iterations was 50,000 for thymus, liver, spleen and 30,000 for gut. All other parameters were used at default settings. Cell2location estimates the posterior distribution of cell abundance of every cell type in every spot. Posterior distribution was summarized as 5% quantile, representing the value of cell abundance that the model has high confidence in, and thus incorporating the uncertainty in the estimate into values reported in the paper and used for downstream colocalization analysis.

To identify microenvironments of colocalizing cell types, we used non-negative matrix factorization (NMF) on the matrix of estimated cell type abundances X of dimensions $n \times c$, where n is the total number of spots in the Visium slides and c is the number of cell types in the reference. We decomposed the estimated cell type abundances X as $X = WZ^{\top}$, where Z is a $n \times d$ matrix of latent factor values for each spot and W is a $d \times c$ matrix representing the fraction of abundance of each cell type attributed to each latent factor. Here latent factors correspond to tissue microenvironments defined by a set of colocalized cell types. We use the NMF implementation in scikit-learn (*81*), with the wrapper in the cell2location package, setting the number of factors d = 10. For downstream analysis, we excluded cell types where the 99% quantile of cell abundance across locations in every slide from the same organ was always below the detection threshold of 0.15. Unless otherwise specified, we consider a cell type to be part of a microenvironment if the cell type fraction was over 0.2.

For analysis of mature T cell localization in the thymic medulla (fig. S27, D and E), we retained factors where the sum of the cell type fractions for mature T cells ($CD4^+$ T, $CD8^+$ T, Treg, type 1 innate T, type 3 innate T, and CD8AA) was above 0.8. We assigned spots to the inner medulla or cortico-medullary microenvironment if the factor value in the spot was above the 90% quantile of all values in the slide. To annotate histological regions in the thymus, we extracted image features from the high resolution images of H&E staining using the python package *squidpy*

(v1.1.2) (*97*) (running *sq.im.calculate_image_features*, with parameters *features = "histogram"*, *spot_scale = 1*, *mask_circle = True*). We scaled and mean centered image feature matrix, and performed Leiden clustering on the first 10 principal components. We manually annotated spot clusters overlapping the thymic cortex and medulla. To define the cortico-medullary junction (CMJ), we detected the spatial neighbors of each spot in medulla or cortex (using the function *squidpy.gr.spatial_neighbours*, with parameters *n_rings = 1*, *coord_type = "grid"*, *n_neighs = 6*). We then labeled a spot as CMJ if it had at least five neighbors (to exclude tissue borders), and if the neighbors included spots from both medulla and cortex regions. For each spot we calculated the distance to the CMJ as the Euclidean distance between the spatial coordinates of the spot and the closest spot annotated as CMJ.

B1 functional validation experiment

Spleens were isolated from two donors, F144 (17 pcw) and F145 (15 pcw). A single-cell suspension was obtained following the protocol described in the "Tissue acquisition and processing" section. Cells were then cryopreserved with 90% FBS and 10% DMSO (Sigma-Aldrich). On the day of the ELISpot experiment, cells were thawed and stained with anti-CD3 (BV510 anti-human CD3 antibody, BD Biosciences, 563109), anti-CD20 (FITC anti-human CD20 antibody, Biolegend, 302303), anti-CD43 (PE anti-human CD43 antibody, BD Biosciences, 560199), anti-CD27 (APC/Cy7 anti-human CD27 antibody, Biolegend, 356424), anti-CD38 (BV711 anti-human CD38 antibody, Biolegend, 303527), anti-CCR10 (APC anti-human CCR10 antibody, Biolegend, 341505) antibodies and DAPI together with control peripheral blood mononuclear cells (PBMC; Stemcell Technologies). B cells were gated as singlet $DAPI^{-}CD3^{-}CD20^{+}$ cells. Plasma cells should generally be $CD20^{lo}$ and therefore not included. To further exclude plasma cell contamination, we also gated out the top 1% of B cells expressing the highest level of CD38. The rest of the B cells were then sorted into four fractions: $CCR10^{hi}$, $CCR10^{lo}CD27^{+}CD43^{+}$, $CCR10^{lo}CD27^{-}CD43^{+}$, and $CCR10^{lo}CD27^{-}CD43^{-}$. CD27 and CD43 gates were chosen based on fluorescence minus one (FMO) controls. The cells were sorted into RPMI supplemented with 10% FBS, penicillin–streptomycin (Gibco) and glutamax (Thermo Fisher Scientific).

The ELISpot experiment was performed with Human IgM $ELISpot^{BASIC}$ kit (ALP) from Mabtech AB. Post sorting, 7000-8000 cells were added into ELISpot plate pre-coated with anti-IgM antibody following manufacturer's instructions and incubated in a 37°C humidified incubator with 5% CO_2 for 22 hours. The plate was then washed and incubated with biotinylated anti-IgM for 2 hours at room temperature, followed by 1-hour incubation of streptavidin-ALP. The colored spots were developed with 15-min incubation of BCIP/NBT substrate solution (Thermo Fisher Scientific). Five rounds of washing were performed between each step of incubation as per manufacturer's instructions. After the colored spots appeared clearly, the reaction was then stopped by rinsing under running tap water for 5 min. Spots were counted with the AID ELISpot reader and iSpot software version 4.

In addition, we performed scRNA-seq of the sorted B cell fractions on a different donor (F149, 18 pcw fetal spleen), using the same gating strategy to further confirm the identity of sorted cells. The scRNA-seq data was preprocessed with scVI as above. Cell annotations were predicted using CellTypist v.0.1.9 (*21*), initially with our original developmental dataset as the training data to find the non-B cells, then with only the non-cycling mature B cells from the developmental dataset as the training data to map the B cells in the sorted data. Default parameters were used for model building and prediction was made with majority voting.

Supplementary Figures

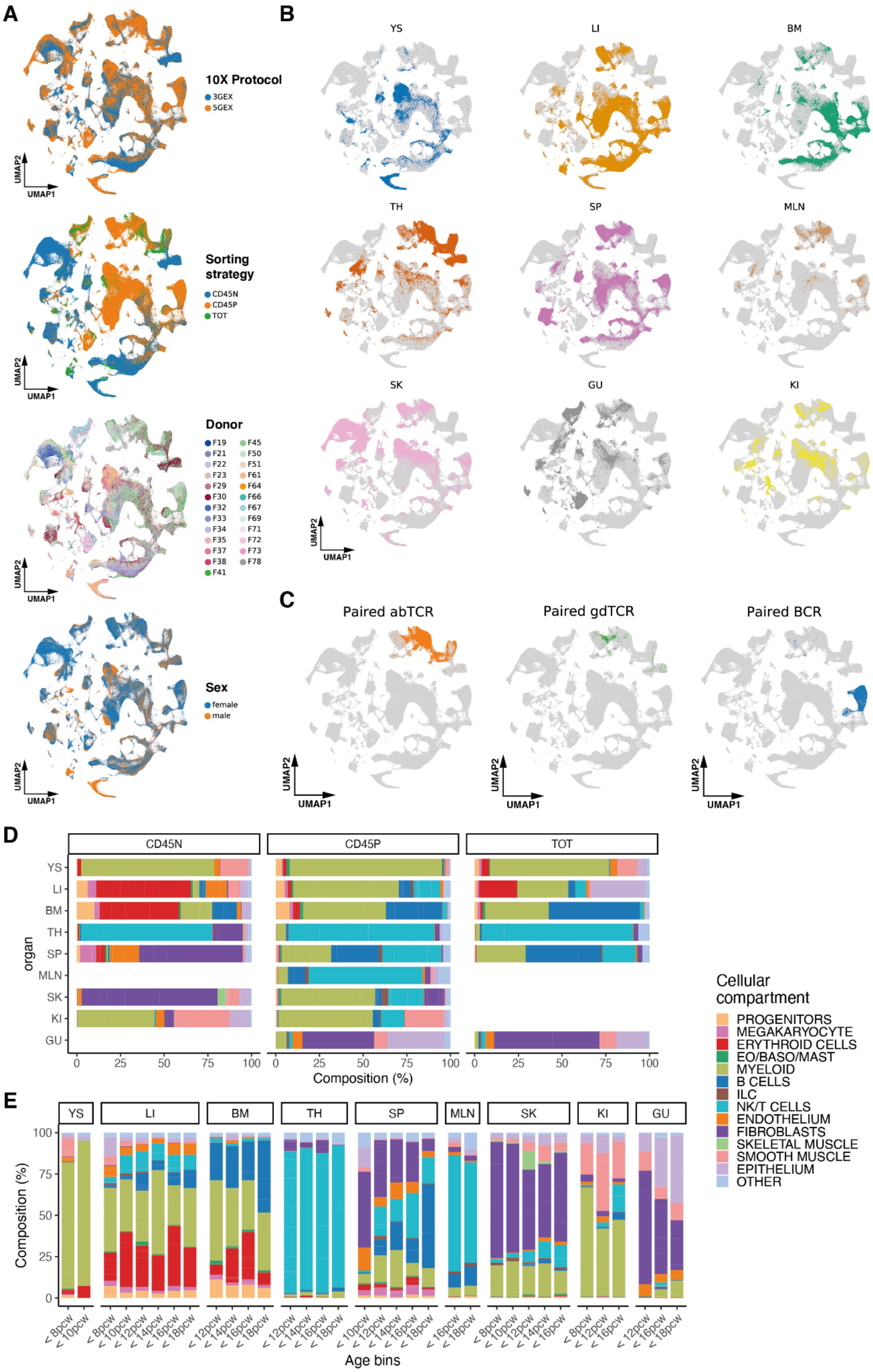
A
10X Protocol
3GEX
5GEX
Sorting strategy
CD45N
CD45P
TOT
Donor
F19
F21
F22
F23
F29
F30
F32
F33
F34
F35
F37
F38
F41
F45
F50
F51
F61
F64
F66
F67
F69
F71
F72
F73
F78
Sex
female
male
UMAP1
UMAP2
B
YS
LI
BM
TH
SP
MLN
SK
GU
KI
C
Paired abTCR
Paired gdTCR
Paired BCR
D
CD45N
CD45P
TOT
organ
Composition (%)
E
Composition (%)
Age bins
Cellular compartment
PROGENITORS
MEGAKARYOCYTE
ERYTHROID CELLS
EO/BASO/MAST
MYELOID
B CELLS
ILC
NK/T CELLS
ENDOTHELIUM
FIBROBLASTS
SKELETAL MUSCLE
SMOOTH MUSCLE
EPITHELIUM
OTHER

fig. S1: Characterization of cross-organ developmental scRNA-seq atlas. (**A**) Sample characteristics in integrated atlas. UMAP embeddings (as in Fig. 1C) of scRNA-seq profiles colored by (top to bottom): 10X chemistry protocol, FACS protocol (CD45P: $CD45^{+}$; CD45N: $CD45^{-}$; TOT: unsorted), donor ID, sex of donor. (**B**) Distribution of cells from different organs in integrated atlas. UMAP embeddings (as in Fig. 1C) of scRNA-seq profiles, highlighting cells from each organ. (**C**) UMAP embeddings (as in Fig. 1C) of scRNA-seq profiles, highlighting cells for which paired αβTCR, γδTCR or BCR sequences were detected. (**D**) Percentage of cells of each broad type in each organ, stratified by FACS protocol. (**E**) Percentage of cells of each broad type in each gestational age group, stratified by organ. (YS: yolk sac; LI: liver; BM: bone marrow; TH: thymus; SP: spleen; SK: skin; GU: gut; KI: kidney).

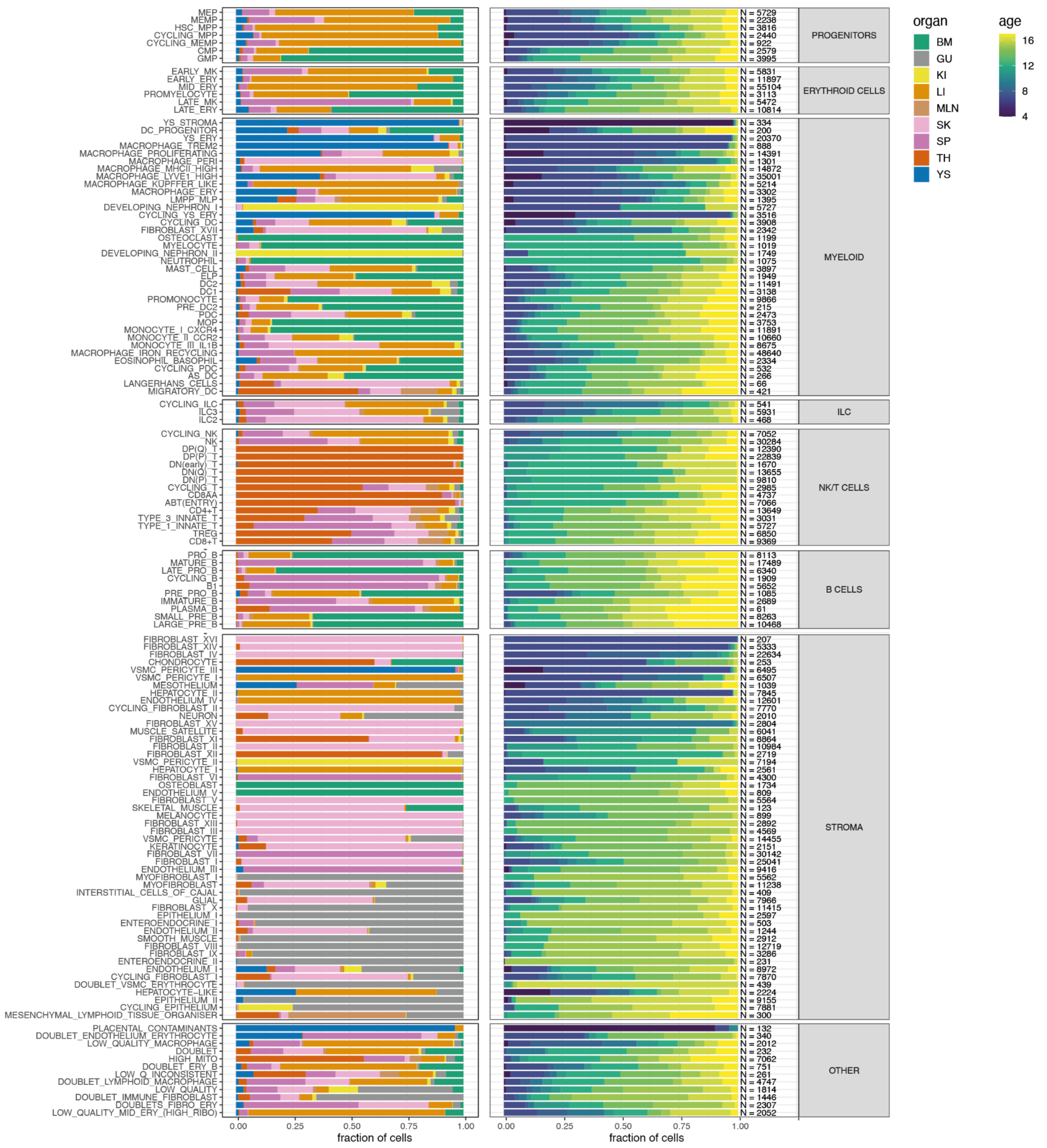

fig. S2: Distribution across organs (left) and gestational age (pcw, right) of annotated cell populations. Cell populations are grouped according to broad population annotations. The category "Other" denotes clusters annotated as low-quality cells. N indicates the total number of cells across the dataset for each annotation. (YS: yolk sac; LI: liver; BM: bone marrow; TH: thymus; SP: spleen; SK: skin; GU: gut; KI: kidney).

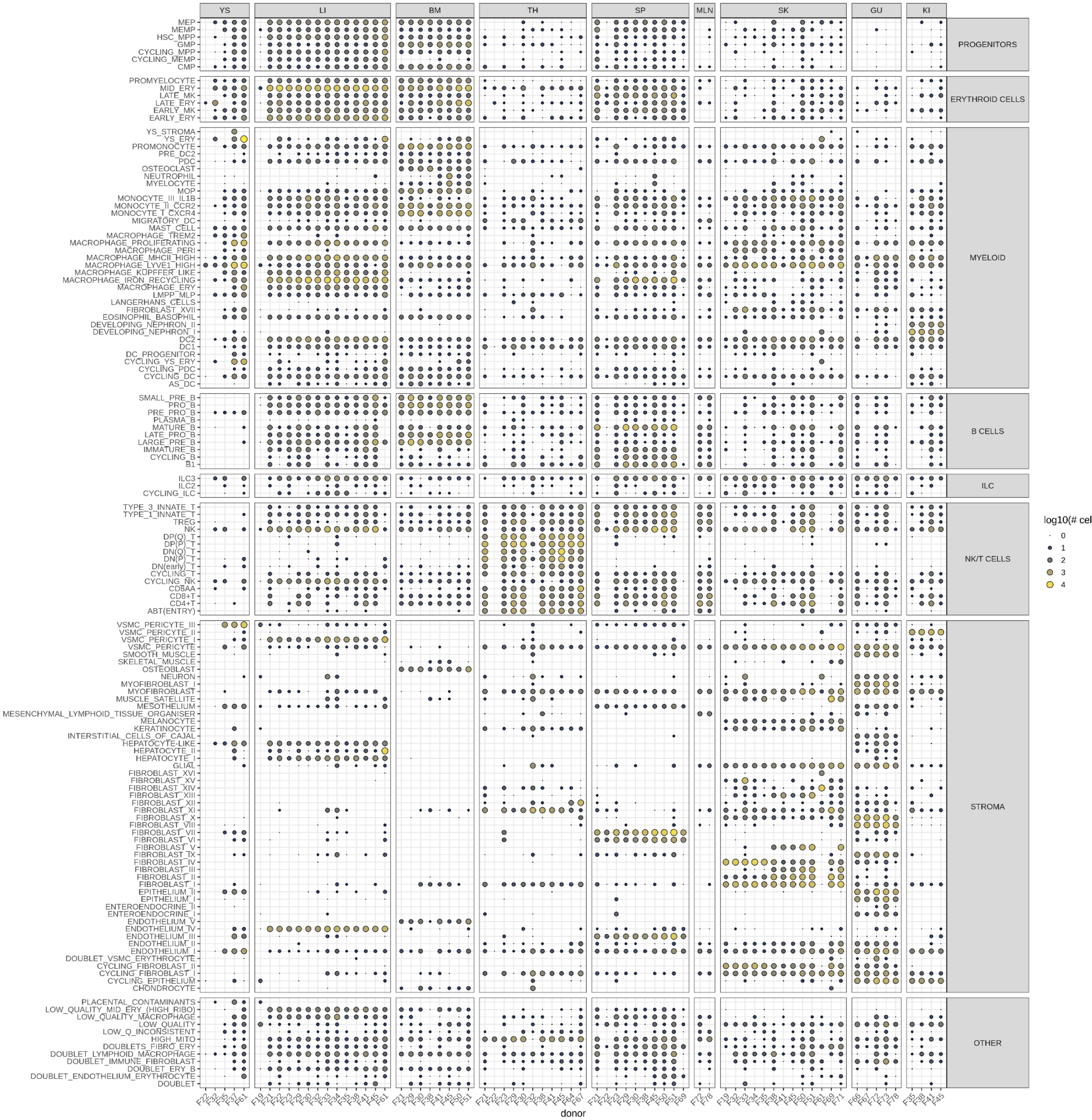

fig. S3: Consistency across donors of annotated cell populations. Dot size and color are proportional to the number of cells of the annotated population from each donor and organ. Cell populations are grouped according to broad population annotations. The category "Other" denotes clusters annotated as low-quality cells.

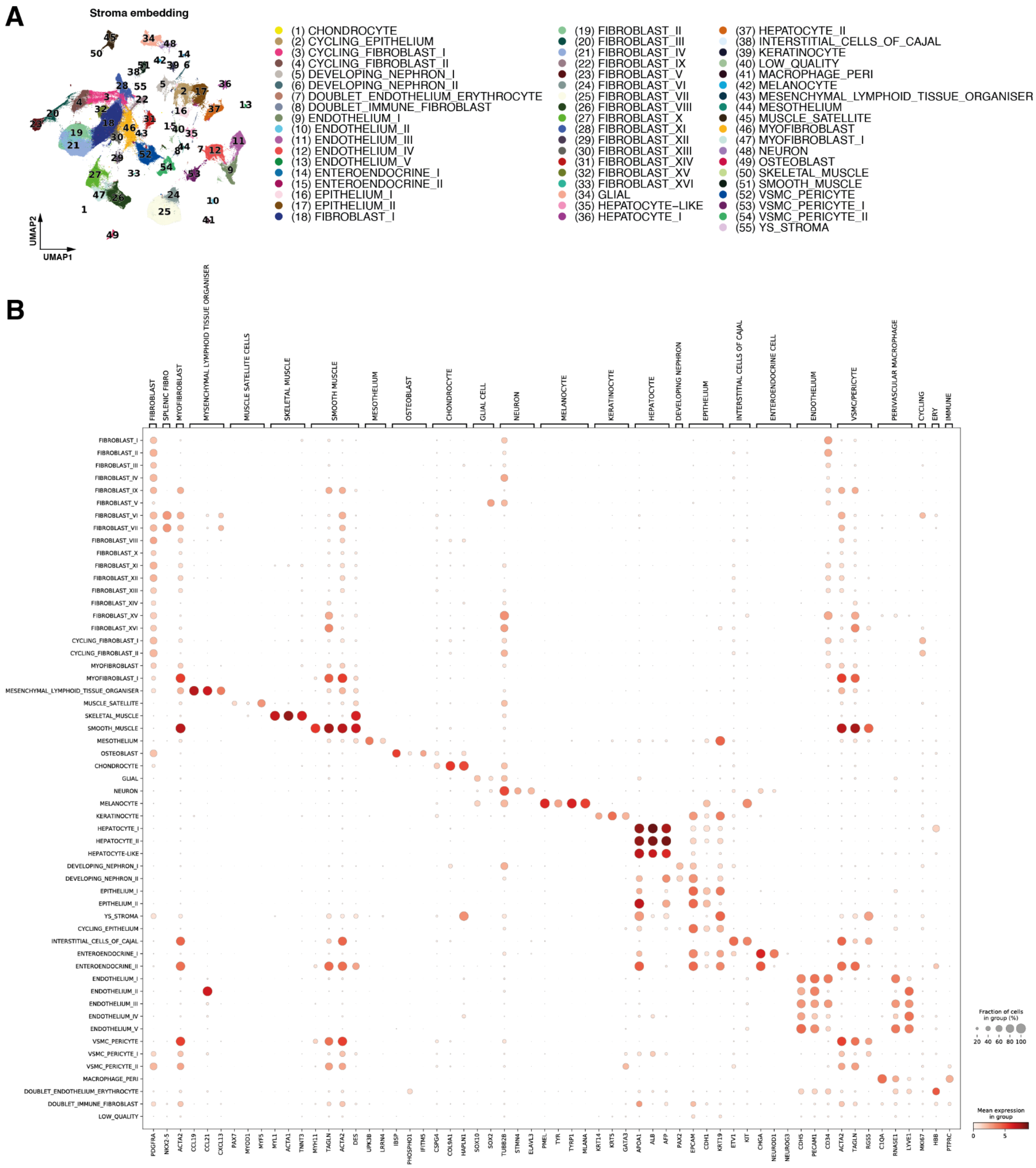
A
Stroma embedding
UMAP1
UMAP2
(1) CHONDROCYTE
(2) CYCLING_EPITHELIUM
(3) CYCLING_FIBROBLAST_I
(4) CYCLING_FIBROBLAST_II
(5) DEVELOPING_NEPHRON_I
(6) DEVELOPING_NEPHRON_II
(7) DOUBLET_ENDOTHELIUM_ERYTHROCYTE
(8) DOUBLET_IMMUNE_FIBROBLAST
(9) ENDOTHELIUM_I
(10) ENDOTHELIUM_II
(11) ENDOTHELIUM_III
(12) ENDOTHELIUM_IV
(13) ENDOTHELIUM_V
(14) ENTEROENDOCRINE_I
(15) ENTEROENDOCRINE_II
(16) EPITHELIUM_I
(17) EPITHELIUM_II
(18) FIBROBLAST_I
(19) FIBROBLAST_II
(20) FIBROBLAST_III
(21) FIBROBLAST_IV
(22) FIBROBLAST_IX
(23) FIBROBLAST_V
(24) FIBROBLAST_VI
(25) FIBROBLAST_VII
(26) FIBROBLAST_VIII
(27) FIBROBLAST_X
(28) FIBROBLAST_XI
(29) FIBROBLAST_XII
(30) FIBROBLAST_XIII
(31) FIBROBLAST_XIV
(32) FIBROBLAST_XV
(33) FIBROBLAST_XVI
(34) GLIAL
(35) HEPATOCYTE-LIKE
(36) HEPATOCYTE_I
(37) HEPATOCYTE_II
(38) INTERSTITIAL_CELLS_OF_CAJAL
(39) KERATINOCYTE
(40) LOW_QUALITY
(41) MACROPHAGE_PERI
(42) MELANOCYTE
(43) MESENCHYMAL_LYMPHOID_TISSUE_ORGANISER
(44) MESOTHELIUM
(45) MUSCLE_SATELLITE
(46) MYOFIBROBLAST
(47) MYOFIBROBLAST_I
(48) NEURON
(49) OSTEOBLAST
(50) SKELETAL_MUSCLE
(51) SMOOTH_MUSCLE
(52) VSMC_PERICYTE
(53) VSMC_PERICYTE_I
(54) VSMC_PERICYTE_II
(55) YS_STROMA
B
FIBROBLAST
SPLENIC FIBRO
MYOFIBROBLAST
MYSENCHYMAL LYMPHOID TISSUE ORGANISER
MUSCLE SATELLITE CELLS
SKELETAL MUSCLE
SMOOTH MUSCLE
MESOTHELIUM
OSTEOBLAST
CHONDROCYTE
GLIAL CELL
NEURON
MELANOCYTE
KERATINOCYTE
HEPATOCYTE
DEVELOPING NEPHRON
EPITHELIUM
INTERSTITIAL CELLS OF CAJAL
ENTEROENDOCRINE CELL
ENDOTHELIUM
VSMC/PERICYTE
PERIVASCULAR MACROPHAGE
CYCLING
ERY
IMMUNE
FIBROBLAST_I
FIBROBLAST_II
FIBROBLAST_III
FIBROBLAST_IV
FIBROBLAST_IX
FIBROBLAST_V
FIBROBLAST_VI
FIBROBLAST_VII
FIBROBLAST_VIII
FIBROBLAST_X
FIBROBLAST_XI
FIBROBLAST_XII
FIBROBLAST_XIII
FIBROBLAST_XIV
FIBROBLAST_XV
FIBROBLAST_XVI
CYCLING_FIBROBLAST_I
CYCLING_FIBROBLAST_II
MYOFIBROBLAST
MYOFIBROBLAST_I
MESENCHYMAL_LYMPHOID_TISSUE_ORGANISER
MUSCLE_SATELLITE
SKELETAL_MUSCLE
SMOOTH_MUSCLE
MESOTHELIUM
OSTEOBLAST
CHONDROCYTE
GLIAL
NEURON
MELANOCYTE
KERATINOCYTE
HEPATOCYTE_I
HEPATOCYTE_II
HEPATOCYTE-LIKE
DEVELOPING_NEPHRON_I
DEVELOPING_NEPHRON_II
EPITHELIUM_I
EPITHELIUM_II
YS_STROMA
CYCLING_EPITHELIUM
INTERSTITIAL_CELLS_OF_CAJAL
ENTEROENDOCRINE_I
ENTEROENDOCRINE_II
ENDOTHELIUM_I
ENDOTHELIUM_II
ENDOTHELIUM_III
ENDOTHELIUM_IV
ENDOTHELIUM_V
VSMC_PERICYTE
VSMC_PERICYTE_I
VSMC_PERICYTE_II
MACROPHAGE_PERI
DOUBLET_ENDOTHELIUM_ERYTHROCYTE
DOUBLET_IMMUNE_FIBROBLAST
LOW_QUALITY
PDGFRA
NKX2-5
ACTA2
CCL19
CCL21
CXCL13
PAX7
MYOD1
MYF5
MYL1
ACTA1
TNNT3
MYH11
TAGLN
ACTA2
DES
UPK3B
LRRN4
IBSP
PHOSPHO1
IFITM5
CSPG4
COL9A1
HAPLN1
SOX10
SOX2
TUBB2B
STMN4
ELAVL3
PMEL
TYR
TYRP1
MLANA
KRT14
KRT5
GATA3
APOA1
ALB
AFP
PAX2
EPCAM
CDH1
KRT19
ETV1
KIT
CHGA
NEUROD1
NEUROG3
CDH5
PECAM1
CD34
ACTA2
TAGLN
RGS5
C1QA
RNASE1
LYVE1
MKI67
HBB
PTPRC
Fraction of cells in group (%)
20 40 60 80 100
Mean expression in group
0 5

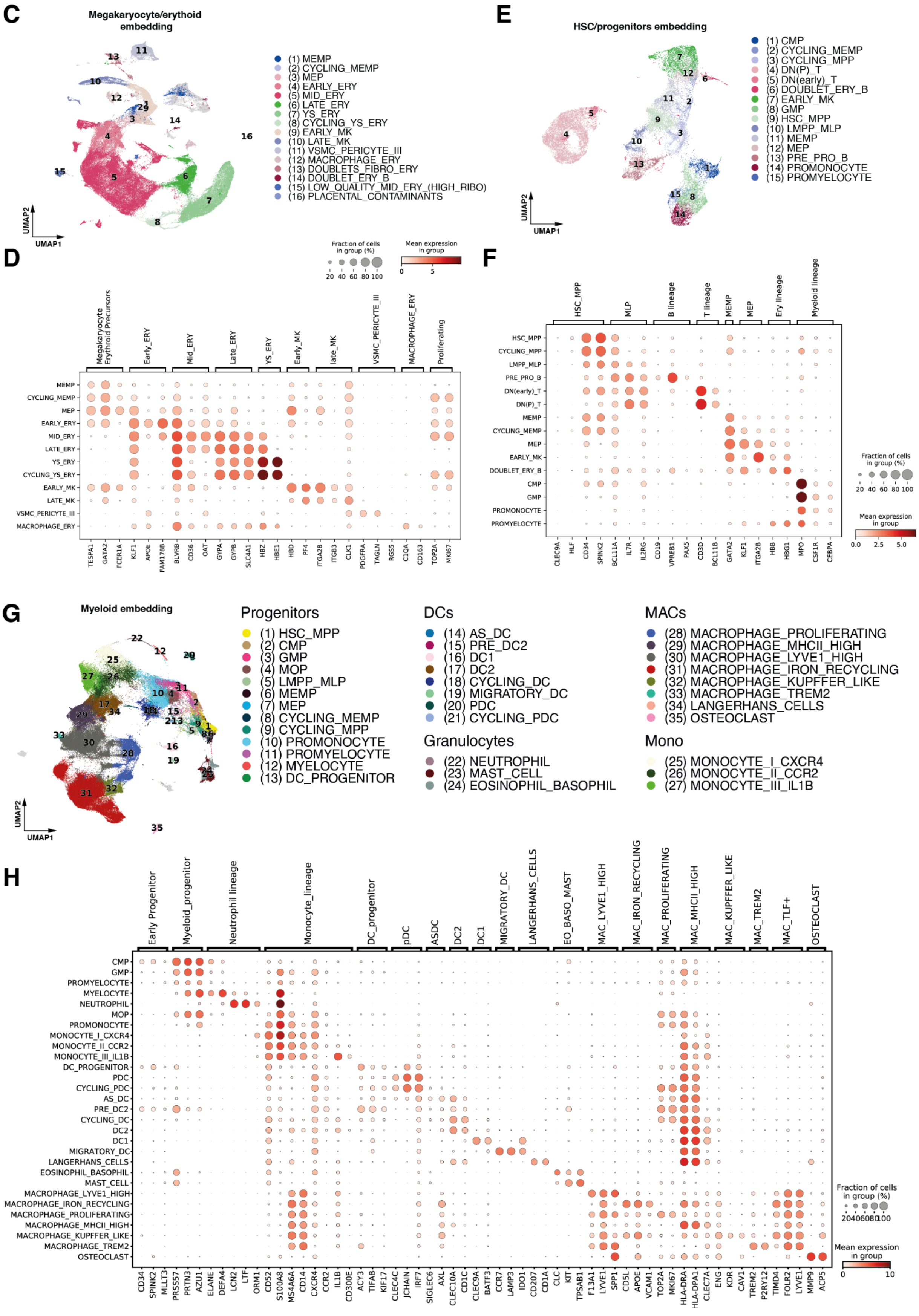
C
Megakaryocyte/erythoid embedding
(1) MEMP
(2) CYCLING_MEMP
(3) MEP
(4) EARLY_ERY
(5) MID_ERY
(6) LATE_ERY
(7) YS_ERY
(8) CYCLING_YS_ERY
(9) EARLY_MK
(10) LATE_MK
(11) VSMC_PERICYTE_III
(12) MACROPHAGE_ERY
(13) DOUBLETS_FIBRO_ERY
(14) DOUBLET_ERY_B
(15) LOW_QUALITY_MID_ERY_(HIGH_RIBO)
(16) PLACENTAL_CONTAMINANTS
UMAP1
UMAP2
E
HSC/progenitors embedding
(1) CMP
(2) CYCLING_MEMP
(3) CYCLING_MPP
(4) DN(P)_T
(5) DN(early)_T
(6) DOUBLET_ERY_B
(7) EARLY_MK
(8) GMP
(9) HSC_MPP
(10) LMPP_MLP
(11) MEMP
(12) MEP
(13) PRE_PRO_B
(14) PROMONOCYTE
(15) PROMYELOCYTE
D
Fraction of cells in group (%)
Mean expression in group
Megakaryocyte Erythroid Precursors
Early_ERY
Mid_ERY
Late_ERY
YS_ERY
Early_MK
late_MK
VSMC_PERICYTE_III
MACROPHAGE_ERY
Proliferating
F
HSC_MPP
MLP
B lineage
T lineage
MEMP
MEP
Ery lineage
Myeloid lineage
G
Myeloid embedding
Progenitors
(1) HSC_MPP
(2) CMP
(3) GMP
(4) MOP
(5) LMPP_MLP
(6) MEMP
(7) MEP
(8) CYCLING_MEMP
(9) CYCLING_MPP
(10) PROMONOCYTE
(11) PROMYELOCYTE
(12) MYELOCYTE
(13) DC_PROGENITOR
DCs
(14) AS_DC
(15) PRE_DC2
(16) DC1
(17) DC2
(18) CYCLING_DC
(19) MIGRATORY_DC
(20) PDC
(21) CYCLING_PDC
Granulocytes
(22) NEUTROPHIL
(23) MAST_CELL
(24) EOSINOPHIL_BASOPHIL
MACs
(28) MACROPHAGE_PROLIFERATING
(29) MACROPHAGE_MHCII_HIGH
(30) MACROPHAGE_LYVE1_HIGH
(31) MACROPHAGE_IRON_RECYCLING
(32) MACROPHAGE_KUPFFER_LIKE
(33) MACROPHAGE_TREM2
(34) LANGERHANS_CELLS
(35) OSTEOCLAST
Mono
(25) MONOCYTE_I_CXCR4
(26) MONOCYTE_II_CCR2
(27) MONOCYTE_III_IL1B
H
Early Progenitor
Myeloid_progenitor
Neutrophil lineage
Monocyte_lineage
DC_progenitor
pDC
ASDC
DC2
DC1
MIGRATORY_DC
LANGERHANS_CELLS
EO_BASO_MAST
MAC_LYVE1_HIGH
MAC_IRON_RECYCLING
MAC_PROLIFERATING
MAC_MHCII_HIGH
MAC_KUPFFER_LIKE
MAC_TREM2
MAC_TLF+
OSTEOCLAST
Fraction of cells in group (%)
Mean expression in group

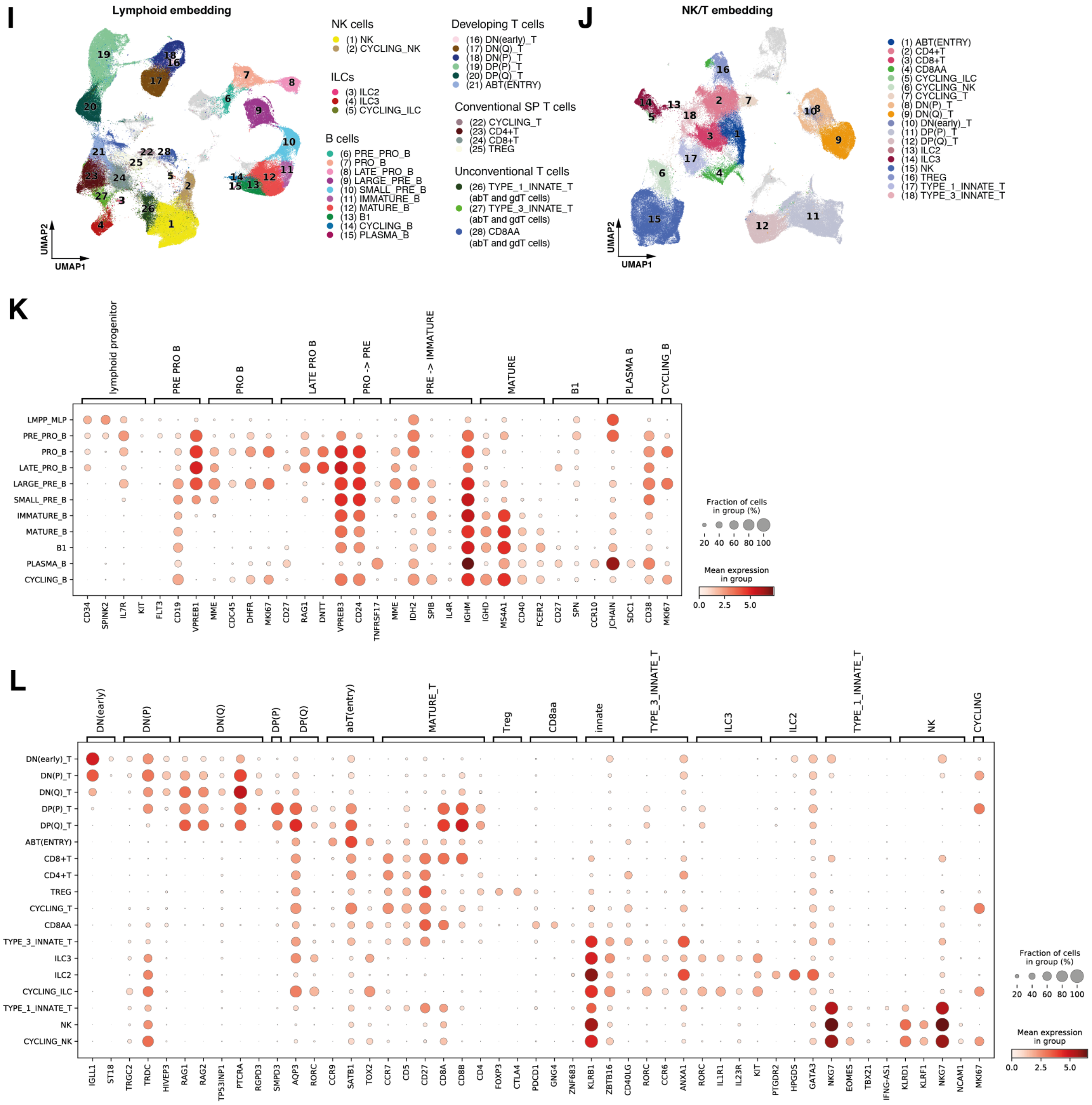

fig. S4: Cross-tissue annotation of hierarchical subsets of scRNA-seq integrated dataset. For each subset embedding generated through scVI, we show UMAP embeddings of cells colored by annotated cell populations and dot plots of mean expression (log-normalized counts, dot color) and fraction of expressing cells (dot size) of marker genes (columns) used for cell population annotation (rows). (**A** and **B**) Annotation of stromal cells. (**C** and **D**) Annotation of megakaryocyte and erythroid cells (cells in gray are progenitors annotated through embedding shown in (E)). (**E** and **F**) Annotation of hematopoietic and immune cell progenitors. (**G** and **H**) Annotation of myeloid cells (cells in gray are progenitors annotated through embedding shown in (E) or low-quality clusters) (**I** and **L**) Annotation of lymphoid cells (cells in gray are progenitors annotated through embedding shown in (E) or low-quality clusters). The embedding of all lymphoid cells is shown in (I), the embedding used for annotation of NK/T cells is visualized in (J). The dot plot for annotation of B cells is shown in (K), and the dot plot for annotation of T cells is displayed in (L).

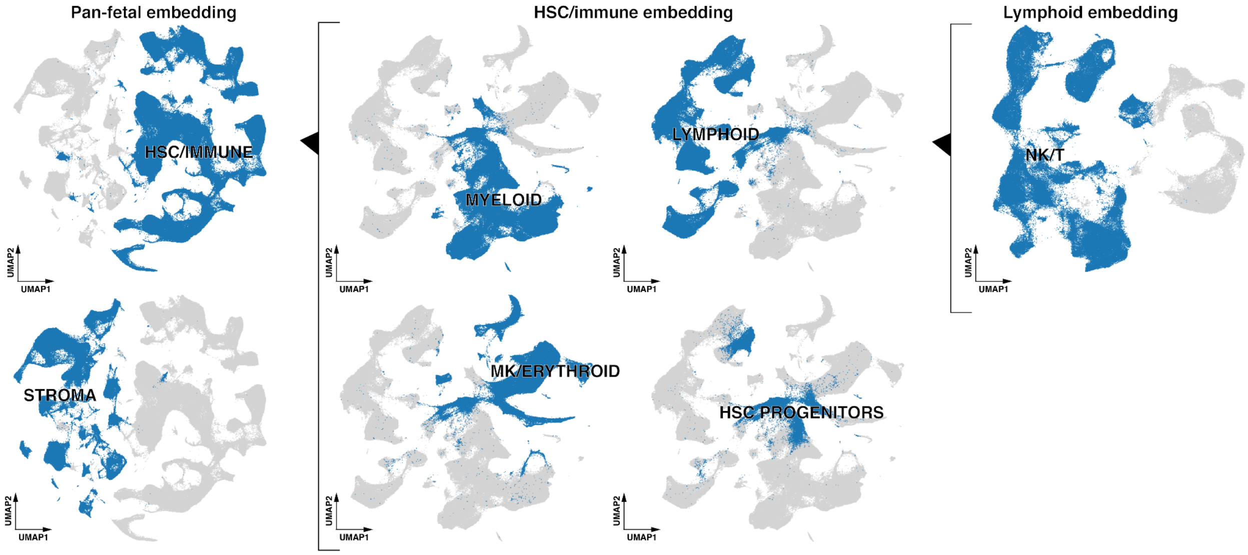

fig. S5: Overview of the hierarchical subsetting strategy used for annotation of fine immune subtypes. In each embedding, the cells that make up the data views in fig. S4 are highlighted in blue.

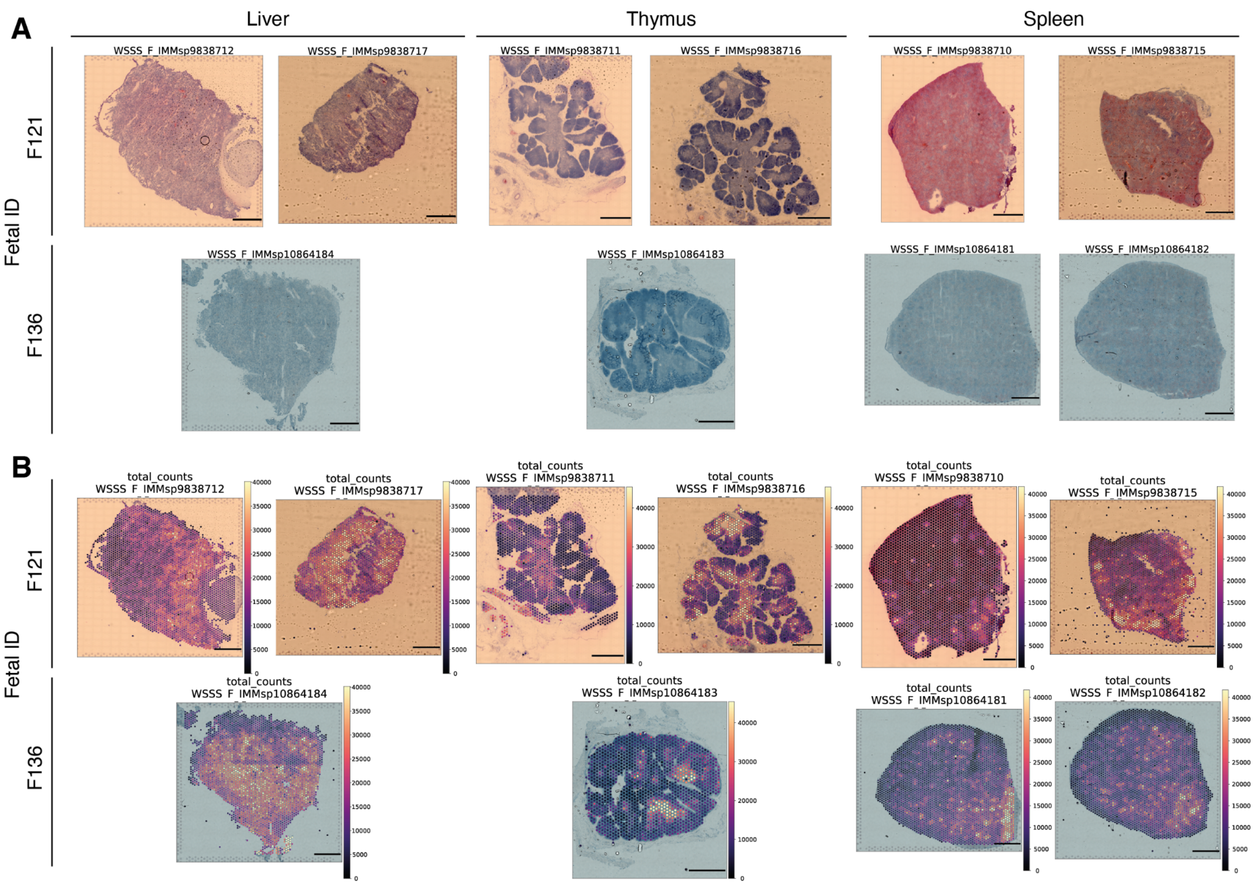

fig. S6: Experimental design and library QC for Visium 10X data (**A**) H&E staining of tissue slides processed for spatial transcriptomics with Visium 10X protocol. Slides are grouped by organ (columns) and embryo/fetus ID (rows). (**B**) Total RNA counts in analyzed tissue spots (scale bar: 1 mm).

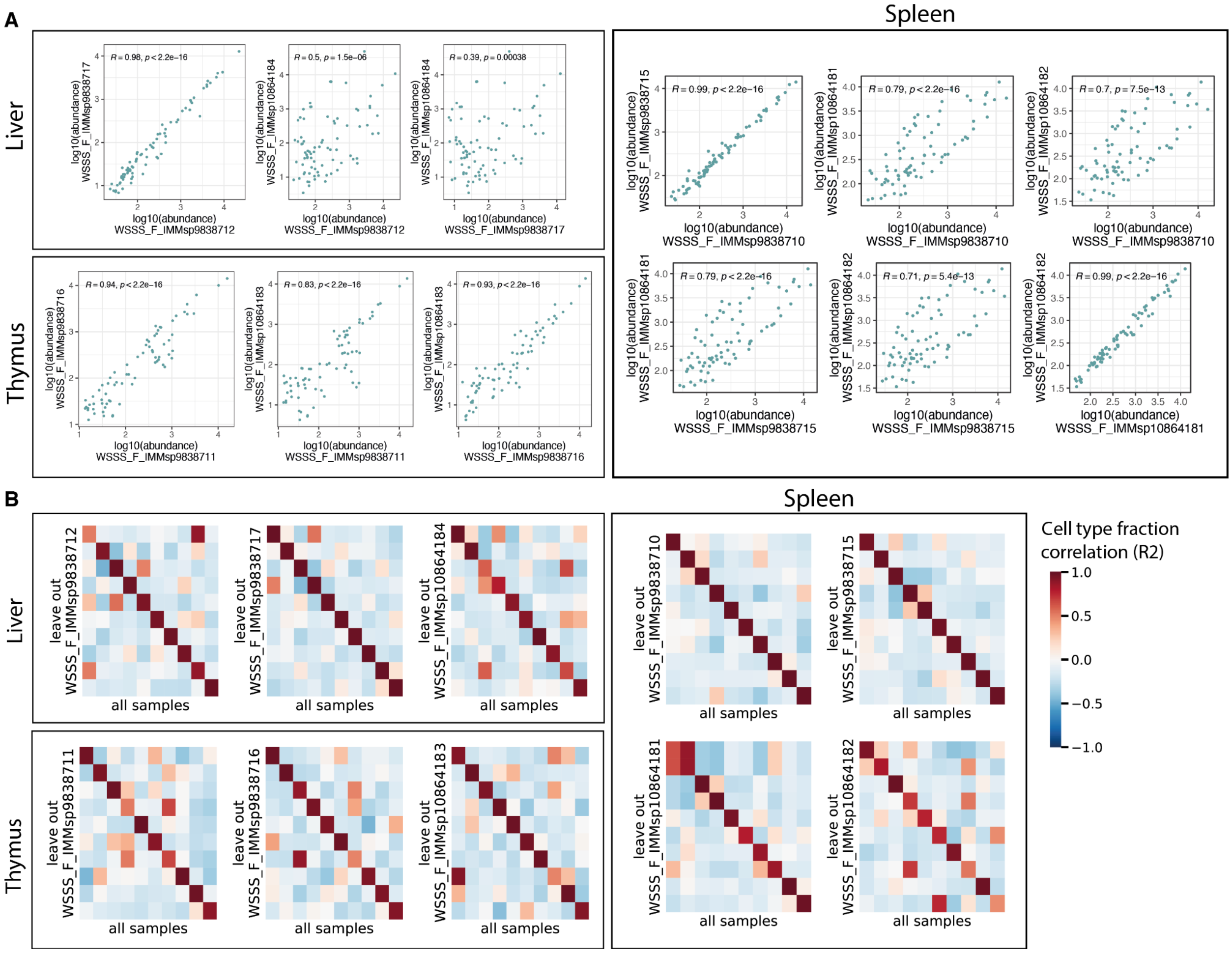

fig. S7: Robustness of spatial cell type abundance predictions with cell2location. (**A**) Analysis of robustness of cell type mapping with cell2location: for mapping on each organ we correlate the total abundance (in $\log_{10}$ scale) of each cell type (points) in different tissue slides from the same organ (biological replicates). The Pearson correlation coefficient and *P*-value for permutation test are reported. (**B**) Robustness of colocation predictions with NMF. We compare the NMF model learnt on all samples from the same organ with NMF models learnt leaving one sample out (as indicated by *y*-axis label). The color of the heatmaps represents the Pearson correlation between cell type fraction attributed to each factor in the compared models.

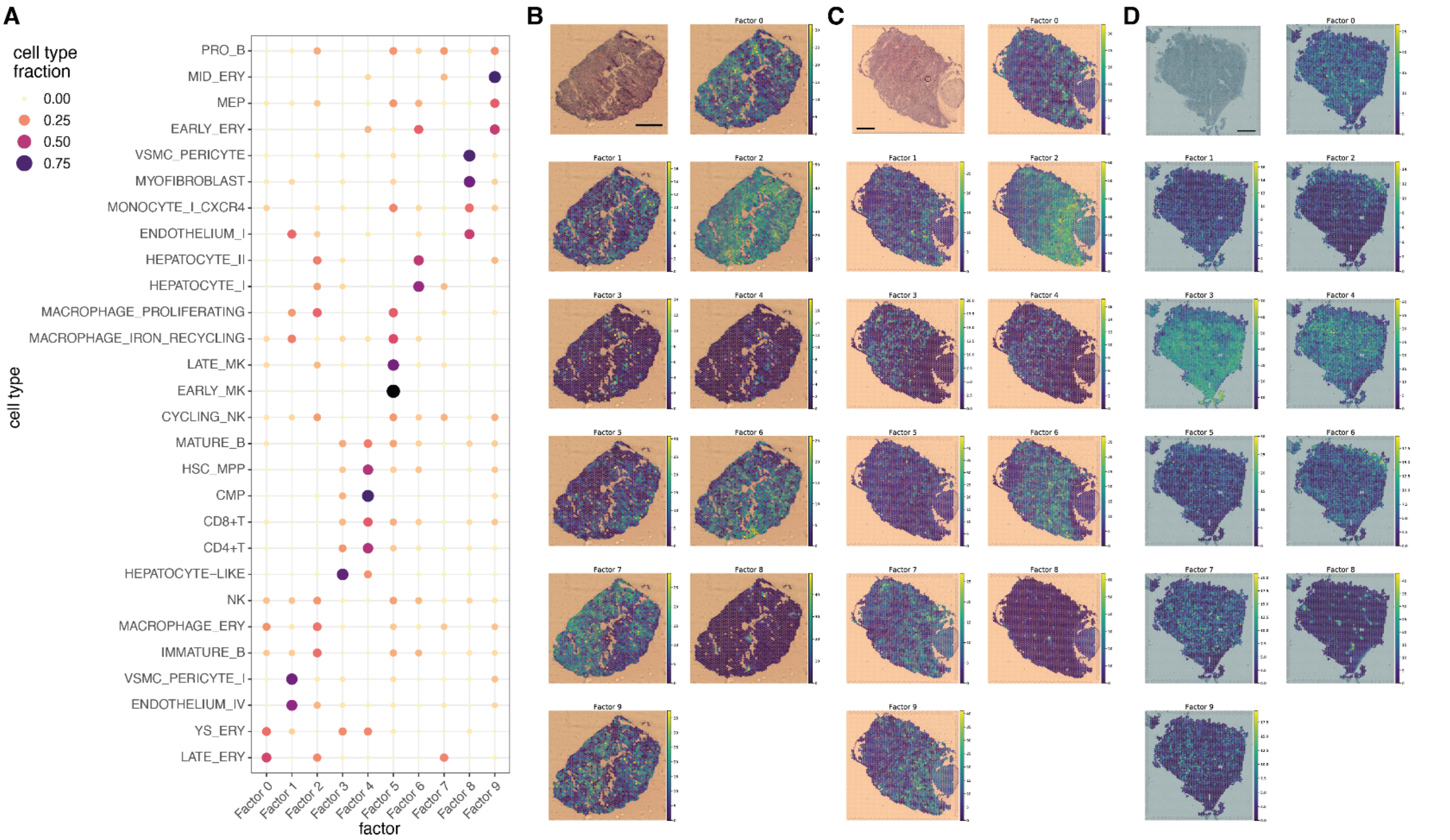

fig. S8: Cell type spatial microenvironments in the fetal liver detected by non-negative matrix factorization on spatial cell type abundances. (**A**) Dot plot of cell type contributions to latent factors (microenvironments) identified with non-negative matrix factorization of spatial cell type abundances estimated with cell2location. The color and the size of the dots represent the relative fraction of the cell population assigned to the factor. We exclude cell types where the value for the 99% quantile of cell abundance in all the slides from the same organ is always below the detection threshold of 0.15. (**B** to **D**) Spatial locations of microenvironments on liver slides, with the color representing the weighted contribution of each microenvironment to each spot. H&E staining images for each slide are shown for reference (scale bar: 1 mm).

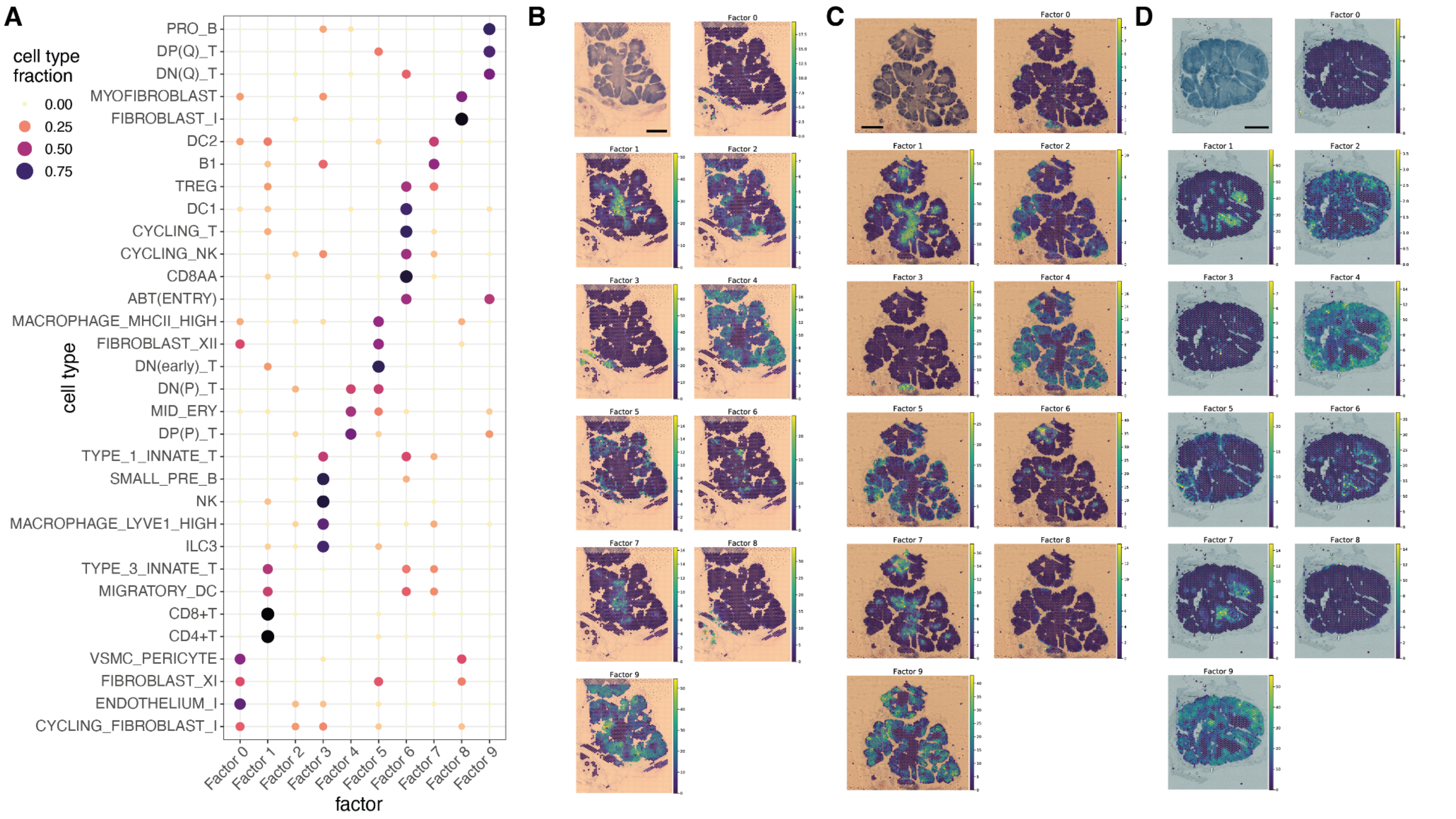

fig. S9: Cell type spatial microenvironments in the fetal thymus detected by non-negative matrix factorization on spatial abundances. (**A**) Dot plot of cell type contributions to latent factors (microenvironments) identified with non-negative matrix factorization of spatial cell type abundances estimated with cell2location. The color and the size of the dots represent the relative fraction of the cell population assigned to the factor. We exclude cell types where the value for the 99% quantile of cell abundance in all the slides from the same organ is always below the detection threshold of 0.15. (**B** to **D**) Spatial locations of microenvironments on thymus slides, with the color representing the weighted contribution of each microenvironment to each spot. H&E staining images for each slide are shown for reference (scale bar: 1 mm).

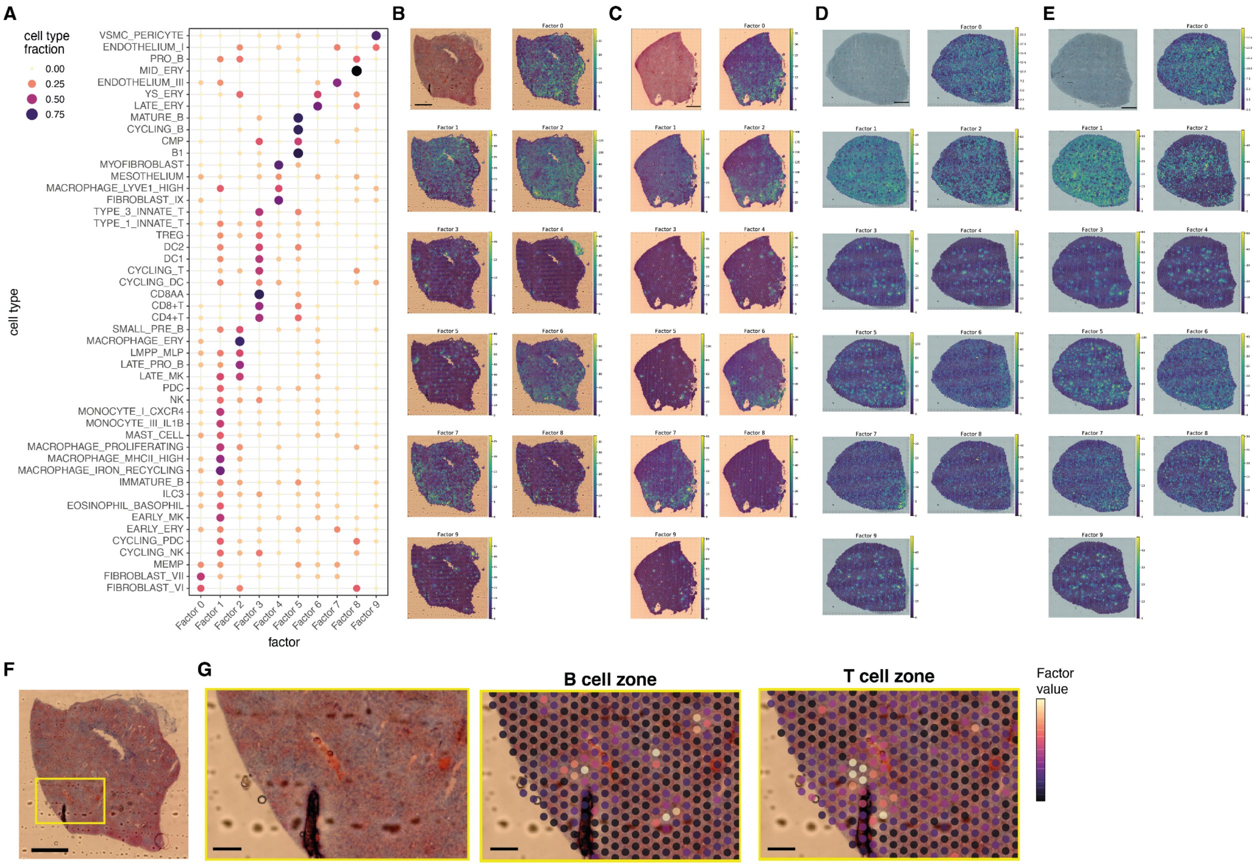

fig. S10: Cell type spatial microenvironments in the fetal spleen detected by non-negative matrix factorization on spatial abundances. (**A**) Dot plot of cell type contributions to latent factors (microenvironments) identified with non-negative matrix factorization of spatial cell type abundances estimated with cell2location. The color and the size of the dots represent the relative fraction of the cell population assigned to the factor. We exclude cell types where the value for the 99% quantile of cell abundance in all the slides from the same organ is always below the detection threshold of 0.15. (**B** to **E**) Spatial locations of microenvironments on spleen slides, with the color representing the weighted contribution of each microenvironment to each spot. H&E staining images for each slide are shown for reference (scale bar: 1 mm). (**F**) Illustration of inset displayed in G of fetal spleen tissue slide shown in B (scale bar: 1 mm). (**G**) Higher magnification view of slide in F showing weighted microenvironment contribution (factor values) of lymphoid aggregates B cell zone microenvironment (Factor 8) and T cell zone microenvironment (Factor 9) (scale bar: 200 μm). These exemplify how the B and T cell zones were proximal to each other but did not completely overlap.

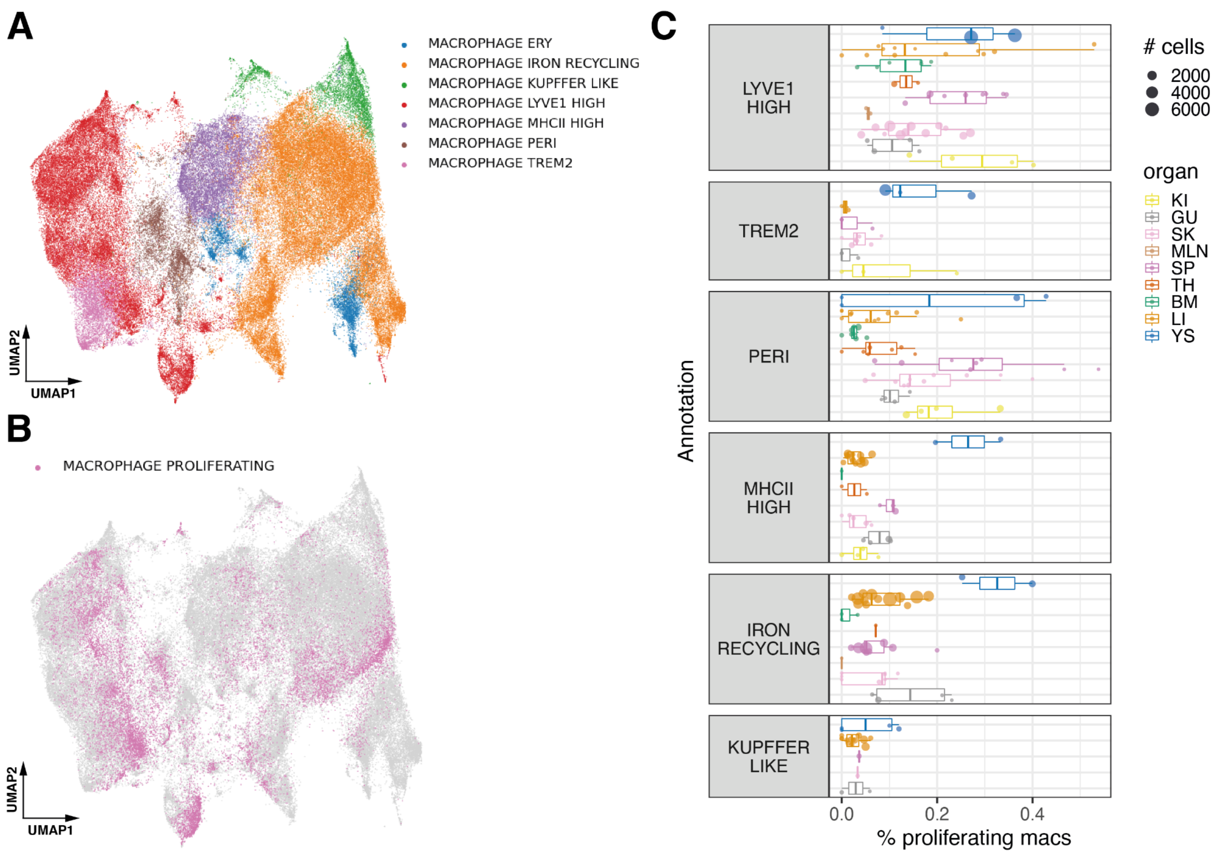

fig. S11: Distribution of proliferating macrophages. (**A**) UMAP embedding of macrophage cells colored by annotated subpopulations. Dimensionality reduction with scVI, KNN graph construction and UMAP embedding were performed on macrophages as described for other subsets. We repeated Leiden clustering on this embedding and propagated labels from the annotations obtained on the myeloid embedding. (**B**) Distribution and mixing of proliferating ($MKI67^{+}TOP2A^{+}$) macrophages identified in myeloid clustering (fig. S4G). (**C**) Fraction of proliferating macrophages within each macrophage subpopulation defined as in (A). Each point represents one embryo/fetus, color-coded by organ. The size of the point represents the cell count (YS: yolk sac; LI: liver; BM: bone marrow; TH: thymus; SP: spleen; MLN: mesenteric lymph node; SK: skin; GU: gut; KI: kidney).

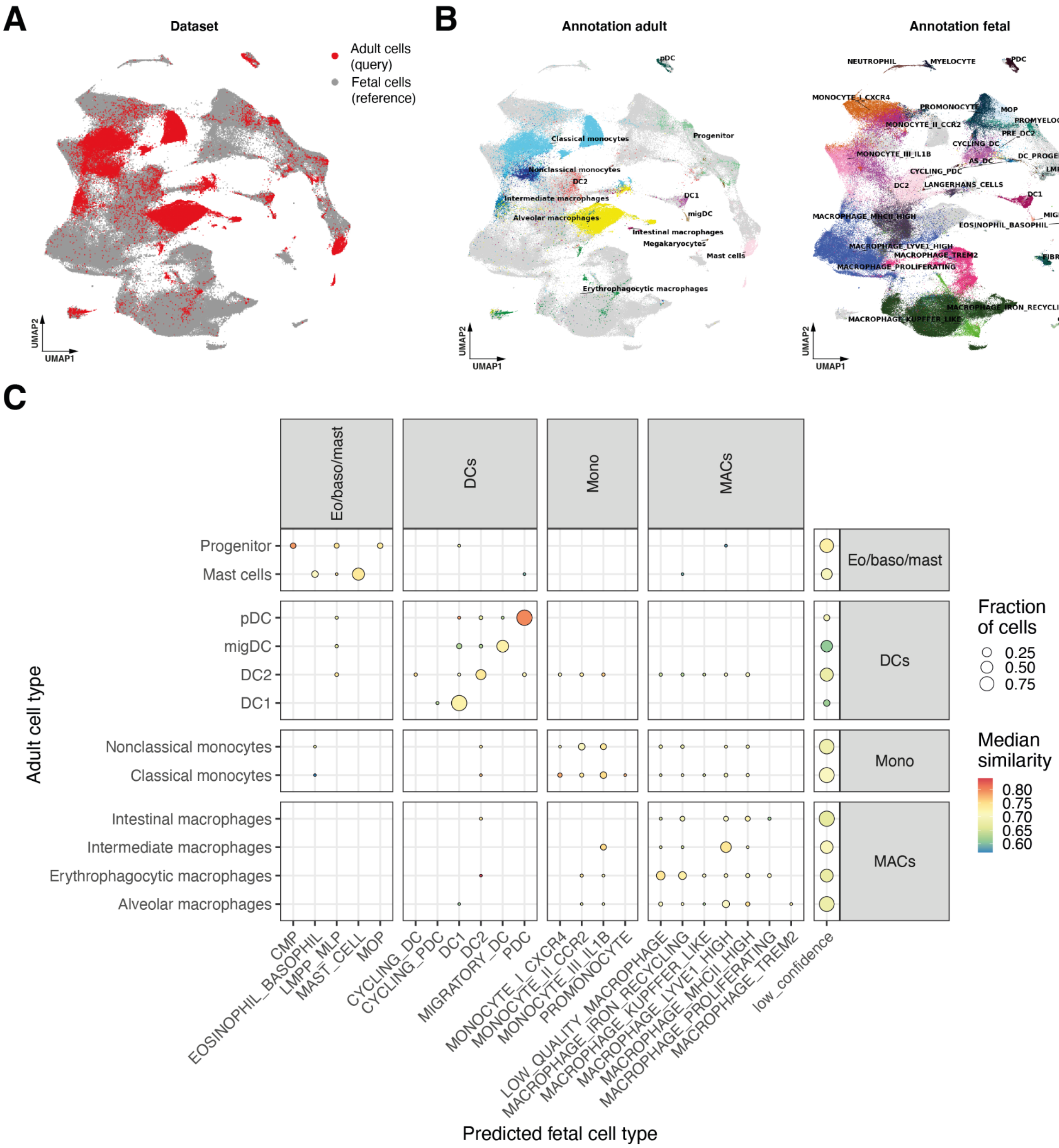

fig. S12: Mapping of adult myeloid cells to prenatal reference with transfer learning. (**A**) UMAP embeddings of mapping of adult myeloid cells (54,047 cells) to developmental myeloid reference (218,758 cells) using scArches on scVI model. Points are colored by the dataset of origin. (**B**) UMAP embedding as in A, points are colored by cell population annotation label, for adult cells (left) and developmental cells (right). (**C**) Correspondence between developmental and adult myeloid transcriptional phenotypes estimated by label transfer after mapping with scArches. The dot size is proportional to the fraction of cells in the adult population (*y*-axis) with a given predicted prenatal cell population label (*x*-axis). The dot color denotes the median similarity of the adult cells to prenatal cells in the common embedding. Adult cells where less than 80% of prenatal neighbors have a uniform annotation are labeled as “low confidence”.

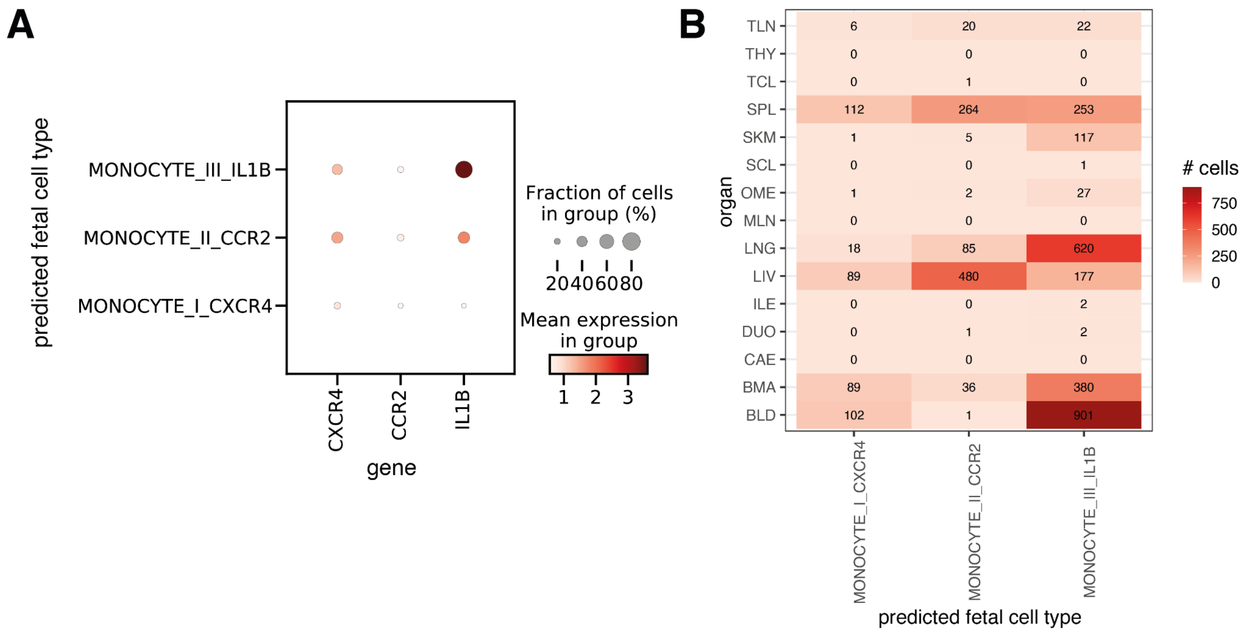

organ	MONOCYTE_I_CXCR4	MONOCYTE_II_CCR2	MONOCYTE_III_IL1B
TLN	6	20	22
THY	0	0	0
TCL	0	1	0
SPL	112	264	253
SKM	1	5	117
SCL	0	0	1
OME	1	2	27
MLN	0	0	0
LNG	18	85	620
LIV	89	480	177
ILE	0	0	2
DUO	0	1	2
CAE	0	0	0
BMA	89	36	380
BLD	102	1	901

fig. S13: Prenatal-adult comparison in monocytes. (**A**) Dot plot of expression of monocyte subtype markers in adult cells aligned to prenatal monocyte subtypes. (**B**) Heat map of distribution across organs of adult cells aligned to prenatal monocyte subtypes (TLN: thoracic lymph nodes, THY: thymus, TCL: transverse colon, SPL: spleen, SKM: skeletal muscle, SCL: sigmoid colon, OME: omentum, MLN: mesenteric lymph node, LNG: lung, LIV: liver, ILE: ileum, DUO: duodenum, CAE: cecum, BMA: bone marrow, BLD: blood).

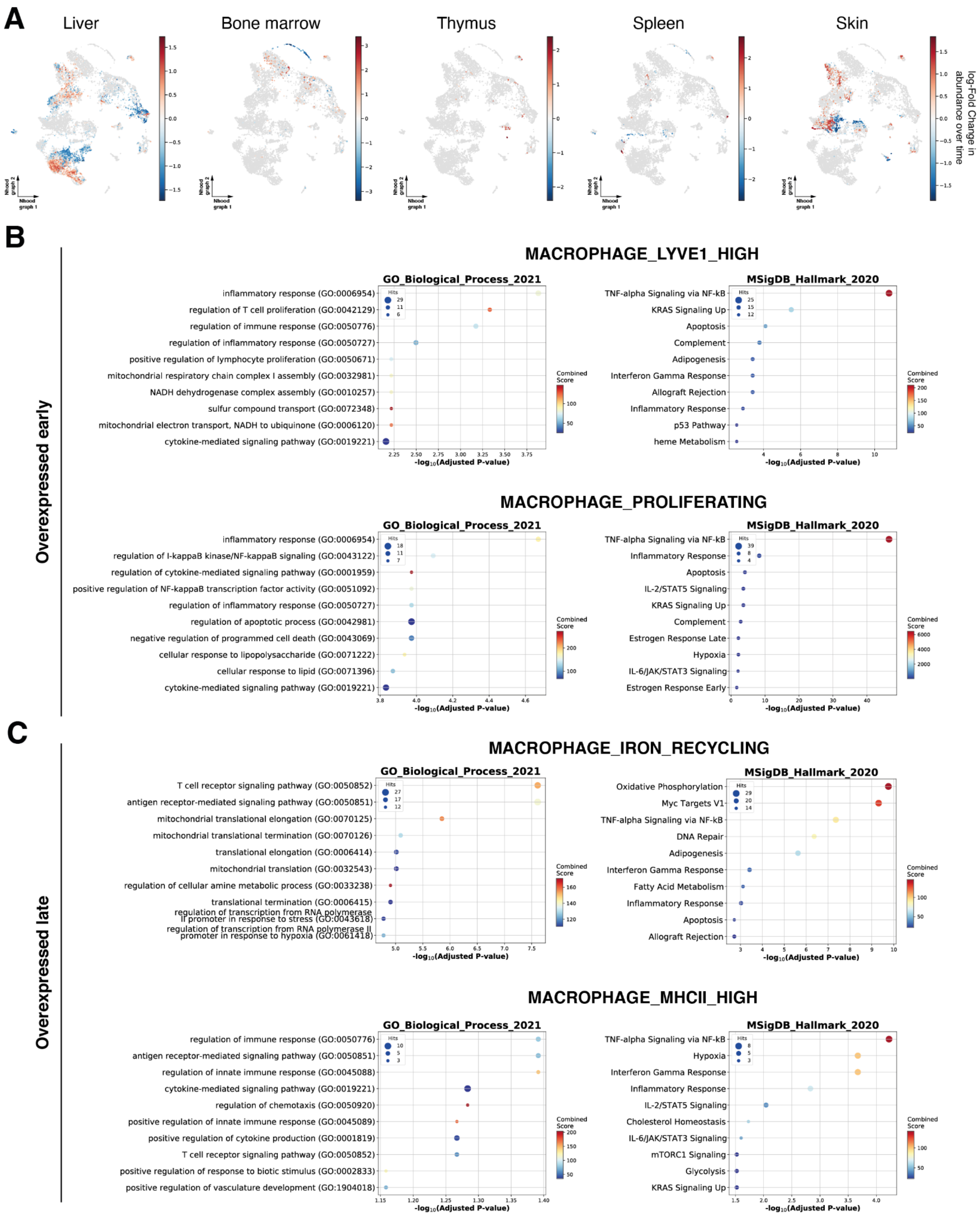

A
Liver
Bone marrow
Thymus
Spleen
Skin
log-Fold Change in abundance over time
Nhood graph 1
Nhood graph 2
B
Overexpressed early
MACROPHAGE_LYVE1_HIGH
GO_Biological_Process_2021
inflammatory response (GO:0006954)
regulation of T cell proliferation (GO:0042129)
regulation of immune response (GO:0050776)
regulation of inflammatory response (GO:0050727)
positive regulation of lymphocyte proliferation (GO:0050671)
mitochondrial respiratory chain complex I assembly (GO:0032981)
NADH dehydrogenase complex assembly (GO:0010257)
sulfur compound transport (GO:0072348)
mitochondrial electron transport, NADH to ubiquinone (GO:0006120)
cytokine-mediated signaling pathway (GO:0019221)
MSigDB_Hallmark_2020
TNF-alpha Signaling via NF-kB
KRAS Signaling Up
Apoptosis
Complement
Adipogenesis
Interferon Gamma Response
Allograft Rejection
Inflammatory Response
p53 Pathway
heme Metabolism
MACROPHAGE_PROLIFERATING
GO_Biological_Process_2021
inflammatory response (GO:0006954)
regulation of I-kappaB kinase/NF-kappaB signaling (GO:0043122)
regulation of cytokine-mediated signaling pathway (GO:0001959)
positive regulation of NF-kappaB transcription factor activity (GO:0051092)
regulation of inflammatory response (GO:0050727)
regulation of apoptotic process (GO:0042981)
negative regulation of programmed cell death (GO:0043069)
cellular response to lipopolysaccharide (GO:0071222)
cellular response to lipid (GO:0071396)
cytokine-mediated signaling pathway (GO:0019221)
MSigDB_Hallmark_2020
TNF-alpha Signaling via NF-kB
Inflammatory Response
Apoptosis
IL-2/STAT5 Signaling
KRAS Signaling Up
Complement
Estrogen Response Late
Hypoxia
IL-6/JAK/STAT3 Signaling
Estrogen Response Early
C
Overexpressed late
MACROPHAGE_IRON_RECYCLING
GO_Biological_Process_2021
T cell receptor signaling pathway (GO:0050852)
antigen receptor-mediated signaling pathway (GO:0050851)
mitochondrial translational elongation (GO:0070125)
mitochondrial translational termination (GO:0070126)
translational elongation (GO:0006414)
mitochondrial translation (GO:0032543)
regulation of cellular amine metabolic process (GO:0033238)
translational termination (GO:0006415)
regulation of transcription from RNA polymerase II promoter in response to stress (GO:0043618)
regulation of transcription from RNA polymerase II promoter in response to hypoxia (GO:0061418)
MSigDB_Hallmark_2020
Oxidative Phosphorylation
Myc Targets V1
TNF-alpha Signaling via NF-kB
DNA Repair
Adipogenesis
Interferon Gamma Response
Fatty Acid Metabolism
Inflammatory Response
Apoptosis
Allograft Rejection
MACROPHAGE_MHCII_HIGH
GO_Biological_Process_2021
regulation of immune response (GO:0050776)
antigen receptor-mediated signaling pathway (GO:0050851)
regulation of innate immune response (GO:0045088)
cytokine-mediated signaling pathway (GO:0019221)
regulation of chemotaxis (GO:0050920)
positive regulation of innate immune response (GO:0045089)
positive regulation of cytokine production (GO:0001819)
T cell receptor signaling pathway (GO:0050852)
positive regulation of response to biotic stimulus (GO:0002833)
positive regulation of vasculature development (GO:1904018)
MSigDB_Hallmark_2020
TNF-alpha Signaling via NF-kB
Hypoxia
Interferon Gamma Response
Inflammatory Response
IL-2/STAT5 Signaling
Cholesterol Homeostasis
IL-6/JAK/STAT3 Signaling
mTORC1 Signaling
Glycolysis
KRAS Signaling Up
Hits
Combined Score
-log10(Adjusted P-value)

fig. S14: Differential abundance across gestation in myeloid cell populations. (**A**) Milo neighborhood embedding of myeloid cells showing differential abundance across gestation. Each point represents a neighborhood, the layout of points is determined by the position of the neighborhood index cell in the UMAP in fig. S4G, the size of points is proportional to the number of cells in the neighborhood. Neighborhoods are colored by their log-fold change (logFC) in abundance over time, where logFC>0 indicates significant enrichment in early cells and logFC > 0 indicates significant enrichment in late cells. Only neighborhoods showing significant differential abundance (SpatialFDR<10%) are colored. (**B** and **C**) Gene set enrichment analysis results for differentially expressed genes in gestation stage-specific neighborhoods of macrophages. Each plot shows the top 10 significant hits for the gene list. The *x*-axis shows the negative $\log_{10}$ of the *P*-value adjusted for multiple testing (Benjamini–Hochberg correction). The size of the dots is proportional to the number of genes associated with the gene set. The color represents the combined enrichr score calculated with gseapy. Results using the Gene Ontology Biological Process and the MSigDB Hallmark 2020 databases are shown. (B) Gene set enrichment analysis for genes overexpressed in early-specific neighborhoods of LYVE1^{hi} macrophages and proliferating macrophages. (C) Gene set enrichment analysis for genes overexpressed in late-specific neighborhoods of iron-recycling macrophages and MHCII^{hi} macrophages.

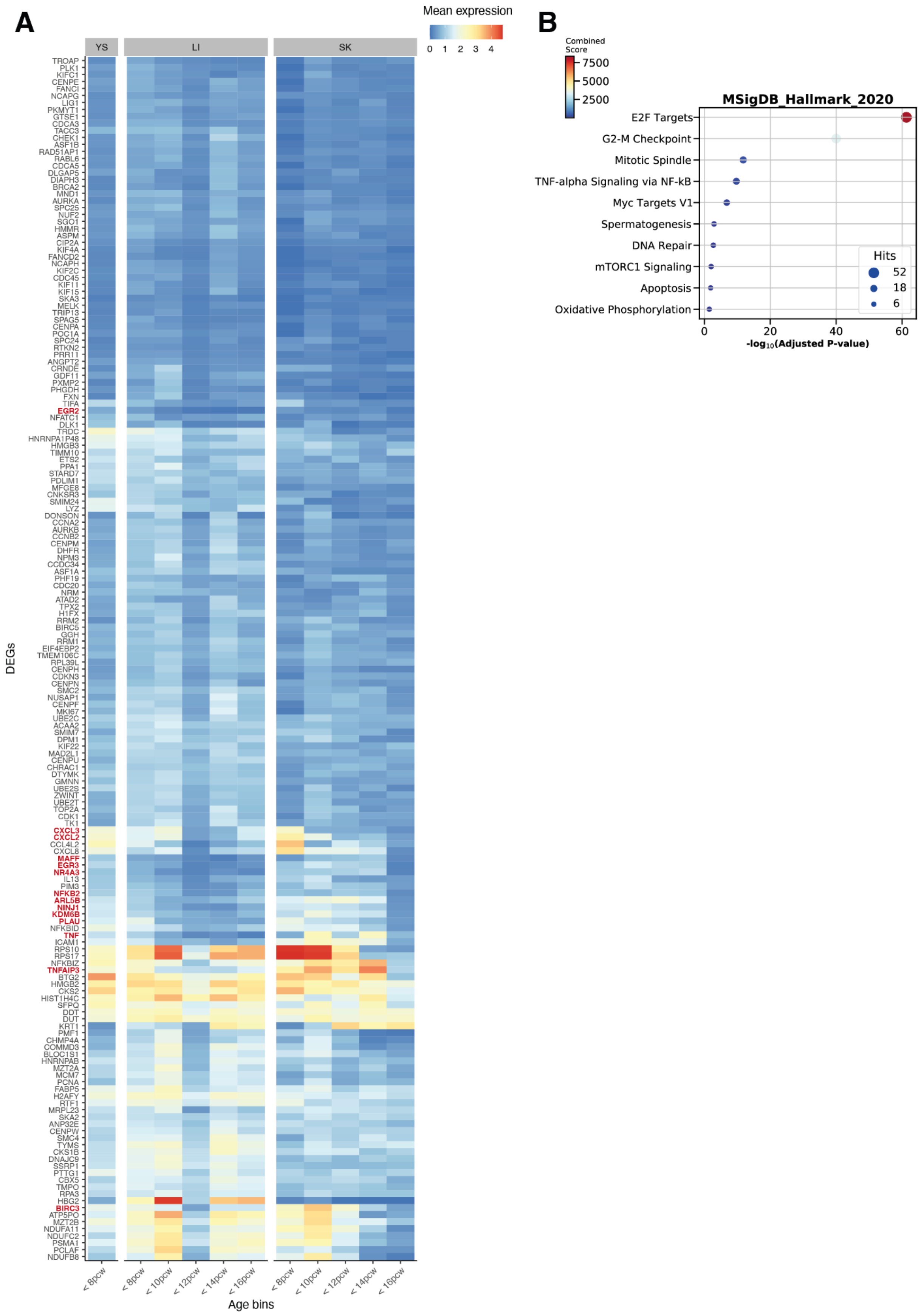
A
Mean expression
0 1 2 3 4
YS
LI
SK
DEGs
TROAP
PLK1
KIFC1
CENPE
FANCI
NCAPG
LIG1
PKMYT1
GTSE1
CDCA3
TACC3
CHEK1
ASF1B
RAD51AP1
RABL6
CDCA5
DLGAP5
DIAPH3
BRCA2
MND1
AURKA
SPC25
NUF2
SGO1
HMMR
ASPM
CIP2A
KIF4A
FANCD2
NCAPH
KIF2C
CDC45
KIF11
KIF15
SKA3
MELK
TRIP13
SPAG5
CENPA
POC1A
SPC24
RTKN2
PRR11
ANGPT2
CRNDE
GDF11
PXMP2
PHGDH
FXN
TIFA
EGR2
NFATC1
DLK1
TRDC
HNRNPA1P48
HMGB3
TIMM10
ETS2
PPA1
STARD7
PDLIM1
MFGE8
CNKSR3
SMIM24
LYZ
DONSON
CCNA2
AURKB
CCNB2
CENPM
DHFR
NPM3
CCDC34
ASF1A
PHF19
CDC20
NRM
ATAD2
TPX2
H1FX
RRM2
BIRC5
GGH
RRM1
EIF4EBP2
TMEM106C
RPL39L
CENPH
CDKN3
CENPN
SMC2
NUSAP1
CENPF
MKI67
UBE2C
ACAA2
SMIM7
DPM1
KIF22
MAD2L1
CENPU
CHRAC1
DTYMK
GMNN
UBE2S
ZWINT
UBE2T
TOP2A
CDK1
TK1
CXCL3
CXCL2
CCL4L2
CXCL8
MAFF
EGR3
NR4A3
IL13
PIM3
NFKB2
ARL5B
NINJ1
KDM6B
PLAU
NFKBID
TNF
ICAM1
RPS10
RPS17
NFKBIZ
TNFAIP3
BTG2
HMGB2
CKS2
HIST1H4C
SFPQ
DDT
DUT
KRT1
PMF1
CHMP4A
COMMD3
BLOC1S1
HNRNPAB
MZT2A
MCM7
PCNA
FABP5
H2AFY
RTF1
MRPL23
SKA2
ANP32E
CENPW
SMC4
TYMS
CKS1B
DNAJC9
SSRP1
PTTG1
CBX5
TMPO
RPA3
HBG2
BIRC3
ATP5PO
MZT2B
NDUFA11
NDUFC2
PSMA1
PCLAF
NDUFB8
< 8pcw
< 8pcw
< 10pcw
< 12pcw
< 14pcw
< 16pcw
< 8pcw
< 10pcw
< 12pcw
< 14pcw
< 16pcw
Age bins
B
Combined Score
7500
5000
2500
MSigDB_Hallmark_2020
E2F Targets
G2-M Checkpoint
Mitotic Spindle
TNF-alpha Signaling via NF-kB
Myc Targets V1
Spermatogenesis
DNA Repair
mTORC1 Signaling
Apoptosis
Oxidative Phosphorylation
Hits
52
18
6
0
20
40
60
-log10(Adjusted P-value)

fig. S15: Differential expression analysis on early-specific neighborhoods of mast cells. (**A**) Average expression by time point of 185 genes overexpressed in early-specific neighborhoods of mast cells. Genes associated with TNF signaling via NF-κB are highlighted in red. (**B**) Gene set enrichment analysis results using the MSigDB Hallmark 2020 database. The *x*-axis shows the negative $\log_{10}$ of the *P*-value adjusted for multiple testing (Benjamini–Hochberg correction). The size of the dots is proportional to the number of genes associated with the gene set. The color represents the combined enrichr score calculated with gseapy.

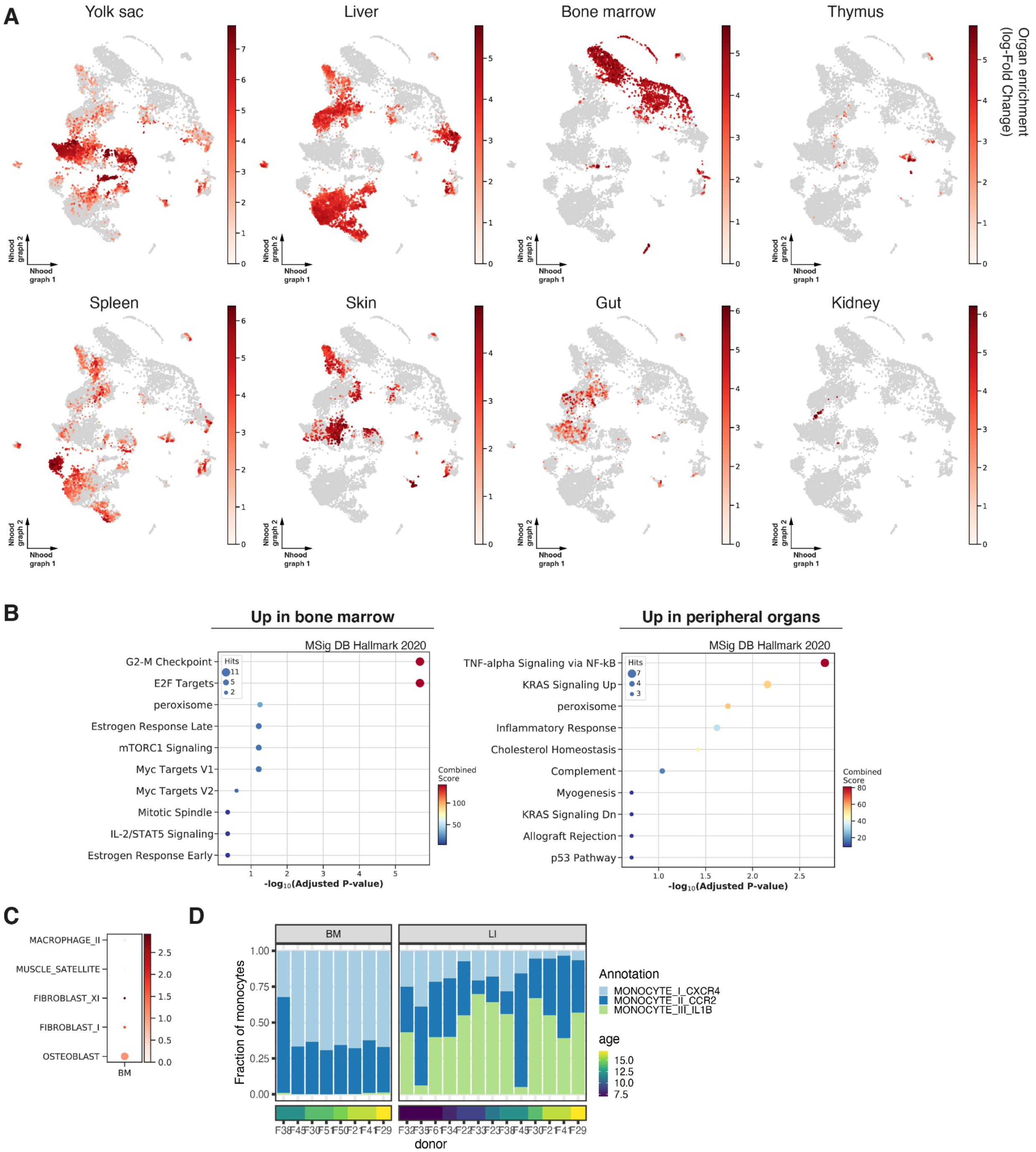

fig. S16: Differential abundance across organs in myeloid cell populations. (**A**) Milo neighborhood embedding of myeloid cells. Each point represents a neighborhood, the layout of points is determined by the position of the neighborhood index cell in the UMAP in fig. S4G, the size of points is proportional to the number of cells in the neighborhood. Neighborhoods are colored by their log-fold change in abundance between the specified organ and all other organs. Only neighborhoods showing significant enrichment (SpatialFDR<10% and logFC≥2) are colored. (**B**) Dot plot of enrichment analysis results on genes upregulated (left) and downregulated (right) in bone marrow CCR2^{hi} monocytes compared to other organs. The *x*-axis represents the negative $\log_{10}$ of *P*-values adjusted for multiple testing (Benjamini–Hochberg correction) The *y*-axis shows the top 10 enriched gene sets (using the MSigDB Hallmark 2020 database). The size of the dots is proportional to the number of genes associated with the

gene set. The color represents the combined enrichr score calculated with gseapy. (**C**) Dot plot of *CXCL12* expression in cell populations in the bone marrow. The color represents the average expression level (normalized and log-transformed counts) and the size represents the cell count of each cell type within bone marrow. Only cell populations with average expression > 1, and cell count > 10 are shown. (**D**) Fraction of abundance of monocyte subsets for each donor in liver (LI) and bone marrow (BM). Donors are ordered by gestational age.

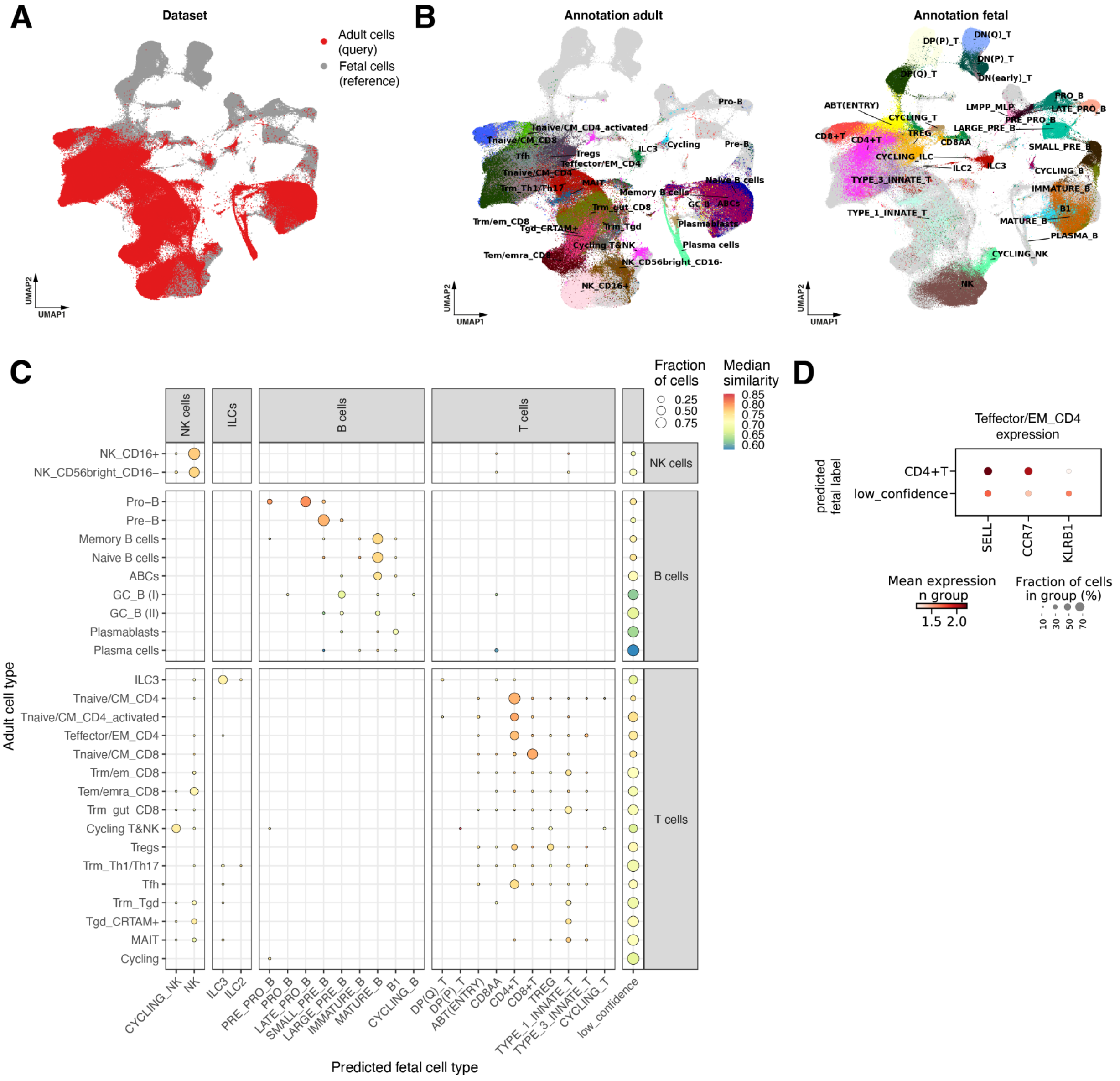

fig. S17: Mapping of adult lymphoid cells to prenatal reference with transfer learning. (**A**) UMAP embeddings of mapping of adult lymphoid cells (264,929) to prenatal lymphoid reference (218,758 cells) using scArches on scVI model. Points are colored by the dataset of origin. (**B**) UMAP embedding as in A, points are colored by cell population annotation label, for adult cells (left) and prenatal cells (right). (**C**) Correspondence between prenatal and adult myeloid transcriptional phenotypes estimated by label transfer after mapping with scArches. The dot size is proportional to the fraction of cells in the adult population (*y*-axis) with a given predicted prenatal cell population label (*x*-axis). The dot color denotes the median similarity of the adult cells to prenatal cells in the common embedding. Adult cells where less than 80% of prenatal neighbors have a uniform annotation are labeled as “low confidence”. (**D**) The fraction of adult CD4^{+} effector T cells that were matched to fetal CD4^{+} T cells show increased expression of naive markers (SELL, CCR7) and CD4^{+}T effector markers (KLRB1) compared to effector cells not having a developmental equivalent (low confidence). This indicates that the matching might be driven by additional heterogeneity in this adult cell compartment, instead of the true correspondent of adult memory T cells within fetal cells.

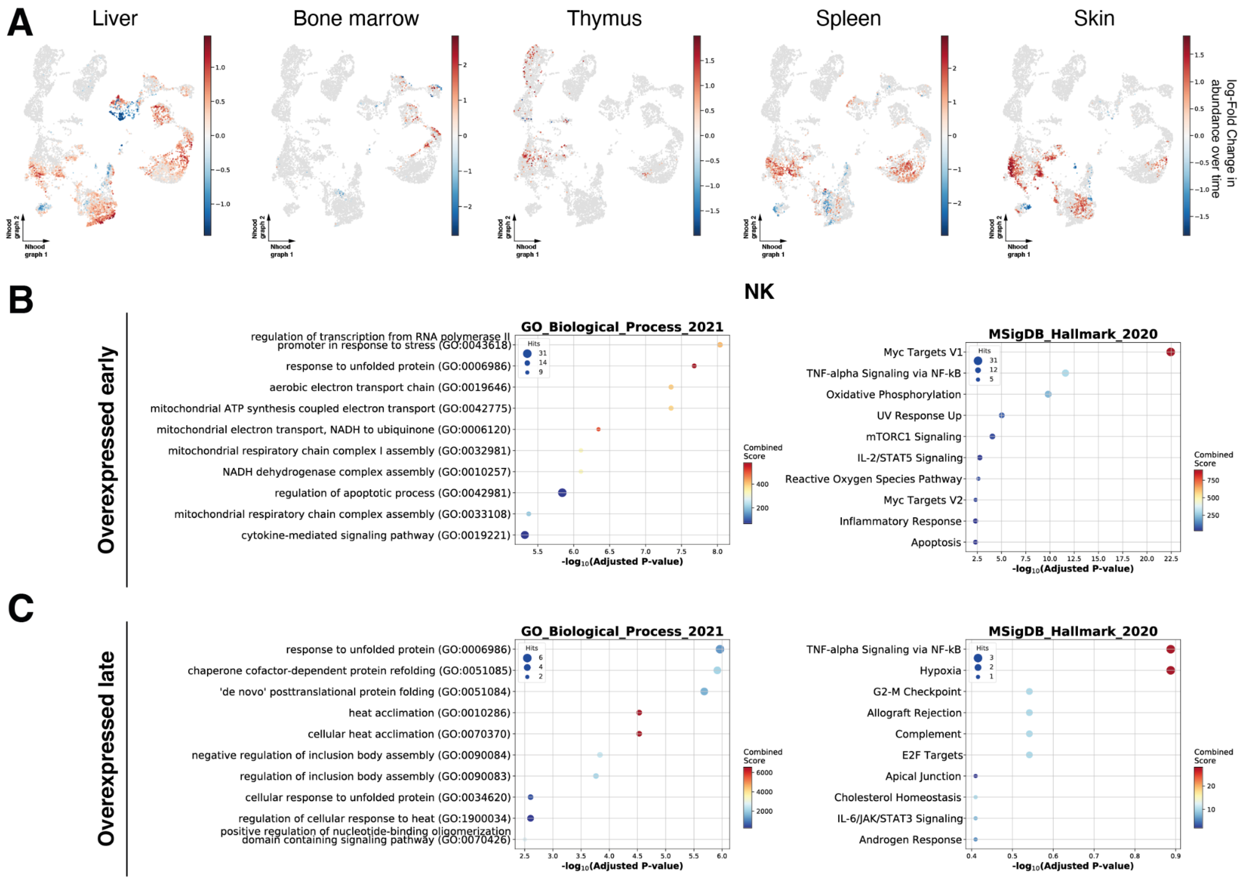

fig. S18: Differential abundance across gestation in lymphoid cell populations. (**A**) Milo neighborhood embedding of lymphoid cells showing differential abundance across gestation. Each point represents a neighborhood, the layout of points is determined by the position of the neighborhood index cell in the UMAP in fig. S4I, the size of points is proportional to the number of cells in the neighborhood. Neighborhoods are colored by their log-fold change (logFC) in abundance over time, where logFC > 0 indicates significant enrichment in early cells and logFC > 0 indicates significant enrichment in late cells. Only neighborhoods showing significant differential abundance (SpatialFDR < 10%) are colored. (**B-C**) Gene set enrichment analysis results for differentially expressed genes in early-specific neighborhoods (B) and late specific neighborhoods (C) of NK cells. Each plot shows the top 10 significant hits for the gene set. The *x*-axis shows the negative $\log_{10}$ of the *P*-value adjusted for multiple testing (Benjamini–Hochberg correction). The size of the dots is proportional to the number of genes associated with the gene set. The color represents the combined enrichr score calculated with gseapy. Results using the Gene Ontology Biological Process and the MSigDB Hallmark 2020 databases are shown.

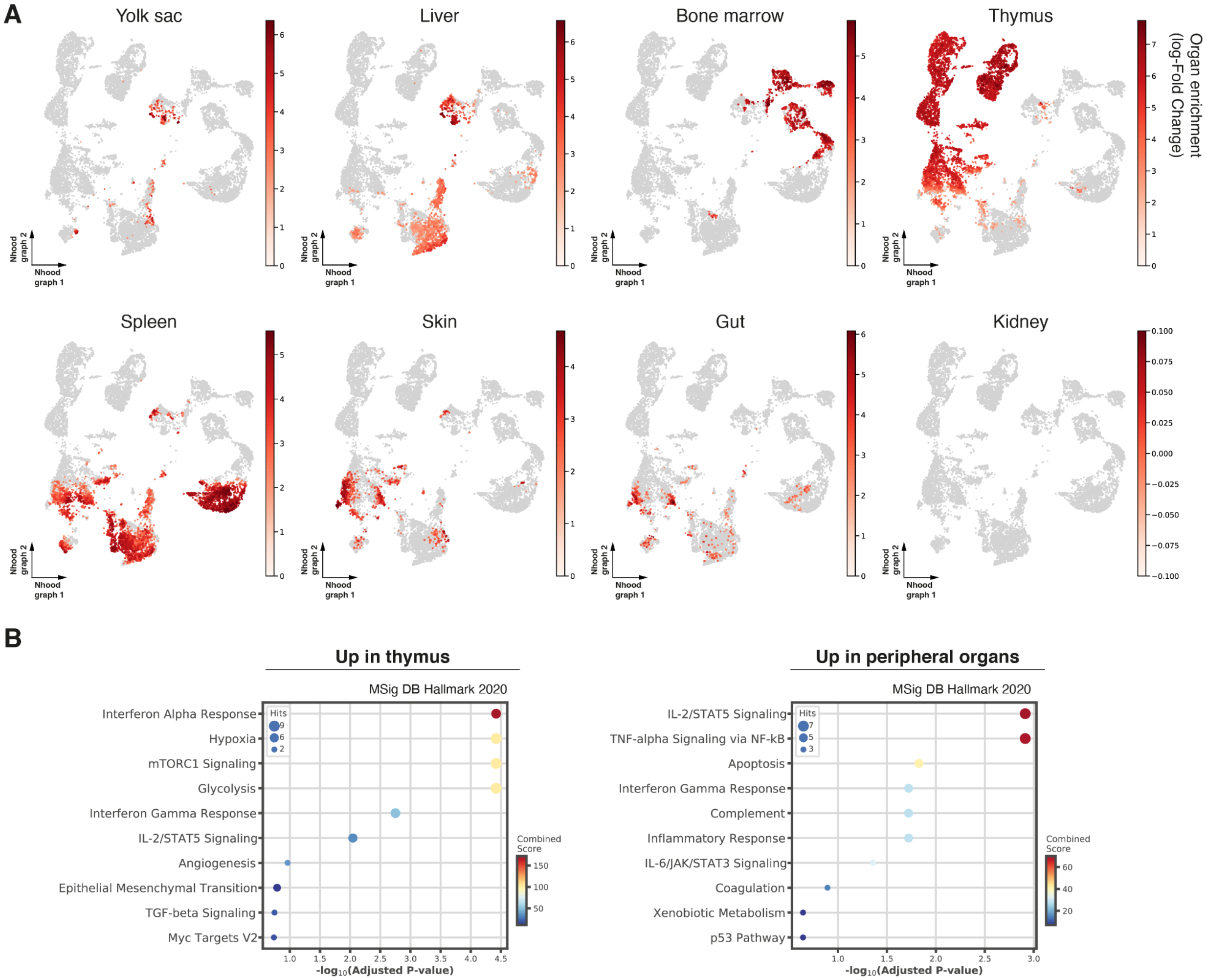

fig. S19: Differential abundance across organs in lymphoid compartment. (**A**) Milo neighborhood embedding of lymphoid cells showing differential abundance between organs. Each point represents a neighborhood, the layout of points is determined by the position of the neighborhood index cell in the UMAP in fig. S4I, the size of points is proportional to the number of cells in the neighborhood. Neighborhoods are colored by their log-fold change in abundance between the specified organ and all other organs. Only neighborhoods showing significant enrichment (SpatialFDR < 10% and log-fold change > 2) are colored. (**B**) Dot plot of enrichment analysis results on genes upregulated (left) and downregulated (right) in thymic mature T cells compared to other organs. The *x*-axis represents the negative $\log_{10}$ of *P*-values adjusted for multiple testing (Benjamini–Hochberg correction) The *y*-axis shows the top 10 enriched gene sets (using the MSigDB Hallmark 2020 database). The size of the dots is proportional to the number of genes associated with the gene set. The color represents the combined enrichr score calculated with gseapy.

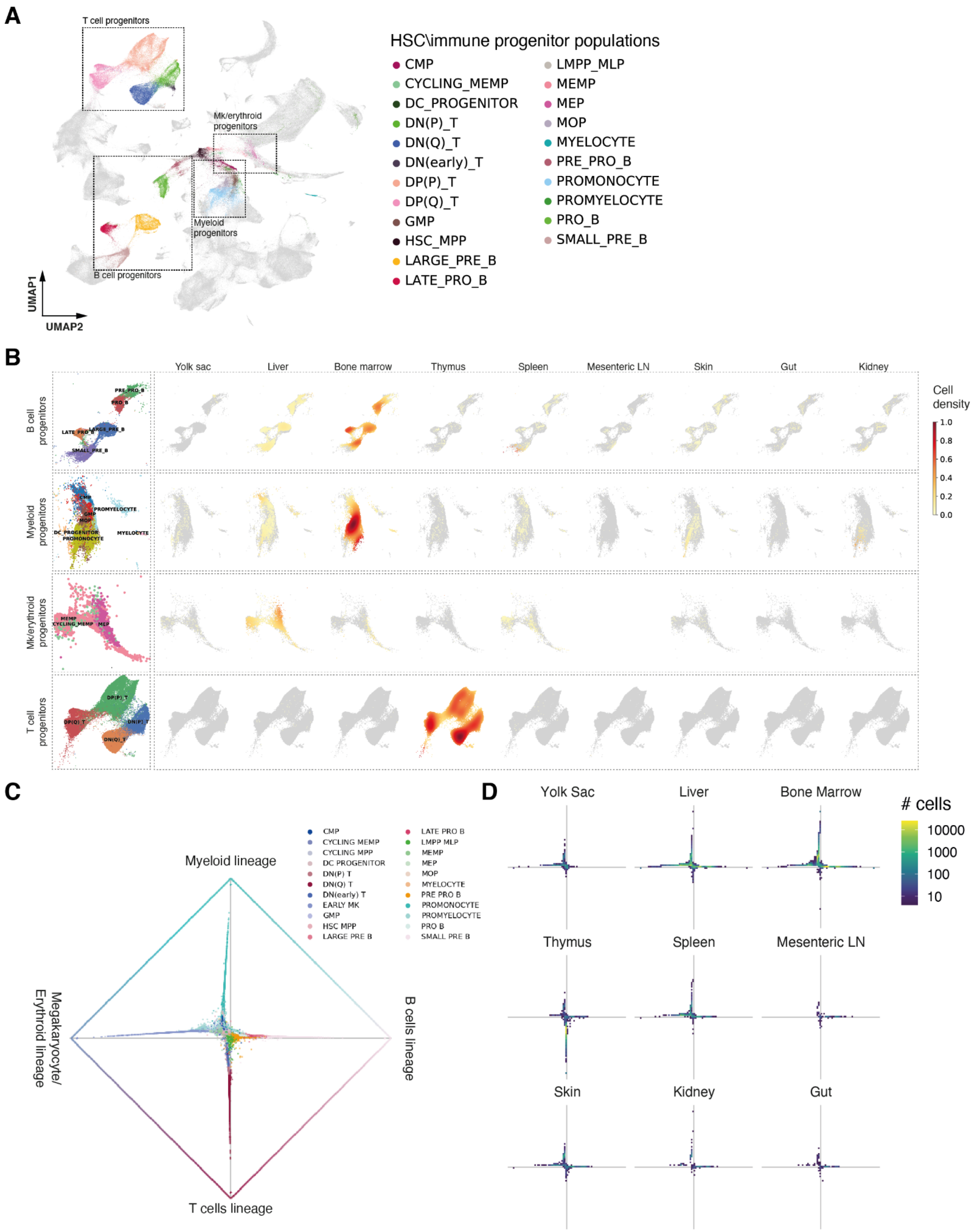
A
T cell progenitors
Mk/erythroid progenitors
Myeloid progenitors
B cell progenitors
UMAP1
UMAP2
HSC\immune progenitor populations
CMP
CYCLING_MEMP
DC_PROGENITOR
DN(P)_T
DN(Q)_T
DN(early)_T
DP(P)_T
DP(Q)_T
GMP
HSC_MPP
LARGE_PRE_B
LATE_PRO_B
LMPP_MLP
MEMP
MEP
MOP
MYELOCYTE
PRE_PRO_B
PROMONOCYTE
PROMYELOCYTE
PRO_B
SMALL_PRE_B
B
Yolk sac
Liver
Bone marrow
Thymus
Spleen
Mesenteric LN
Skin
Gut
Kidney
B cell progenitors
Myeloid progenitors
Mk/erythroid progenitors
T cell progenitors
Cell density
C
Myeloid lineage
B cells lineage
T cells lineage
Megakaryocyte/ Erythroid lineage
D
Yolk Sac
Liver
Bone Marrow
Thymus
Spleen
Mesenteric LN
Skin
Kidney
Gut
cells
10000
1000
100
10

fig. S20. Full spectrum of hematopoietic progenitors in peripheral organs. (**A**) UMAP embedding of all immune and blood cells, highlighting the progenitor cell populations. Dashed boxes highlight lineage populations shown in B. (**B**) Density plot of cells from each organ on a subset of UMAP embedding for different hematopoietic lineages. Density is calculated over all immune cells within each organ. (**C**) Simplex projection of cells in progenitor populations according to fate probabilities for each immune and blood cell lineage. Each cell is placed inside the simplex according to its probability of reaching any of the terminal states. Cells in the center have higher multilineage potential, whereas cells closer to one of the corners are more committed. (**D**) Binned density of cells for each organ over the fate simplex shown in C, where the color of the 2D bin represents the number of cells from the organ in that position in the simplex.

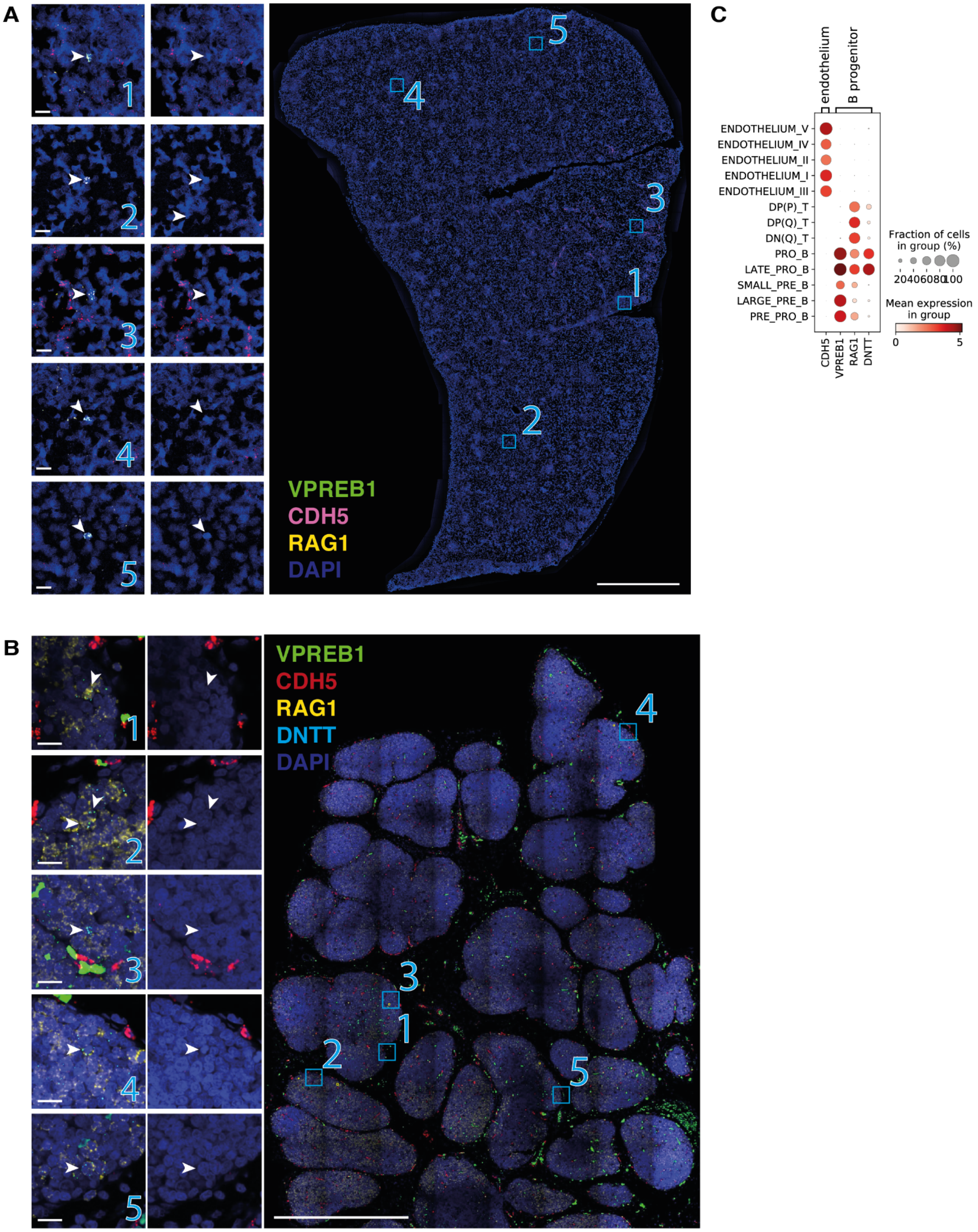

fig. S21. Multiplex smFISH validation of B cell progenitors in peripheral tissues. Multiplex smFISH staining of DAPI, *CDH5* for endothelial cells, and *VPREB1*, *RAG1* with/without *DNTT* for B cell progenitors in the human (**A**) prenatal spleen at 14 pcw and (**B**) prenatal thymus at 16 pcw. Left: cells highlighted from corresponding regions in the right panel overview matched by numbers (scale bar: 20 µm). Middle: *CDH5* channel alone depicting endothelial cells. Right: full section view with the areas of interest boxed (scale bar: 1 mm). White arrows point to B cell progenitors identified. (**C**) Dot plot showing log normalized expressions of *CDH5*, *VPREB1*, *RAG1*, and *DNTT* in the corresponding cell populations. Only cell types with log normalized expression at least 2 in at least one of the four genes are shown here.

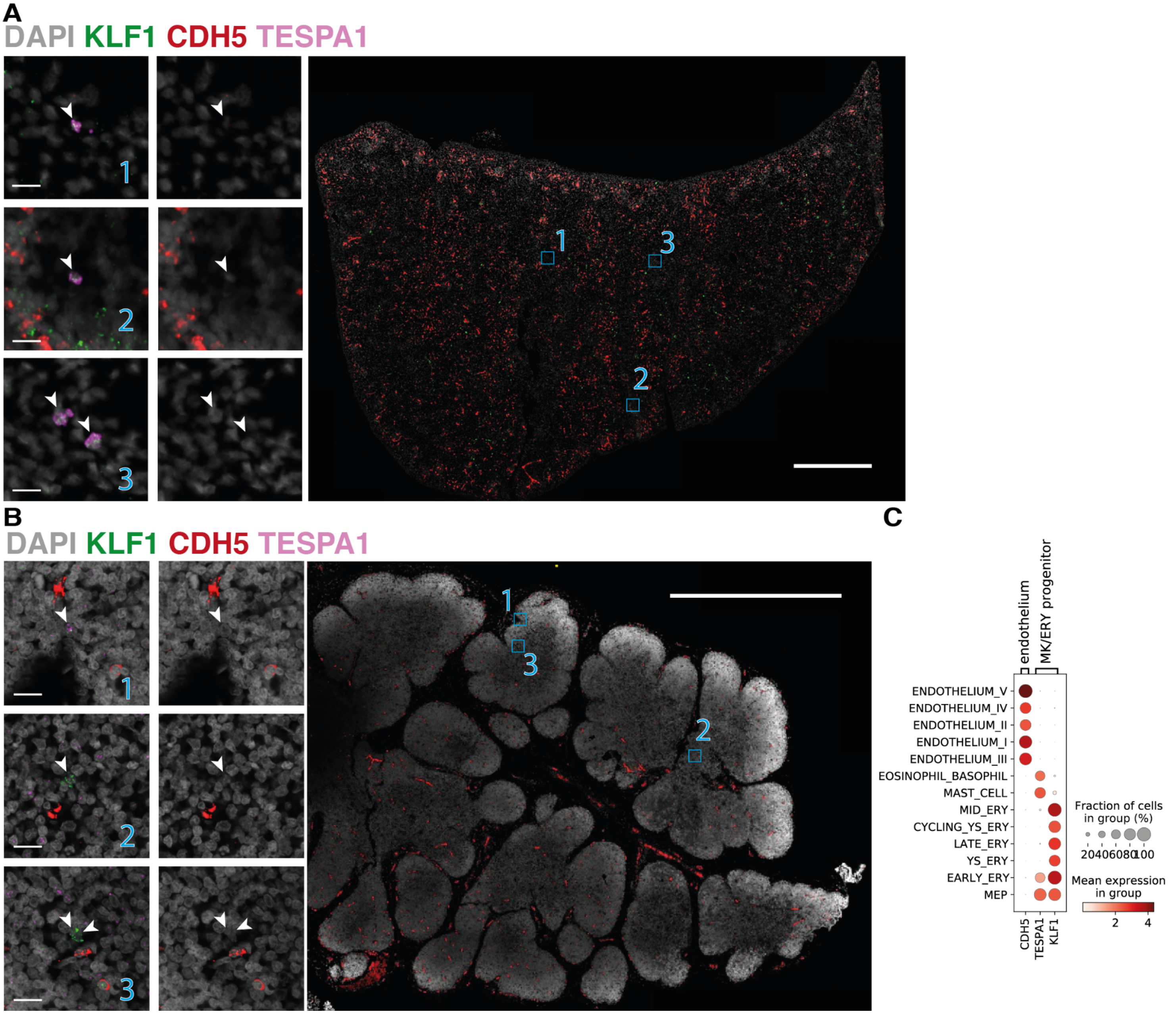

fig. S22. Multiplex smFISH validation of megakaryocyte/erythroid progenitors in peripheral tissues. Multiplex smFISH staining of DAPI, *CDH5* for endothelial cells, and *KLF1*, *TESPA1* for megakaryocyte/erythroid progenitors in the human (**A**) prenatal spleen at 14 pcw and (**B**) prenatal thymus at 16 pcw. Left: cells highlighted from corresponding regions in the right panel overview matched by numbers (scale bar: 20 µm). Middle: *CDH5* channel alone depicting endothelial cells. Right: full section view with the areas of interest boxed (scale bar: 1 mm). White arrows point to megakaryocyte/erythroid progenitors identified. (**C**) Dot plot showing log normalized expressions of *CDH5*, *KLF1*, and *TESPA1* in the corresponding cell populations. Only cell types with log normalized expression at least 2 in at least one of the three genes are shown here.

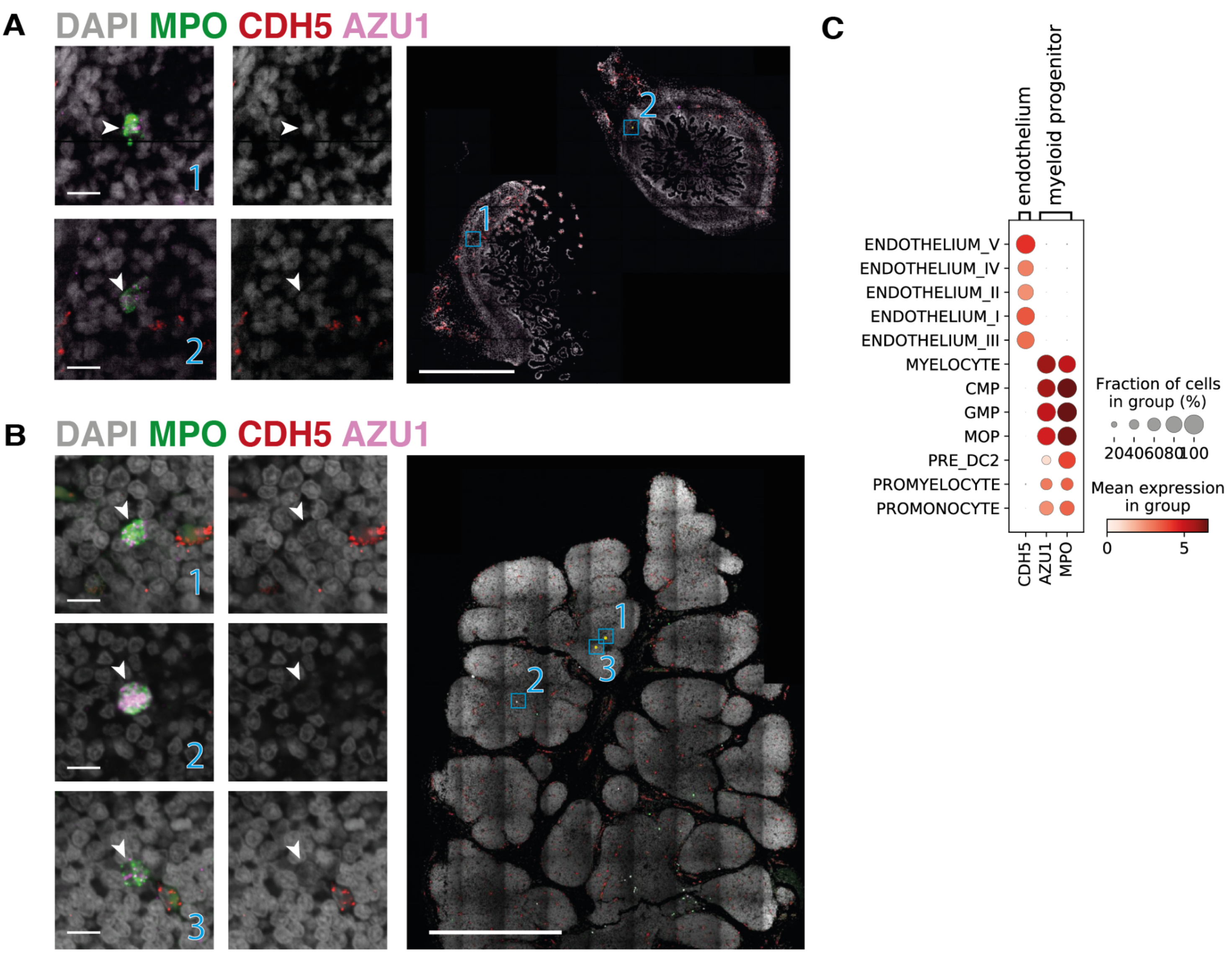

fig. S23. Multiplex smFISH validation of myeloid progenitors in peripheral tissues. Multiplex smFISH staining of DAPI, *CDH5* for endothelial cells, and *MPO* and *AZU1* for myeloid progenitors in the human (**A**) prenatal intestine at 15 pcw and (**B**) prenatal thymus at 16 pcw. Left: cells highlighted from corresponding regions in the right panel overview matched by numbers (scale bar: 20 μm in (**A**) and 10 μm in (**B**)). Middle: *CDH5* channel alone depicting endothelial cells. Right: full section view with the areas of interest boxed (scale bar: 1 mm). White arrows point to myeloid progenitors identified. (**C**) Dot plot showing log normalized expressions of *CDH5*, *MPO*, and *AZU1* in the corresponding cell populations. Only cell types with log normalized expression at least 2 in at least one of the three genes are shown here.

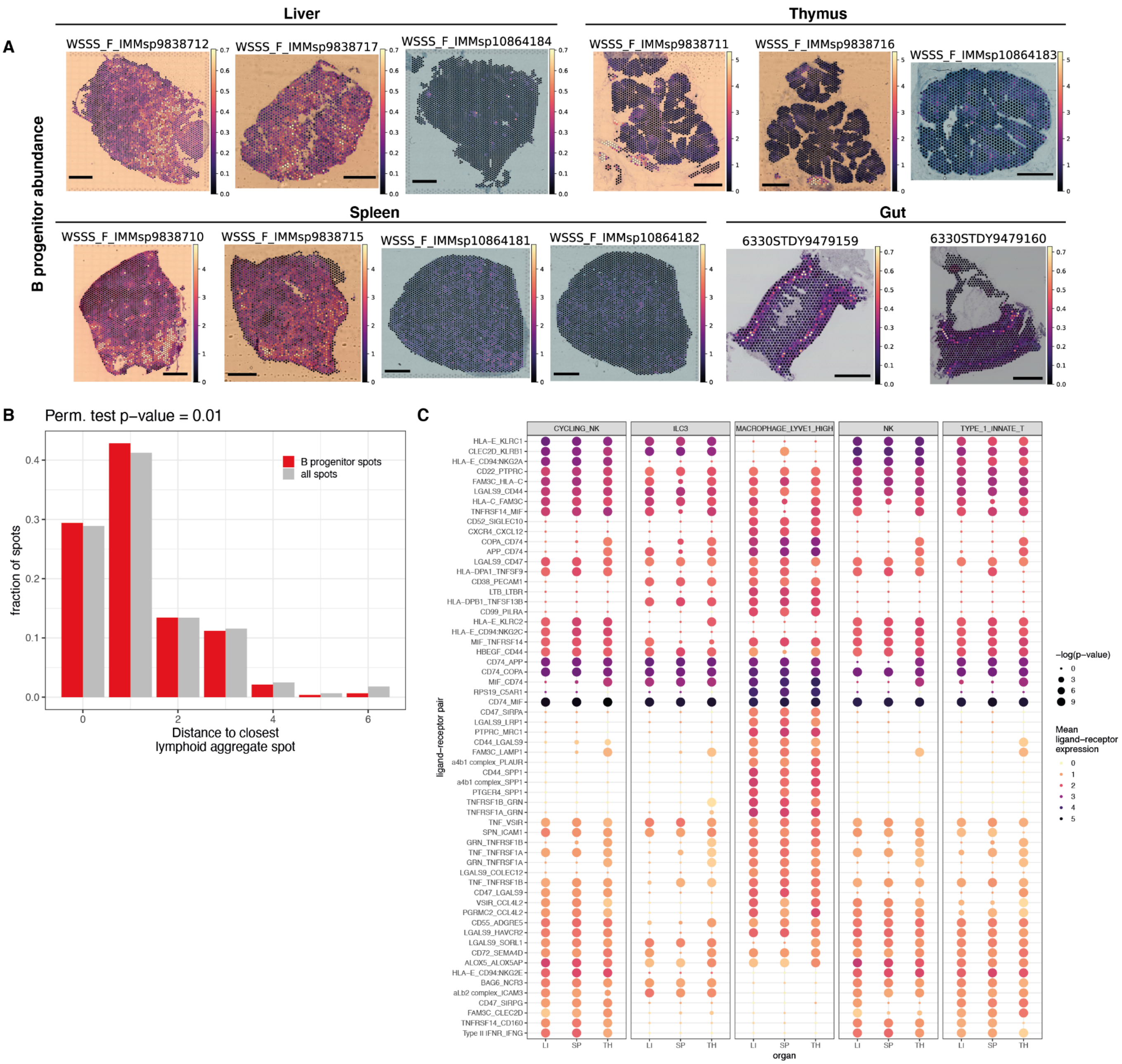

fig. S24. System-wide B lymphopoiesis. (**A**) Sum of abundances of B progenitor cell populations in spatial transcriptomics slides estimated with cell2location (scale bar: 1 mm). (**B**) Distribution of distance to the closest spot assigned as splenic lymphoid aggregate microenvironment from spots containing B cell progenitors (red) and from all analyzed spots (gray). Distance is measured as Euclidean distance of spots in spatial coordinates. We test if the distance to lymphoid aggregates is significantly smaller in B cell progenitor spots compared to other spots with a permutation test (5000 samples). Spots were assigned to the lymphoid aggregate microenvironment if the NMF factor value for the microenvironment (Fig. 1D, “B cell zone”) was above the 95% quantile for that slide. We considered spots to contain B cell progenitors if the sum of abundances of the B cell progenitors was above the 95% quantile for that slide. (**C**) Predicted cell–cell interactions between B cell progenitors and colocalizing cell types (ILC3, LYVE1^{hi} macrophage, NK cells, cycling NK cells and type 1 innate T cells, excluding LMPP_MLP as they are likely to be upstream progenitors of B lineage progenitors) from CellPhoneDB across liver (LI), spleen (SP) and thymus (TH). The first gene in each ligand-receptor pair is expressed in B cell progenitors and the second in the

interacting cell type. The color represents the average expression values of the ligand and receptor within their corresponding cell types, and the size represents −log(*P*-value). In addition to the previously described CXCL12–CXCR4 interaction in murine studies (*102*, *103*), our analysis identified many additional novel interactions that may inform efforts to generate and engineer B cells in vitro.

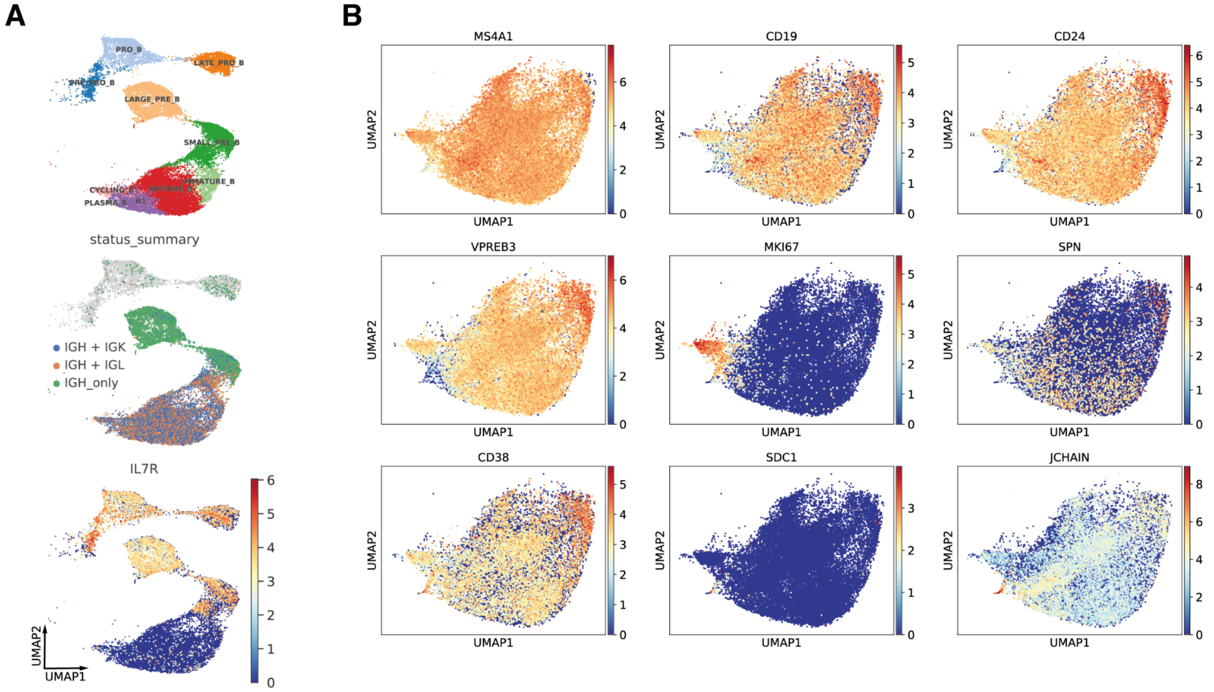

fig. S25. Characterization of B cells. (**A**) Close-up view of B cell populations on the UMAP embedding of all lymphoid cells (as shown in fig. S4I), colored by annotated cell population identity (top), status summary of cells expressing productive heavy (IGH) and/or light chains (IGK or IGL) of BCR from single-cell BCR sequencing (middle), and colored by *IL7R* expression pattern (bottom). (**B**) Marker gene expression patterns overlaid onto the same UMAP plot in Fig. 5A. Immature B cells were characterized by higher expression of *CD19*, *CD24*, and *VPREB3*. Mature B, cycling B, plasma B, and putative B1 cells expressed *MS4A1* except plasma B (*MS4A1*^{lo} and expressing *CD38*, *SDC1*, and *JCHAIN*). Cycling B cells were additionally marked with *MKI67*.

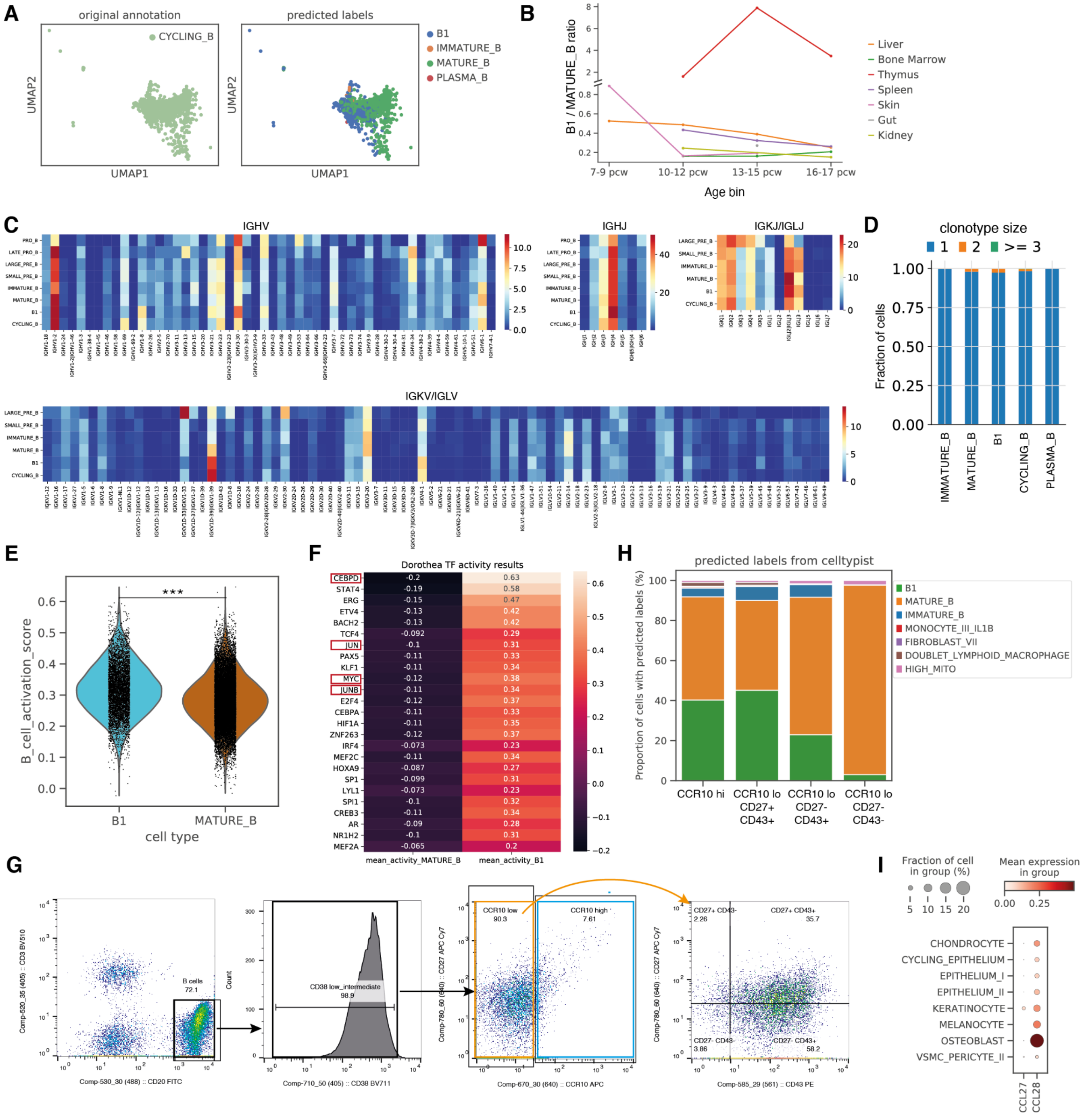
A
original annotation
CYCLING_B
UMAP2
UMAP1
predicted labels
B1
IMMATURE_B
MATURE_B
PLASMA_B
B
B1 / MATURE_B ratio
Age bin
7-9 pcw
10-12 pcw
13-15 pcw
16-17 pcw
Liver
Bone Marrow
Thymus
Spleen
Skin
Gut
Kidney
C
IGHV
IGHJ
IGKJ/IGLJ
IGKV/IGLV
PRO_B
LATE_PRO_B
LARGE_PRE_B
SMALL_PRE_B
IMMATURE_B
MATURE_B
B1
CYCLING_B
D
clonotype size
1
2
>= 3
Fraction of cells
IMMATURE_B
MATURE_B
B1
CYCLING_B
PLASMA_B
E
B_cell_activation_score
***
B1
MATURE_B
cell type
F
Dorothea TF activity results
CEBPD -0.2 0.63
STAT4 -0.19 0.58
ERG -0.15 0.47
ETV4 -0.13 0.42
BACH2 -0.13 0.42
TCF4 -0.092 0.29
JUN -0.1 0.31
PAX5 -0.11 0.33
KLF1 -0.11 0.34
MYC -0.12 0.38
JUNB -0.11 0.34
E2F4 -0.12 0.37
CEBPA -0.11 0.33
HIF1A -0.11 0.35
ZNF263 -0.12 0.37
IRF4 -0.073 0.23
MEF2C -0.11 0.34
HOXA9 -0.087 0.27
SP1 -0.099 0.31
LYL1 -0.073 0.23
SPI1 -0.1 0.32
CREB3 -0.11 0.34
AR -0.09 0.28
NR1H2 -0.1 0.31
MEF2A -0.065 0.2
mean_activity_MATURE_B
mean_activity_B1
G
B cells 72.1
CD38 low_intermediate 98.9
CCR10 low 90.3
CCR10 high 7.61
CD27+ CD43- 2.26
CD27+ CD43+ 35.7
CD27- CD43- 3.86
CD27- CD43+ 58.2
Comp-530_30 (488) :: CD20 FITC
Comp-520_35 (405) :: CD3 BV510
Comp-710_50 (405) :: CD38 BV711
Count
Comp-670_30 (640) :: CCR10 APC
Comp-780_60 (640) :: CD27 APC Cy7
Comp-585_29 (561) :: CD43 PE
H
predicted labels from celltypist
Proportion of cells with predicted labels (%)
B1
MATURE_B
IMMATURE_B
MONOCYTE_III_IL1B
FIBROBLAST_VII
DOUBLET_LYMPHOID_MACROPHAGE
HIGH_MITO
CCR10 hi
CCR10 lo CD27+ CD43+
CCR10 lo CD27- CD43+
CCR10 lo CD27- CD43-
I
Fraction of cell in group (%)
Mean expression in group
CHONDROCYTE
CYCLING_EPITHELIUM
EPITHELIUM_I
EPITHELIUM_II
KERATINOCYTE
MELANOCYTE
OSTEOBLAST
VSMC_PERICYTE_II
CCL27
CCL28

fig. S26. Characterization of putative B1 cells. (**A**) Close-up view of cycling B cell population on UMAP embedding of all lymphoid cells (as shown in fig. S4I), colored by original cell population annotation (left) and annotations predicted by logistic regression trained on all non-progenitor B cell subsets (right). This assigns specific cell identity to each of the cells within the cycling B cell group. The results are used in Fig. 5B. (**B**) Ratio of B1 cell number over mature B cell number in different organs across different gestational age bins. (**C**) Heat map showing the percentage of each BCR heavy (IGHV, IGHJ) and light chain (IGKV/IGLV, IGKJ/IGLJ) V and J gene segments present in different B cell subtypes. (**D**) Barplot of cell fractions with different clonotype size across different mature B cell subtypes. (**E**) Violin plot of B cell activation scores in B1 and mature B cells. Cells were scored according to expression values of all genes in the Gene Ontology B Cell Activation gene list. A significant difference in B cell activation scores was observed between B1 and mature B cells after controlling for donors and organs with linear regression (****P*-value$<10^{-10}$). (**F**) Heatmap of TF activity means in B1 and mature B cells. The color and the number represent the average TF activity estimated by DoRothEA (*94*). Only the top 25 TFs that had significantly higher activities in B1 cells were shown here. TFs in TNF-α and NF-κB signaling pathway (*CEBPD*, *JUN*, *MYC*, *JUNB*) are boxed in red. (**G**) Representative flow cytometry plots showing the sorting strategy for the ELISpot experiment shown in Fig. 5E. The splenic B cells were gated from live single cells which were $CD3^{-}CD20^{+}$, excluding the top 1% of cells expressing the highest level of CD38 to avoid plasma cells (which should also be CD20 low and therefore not gated in), and split the rest into four fractions: $CCR10^{hi}$, $CCR10^{lo}CD27^{+}CD43^{+}$, $CCR10^{lo}CD27^{-}CD43^{+}$, and $CCR10^{lo}CD27^{-}CD43^{-}$. We then performed an ELISpot experiment on all four fractions without any stimulation. (**H**) Barplot of cell proportions with different predicted annotations in the four sort fractions: $CCR10^{hi}$, $CCR10^{lo}CD27^{+}CD43^{+}$, $CCR10^{lo}CD27^{-}CD43^{+}$, and $CCR10^{lo}CD27^{-}CD43^{-}$. (**I**) Dot plot showing gene expressions of *CCL27* and *CCL28* within the stromal cell populations. Only cell types with log normalized expression of *CCL27* or that of *CCL28* above 0.05 are shown here.

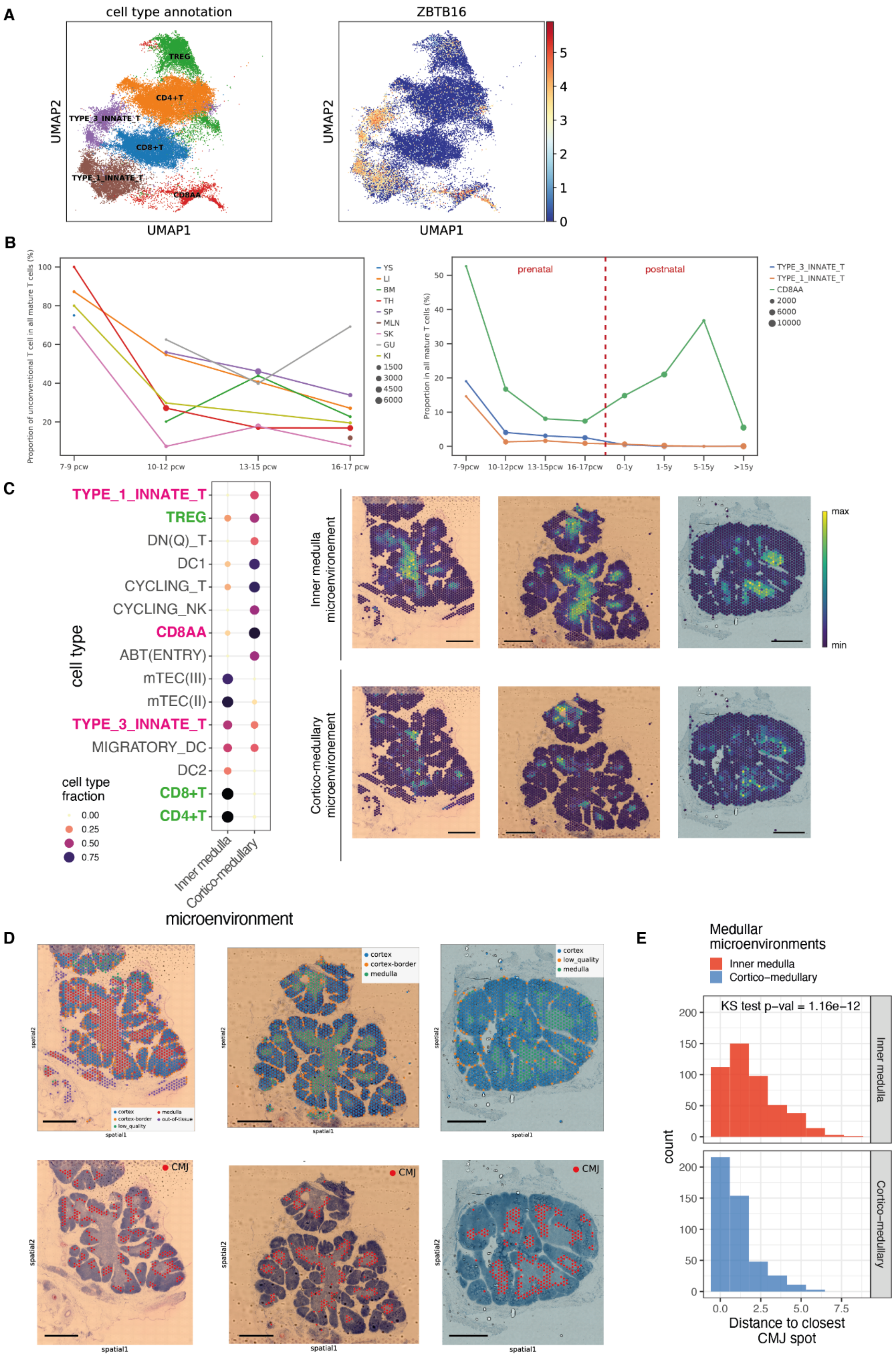

A
cell type annotation
ZBTB16
UMAP2
UMAP1
TREG
CD4+T
TYPE_3_INNATE_T
CD8+T
TYPE_1_INNATE_T
CD8AA
B
Proportion of unconventional T cell in all mature T cells (%)
7-9 pcw
10-12 pcw
13-15 pcw
16-17 pcw
YS
LI
BM
TH
SP
MLN
SK
GU
KI
prenatal
postnatal
Proportion in all mature T cells (%)
TYPE_3_INNATE_T
TYPE_1_INNATE_T
CD8AA
C
TYPE_1_INNATE_T
TREG
DN(Q)_T
DC1
CYCLING_T
CYCLING_NK
CD8AA
ABT(ENTRY)
mTEC(III)
mTEC(II)
TYPE_3_INNATE_T
MIGRATORY_DC
DC2
CD8+T
CD4+T
cell type
cell type fraction
Inner medulla
Cortico-medullary
microenvironment
Inner medulla microenvironement
Cortico-medullary microenvironement
max
min
D
cortex
cortex-border
medulla
low_quality
out-of-tissue
spatial1
spatial2
CMJ
E
Medullar microenvironments
Inner medulla
Cortico–medullary
KS test p–val = 1.16e–12
count
Distance to closest CMJ spot

fig. S27. Distribution of unconventional T cells across gestation and in thymic tissue. (**A**) Left: Close-up view of mature T cells on UMAP embedding of all NK/T cells (as shown in fig. S4J). Type 1 innate T, type 3 innate T and CD8AA contain both αβT cells and γδT cells. Right: *ZBTB16* expression pattern overlaid onto the same UMAP plot. (**B**) Left: proportion of unconventional T cells in all mature T cells in different organs across different gestational age bins. Point size represents the number of mature T cells in a given organ within that age bin. Lines and points are color-coded by organs (YS: yolk sac; LI: liver; BM: bone marrow; TH: thymus; SP: spleen; MLN: mesenteric lymph node; SK: skin; GU: gut; KI: kidney). Right: proportion of each unconventional T cell subtype in all mature T cells in thymus across different age groups, using dataset from (*7*). Point size represents the number of mature T cells in thymus within that age bin. (**C**) Left: cell type contributions to medullary microenvironments containing mature T cells in thymus, identified with non-negative matrix factorization of spatial cell type abundances estimated with cell2location. The color and the size of the dots represent the relative fraction of the cell population assigned to the microenvironment. Unconventional T cell types are highlighted in magenta. Conventional T cell types are highlighted in green. Right: spatial locations of medullary microenvironments on different thymic slides, with the color representing the weighted contribution of each microenvironment to each spot (scale bar: 1 mm). (**D**) Top: annotation of tissue regions on Visium spots inferred by clustering of H&E image features. Bottom: location of interface region between cortex and medulla region, which we consider as the cortico-medullary junction (CMJ) (scale bar: 1 mm). (**E**) Histogram of Euclidean distance to the nearest CMJ spot for spots assigned to inner-medulla microenvironment (red) or cortico-medullary microenvironment (blue). Spots were assigned to a microenvironment if the NMF factor value for the microenvironment (see C) was above the 90% quantile. The *P*-value for the Kolmogorov–Smirnov test comparing the two distributions is reported.

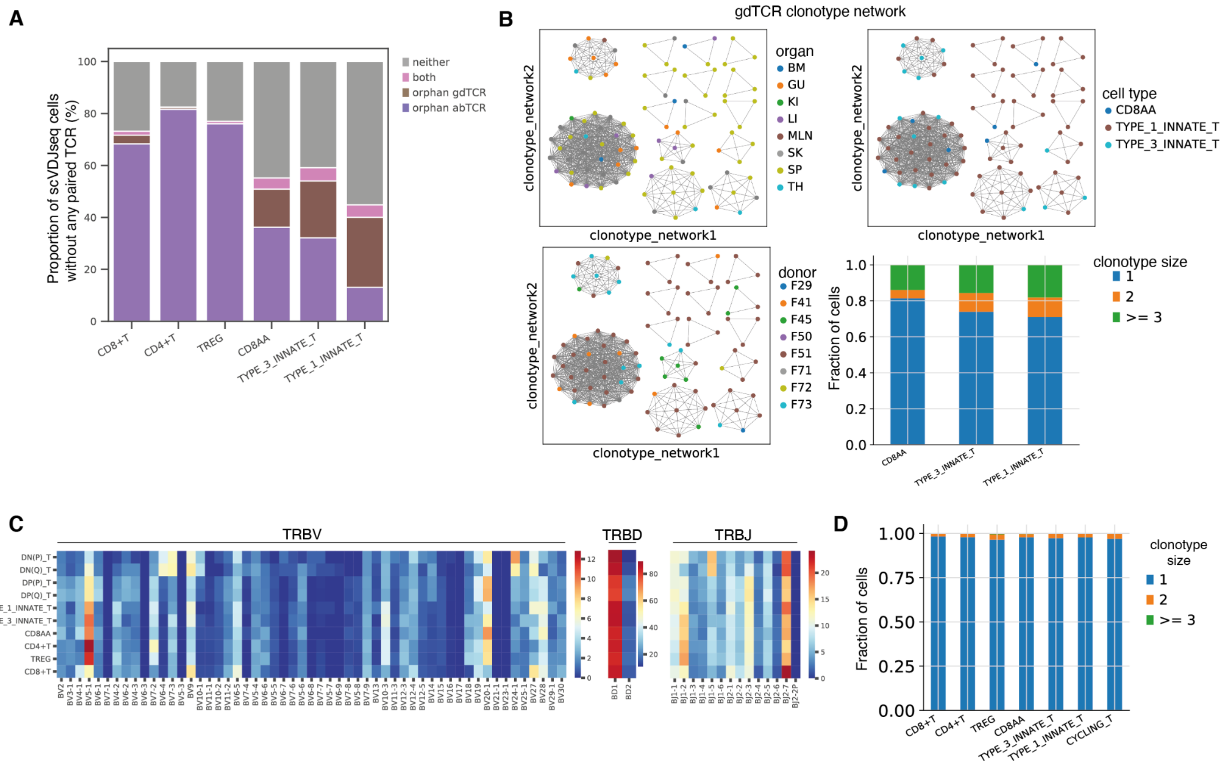

fig. S28. T cell receptor (TCR) sequence analysis. (**A**) Proportions of cells without any paired TCR expressing orphan γδTCR, i.e. one of γ or δ chain, or orphan αβTCR, i.e. one of α or β chain, or both or neither. (**B**) Top left: clonotype network graph of γδTCR. Each fully connected subnetwork represents a clonotype cluster, with each dot representing a cell. The dots are color-coded by organs (YS: yolk sac; LI: liver; BM: bone marrow; TH: thymus; SP: spleen; MLN: mesenteric lymph node; SK: skin; GU: gut; KI: kidney). Top right: γδTCR clonotype network graph color-coded by cell types. Bottom left: γδTCR clonotype network graph color-coded by donors. Bottom right: barplot of cell fractions with different γδTCR clonotype size across different unconventional T cell subtypes. (**C**) Heat map showing the percentage of each TRBV, TRBD and TRBJ gene segment present in different T cell subtypes. (**D**) Barplot of cell fractions with different clonotype size across different mature T cell subtypes.

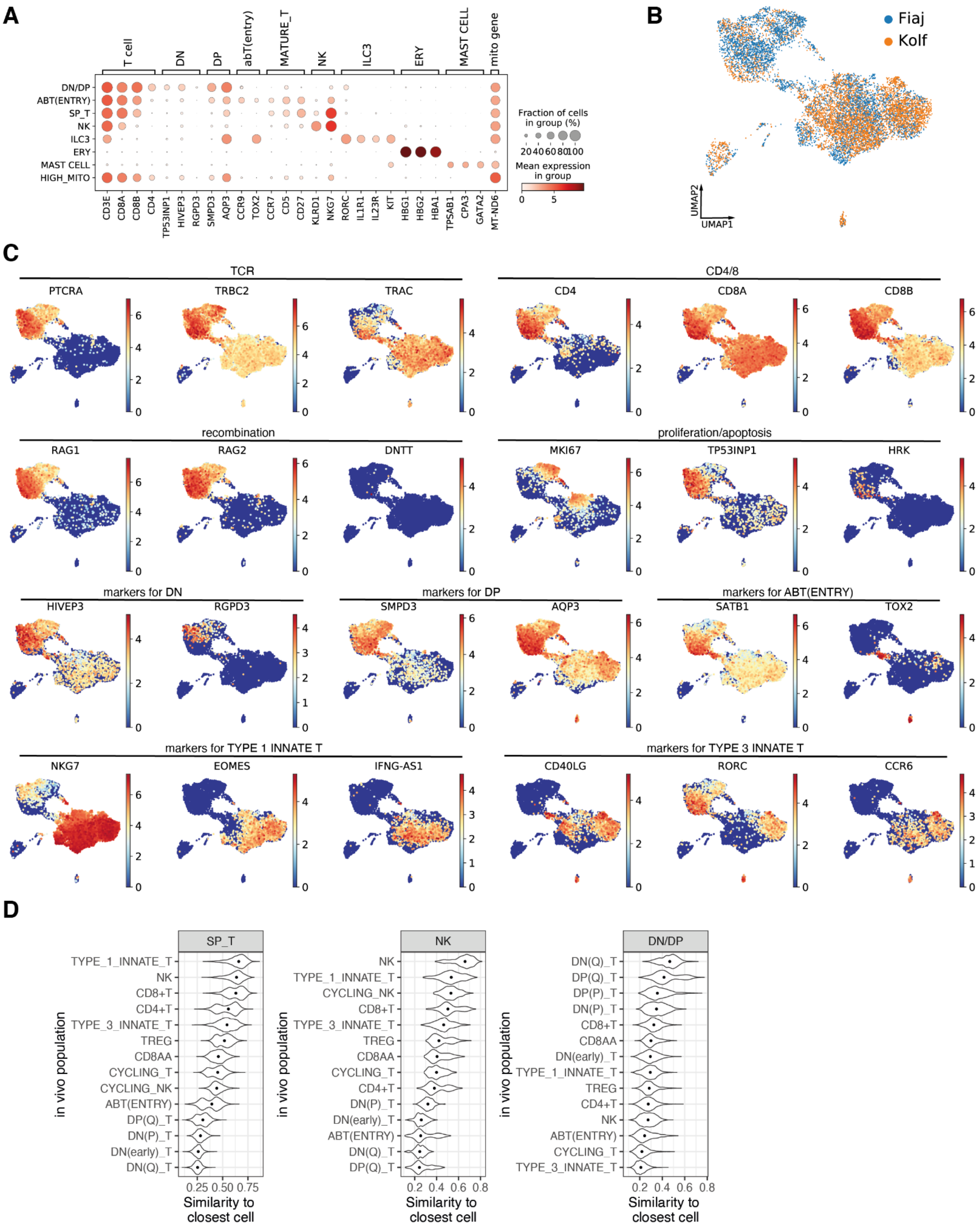

fig. S29. Analysis of artificial thymic organoids scRNA-seq data. (**A**) Dot plot of marker genes for ATO cell populations. (**B**) Cells colored by the starting iPSC lines in ATO overlaid on UMAP embedding shown in Fig. 6F. (**C**) Expression of T cell marker genes in ATO overlaid on UMAP embedding shown in Fig. 6F. (**D**) Violin plots of similarity to closest in vivo cell (*x*-axis) for each cell type in the in vivo dataset (*y*-axis) for in vitro single-positive T

cells (SP_T), NK cells and developing T cells (DN/DP). Similarities are calculated in the scVI latent space for lymphoid cells after mapping in vitro cells with scArches.

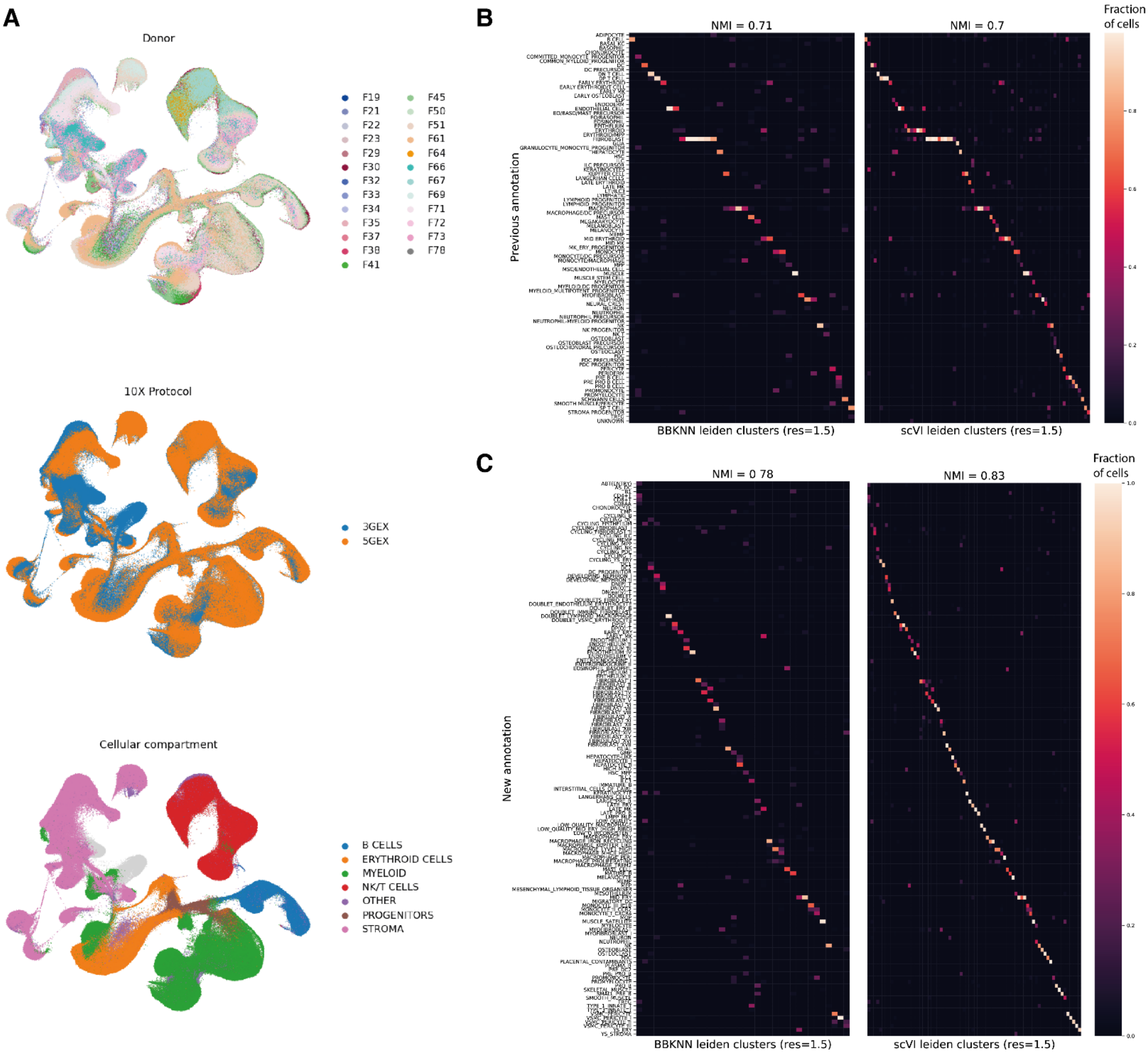

fig. S30: Comparison across data integration methods. (**A**) UMAP embeddings of scRNA-seq profiles after data integration with BBKNN colored by (top to bottom): 10X library prep protocol, donor ID, cellular compartment. (**B**) heat map of confusion matrices between Leiden clusters and previously annotated cell type labels, with clustering on BBKNN integration (left, 37 clusters) or scVI integration (right, 75 clusters) of the full dataset (clustering resolution = 1.5). (**C**) heat map of confusion matrices between Leiden clusters and newly annotated cell type labels, with clustering on BBKNN integration (left, 37 clusters) or scVI integration (right, 75 clusters) of the full dataset (clustering resolution = 1.5). For each confusion matrix, the normalized mutual information (NMI) score between cluster labels and annotation labels is shown.

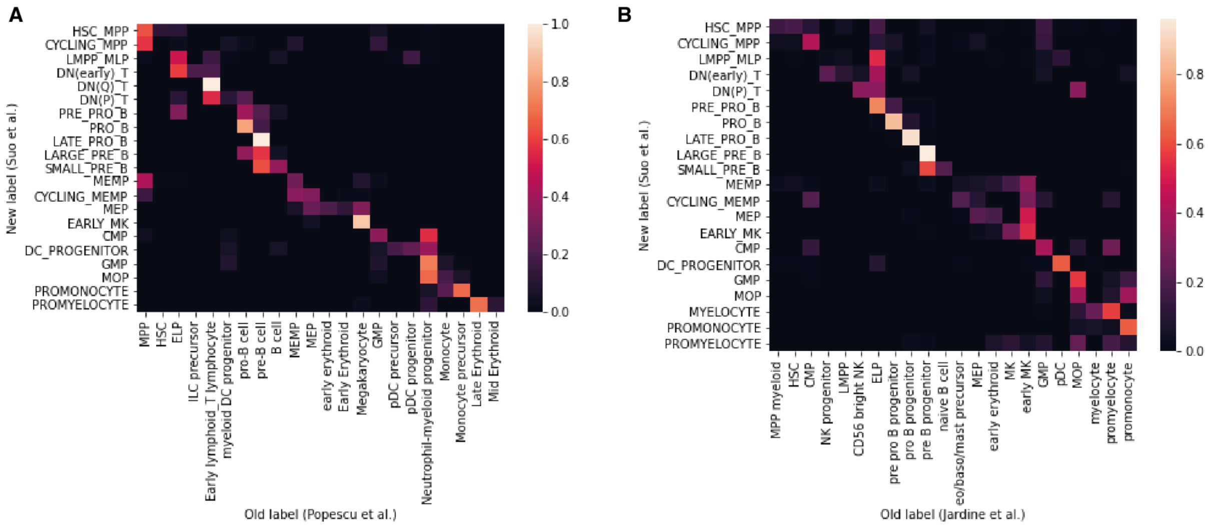

fig. S31: Agreement between annotations of progenitor cells in this study and annotations from fetal bone marrow atlas (*11*)**.** (**A**) Confusion table between annotations of progenitor cells in bone marrow in this study and the annotations for the same cells in fetal bone marrow atlas (*11*). The color is proportional to the fraction of cells in the new population with a given old label. Only bone marrow cells for which a previous annotation was available are shown (32,274/46,448 bone marrow cells). (**B**) Confusion table between annotations of progenitor cells in bone marrow in this study and the annotations for the same cells in fetal liver atlas (*3*). The color is proportional to the fraction of cells in the new population with a given old label. Only liver cells for which a previous annotation was available are shown (11,330/26,377 liver cells).

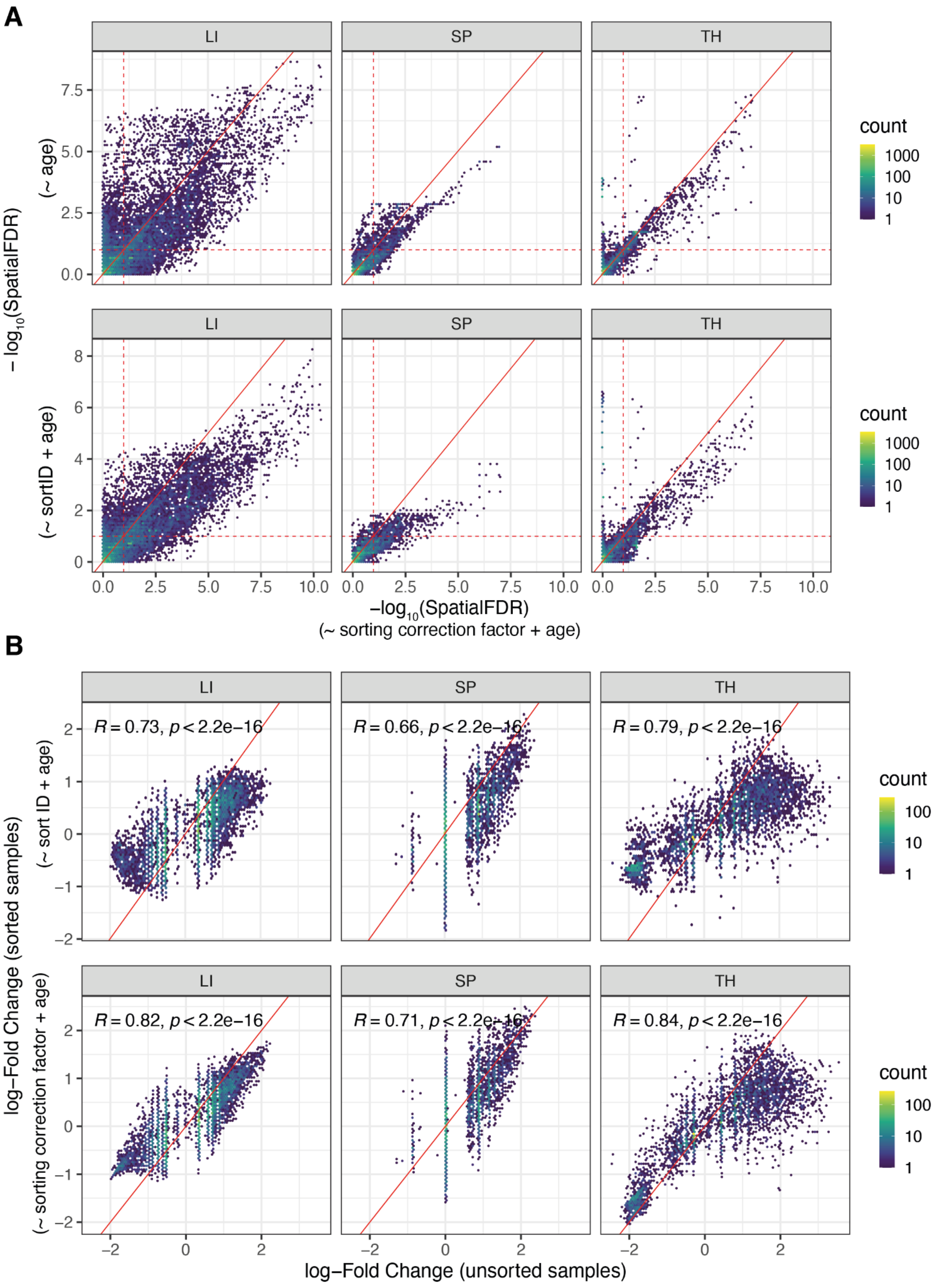
A
LI
SP
TH
count
1000
100
10
1
− log10(SpatialFDR)
(~ age)
(~ sortID + age)
−log10(SpatialFDR)
(~ sorting correction factor + age)
B
R = 0.73, p < 2.2e−16
R = 0.66, p < 2.2e−16
R = 0.79, p < 2.2e−16
R = 0.82, p < 2.2e−16
R = 0.71, p < 2.2e−16
R = 0.84, p < 2.2e−16
log−Fold Change (sorted samples)
(~ sort ID + age)
(~ sorting correction factor + age)
log−Fold Change (unsorted samples)

fig. S32: Validation of quantification of FACS effect on cell abundances in Milo neighborhoods. (**A**) Scatter plot of SpatialFDR (in $-\log_{10}$ scale) estimated in test for differential abundance across gestational age, regressing out continuous FACS factor (*x*-axis), without regressing out FACS effect (*y*-axis, top) and regressing out FACS protocol label ($CD45^{+}$/$CD45^{-}$/unsorted) (*y*-axis, bottom). Results for the test on cells from liver (LI), spleen (SP) and thymus (TH) are shown. The dotted red lines indicate the significance threshold of 10% SpatialFDR. (**B**) Scatterplot of log-fold change estimated in test for differential abundance across gestational age testing on the subset of unsorted samples (*x*-axis) and on FACS-isolated samples (y-axis), regressing out the FACS protocol label (top) or the FACS factor (bottom).

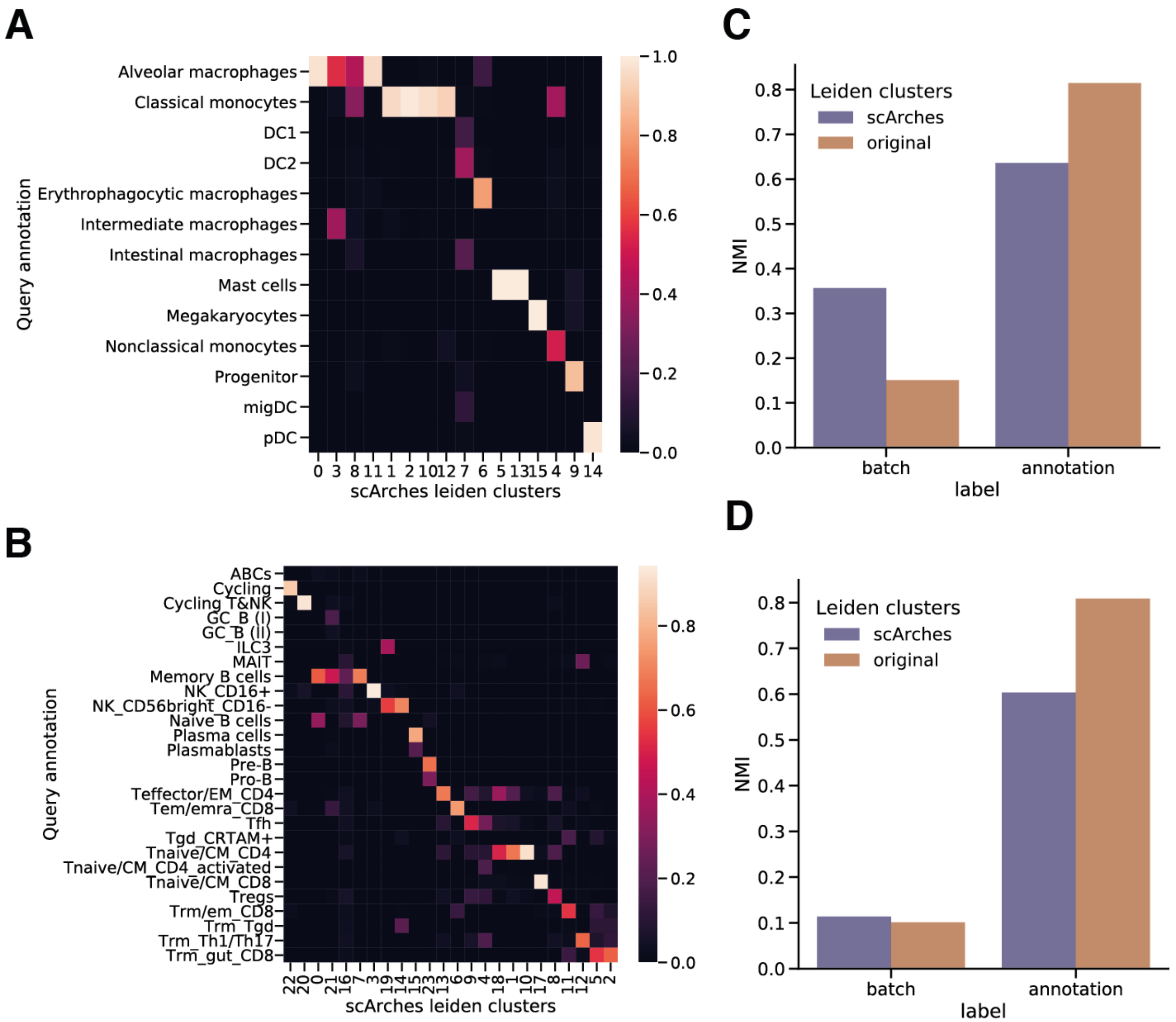

fig. S33: Validation of biological conservation after scArches mapping of adult cells to prenatal reference. (**A** and **B**) Heat maps of confusion matrix between adult immune cell annotations (*21*) and Leiden clusters obtained with latent dimensions after mapping adult cells on the prenatal reference with scArches, for myeloid cells (A) and lymphoid cells (B). (**C** and **D**) Barplots of normalized mutual information (NMI) between technical batch or cell type annotation and Leiden clusters from scArches mapping or the clusters in the original BBKNN embedding *(21*).

Supplementary Tables

table S1: Table_S1.csv (separate file)
Differential expression analysis results for test on gestation-stage specific macrophage and mast cells neighborhoods

table S2: Table_S2.csv (separate file)
Differential expression analysis results for comparison of bone marrow and peripheral CCR2hi monocytes

table S3: Table_S3.csv (separate file)
Differential expression analysis results for test on gestation-stage specific NK cells neighborhoods

table S4: Table_S4.csv (separate file)
Differential expression analysis results for comparison of thymic and peripheral mature T cells

table S5: Table_S5.csv (separate file)
Cell types included in each lineage progenitors shown in Fig. 4A.

table S6: Table_S6.csv (separate file)
Results from the ELISpot experiment.

table S7: Table_S7.csv (separate file)
Metadata for sequenced samples.

[File] Prefix for raw sequencing files
[Organ] YS: yolk sac; LI: liver; BM: bone marrow; TH: thymus; SP: spleen; MLN: mesenteric lymph node; SK: skin; GU: gut; KI: kidney
[Sort_id] Sorting scheme used for each sample. CD45P: $CD45^{+}$, CD45N: $CD45^{-}$, CD137: $CD3^{+}$ and $CD137^{hi}$; MAIT: $CD3^{+}CD25^{hi}$, TOT: Unsorted
[Age] Age of donor (post conceptional weeks)
[Method] 3GEX: 10X 3' chemistry gene expression profiling, 5GEX: 10X 5' chemistry gene expression profiling
[Donor] Unique ID assigned for each donor
[Sample]: Sample description (DonorID-Organ-Sort-File)
[Sex] Sex of donor
[Processing_method] Protocol used for dissociation
[Anatomical_region] Region of dissection
[N_cells] Total number of cells in sample (post-QC)
[Termination_method] Termination of pregnancy method

table S8: Table_S8.csv (separate file)
Primers used in targeted γδTCR amplification.

table S9: Table_S9.csv (separate file)
Metadata for samples profiled with Visium 10X spatial transcriptomics.

[File] Prefix for raw sequencing files

[Image_id] Unique ID assigned to each image for H&E staining
[Organ] Sampled tissue
[Donor] Unique ID assigned for each donor
[Slide_number] Unique ID for Visium 10X slide (experimental batch)
[Visium_area_id] Unique ID for position of tissue on Visium 10X slide
[Age] Age of donor (post conceptional weeks)
[Sex] Sex of donor
[Digestion time] Time of digestion in tissue optimization (minutes)
[Termination_method] Termination of pregnancy method
